# Supplementary figures and images for: Essential Role of CFAP53 in Sperm Flagellum Biogenesis (part 1 of 3)
Source: Front Cell Dev Biol. 2021 May 28;9:676910. doi: 10.3389/fcell.2021.676910 (PMC8195676; doi:10.3389/fcell.2021.676910)

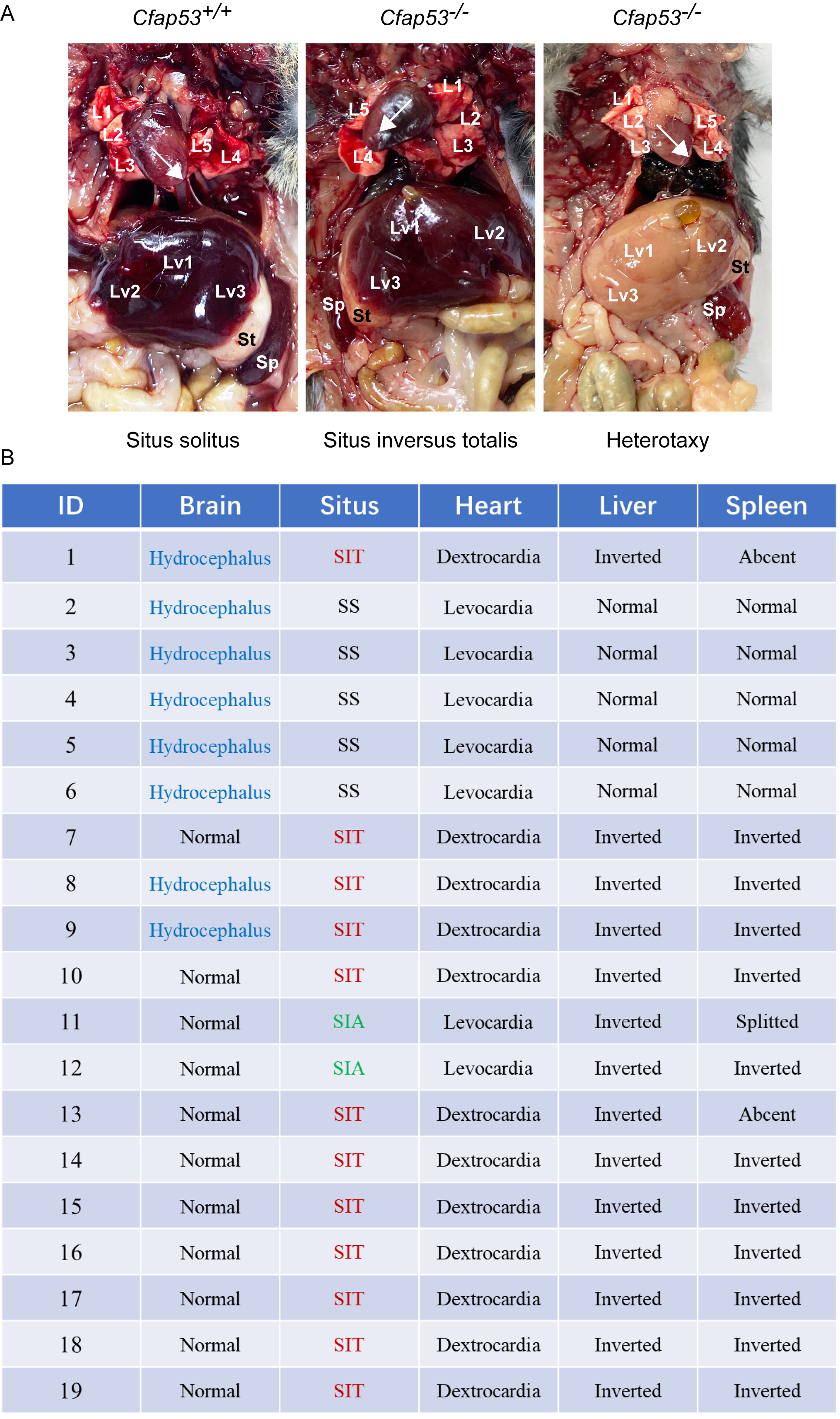

Supplement: Supplementary Figure 1 — Left–right body asymmetry defects and hydrocephalus in Cfap53–/– mice. (A) Cfap53–/– mice presented with situs inversus totalis (SIT), situs inversus abdominalis (SIA), and situs solitus (SS). L1-5 (white numbers): lung lobes, Lv1-3 (white numbers): liver lobes, St: stomach, Sp: spleen. (B) Summary of the phenotypes detected in Cfap53–/– mice. [file Image_1.TIF]

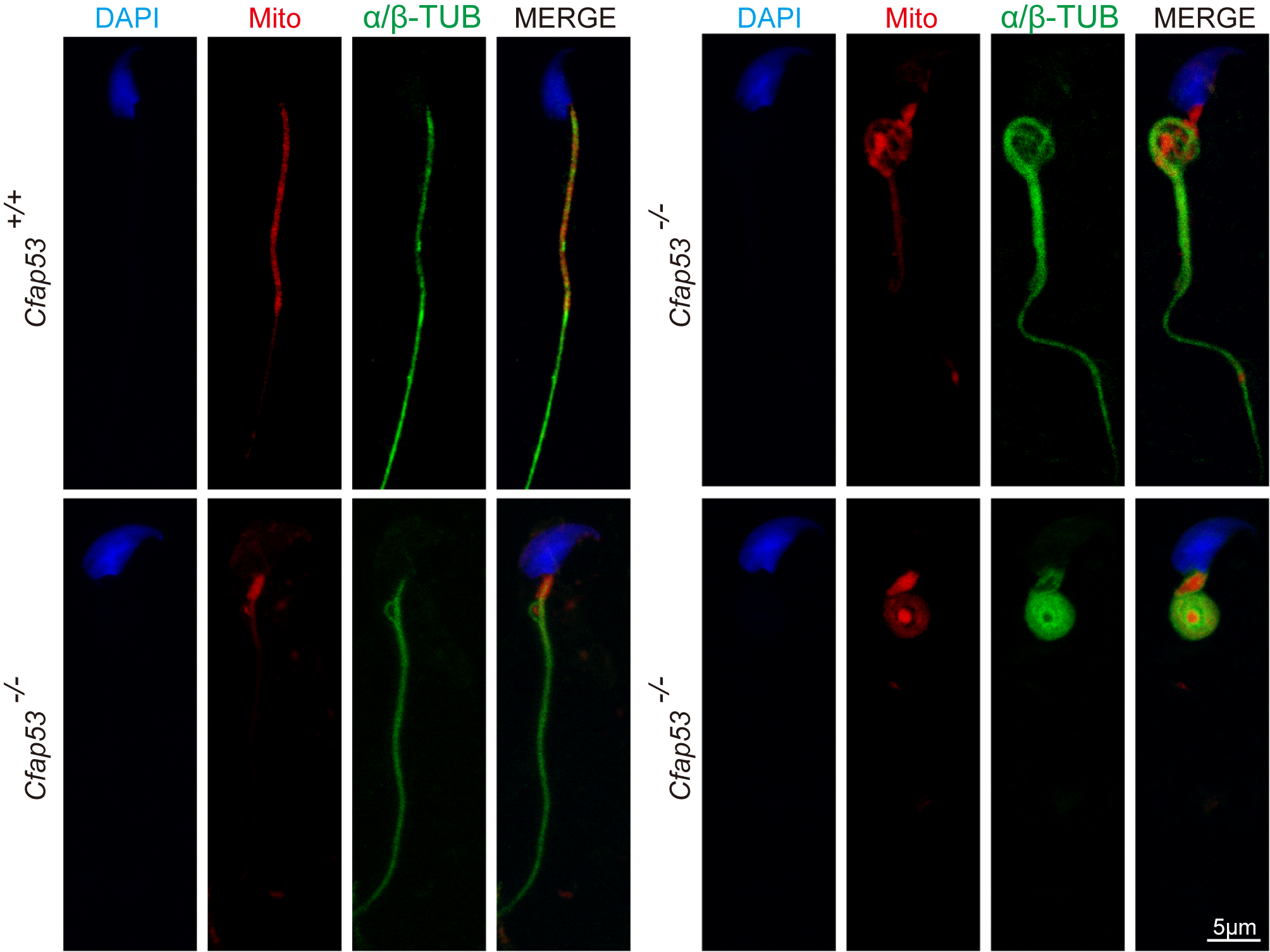

Supplement: Supplementary Figure 2 — Mitochondrial sheath defects in Cfap53–/– spermatozoa. The immunofluorescence analysis for α/β-tubulin (green) and MitoTracker (red) was performed in Cfap53+/+ and Cfap53–/– spermatozoa. The nucleus was stained with DAPI (blue). [file Image_2.TIF]

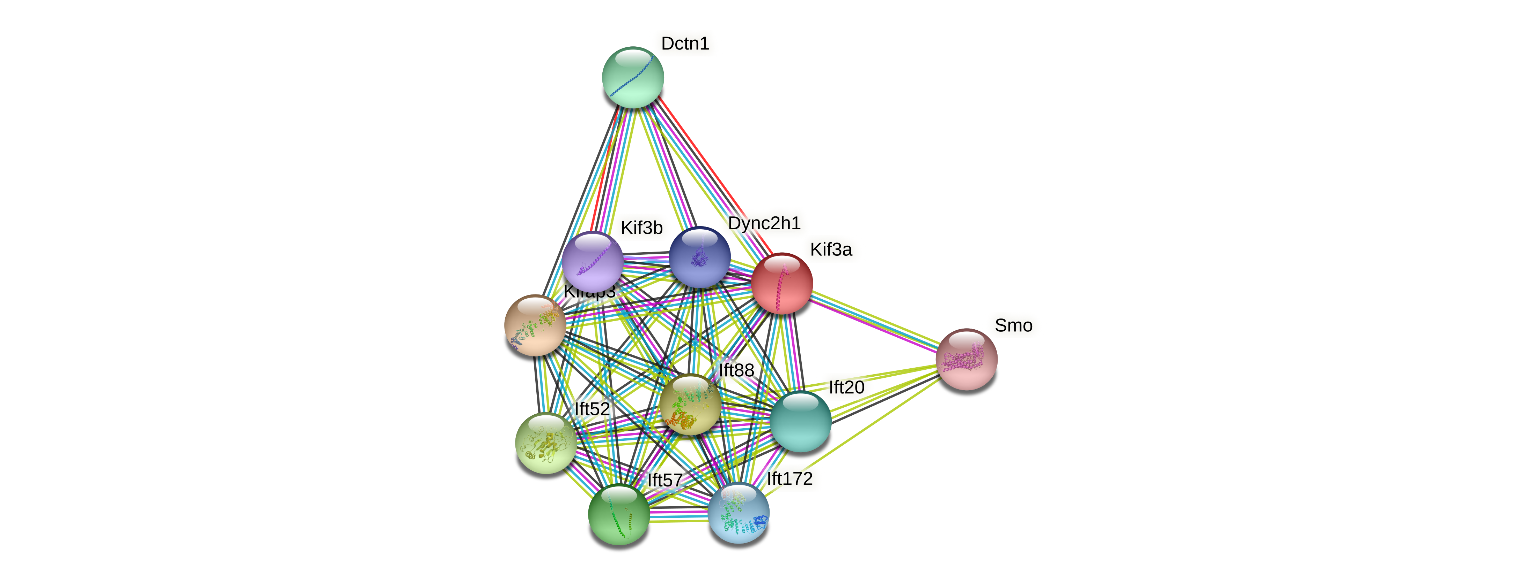

Supplement: Supplementary Figure 3 — Predicted KIF3A partners. STRING version 11 predicted the protein-protein interaction networks. [file Image_3.TIF]

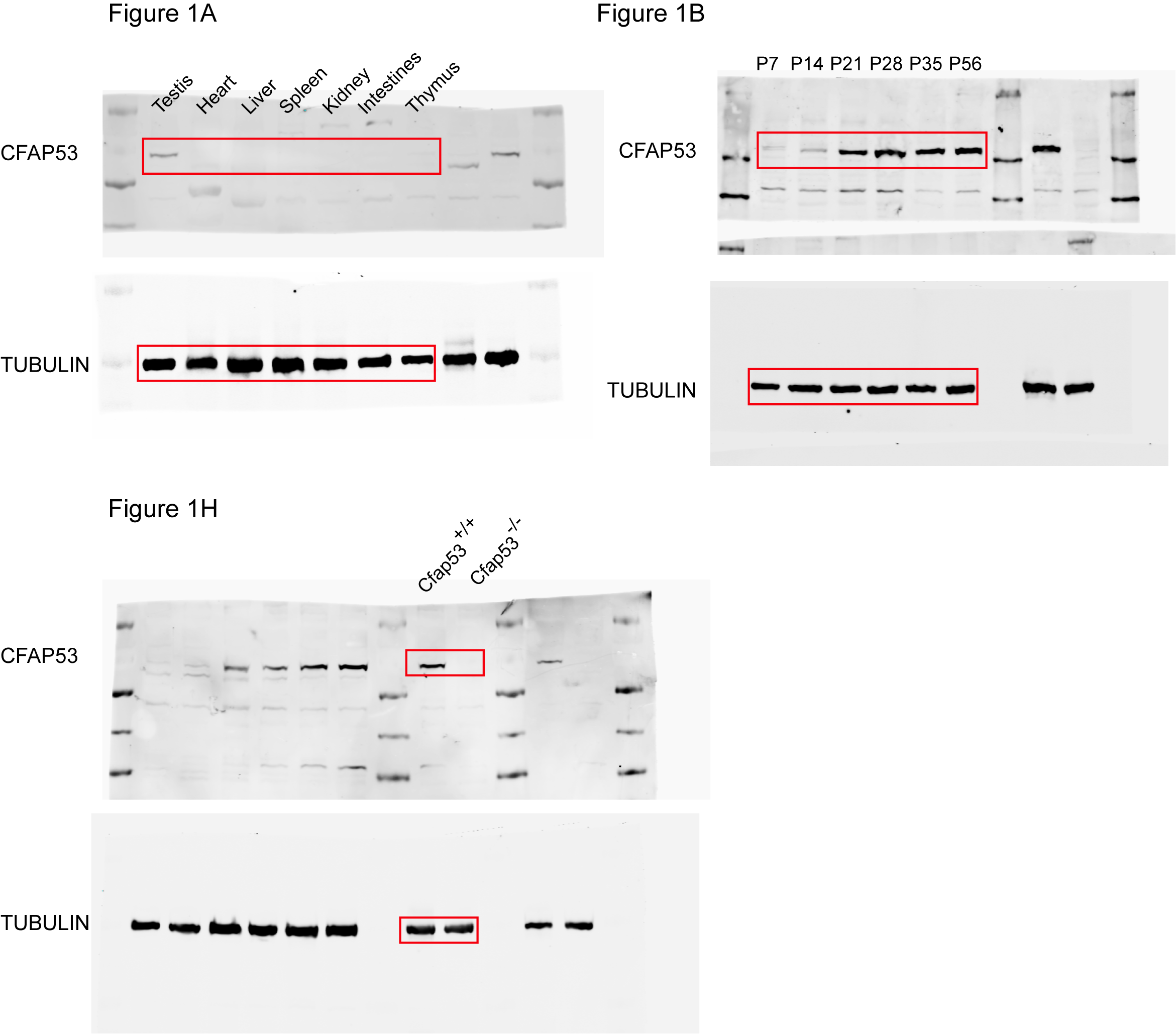

Supplement: Supplementary file 4 [file Data_Sheet_1.ZIP › original blots results/figure 1A 1B 1H.tif]

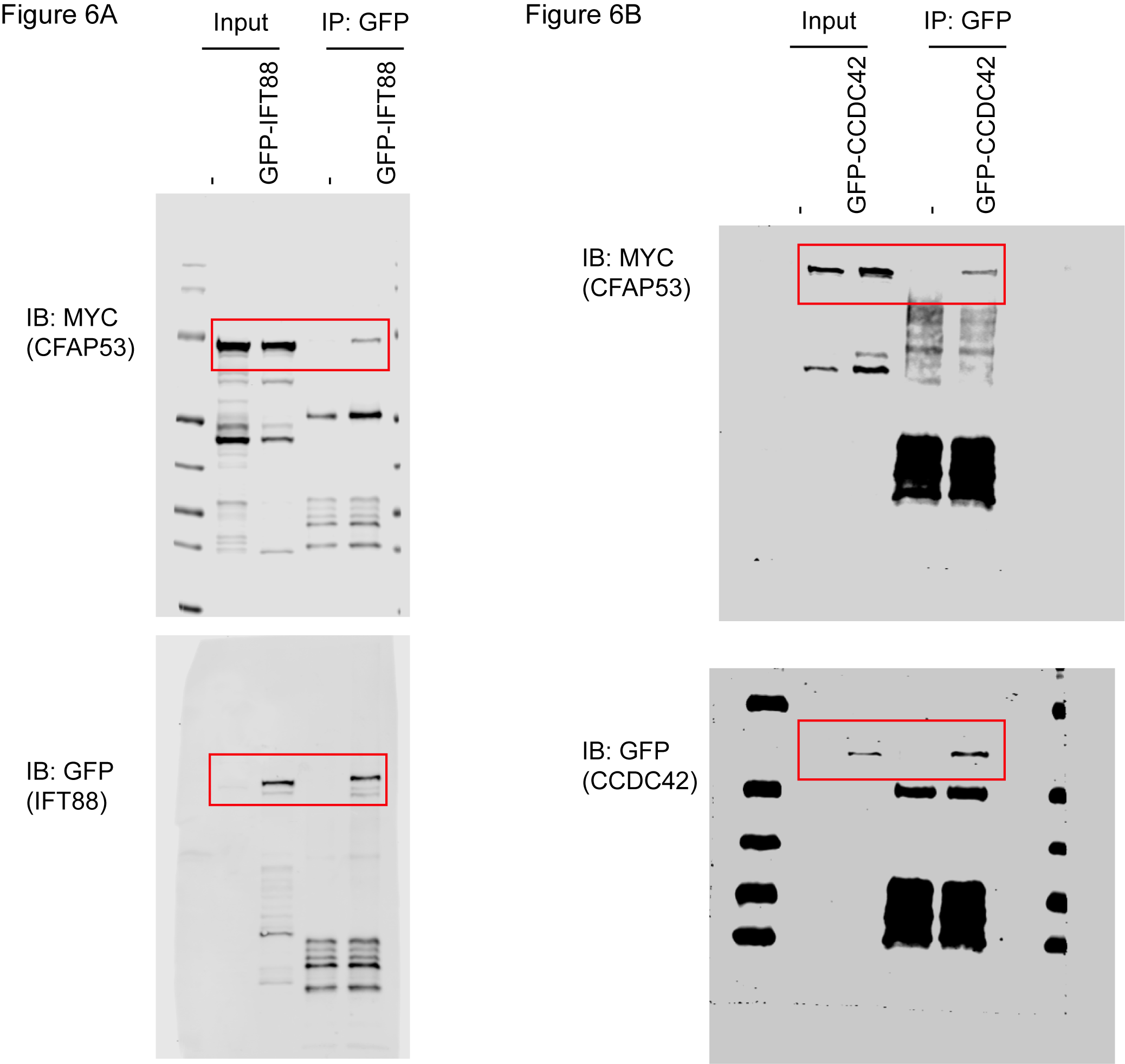

Supplement: Supplementary file 4 [file Data_Sheet_1.ZIP › original blots results/figure 6A 6B.tif]

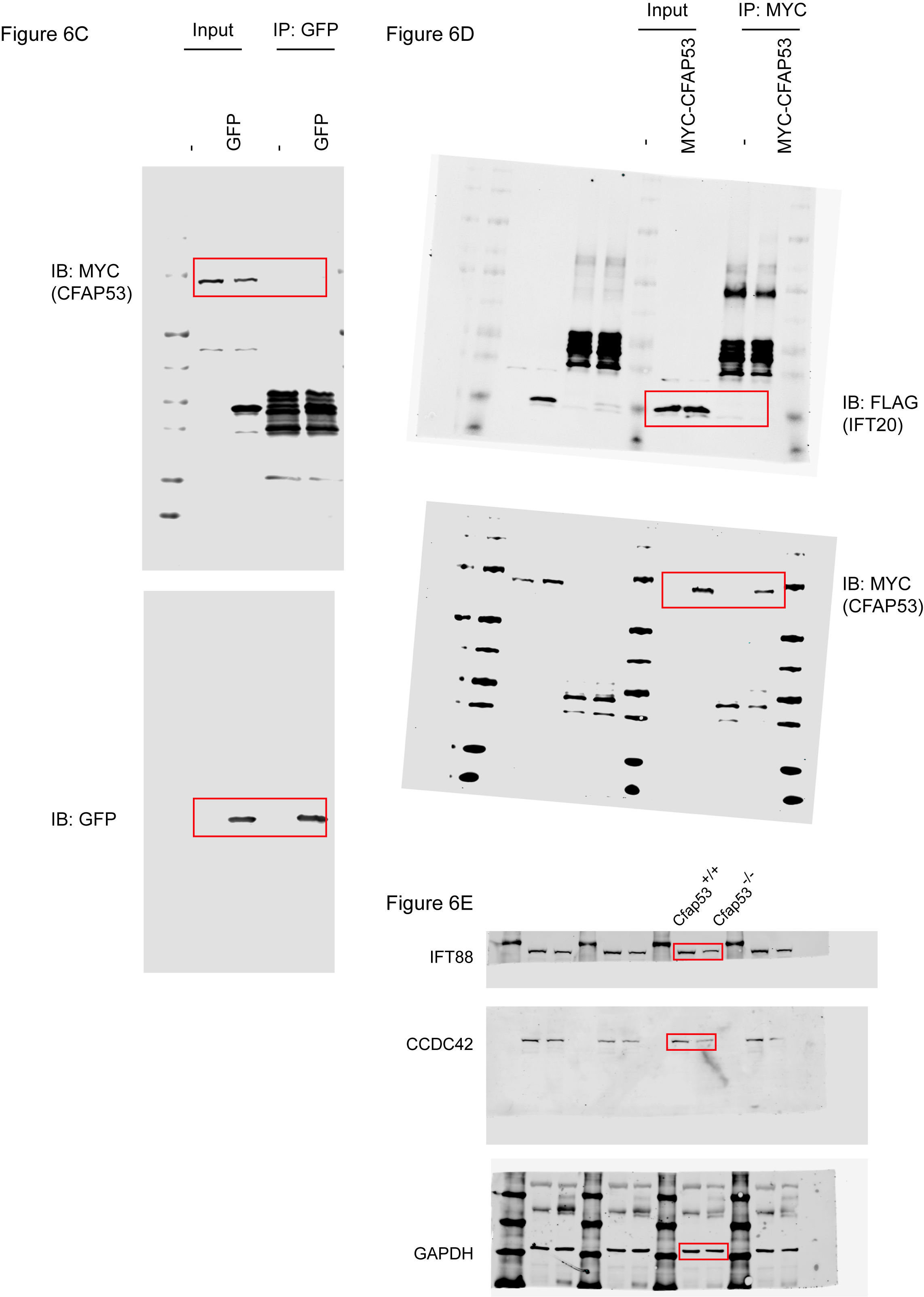

Supplement: Supplementary file 4 [file Data_Sheet_1.ZIP › original blots results/figure 6C 6D 6E.tif]

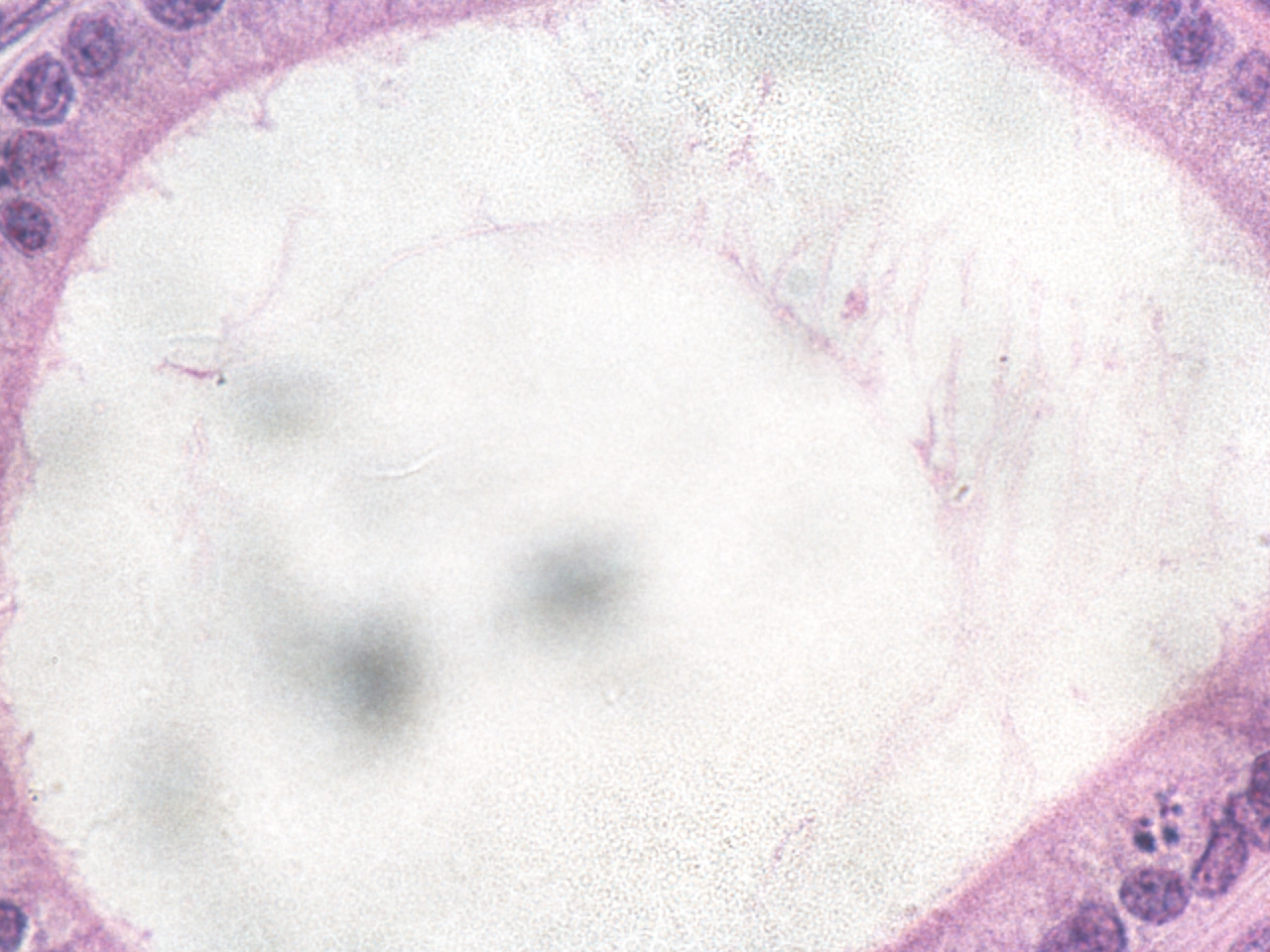

Supplement: Supplementary file 5 [file Data_Sheet_2.ZIP › original microscopy Fig2/Fig2A/KO FU 1000 .jpg]

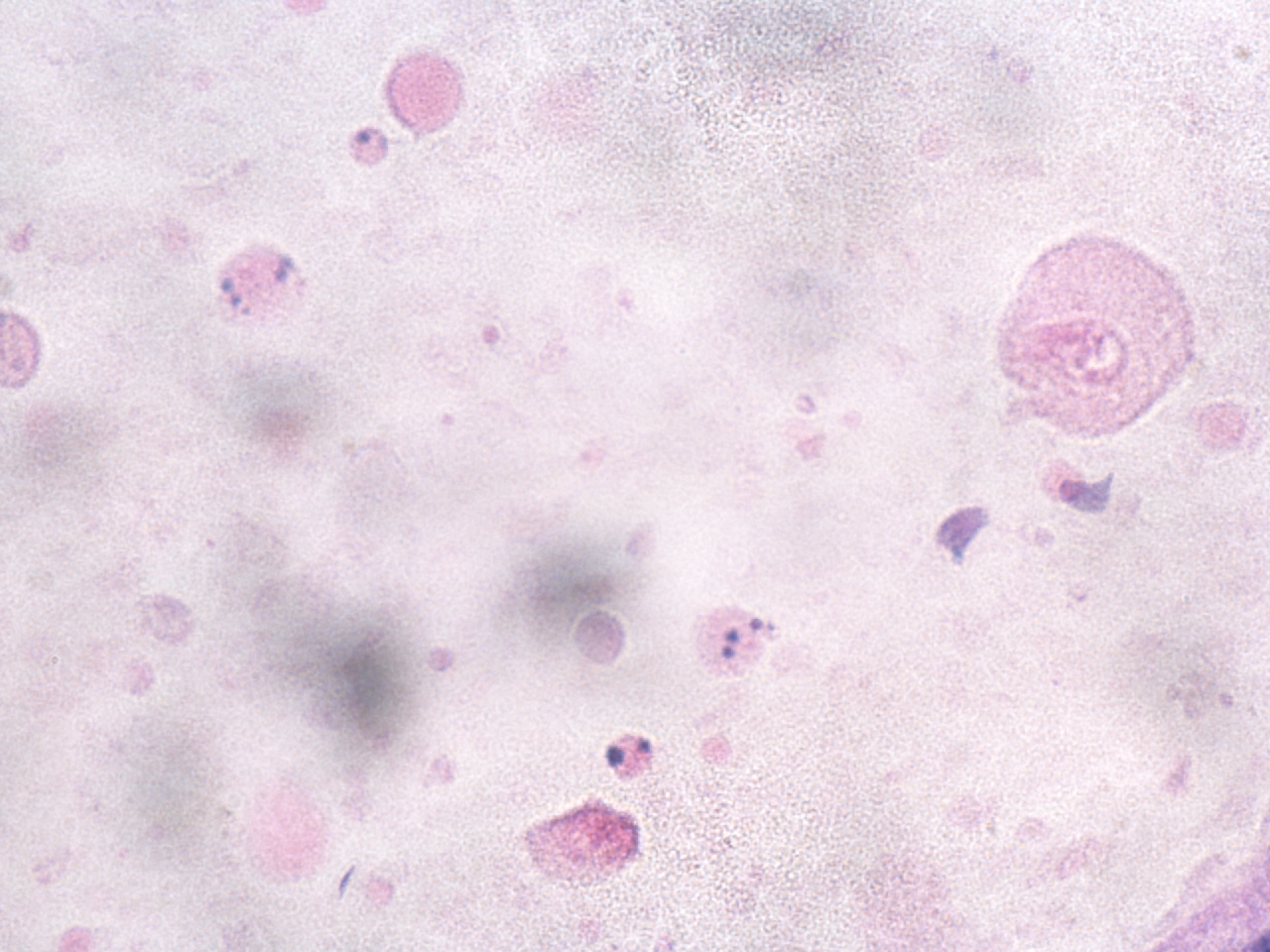

Supplement: Supplementary file 5 [file Data_Sheet_2.ZIP › original microscopy Fig2/Fig2A/KO FU 1000 2.jpg]

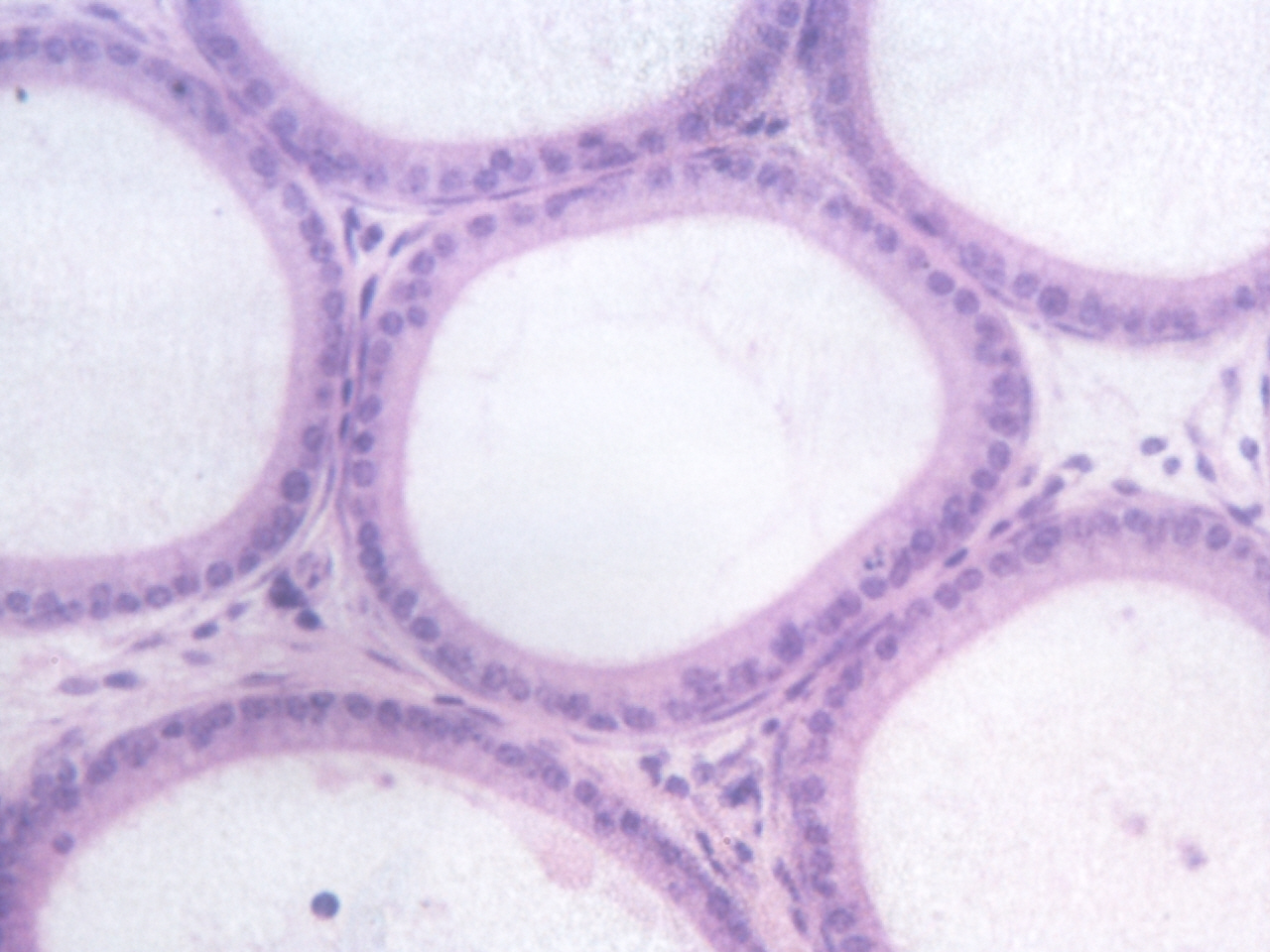

Supplement: Supplementary file 5 [file Data_Sheet_2.ZIP › original microscopy Fig2/Fig2A/KO FU 400 .jpg]

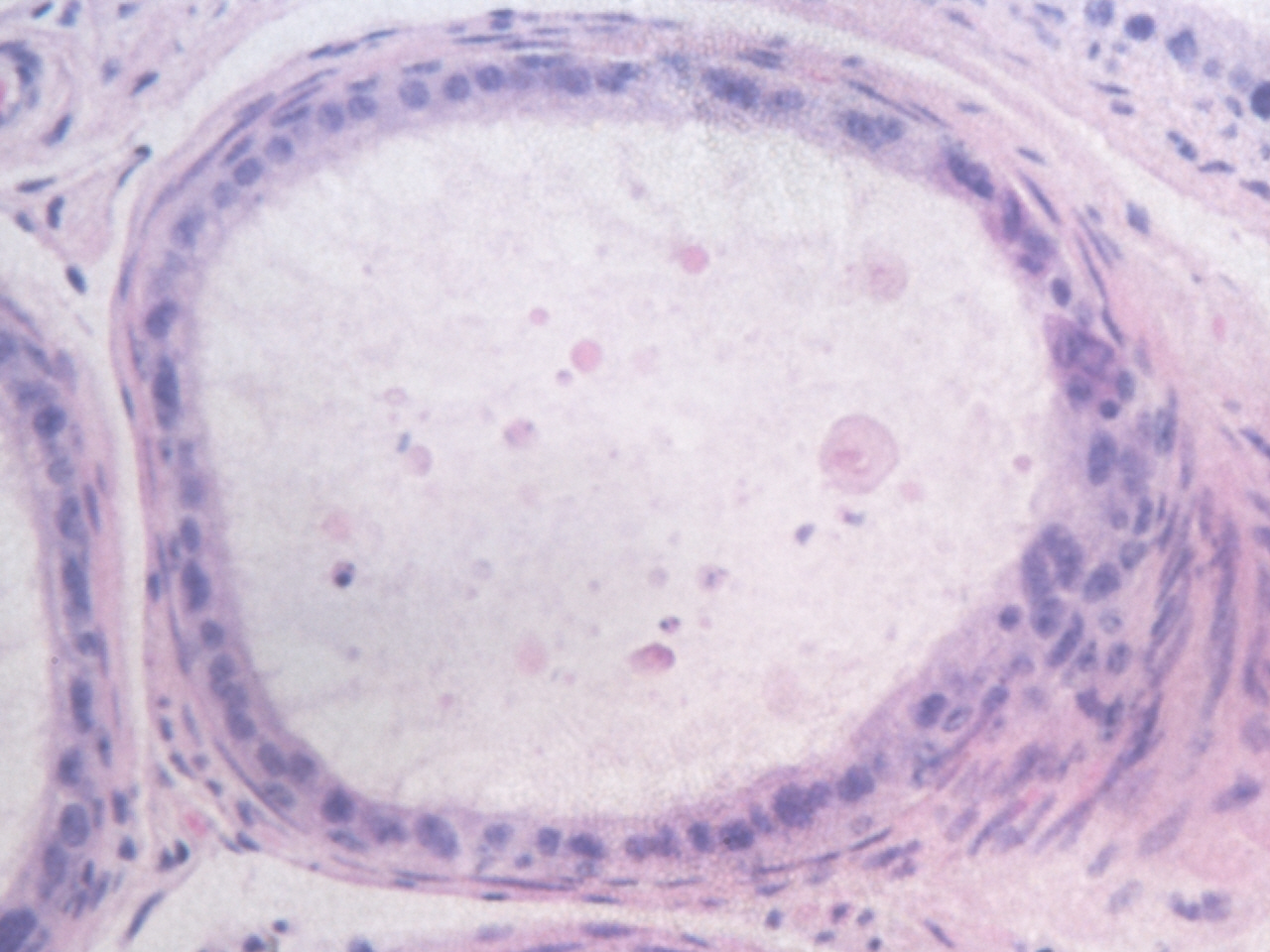

Supplement: Supplementary file 5 [file Data_Sheet_2.ZIP › original microscopy Fig2/Fig2A/KO FU 400 2.jpg]

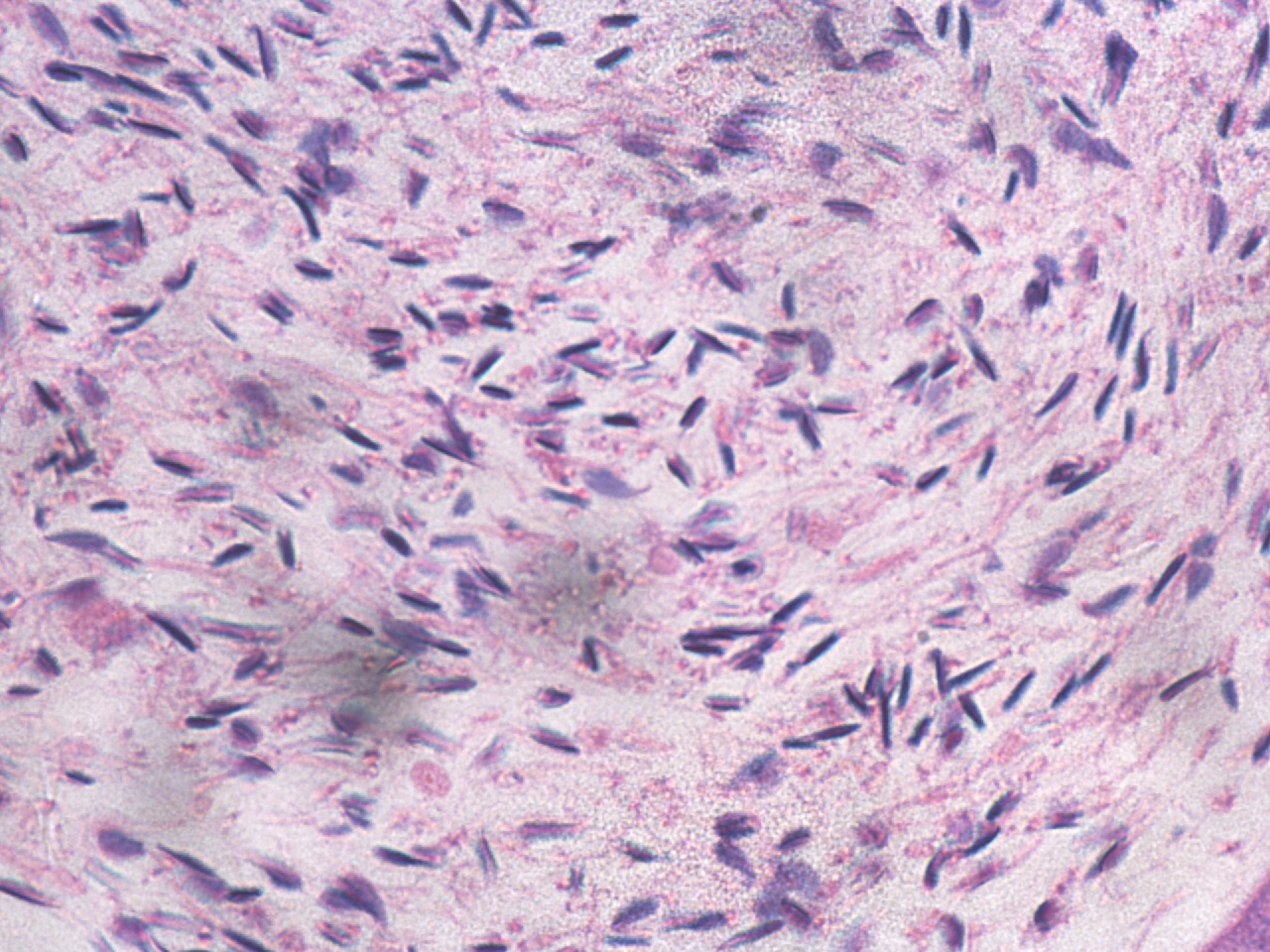

Supplement: Supplementary file 5 [file Data_Sheet_2.ZIP › original microscopy Fig2/Fig2A/WT FU 1000.jpg]

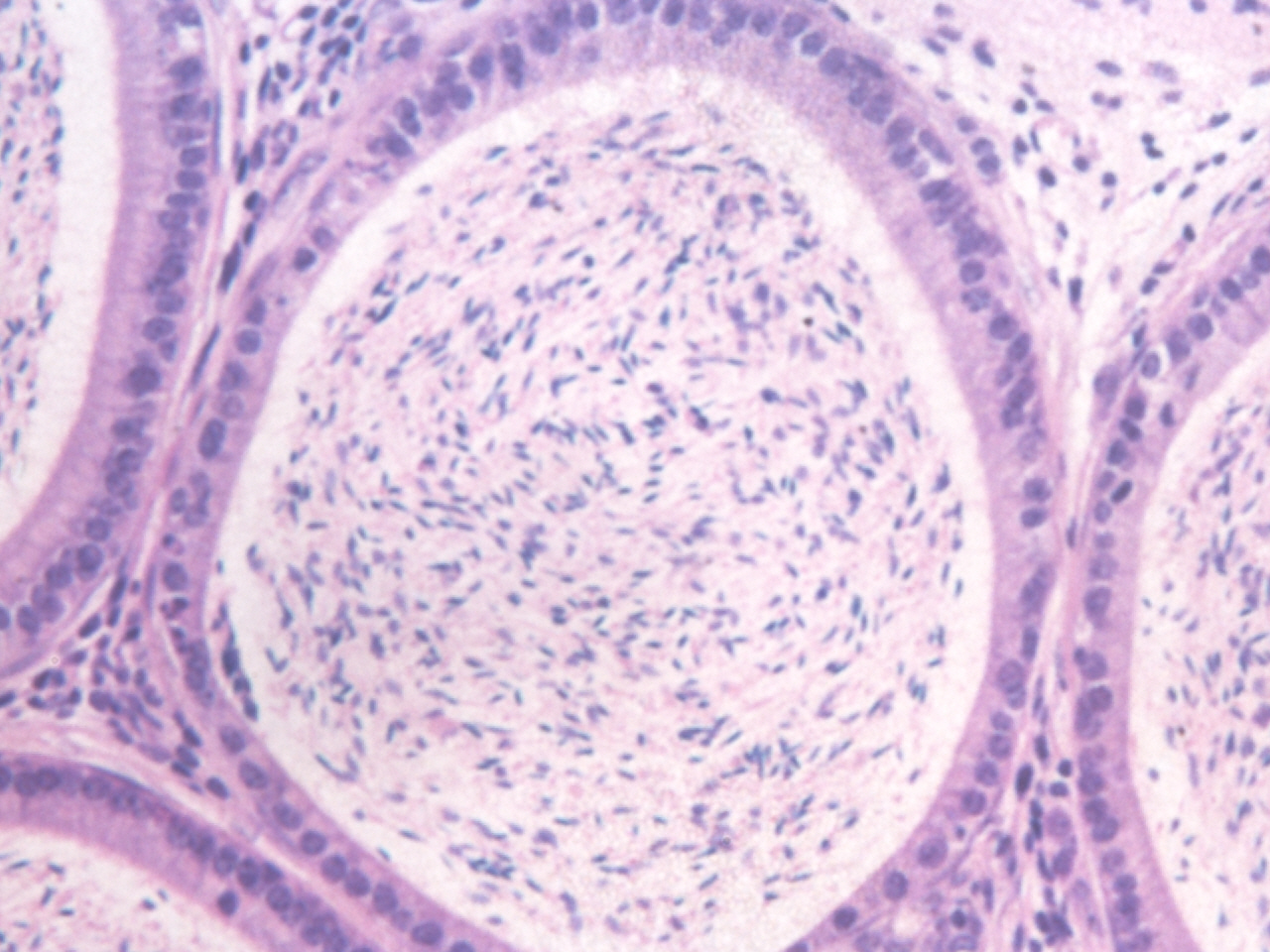

Supplement: Supplementary file 5 [file Data_Sheet_2.ZIP › original microscopy Fig2/Fig2A/WT FU 400.jpg]

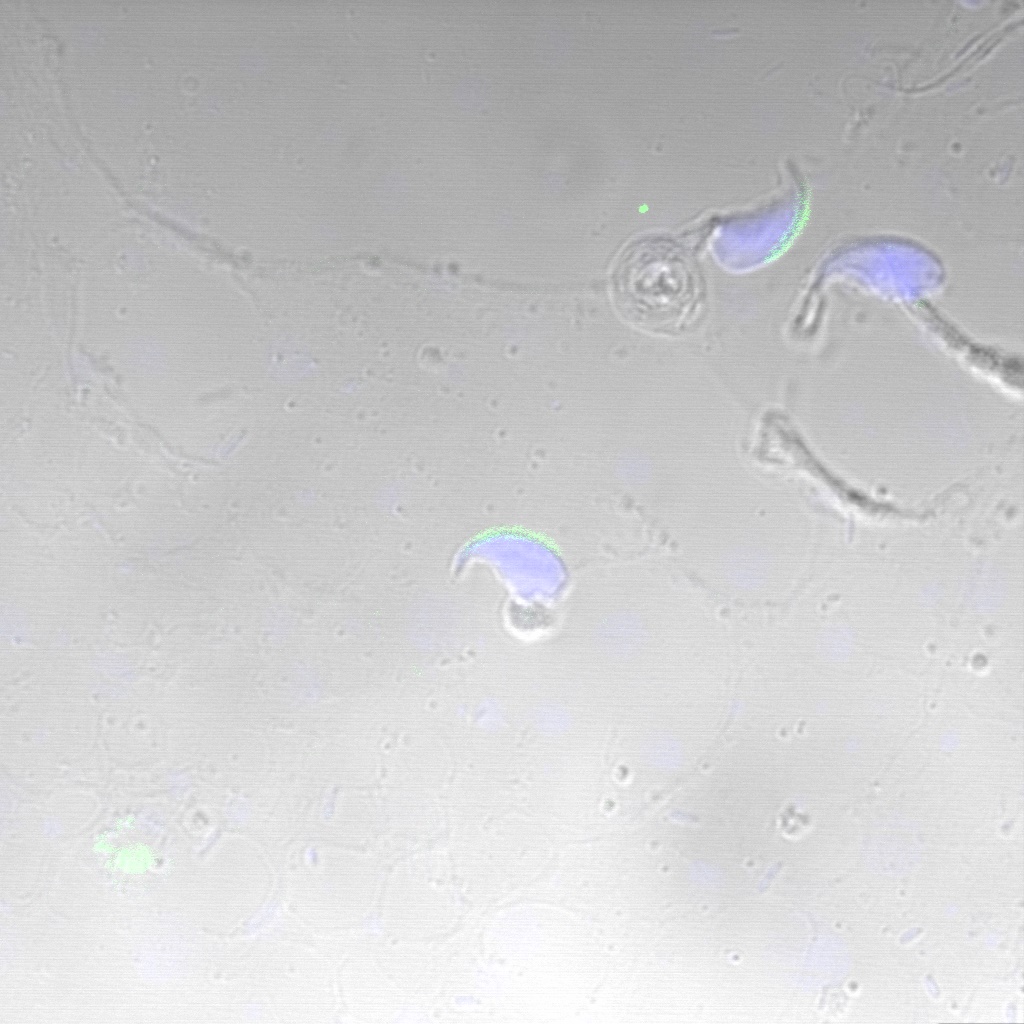

Supplement: Supplementary file 5 [file Data_Sheet_2.ZIP › original microscopy Fig2/Fig2C/ko1.jpg]

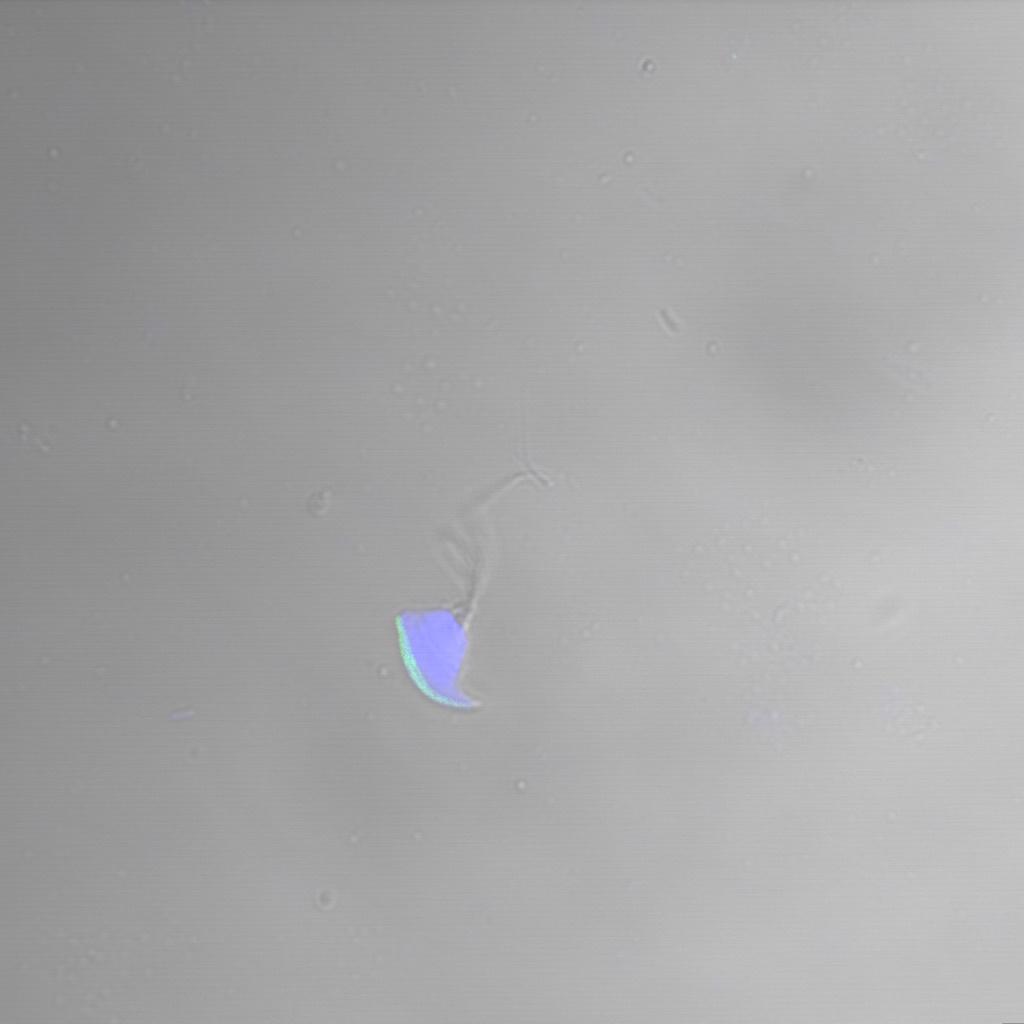

Supplement: Supplementary file 5 [file Data_Sheet_2.ZIP › original microscopy Fig2/Fig2C/ko2.jpg]

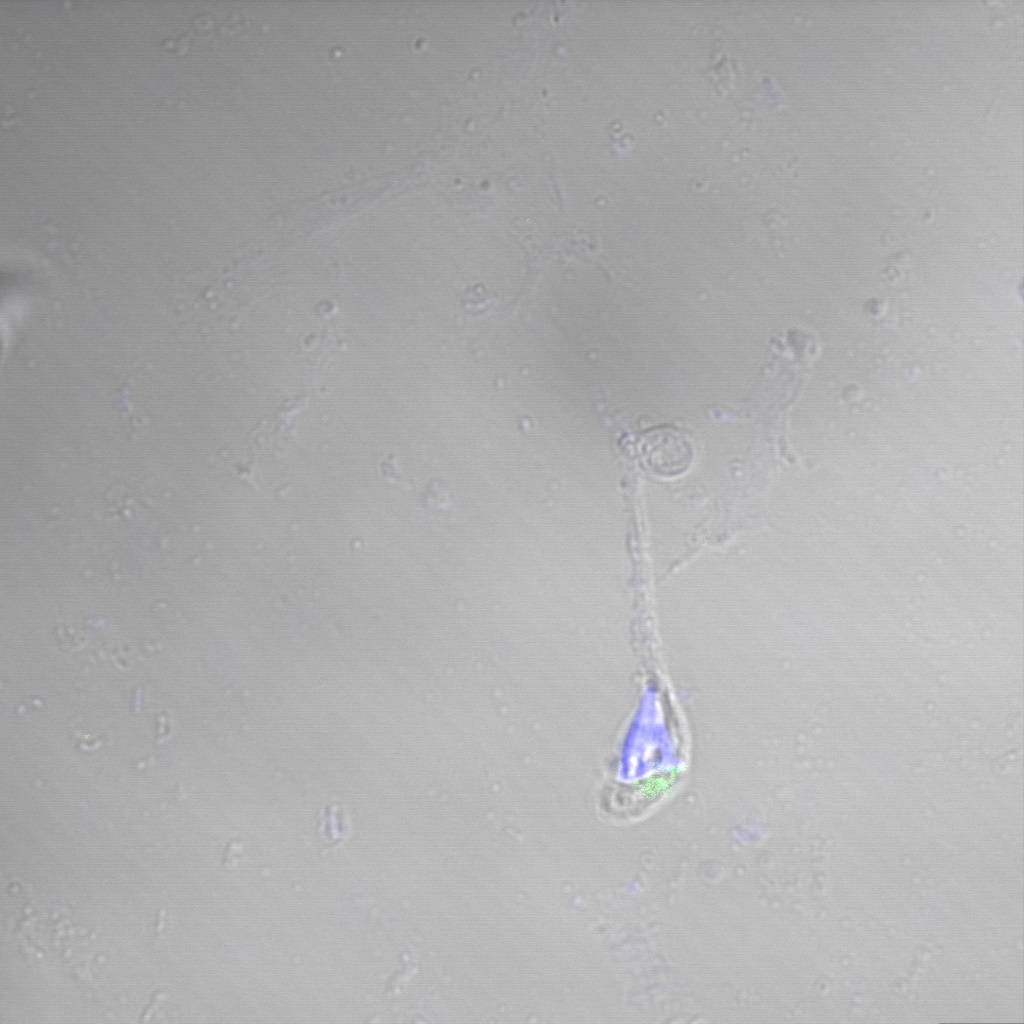

Supplement: Supplementary file 5 [file Data_Sheet_2.ZIP › original microscopy Fig2/Fig2C/ko3.jpg]

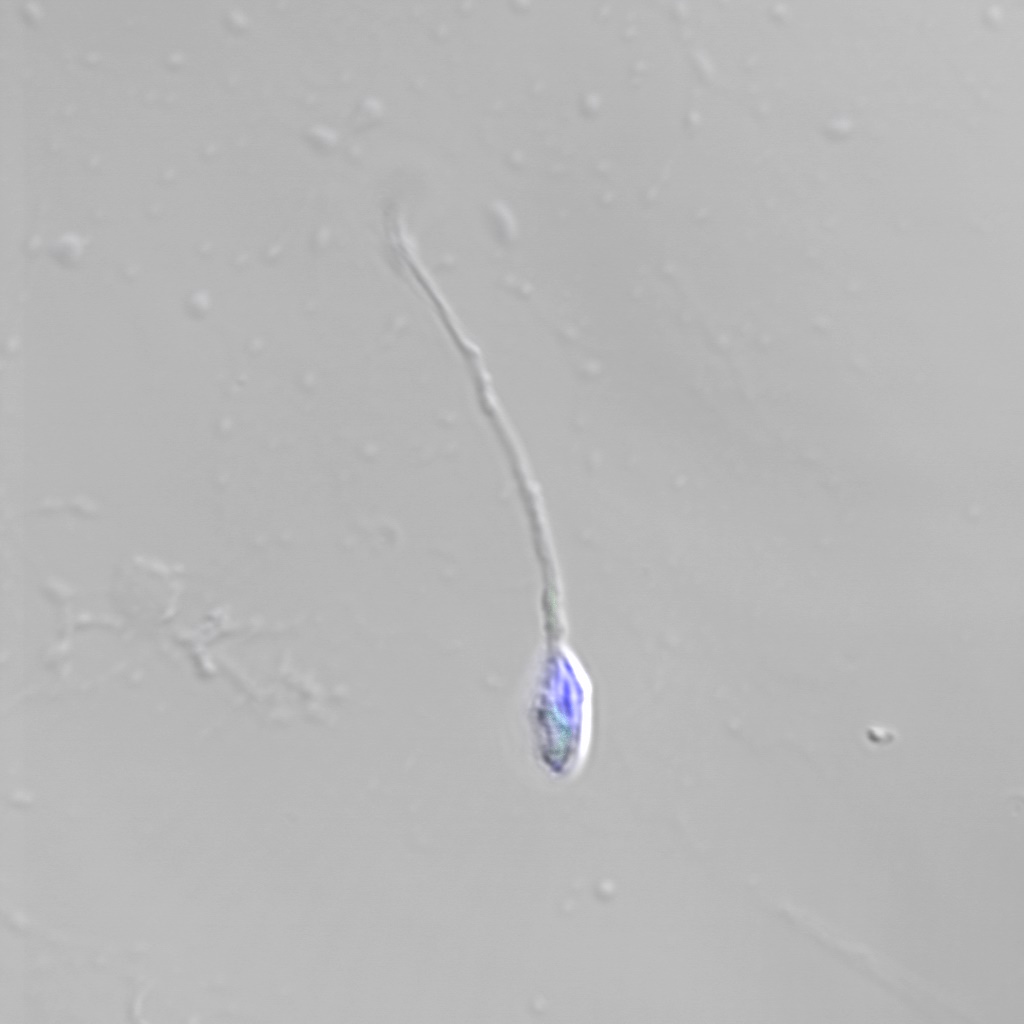

Supplement: Supplementary file 5 [file Data_Sheet_2.ZIP › original microscopy Fig2/Fig2C/ko4.jpg]

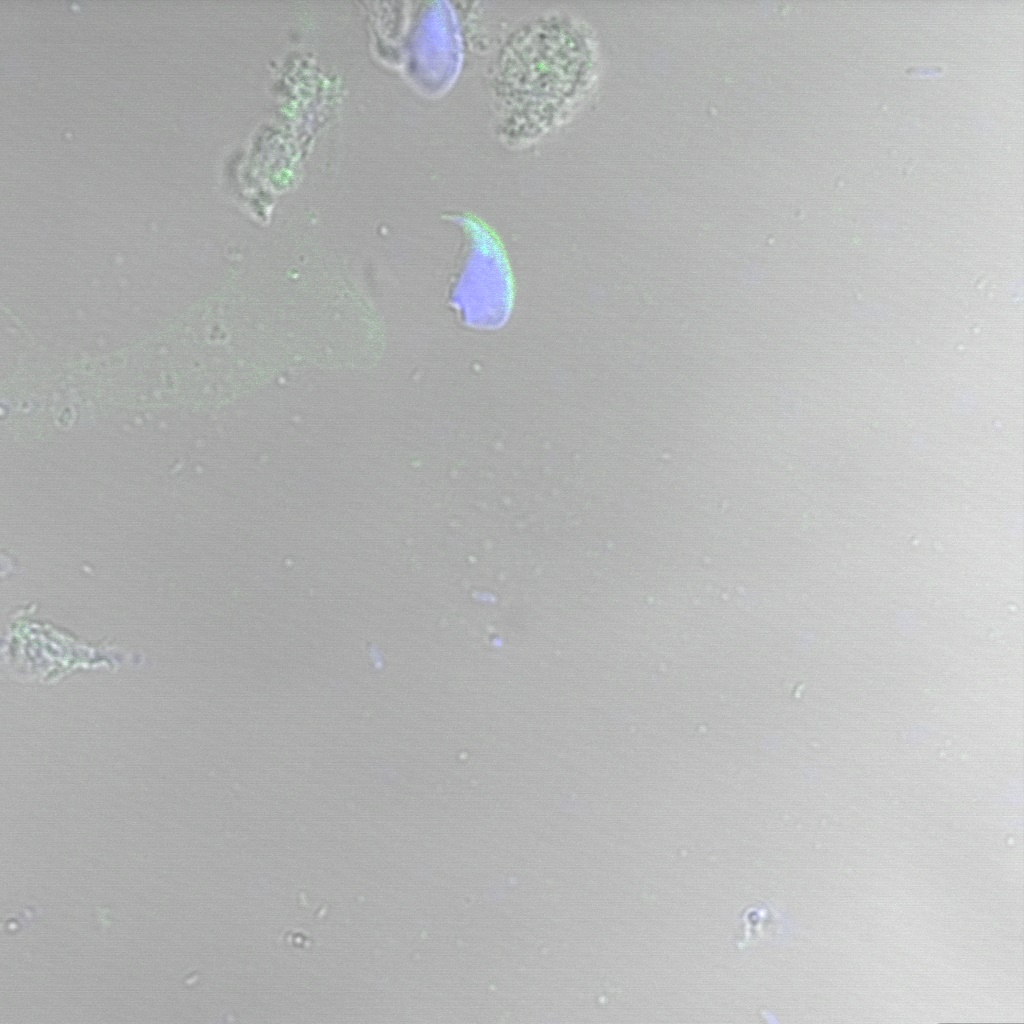

Supplement: Supplementary file 5 [file Data_Sheet_2.ZIP › original microscopy Fig2/Fig2C/ko5.jpg]

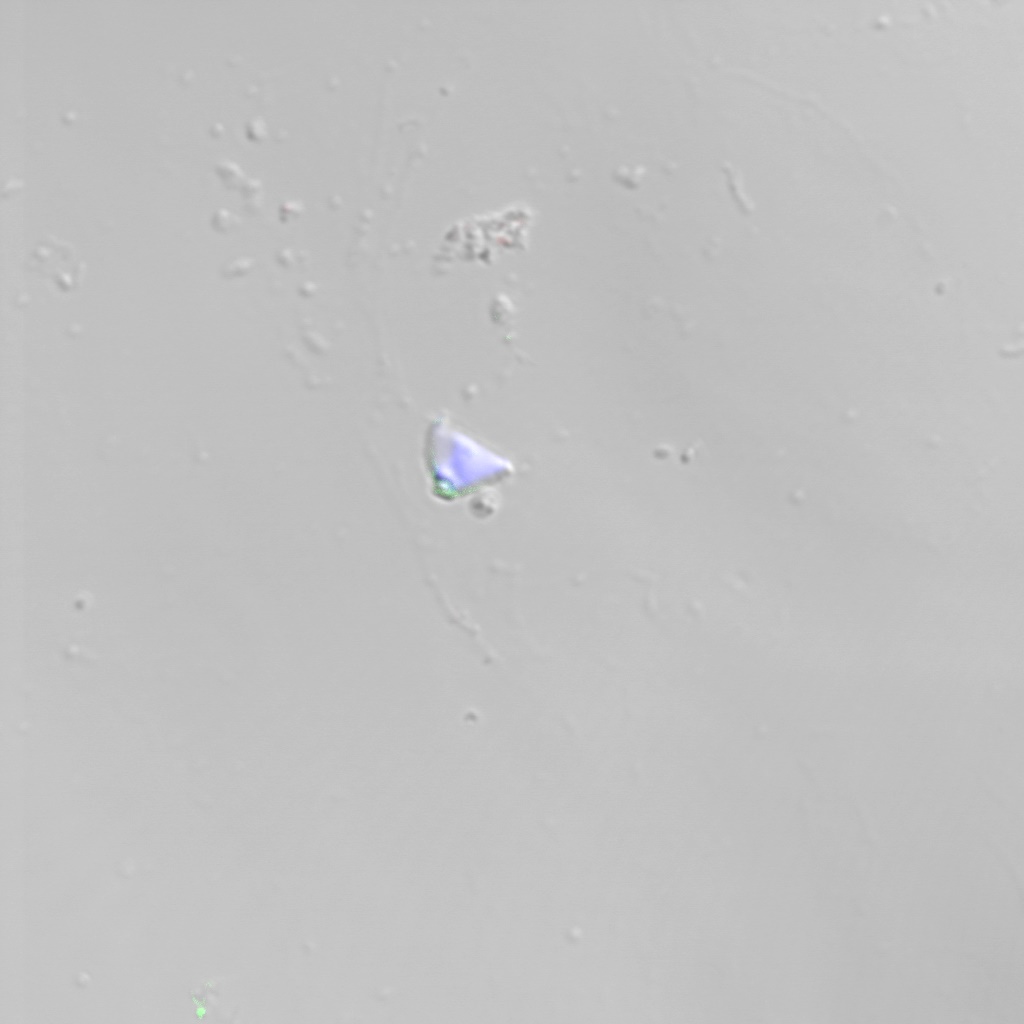

Supplement: Supplementary file 5 [file Data_Sheet_2.ZIP › original microscopy Fig2/Fig2C/ko6.jpg]

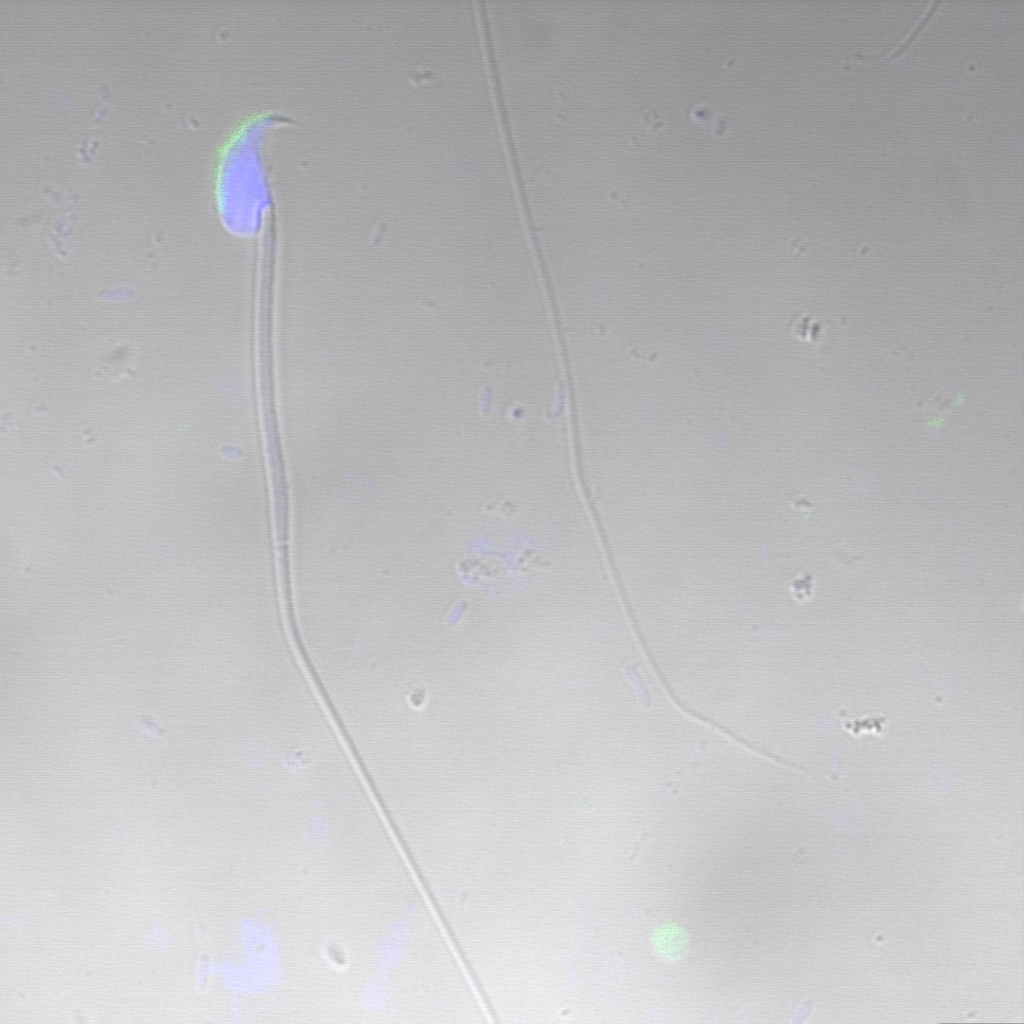

Supplement: Supplementary file 5 [file Data_Sheet_2.ZIP › original microscopy Fig2/Fig2C/wt.jpg]

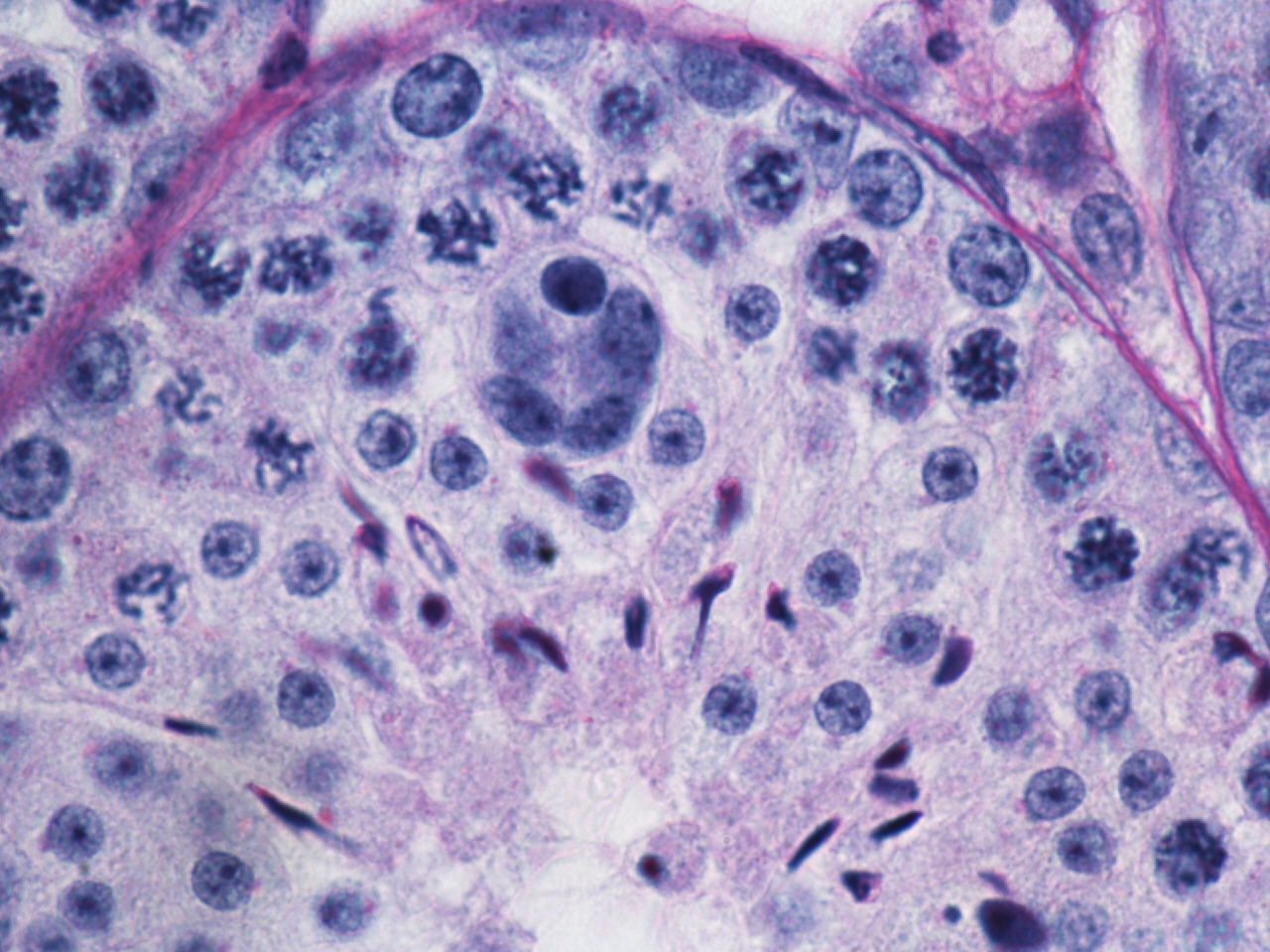

Supplement: Supplementary file 6 [file Data_Sheet_3.ZIP › Fig3A/cfap53 ko/1-3 .jpg]

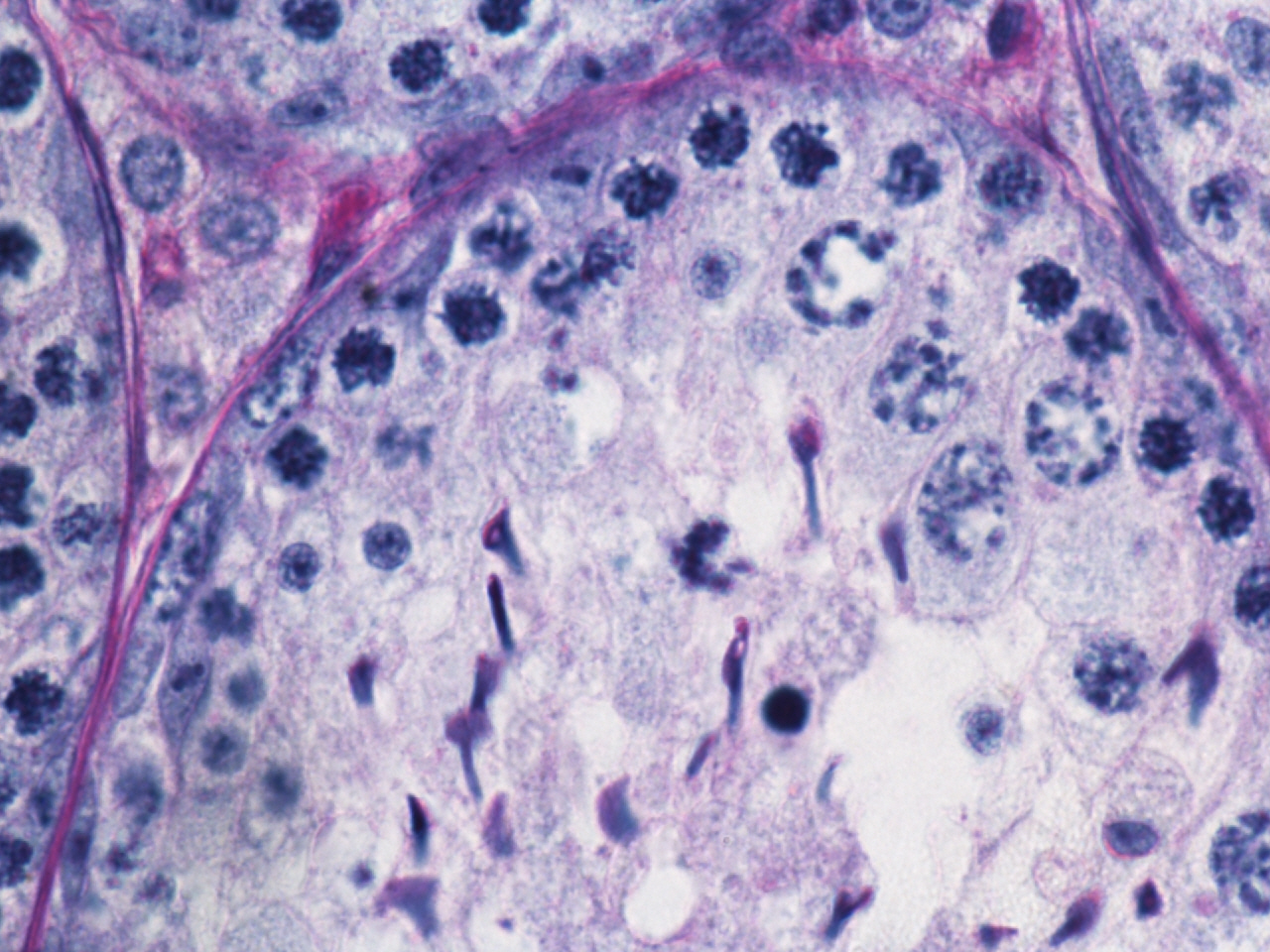

Supplement: Supplementary file 6 [file Data_Sheet_3.ZIP › Fig3A/cfap53 ko/10-11.jpg]

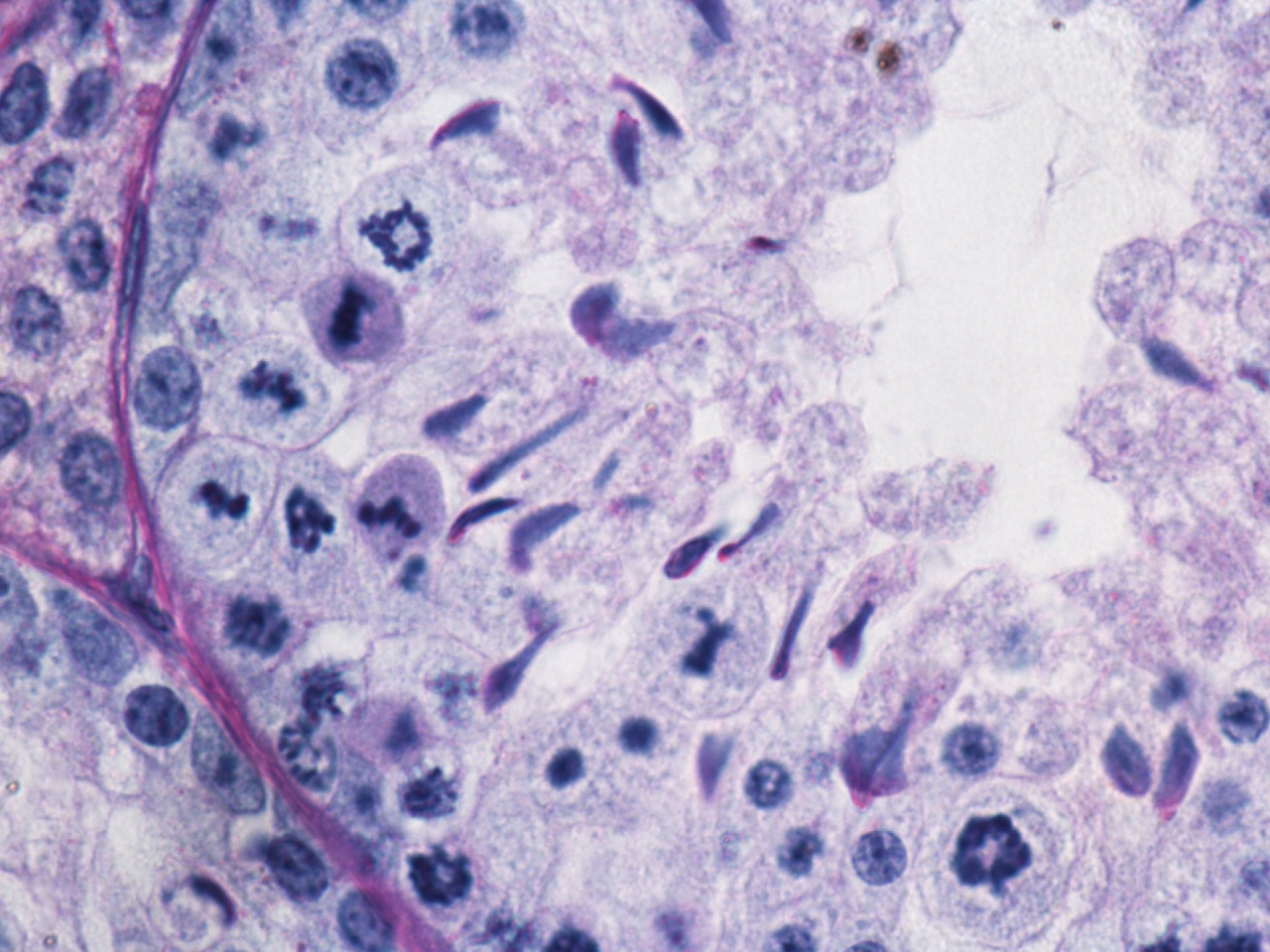

Supplement: Supplementary file 6 [file Data_Sheet_3.ZIP › Fig3A/cfap53 ko/12.jpg]

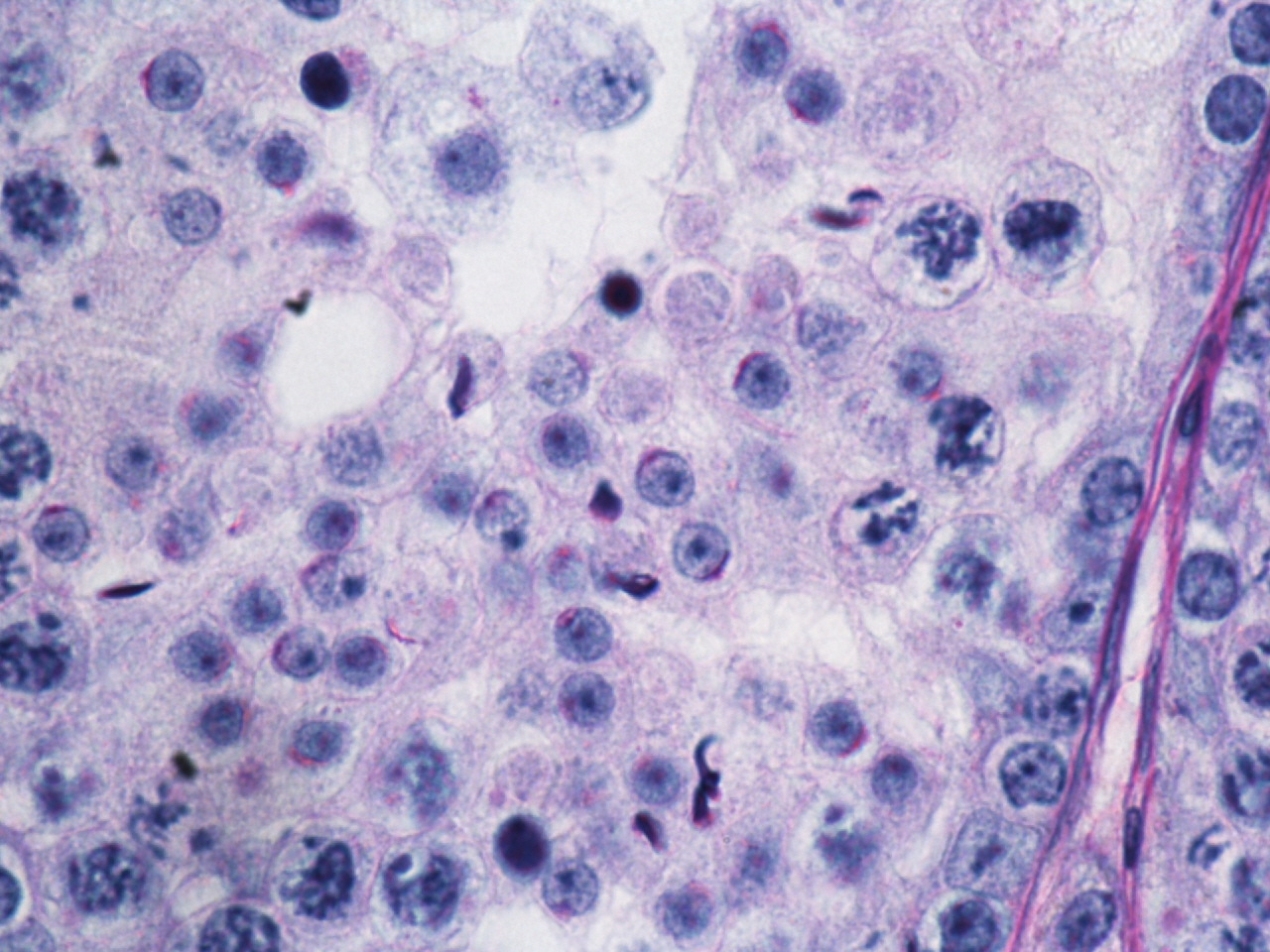

Supplement: Supplementary file 6 [file Data_Sheet_3.ZIP › Fig3A/cfap53 ko/4.jpg]

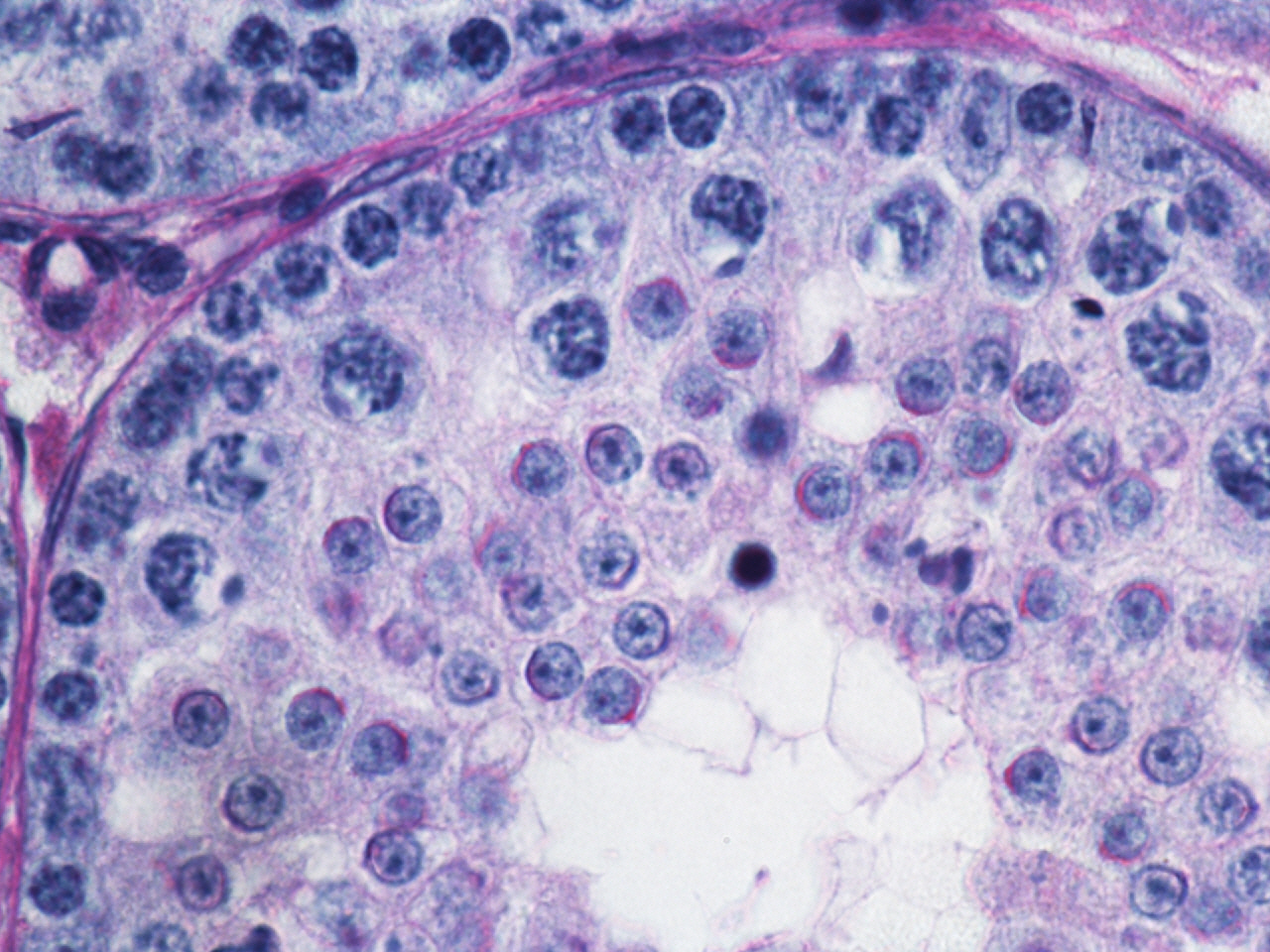

Supplement: Supplementary file 6 [file Data_Sheet_3.ZIP › Fig3A/cfap53 ko/5-8.jpg]

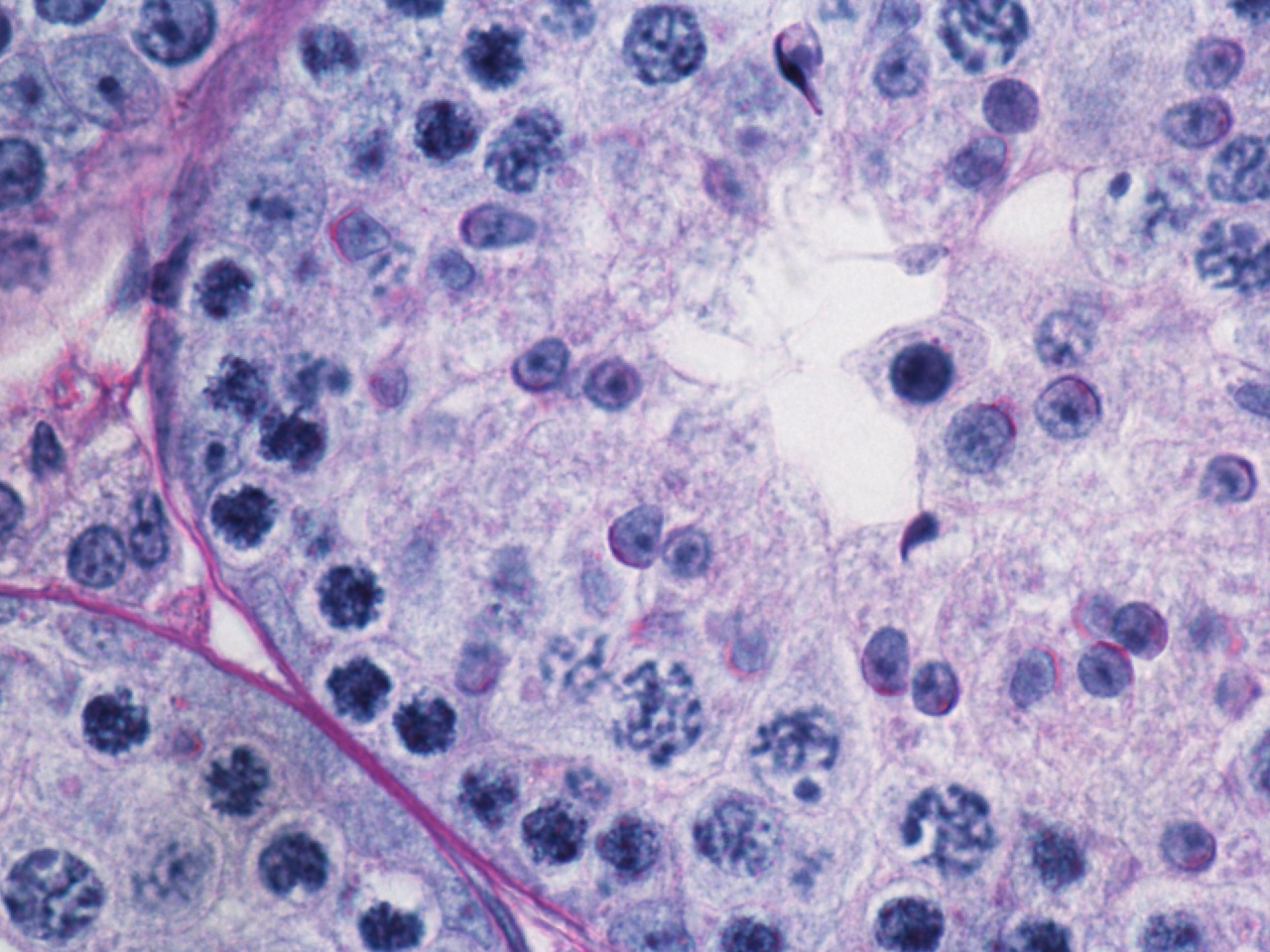

Supplement: Supplementary file 6 [file Data_Sheet_3.ZIP › Fig3A/cfap53 ko/9.jpg]

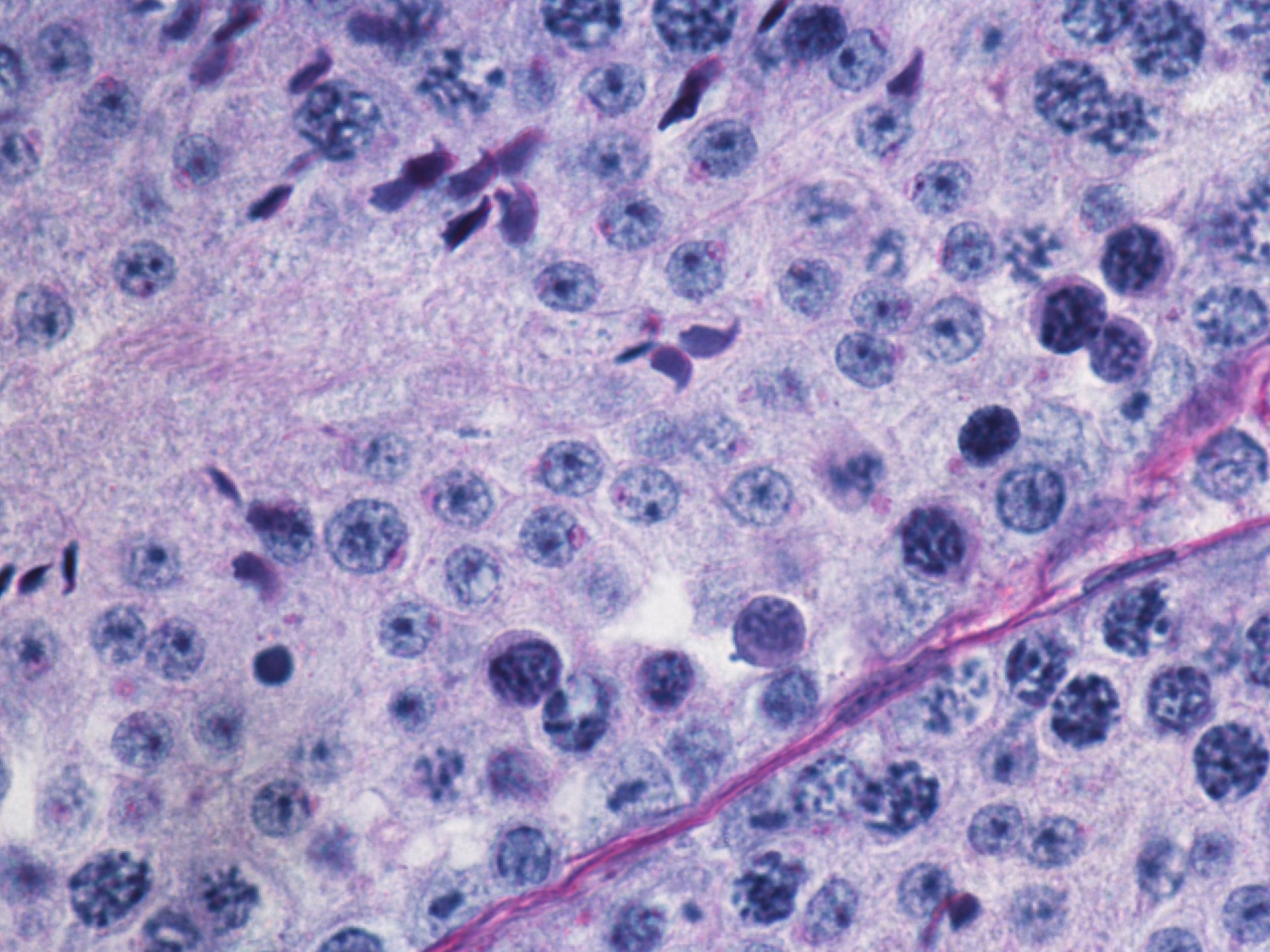

Supplement: Supplementary file 6 [file Data_Sheet_3.ZIP › Fig3A/cfap53 wt/1-3.jpg]

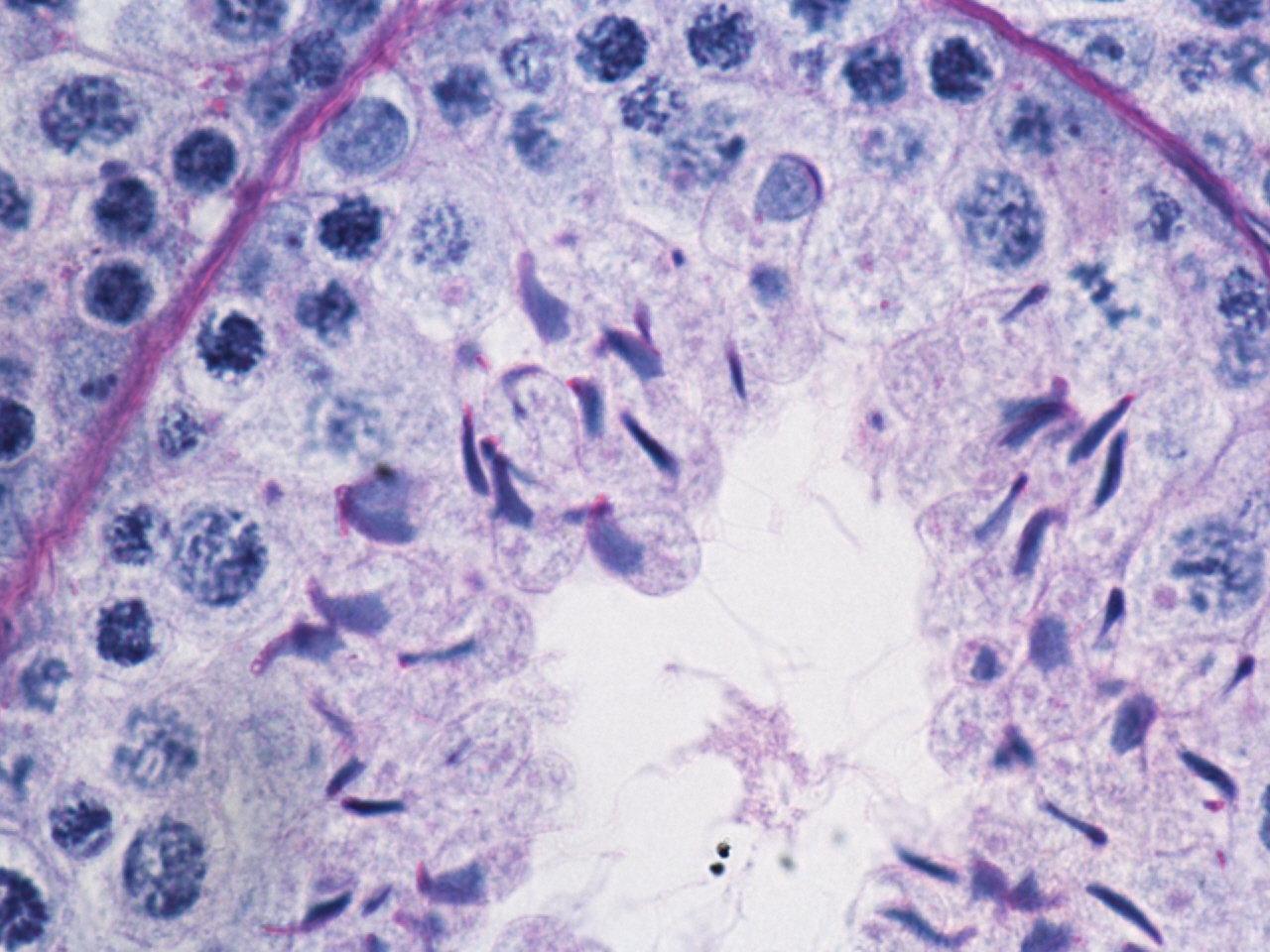

Supplement: Supplementary file 6 [file Data_Sheet_3.ZIP › Fig3A/cfap53 wt/10-11.jpg]

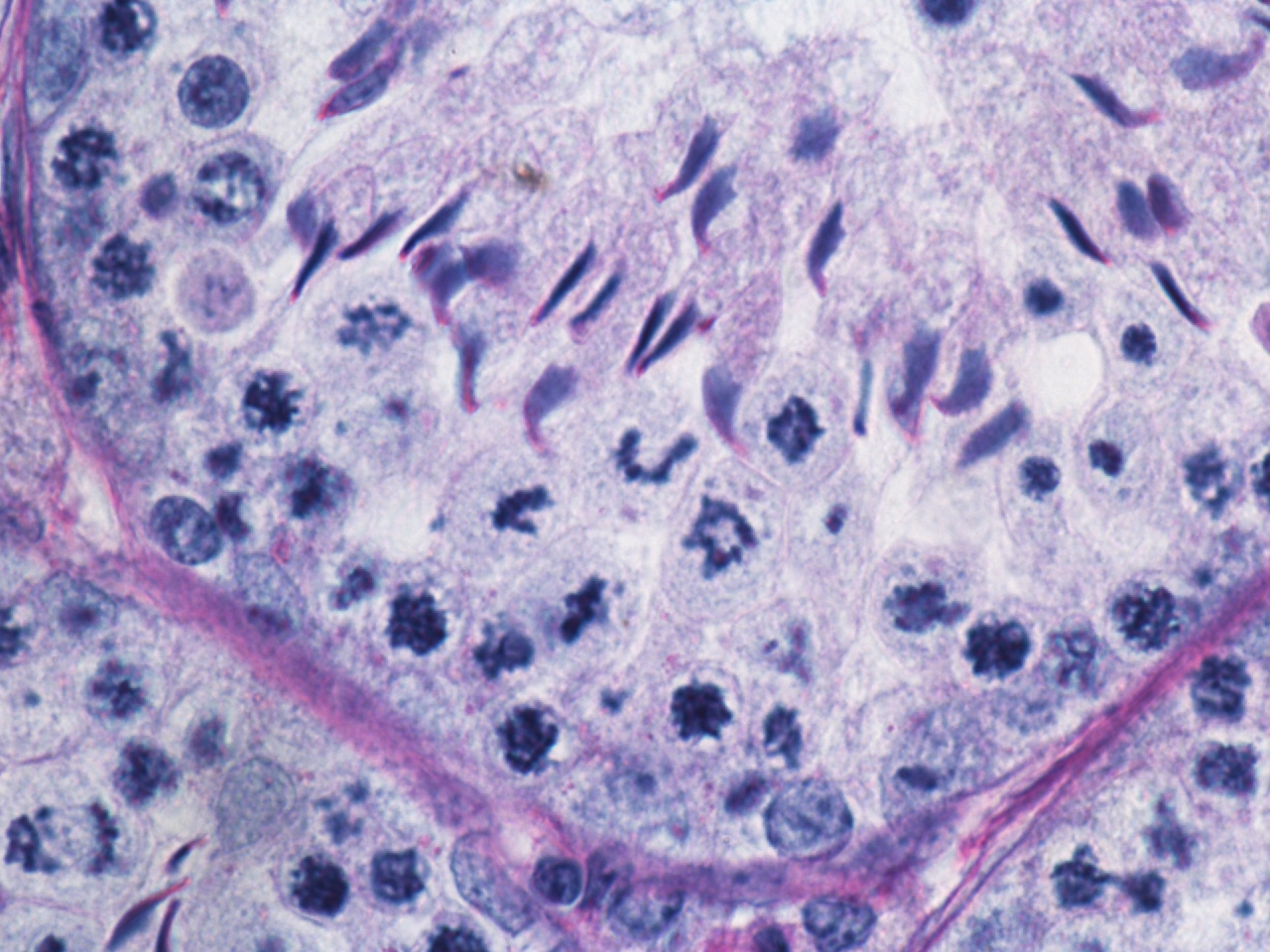

Supplement: Supplementary file 6 [file Data_Sheet_3.ZIP › Fig3A/cfap53 wt/12.jpg]

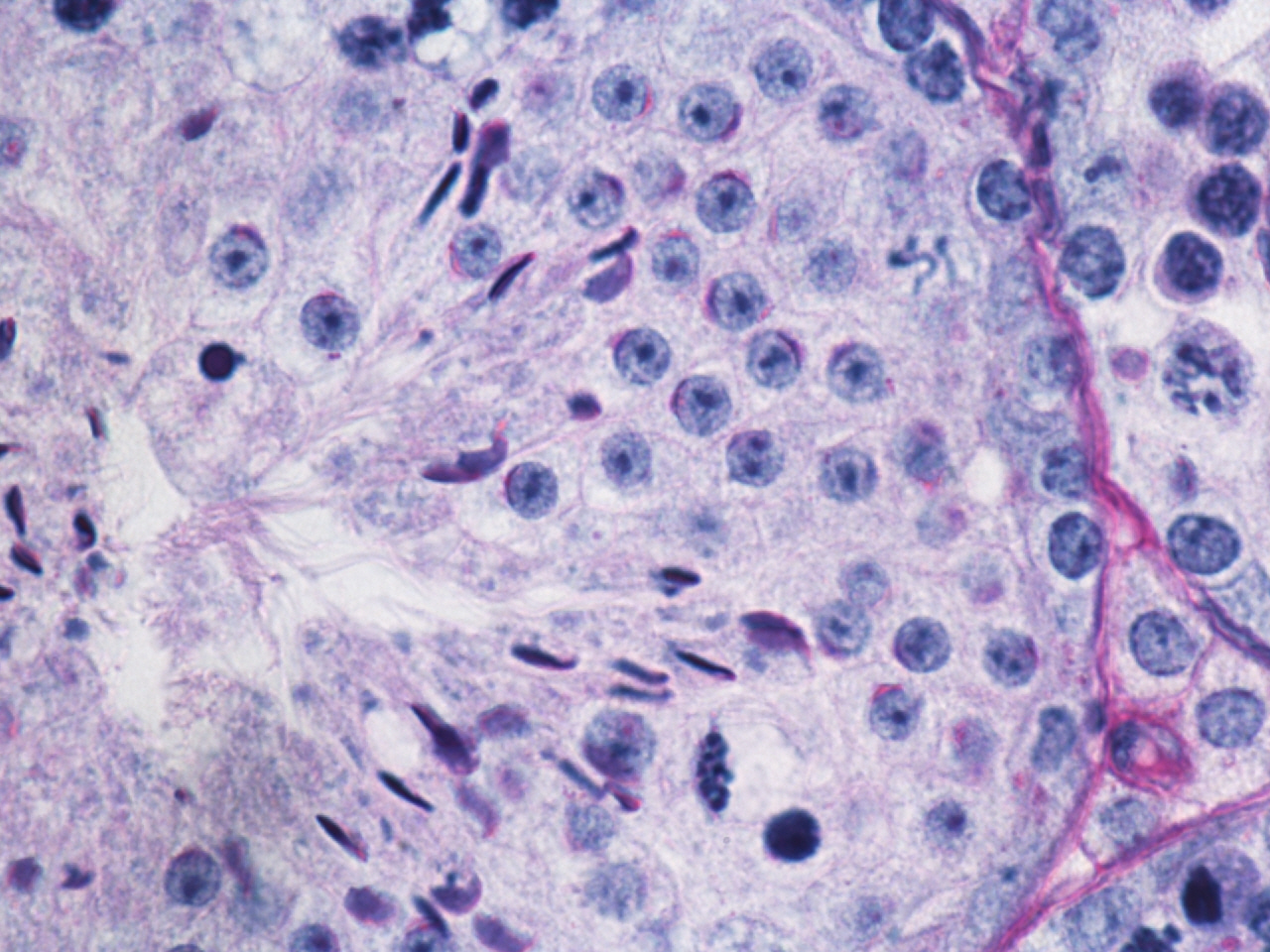

Supplement: Supplementary file 6 [file Data_Sheet_3.ZIP › Fig3A/cfap53 wt/4.jpg]

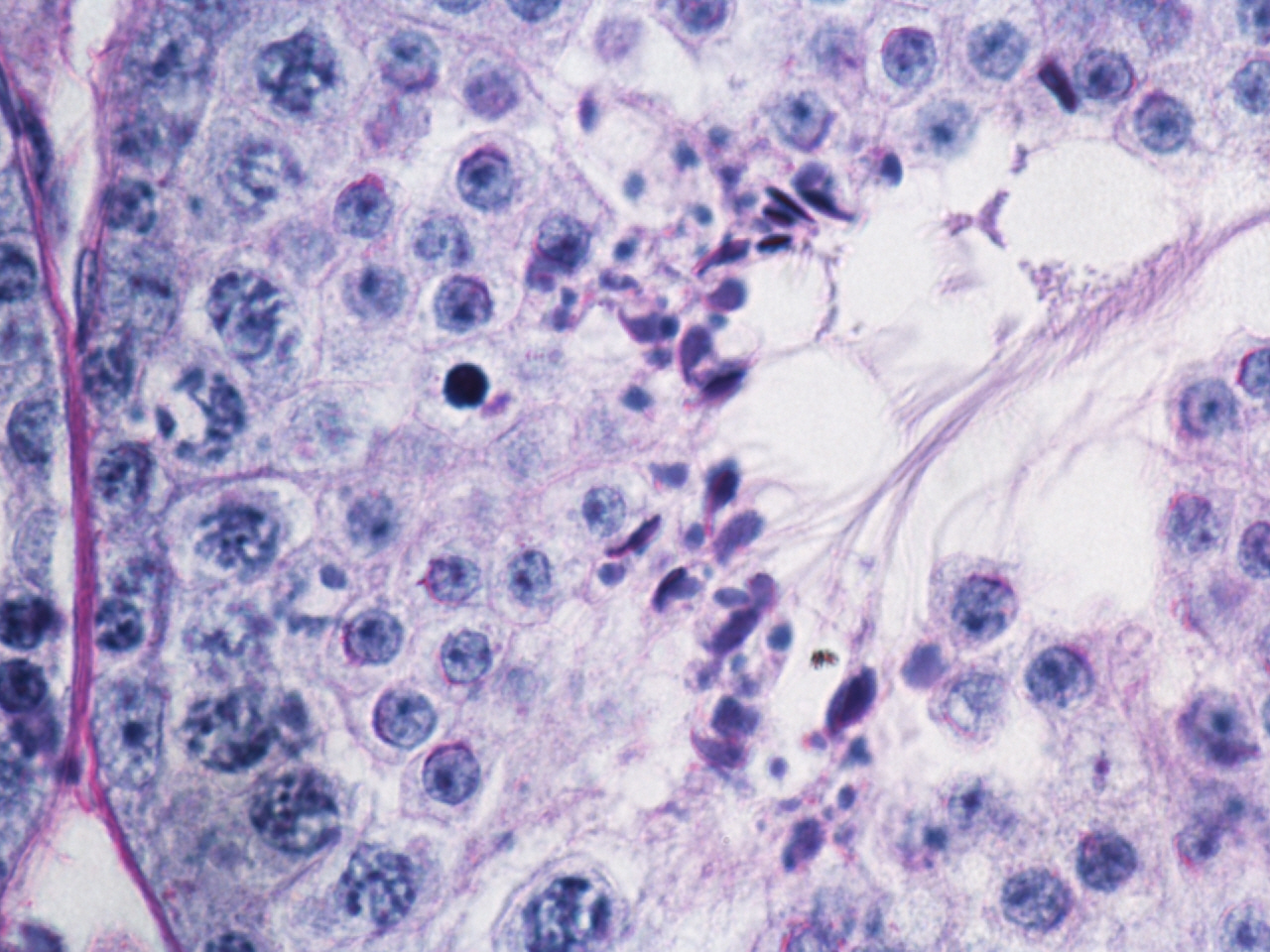

Supplement: Supplementary file 6 [file Data_Sheet_3.ZIP › Fig3A/cfap53 wt/5-8.jpg]

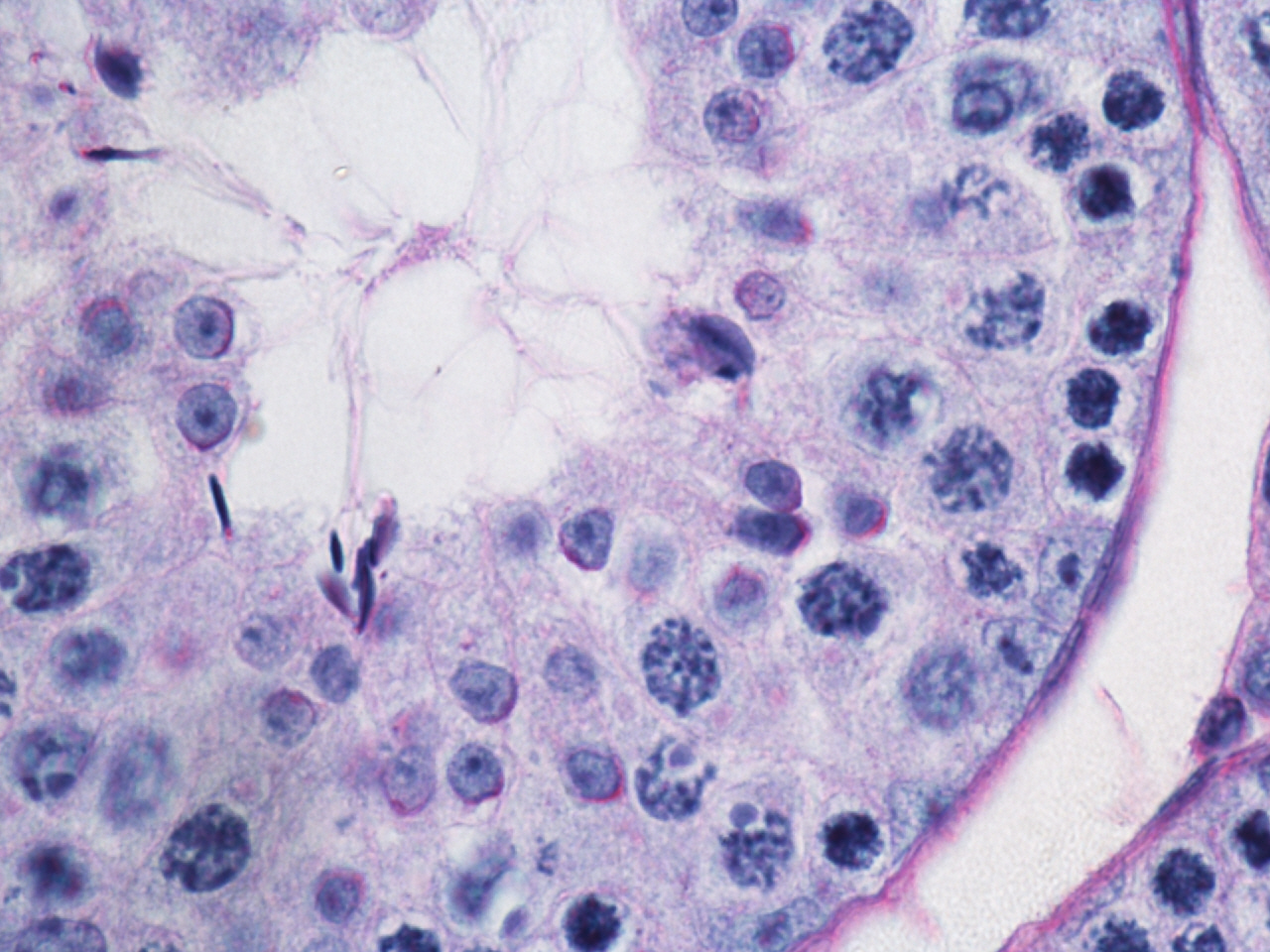

Supplement: Supplementary file 6 [file Data_Sheet_3.ZIP › Fig3A/cfap53 wt/9.jpg]

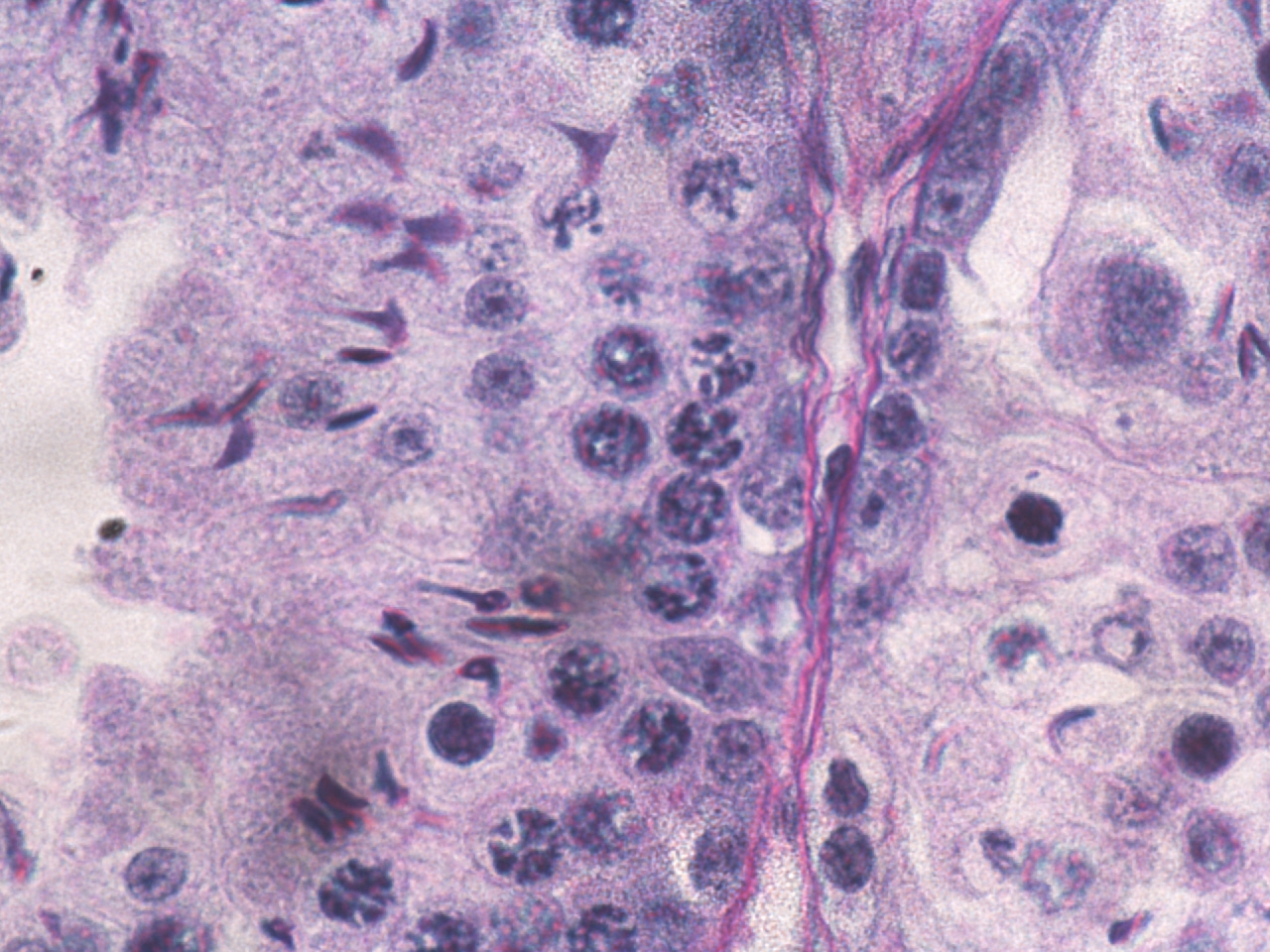

Supplement: Supplementary file 7 [file Data_Sheet_4.zip › Fig3B/ko/1.jpg]

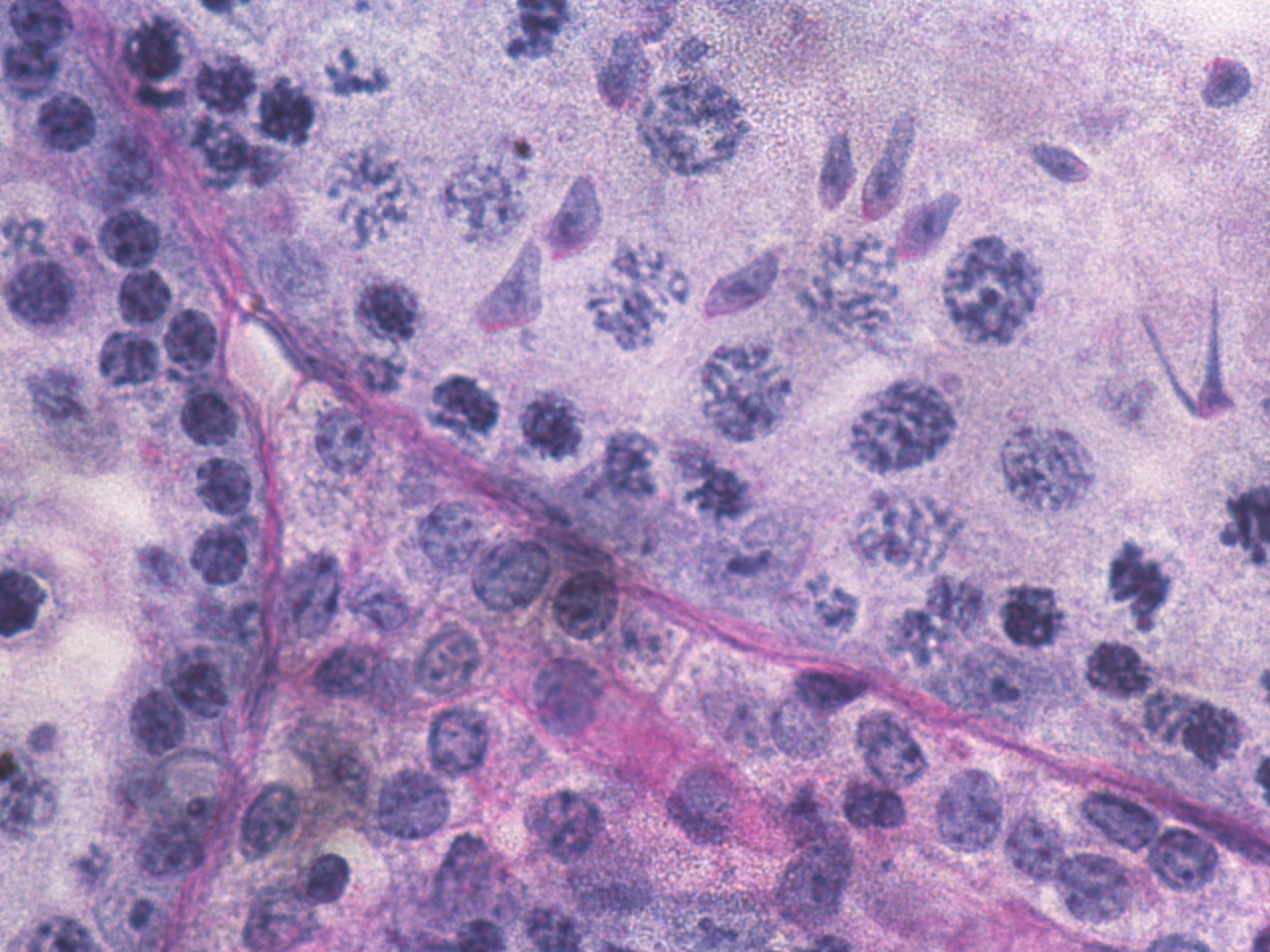

Supplement: Supplementary file 7 [file Data_Sheet_4.zip › Fig3B/ko/10.jpg]

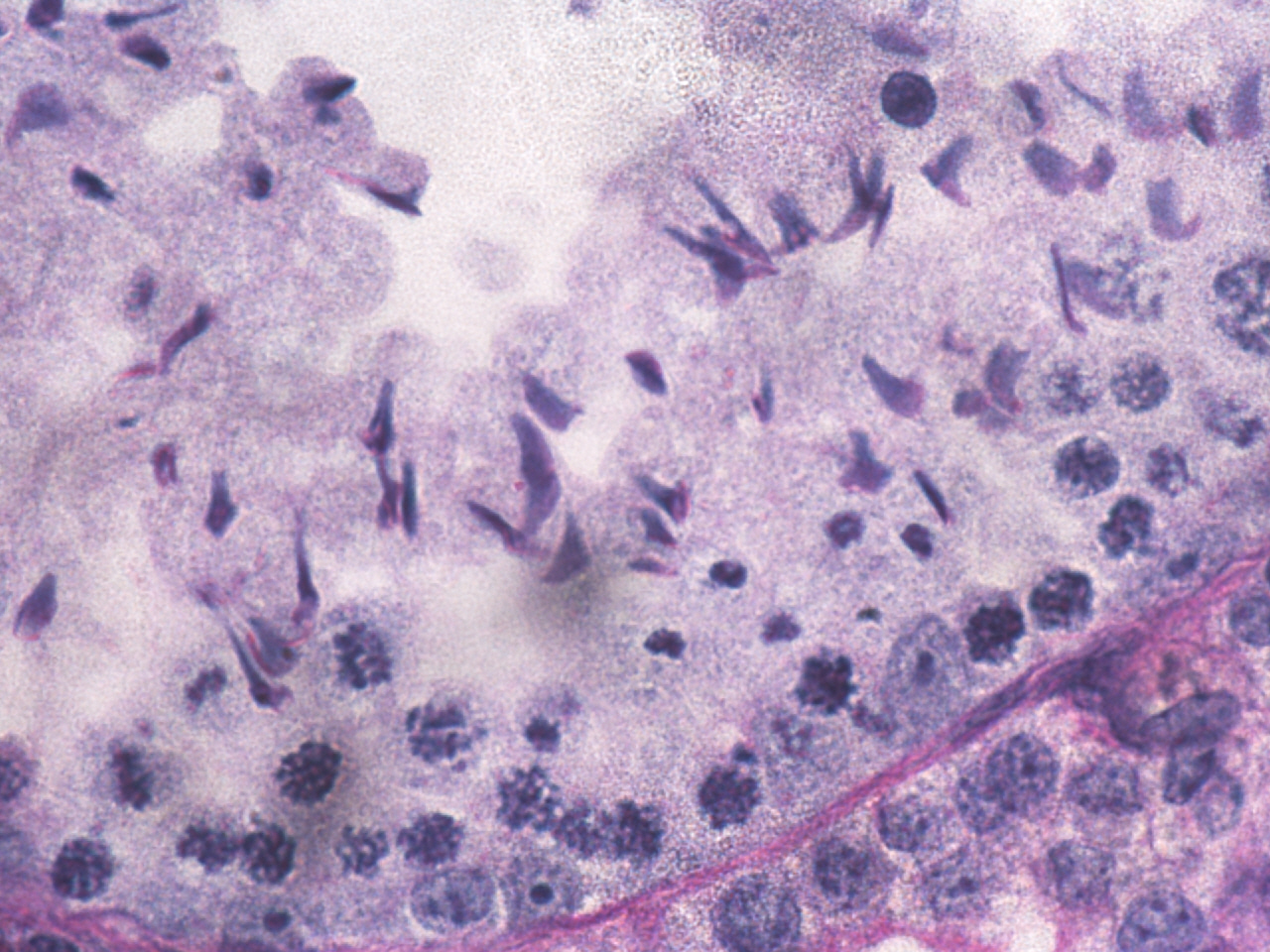

Supplement: Supplementary file 7 [file Data_Sheet_4.zip › Fig3B/ko/11.jpg]

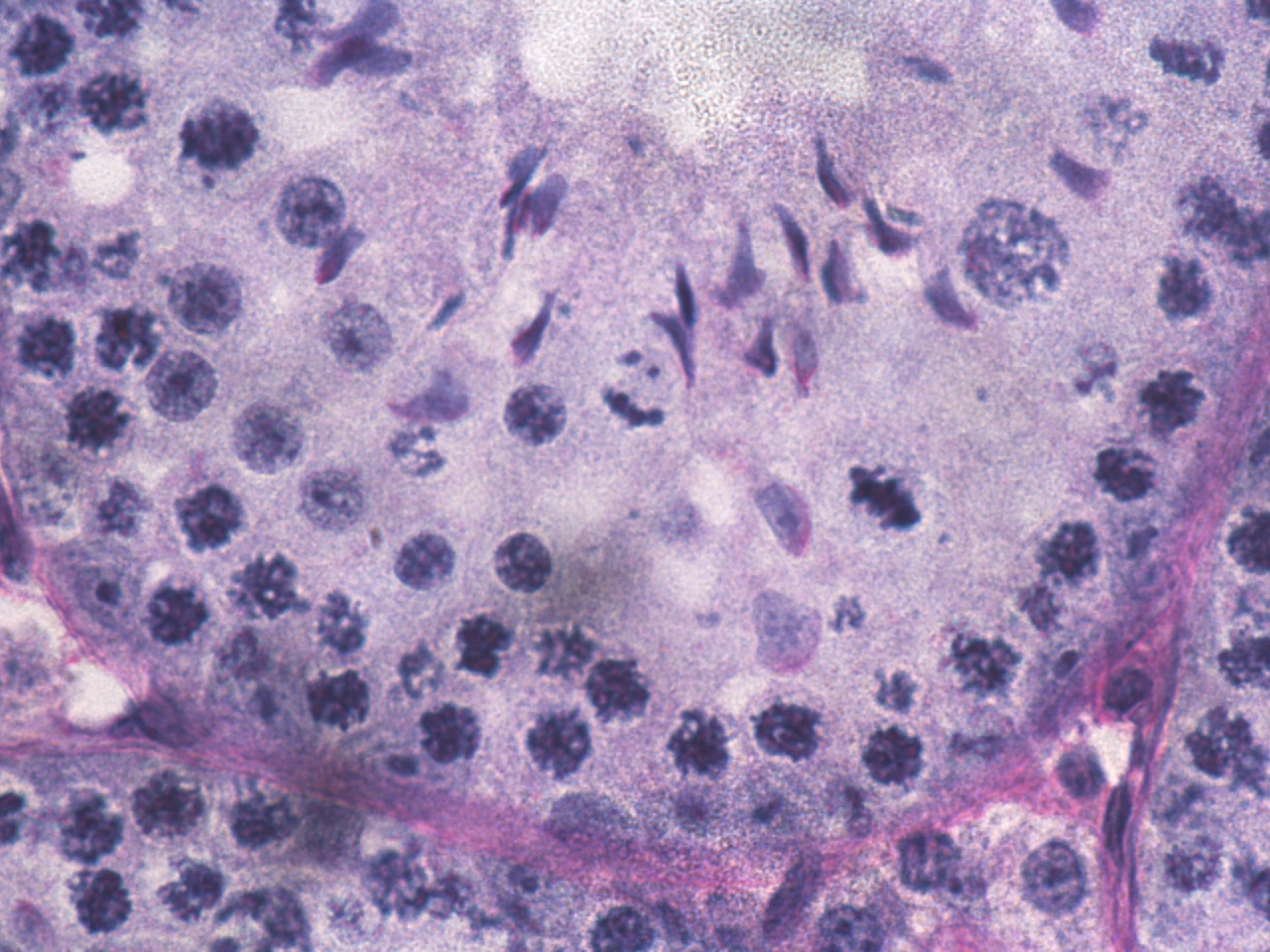

Supplement: Supplementary file 7 [file Data_Sheet_4.zip › Fig3B/ko/12.jpg]

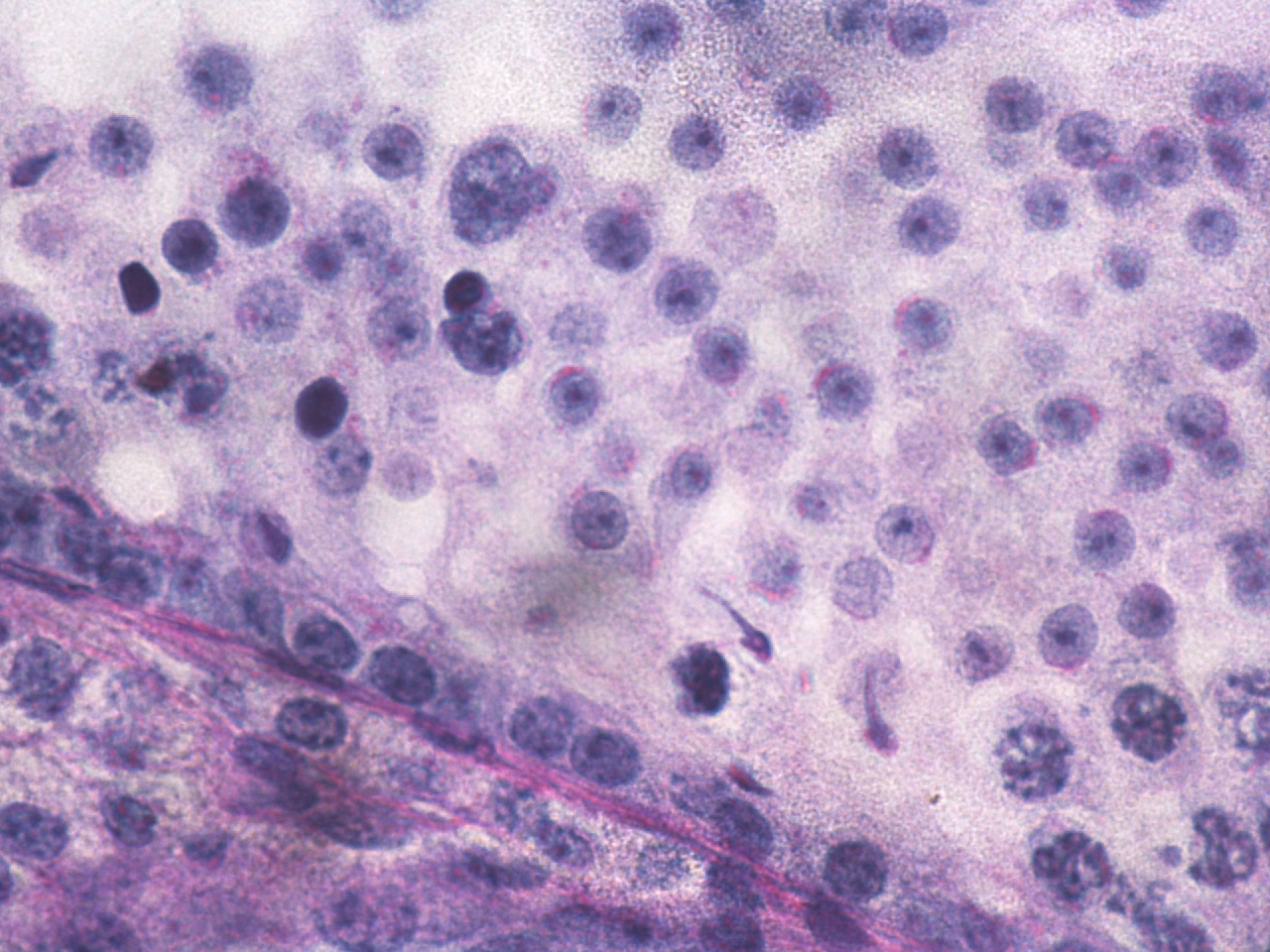

Supplement: Supplementary file 7 [file Data_Sheet_4.zip › Fig3B/ko/13.jpg]

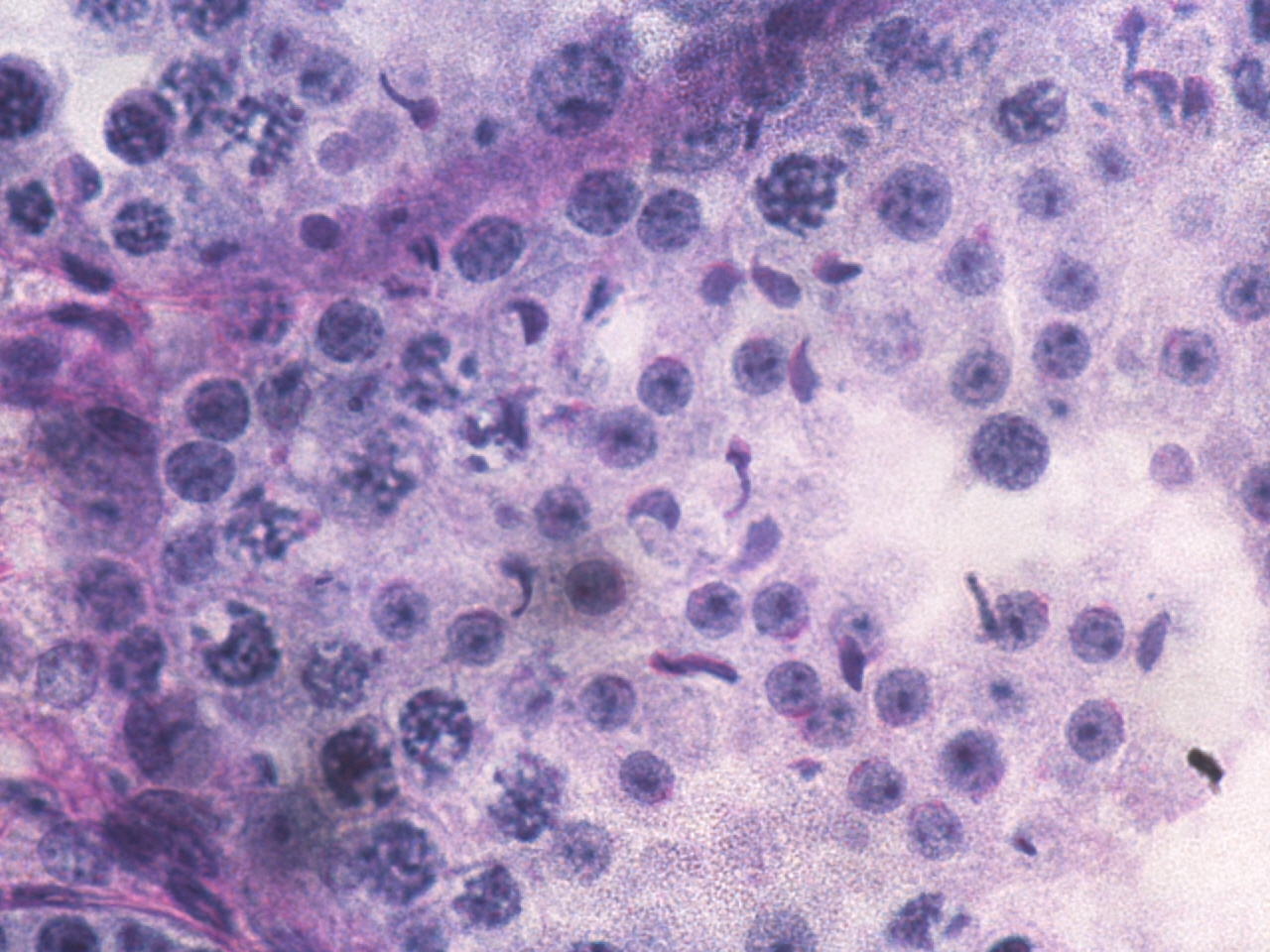

Supplement: Supplementary file 7 [file Data_Sheet_4.zip › Fig3B/ko/14.jpg]

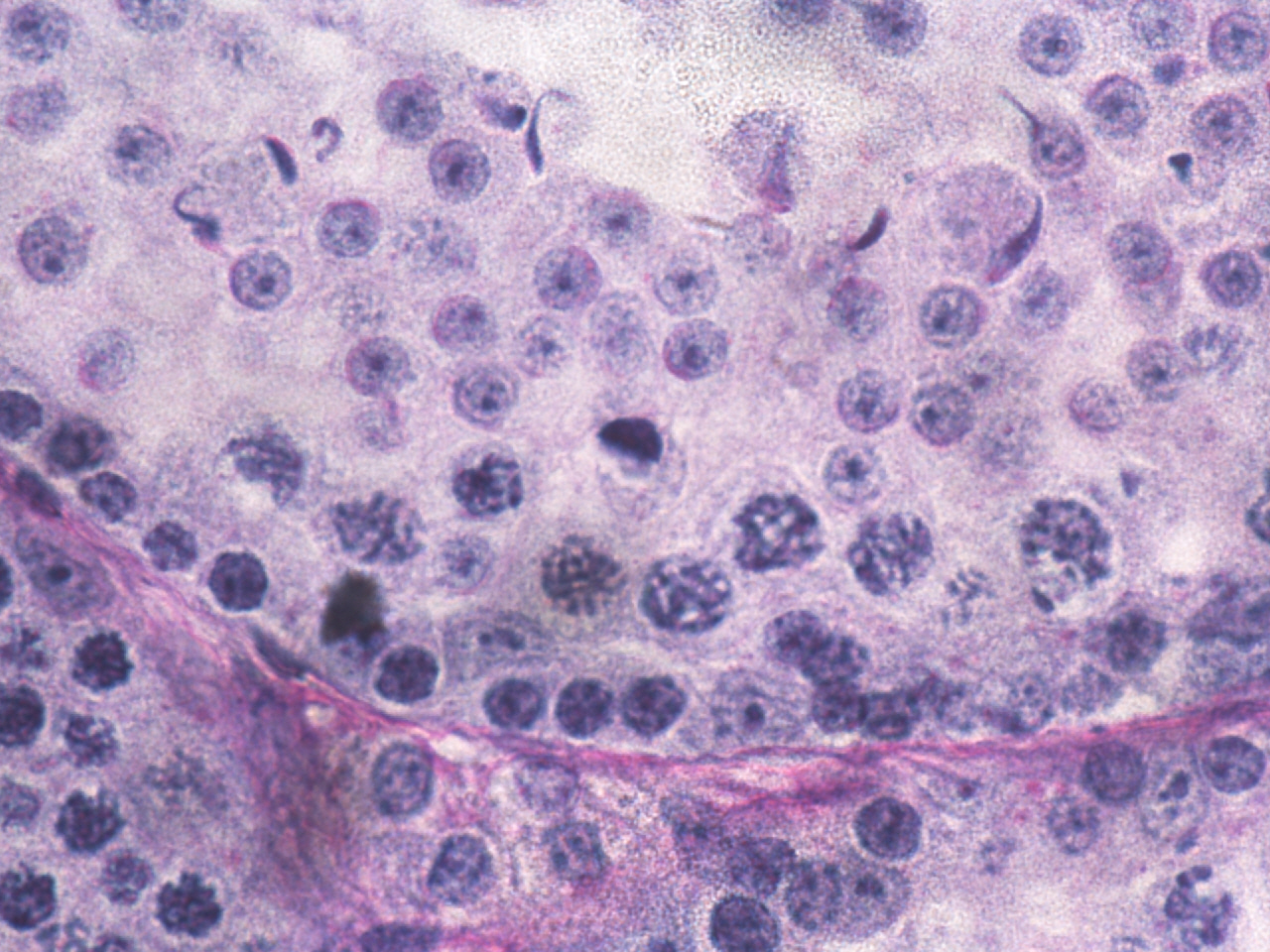

Supplement: Supplementary file 7 [file Data_Sheet_4.zip › Fig3B/ko/15.jpg]

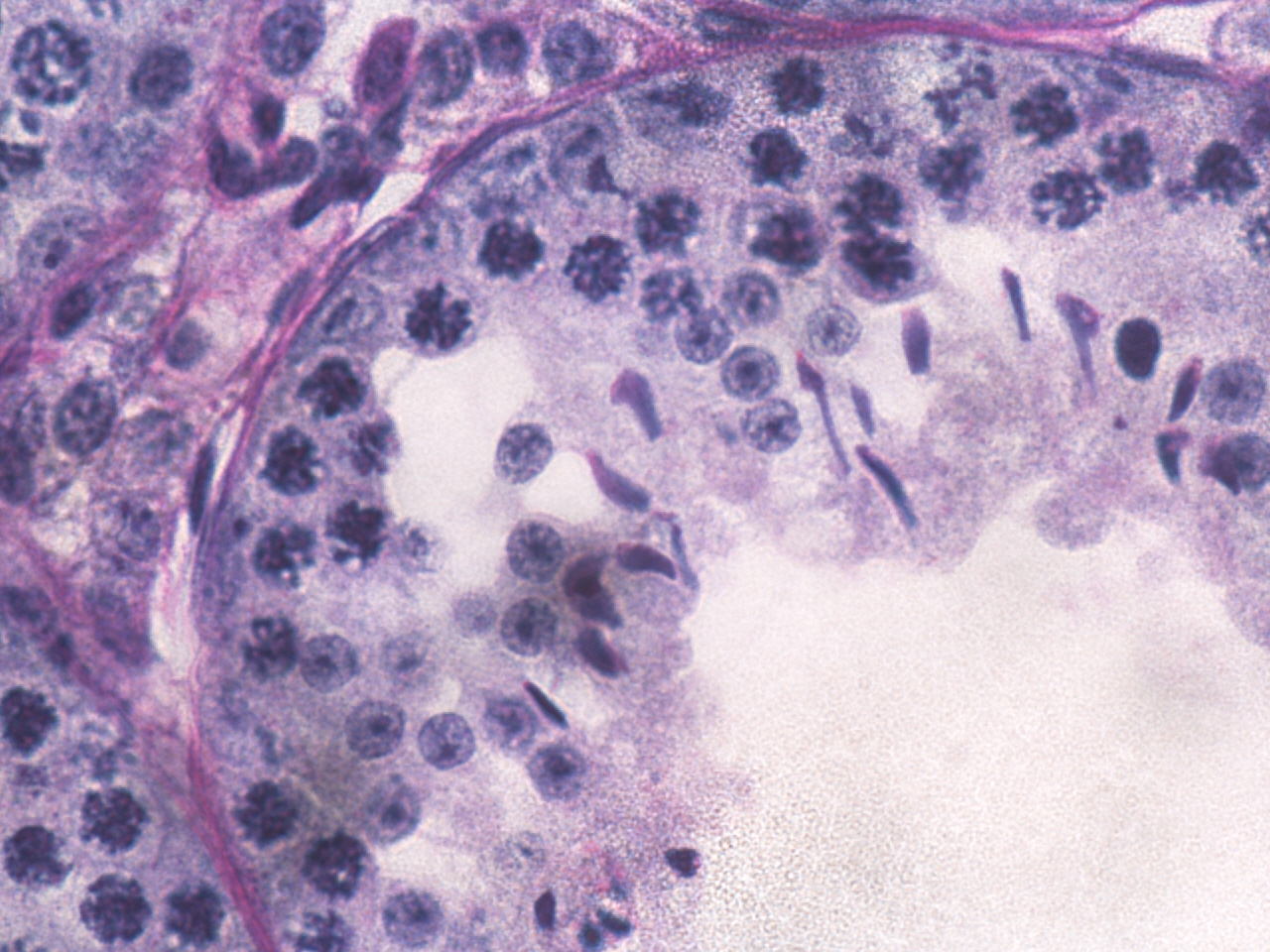

Supplement: Supplementary file 7 [file Data_Sheet_4.zip › Fig3B/ko/16.jpg]

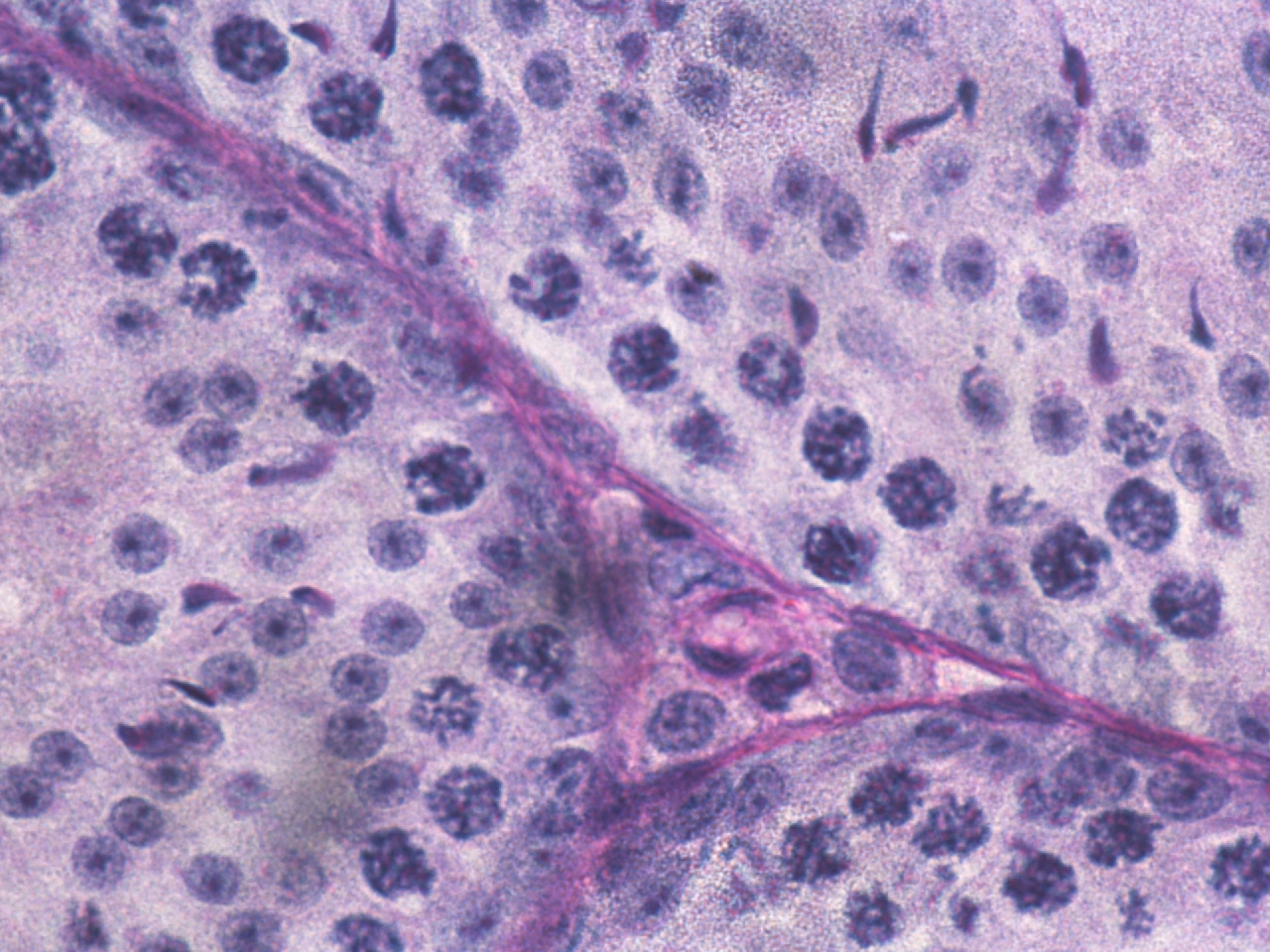

Supplement: Supplementary file 7 [file Data_Sheet_4.zip › Fig3B/ko/2.jpg]

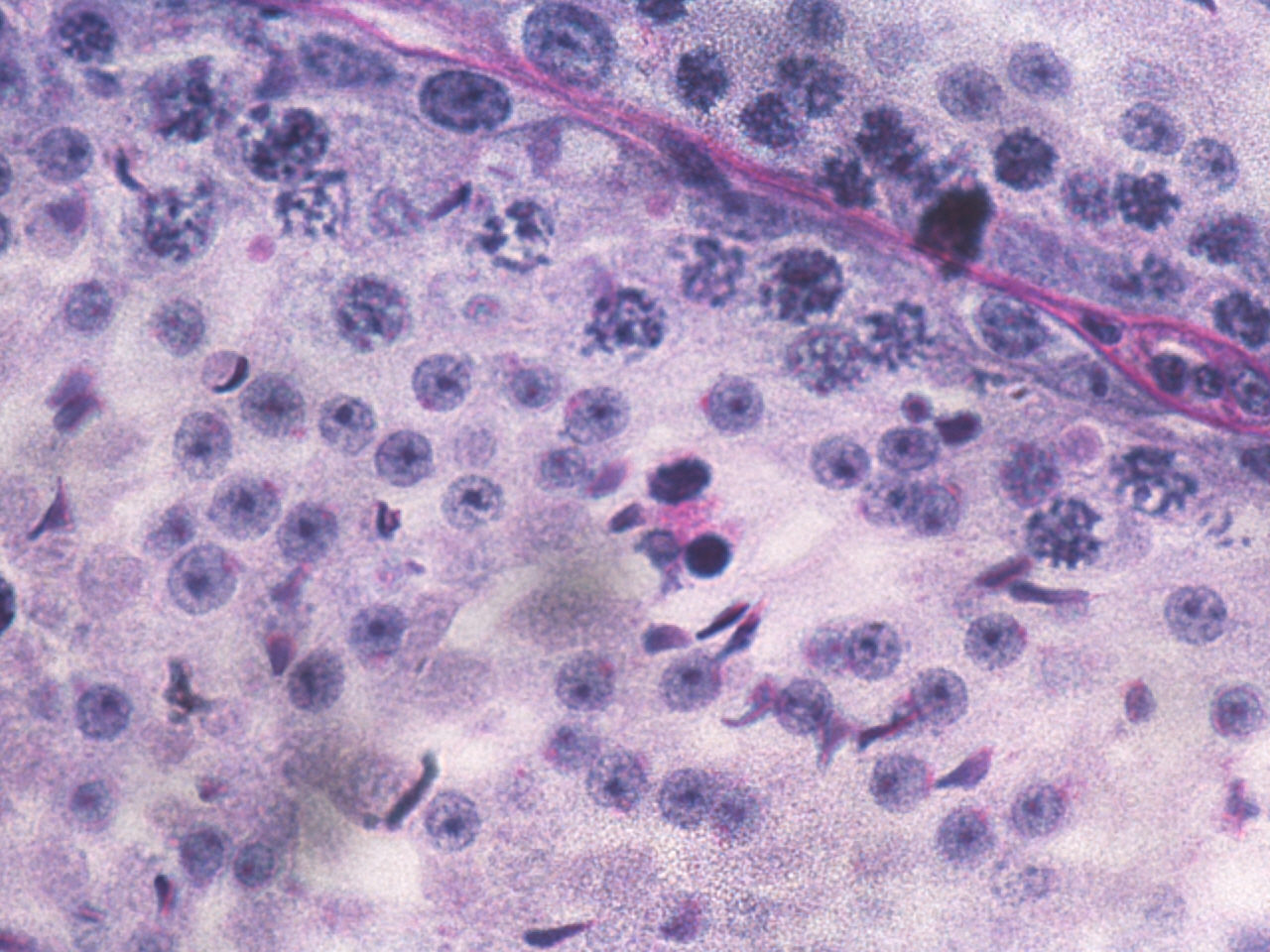

Supplement: Supplementary file 7 [file Data_Sheet_4.zip › Fig3B/ko/3.jpg]

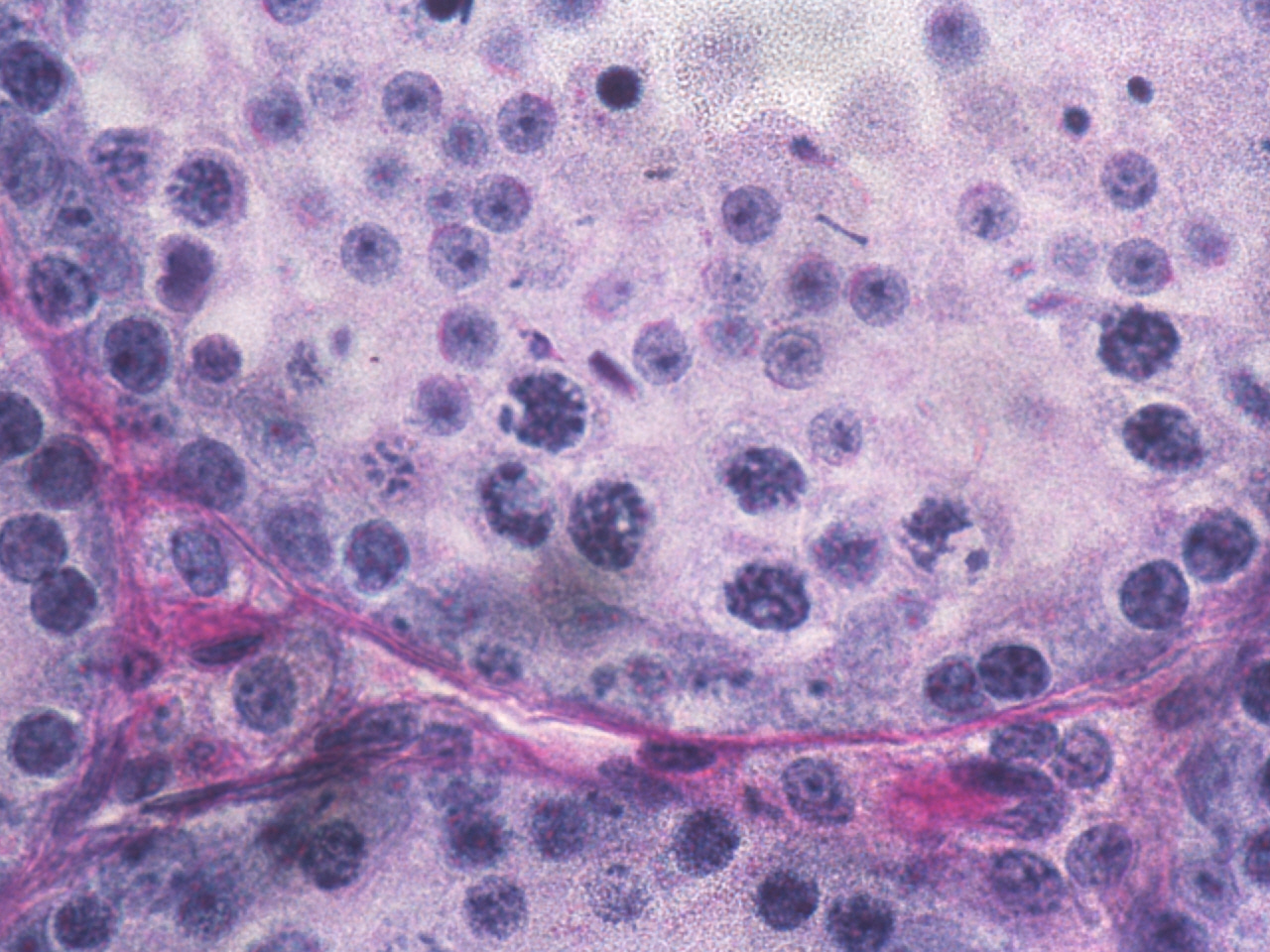

Supplement: Supplementary file 7 [file Data_Sheet_4.zip › Fig3B/ko/7 .jpg]

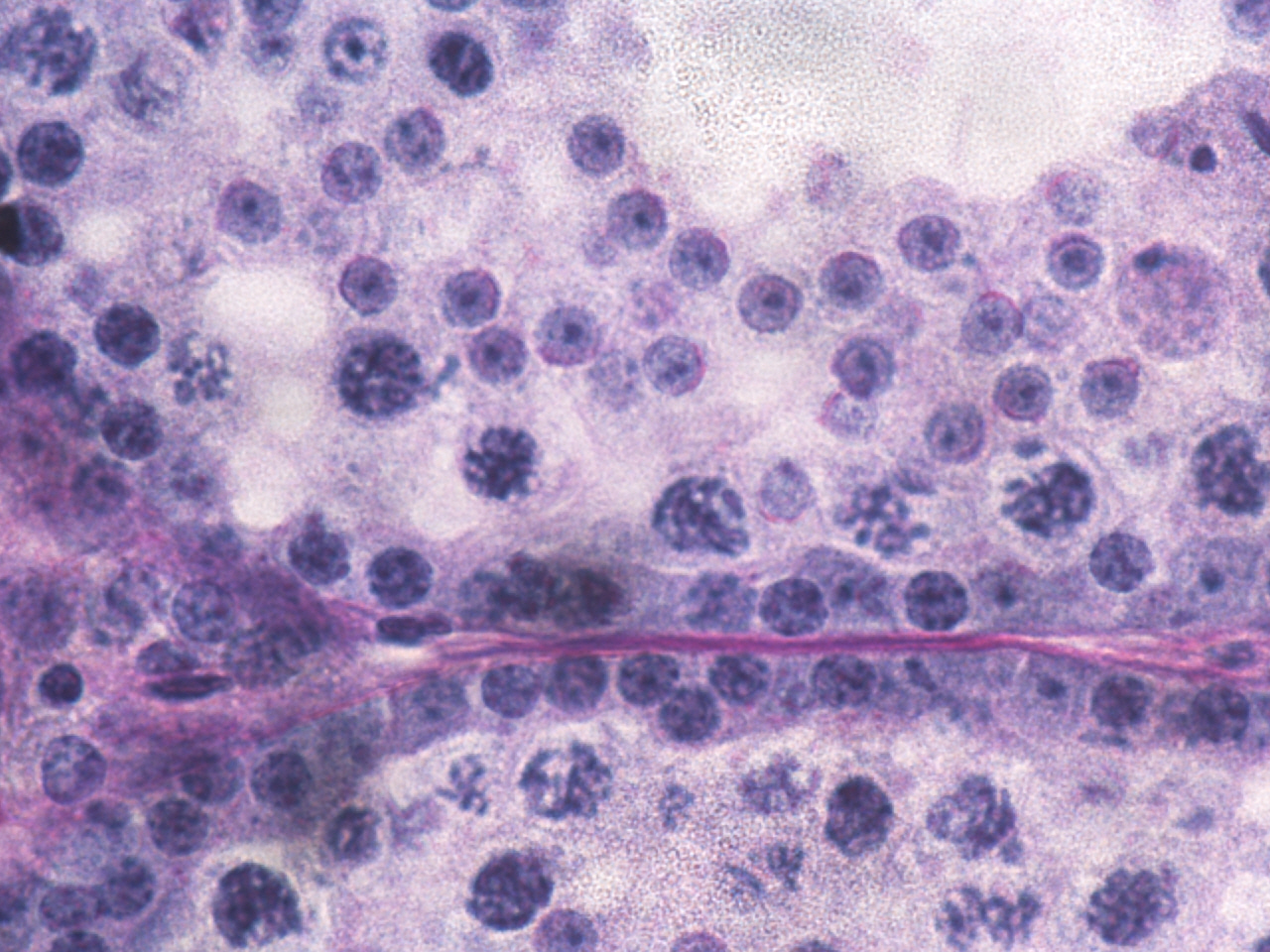

Supplement: Supplementary file 7 [file Data_Sheet_4.zip › Fig3B/ko/8.jpg]

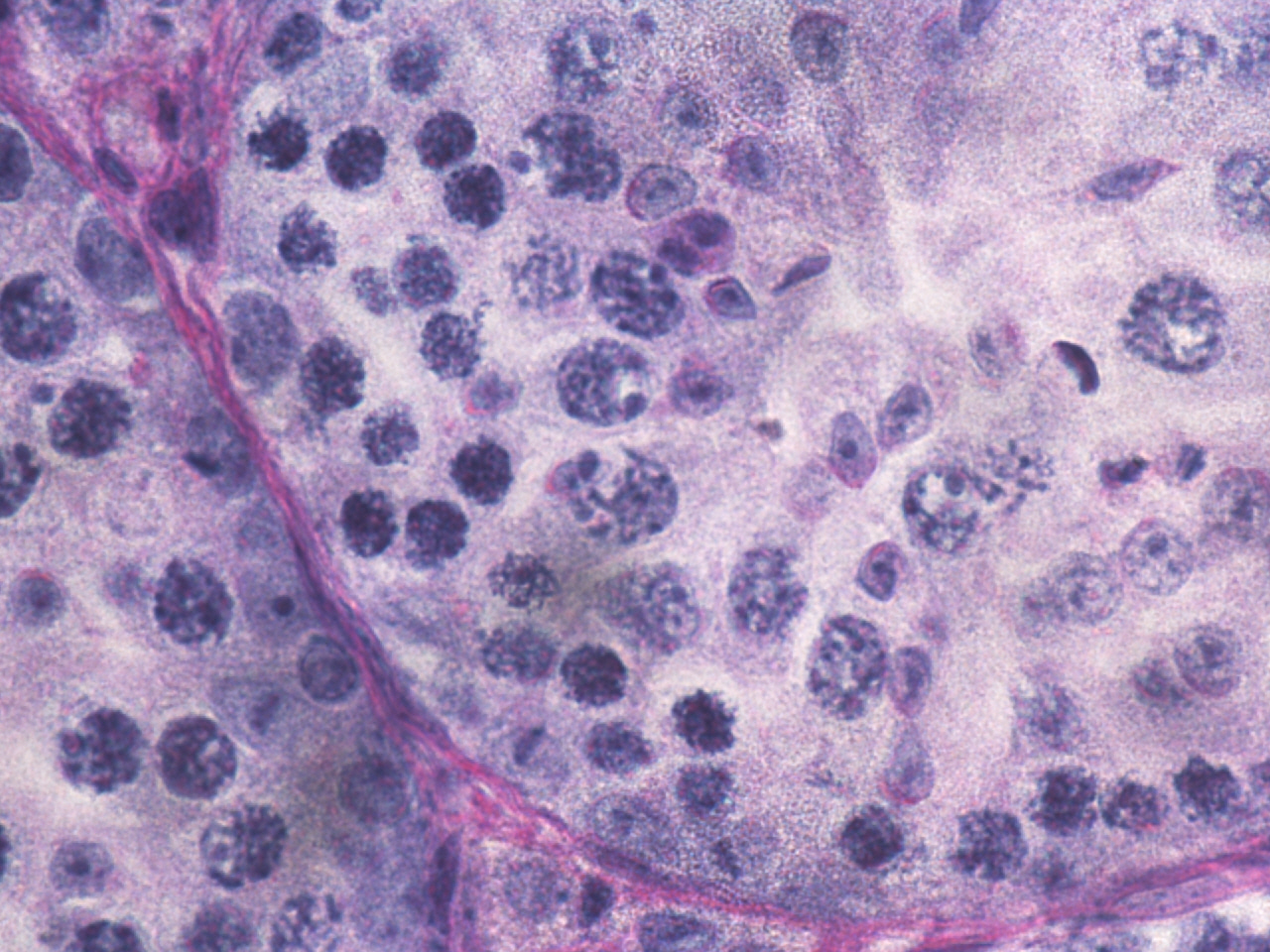

Supplement: Supplementary file 7 [file Data_Sheet_4.zip › Fig3B/ko/9.jpg]

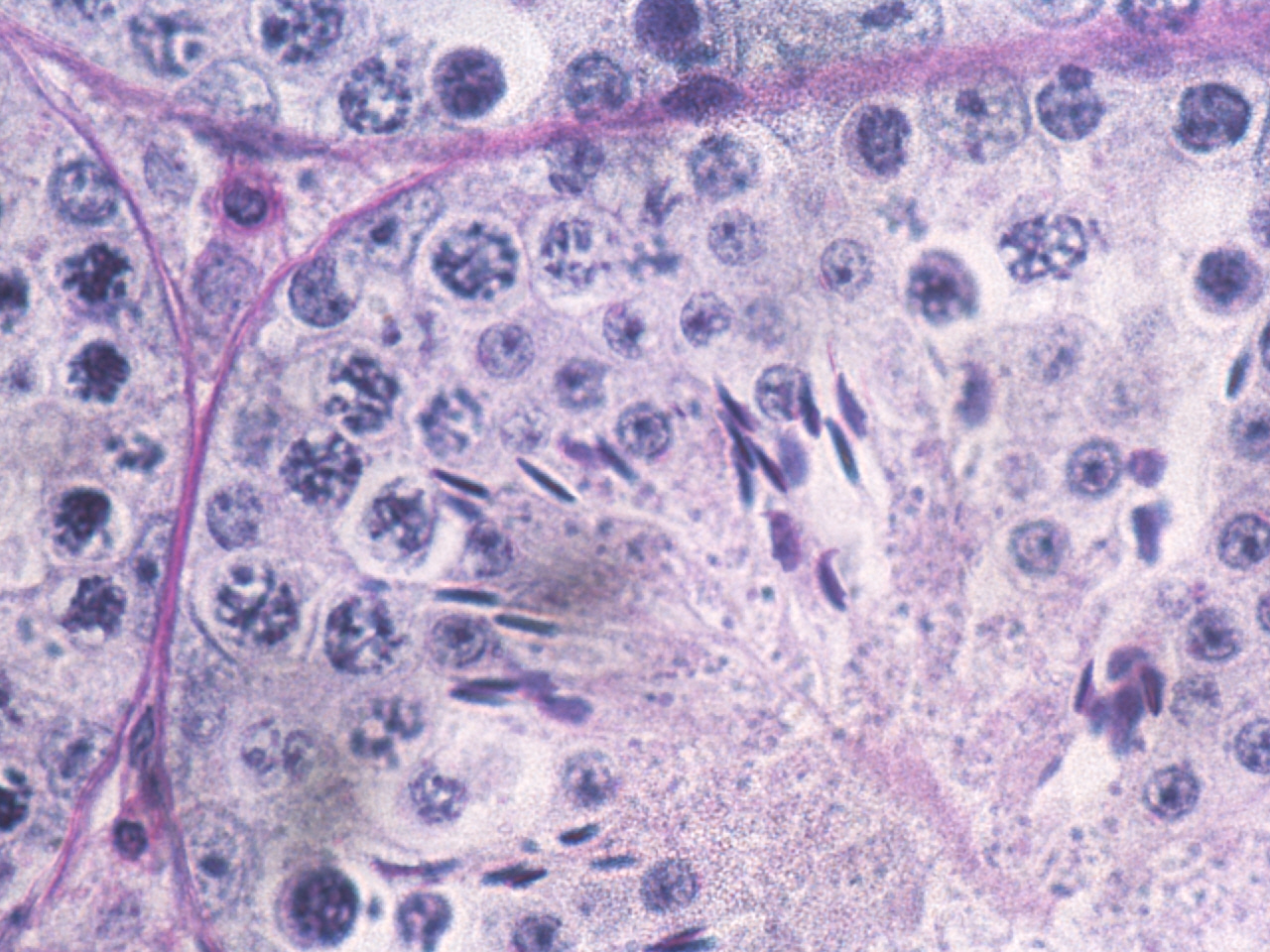

Supplement: Supplementary file 7 [file Data_Sheet_4.zip › Fig3B/wt/wt 1 2.jpg]

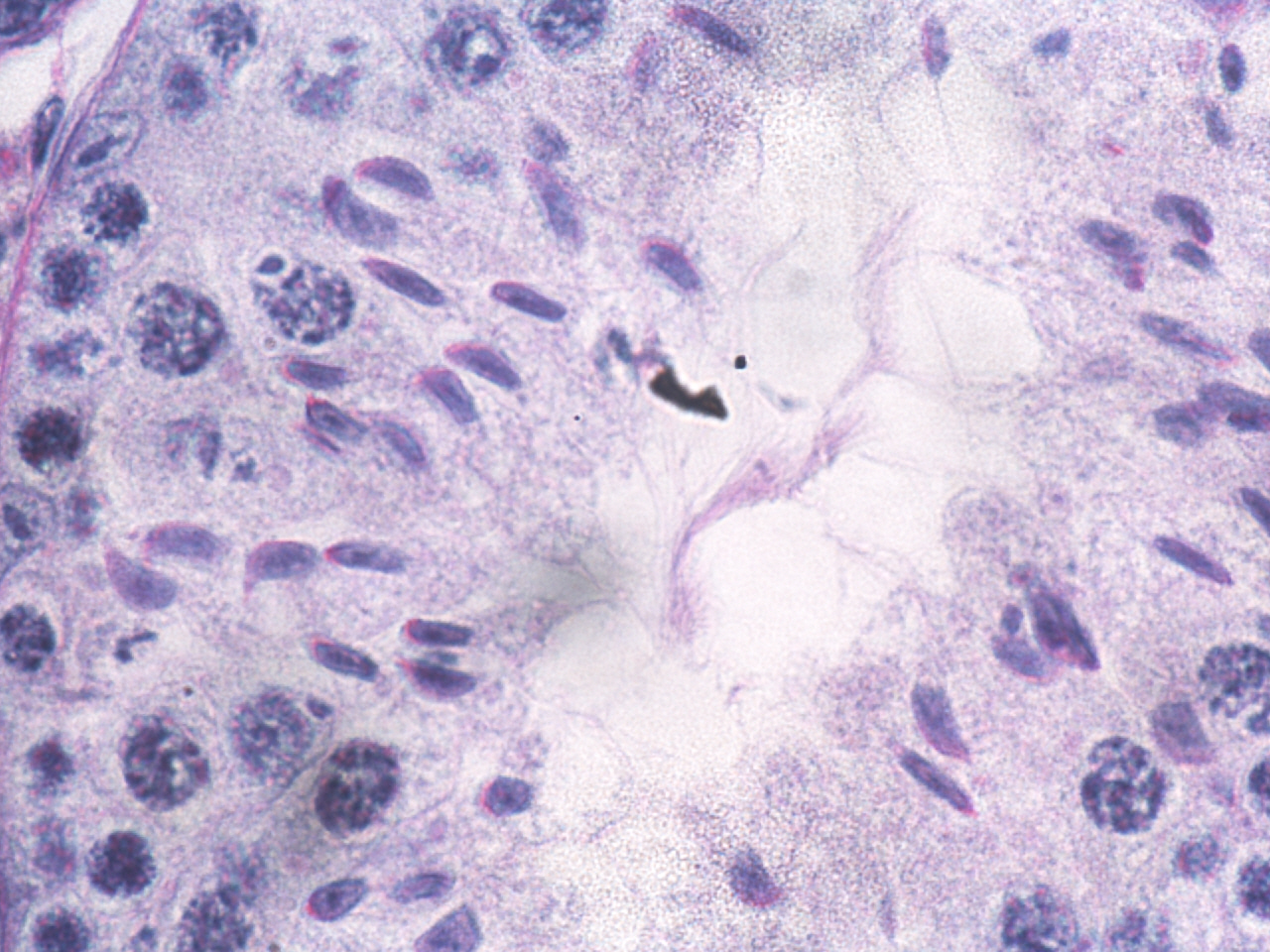

Supplement: Supplementary file 7 [file Data_Sheet_4.zip › Fig3B/wt/wt 10.jpg]

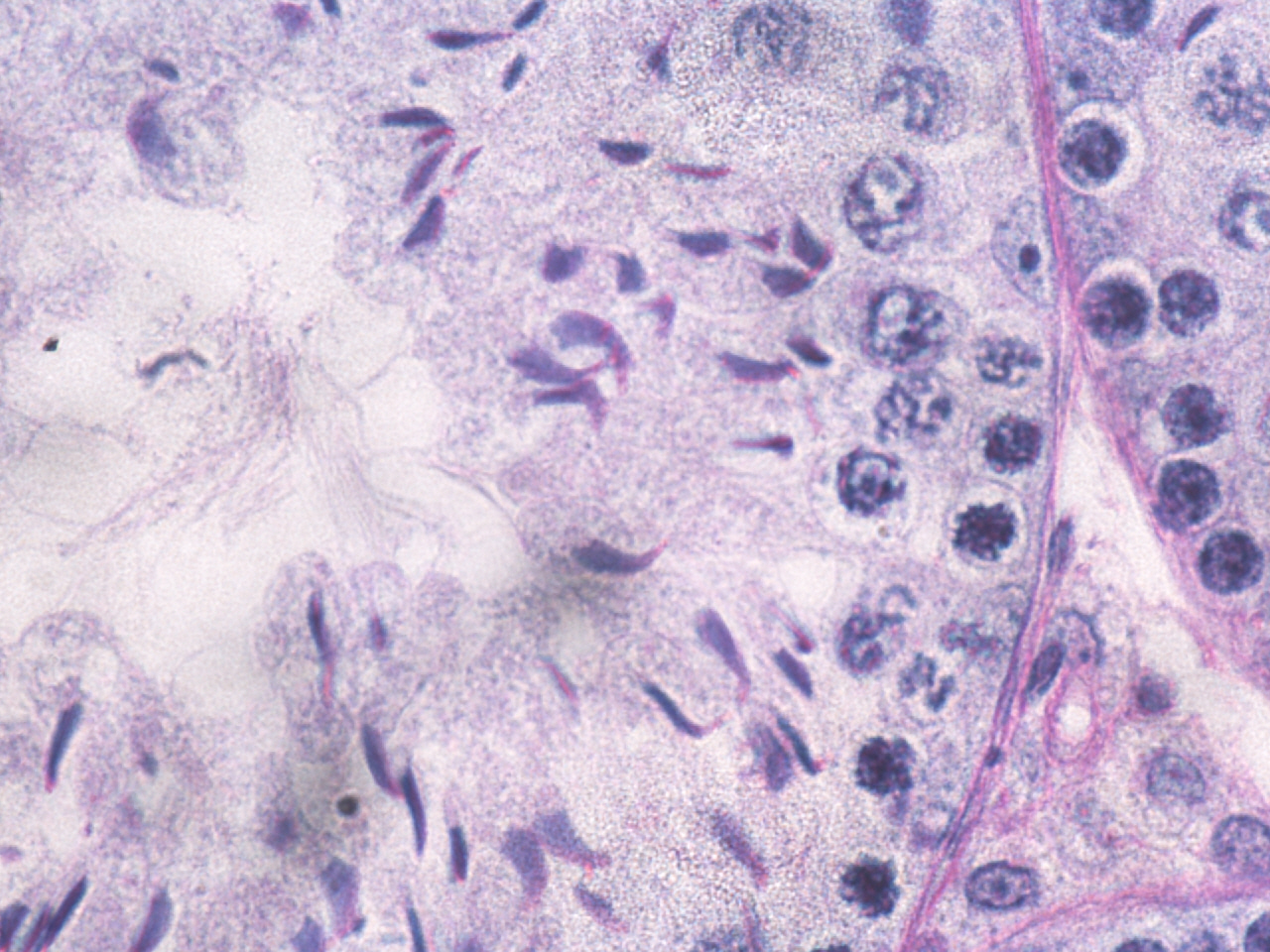

Supplement: Supplementary file 7 [file Data_Sheet_4.zip › Fig3B/wt/wt 11.jpg]

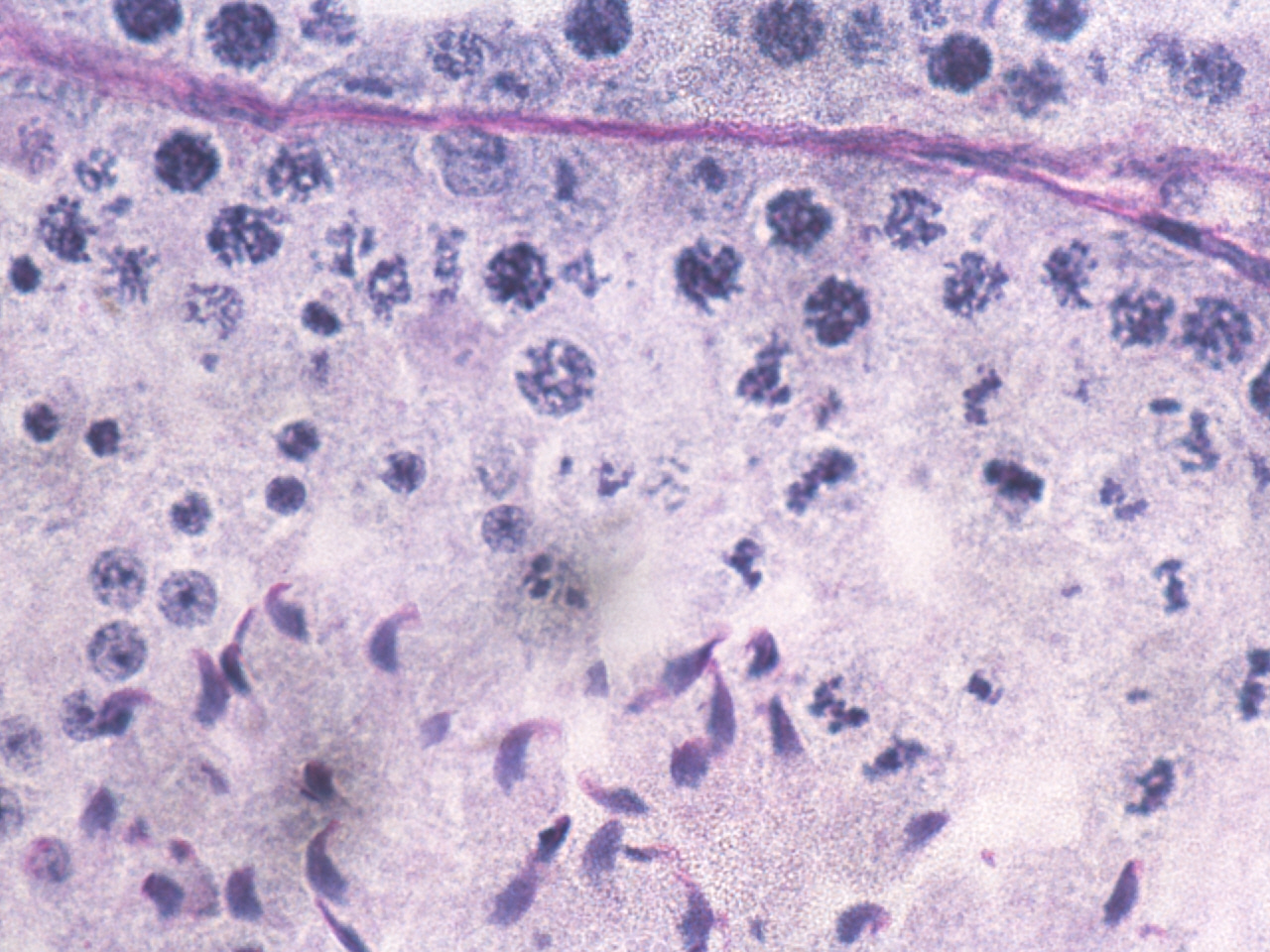

Supplement: Supplementary file 7 [file Data_Sheet_4.zip › Fig3B/wt/wt 12 .jpg]

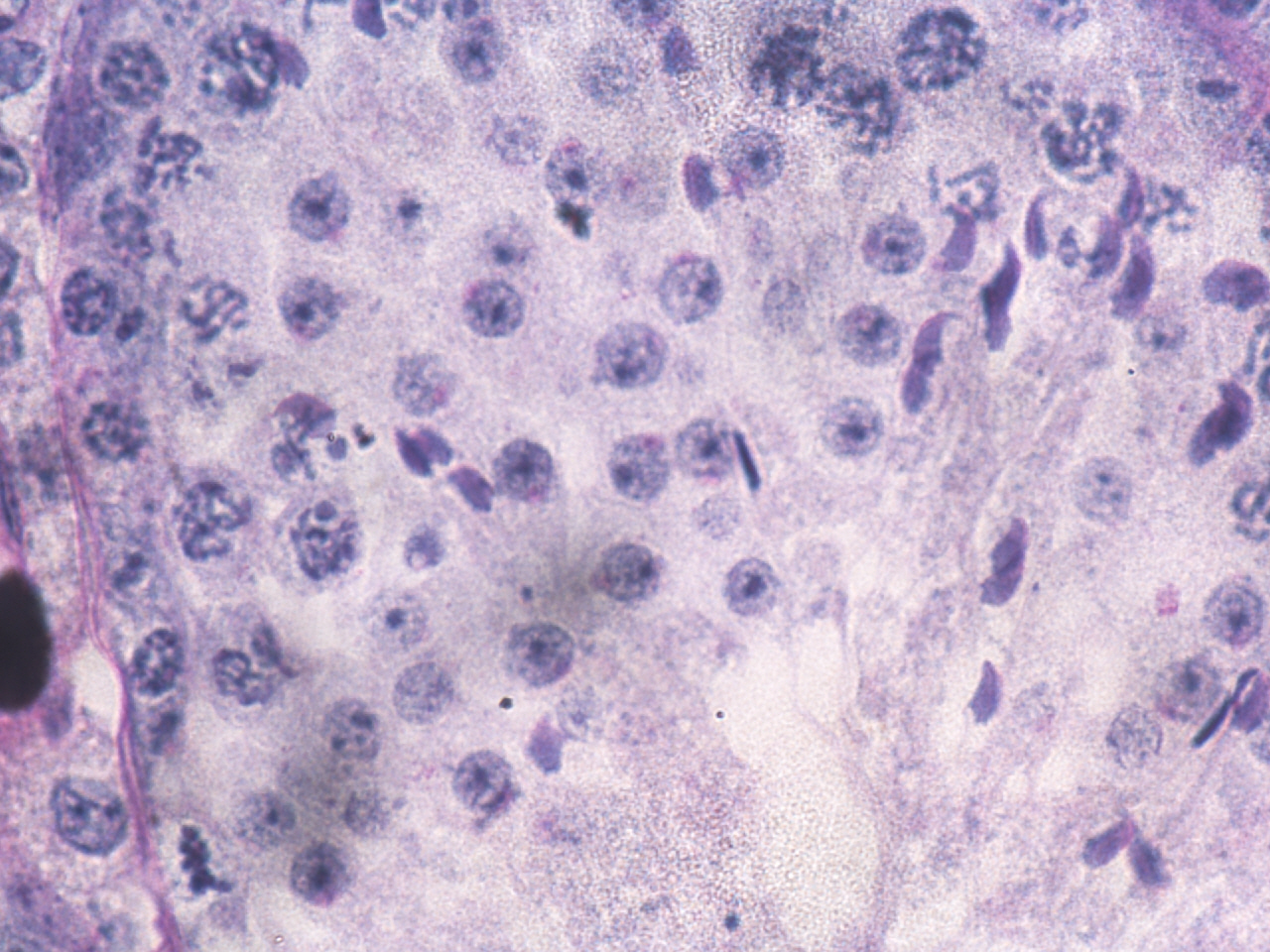

Supplement: Supplementary file 7 [file Data_Sheet_4.zip › Fig3B/wt/wt 13 .jpg]

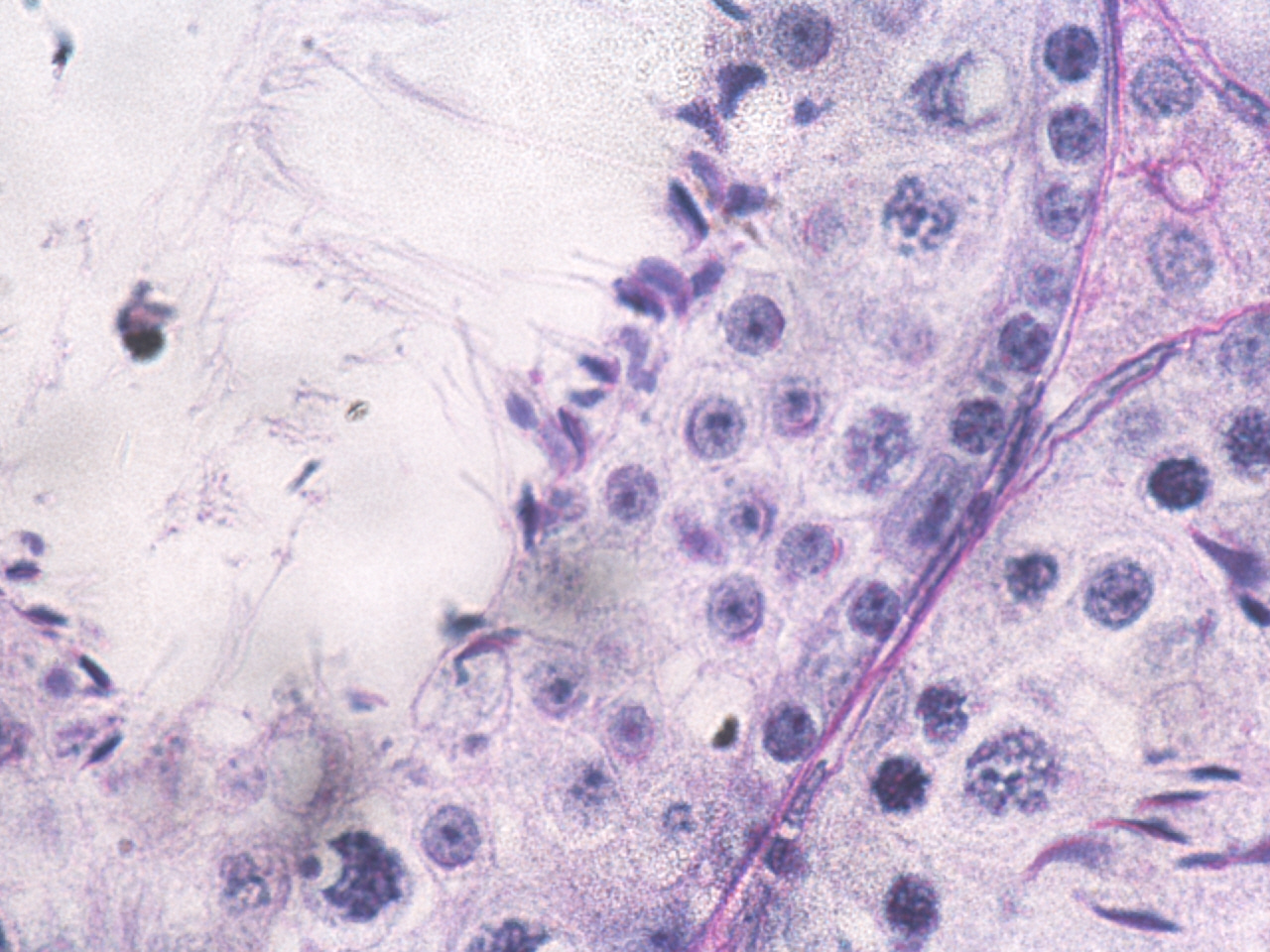

Supplement: Supplementary file 7 [file Data_Sheet_4.zip › Fig3B/wt/wt 15 .jpg]

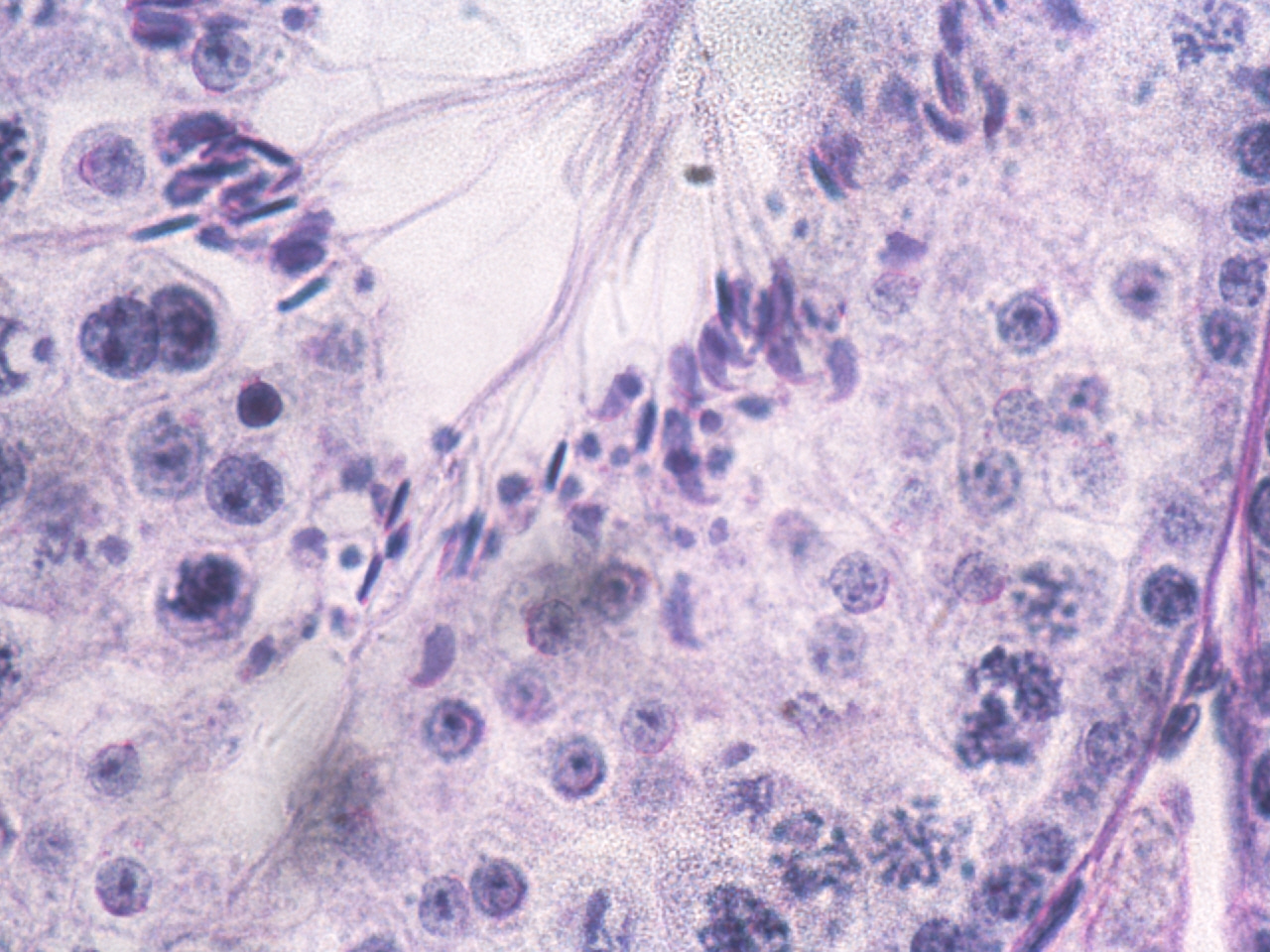

Supplement: Supplementary file 7 [file Data_Sheet_4.zip › Fig3B/wt/wt 16.jpg]

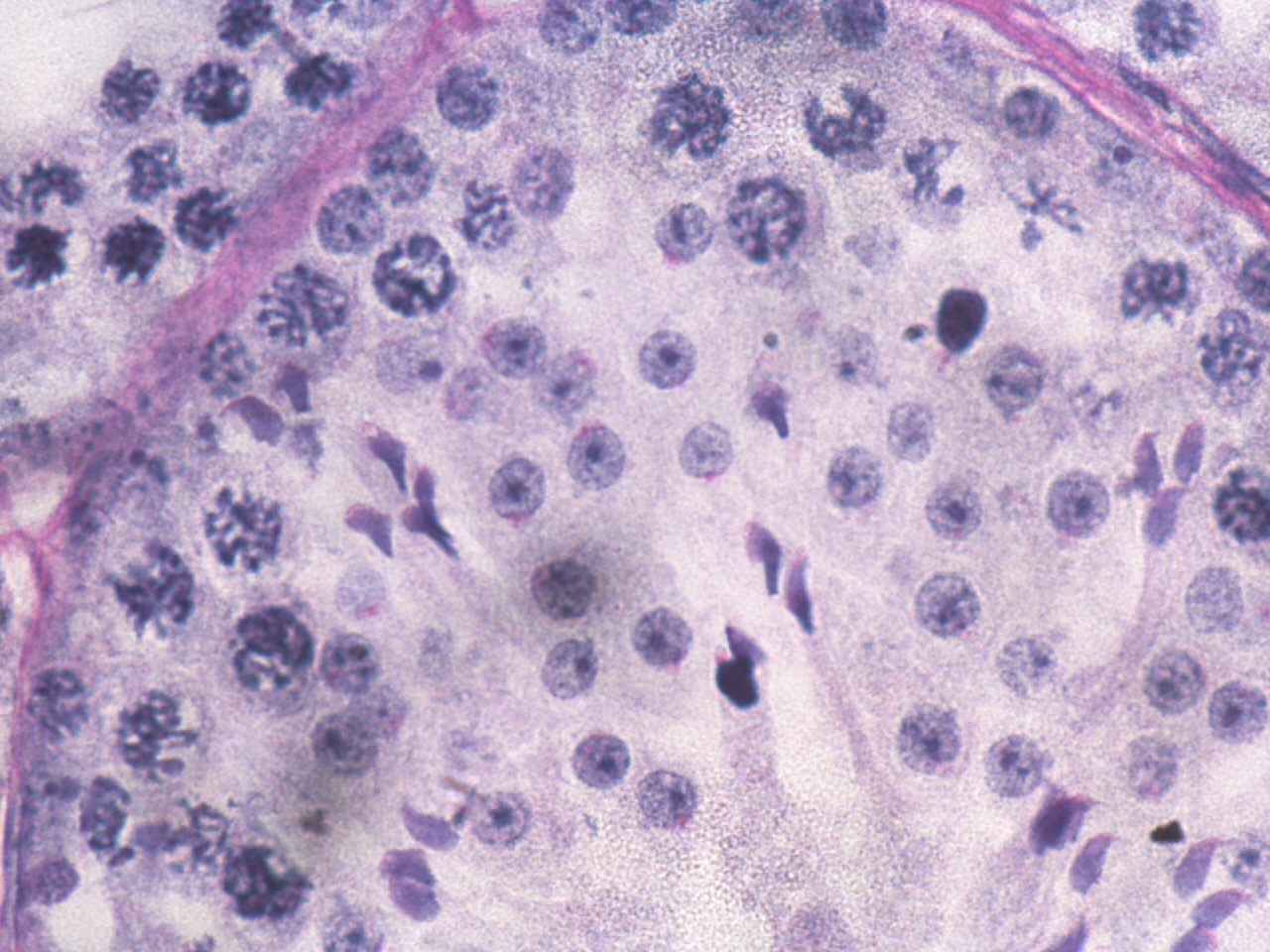

Supplement: Supplementary file 7 [file Data_Sheet_4.zip › Fig3B/wt/wt 4 .jpg]

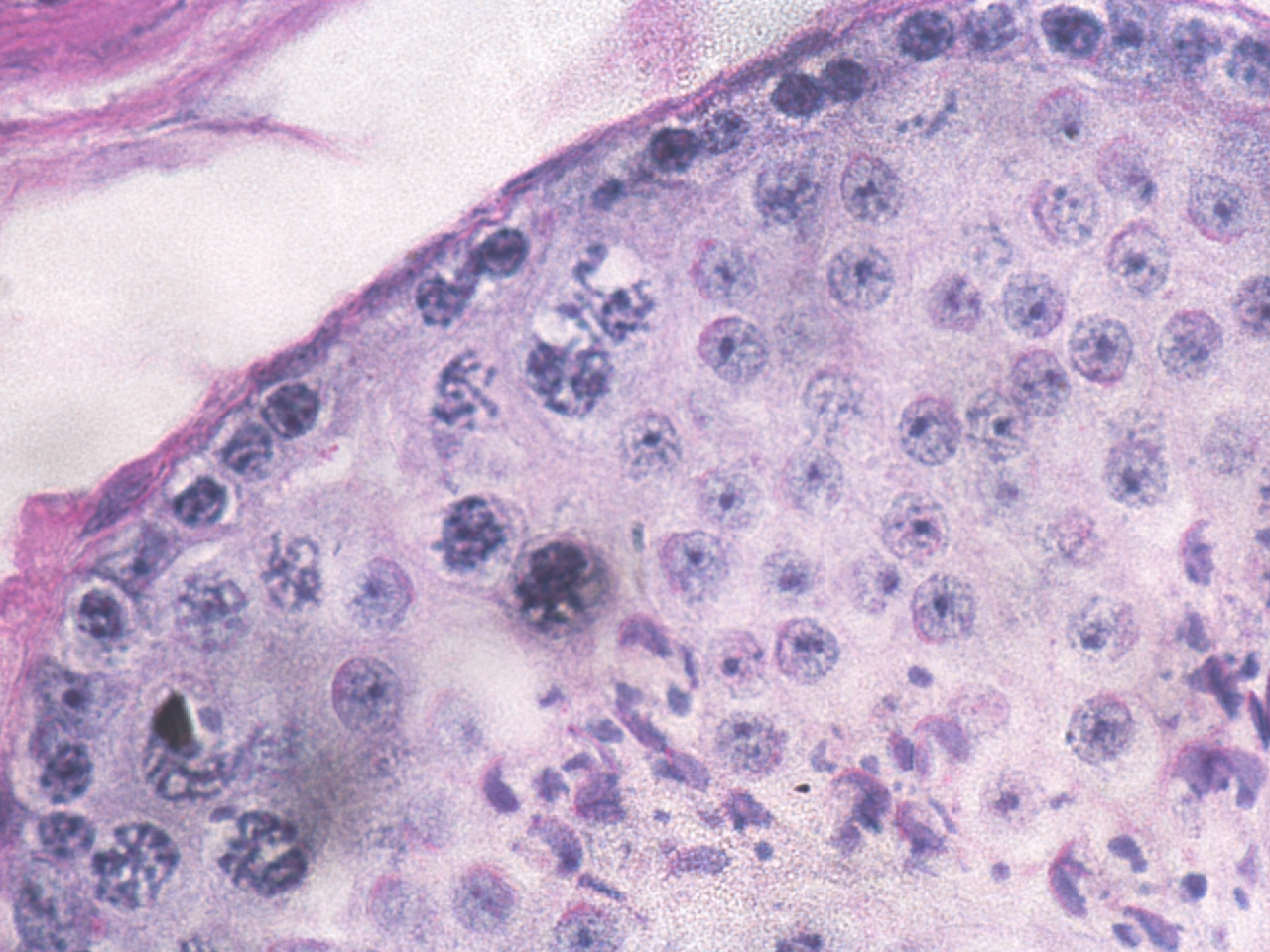

Supplement: Supplementary file 7 [file Data_Sheet_4.zip › Fig3B/wt/wt 5 .jpg]

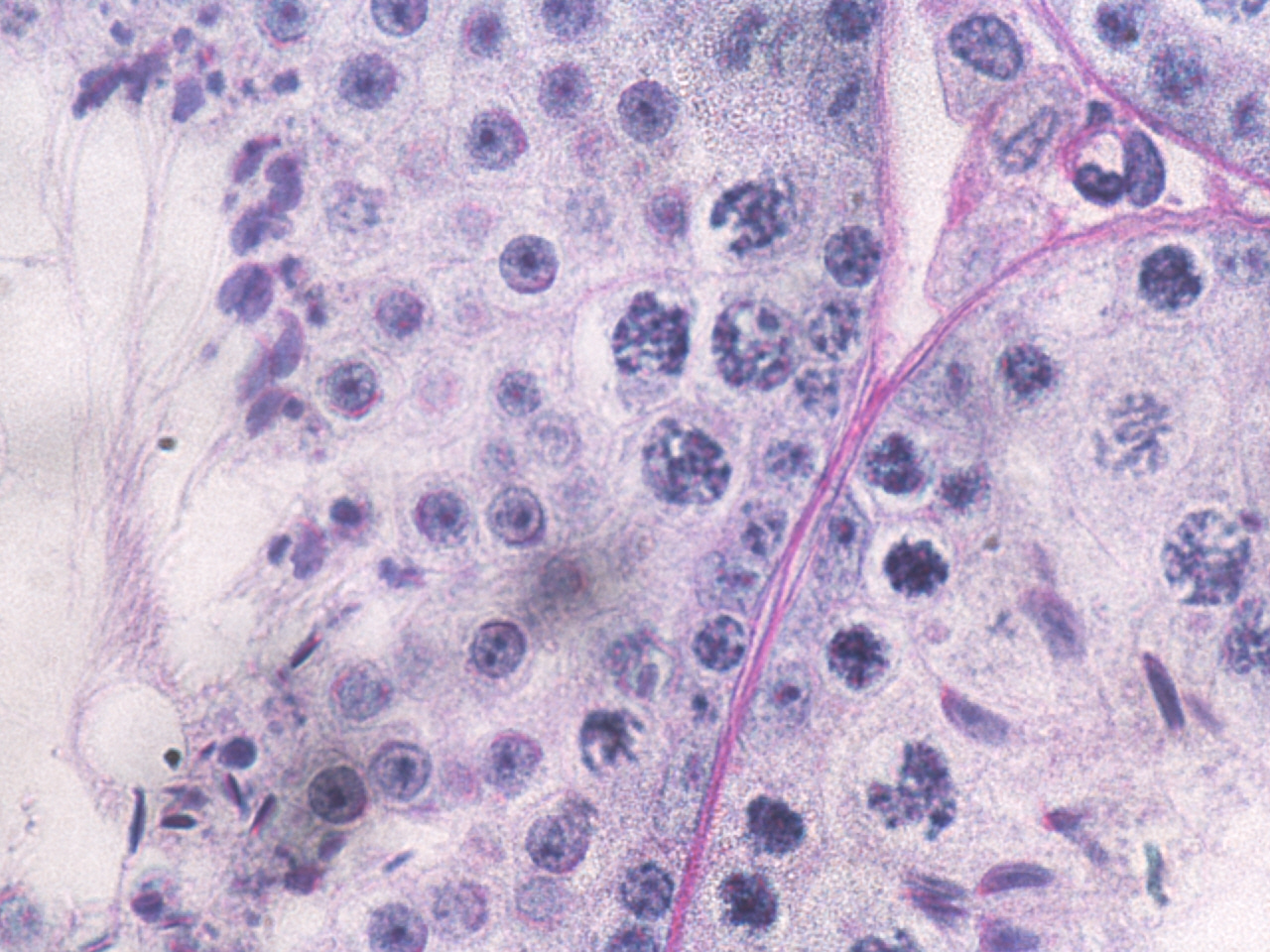

Supplement: Supplementary file 7 [file Data_Sheet_4.zip › Fig3B/wt/wt 6 7 .jpg]

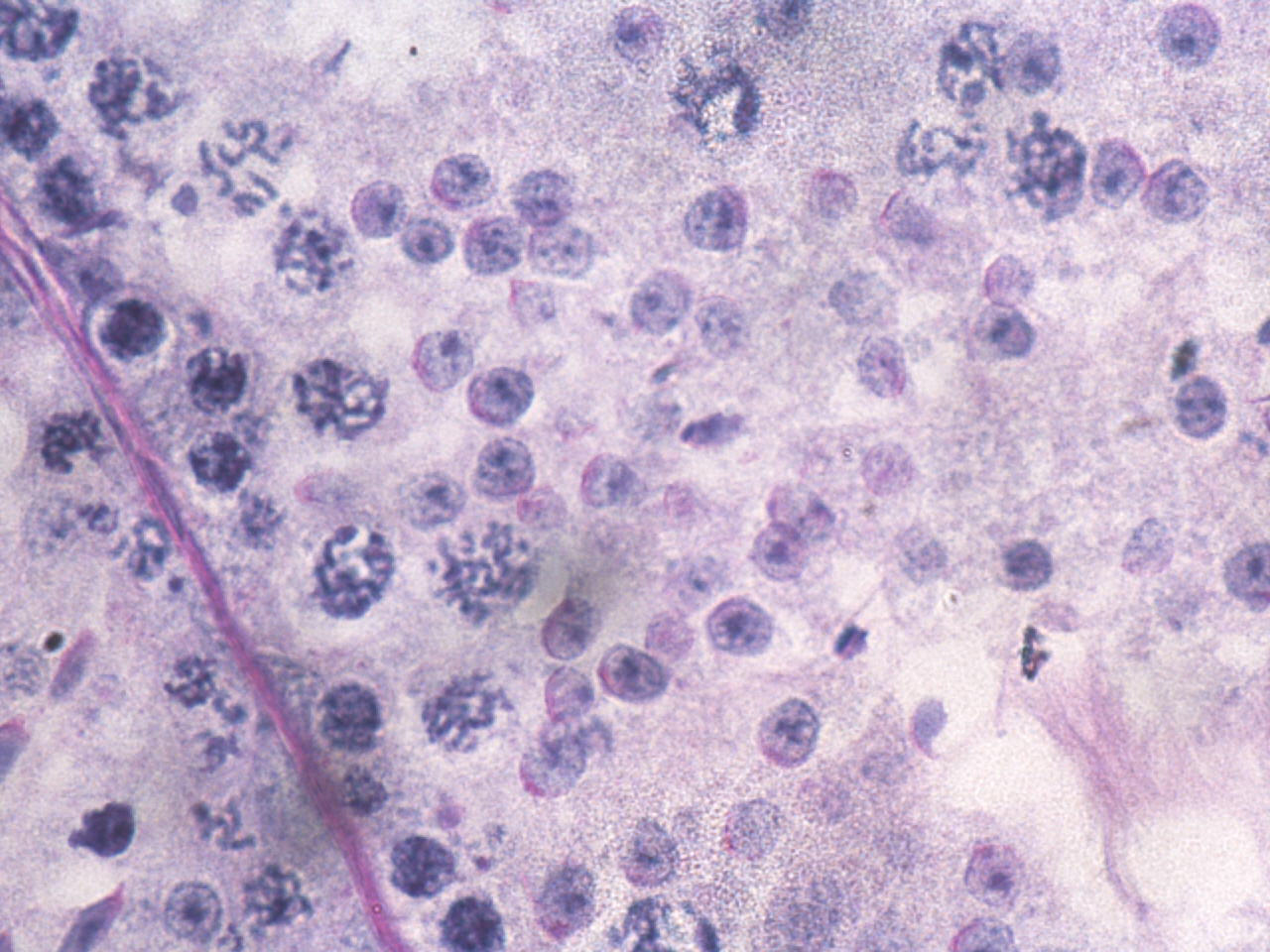

Supplement: Supplementary file 7 [file Data_Sheet_4.zip › Fig3B/wt/wt 8 .jpg]

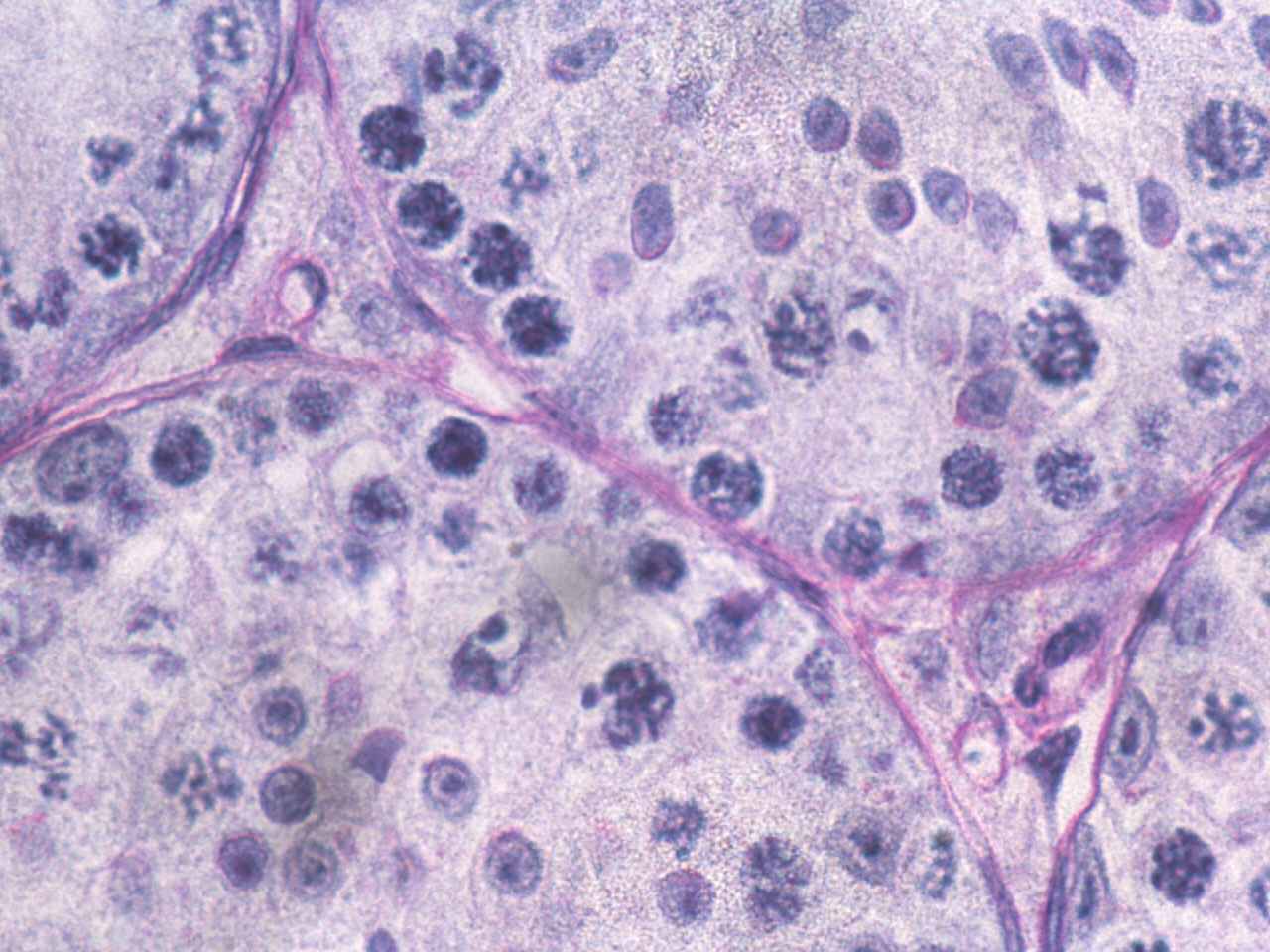

Supplement: Supplementary file 7 [file Data_Sheet_4.zip › Fig3B/wt/wt 9 .jpg]

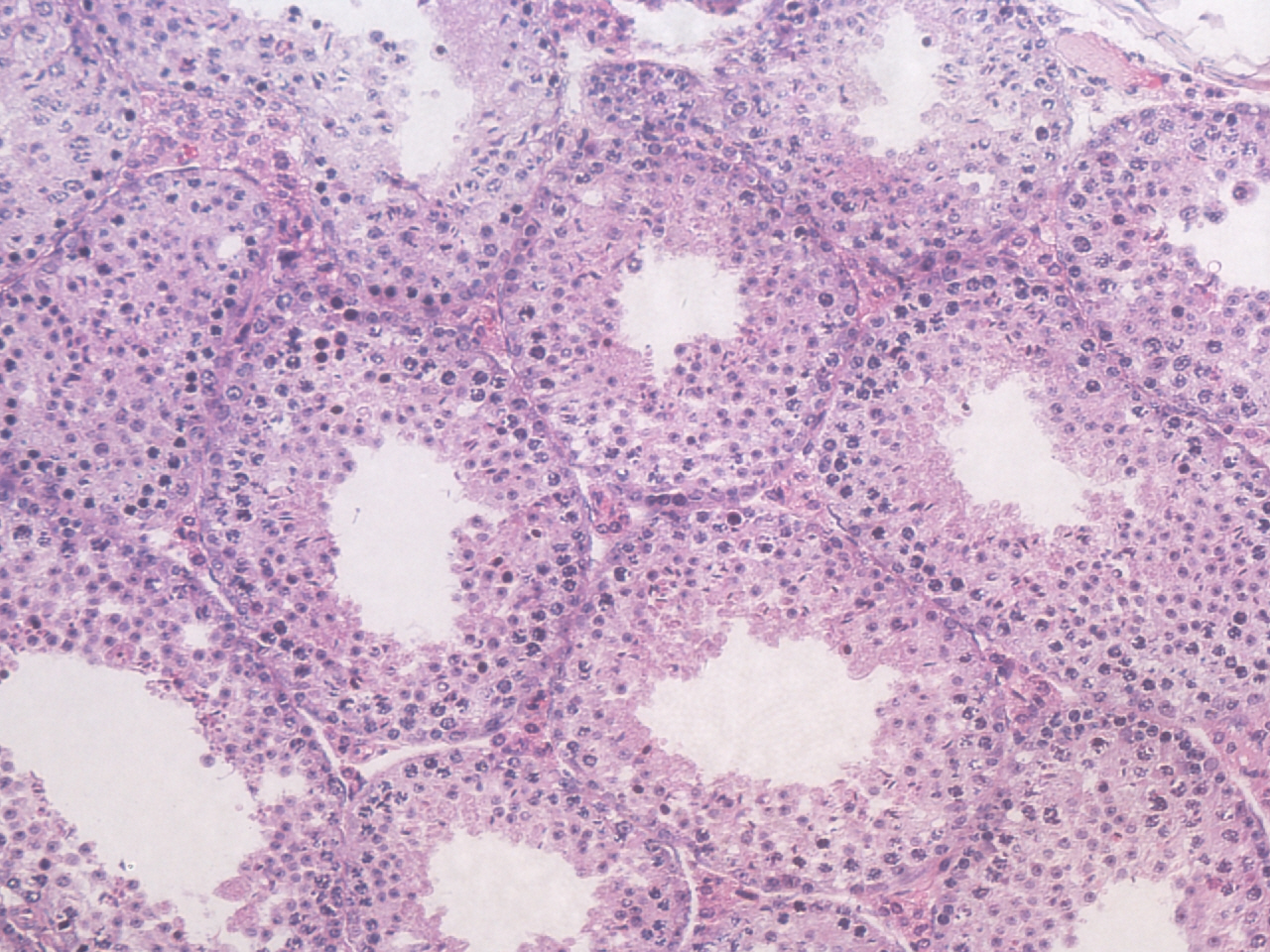

Supplement: Supplementary file 8 [file Data_Sheet_5.ZIP › Fig4A/ko 20.jpg]

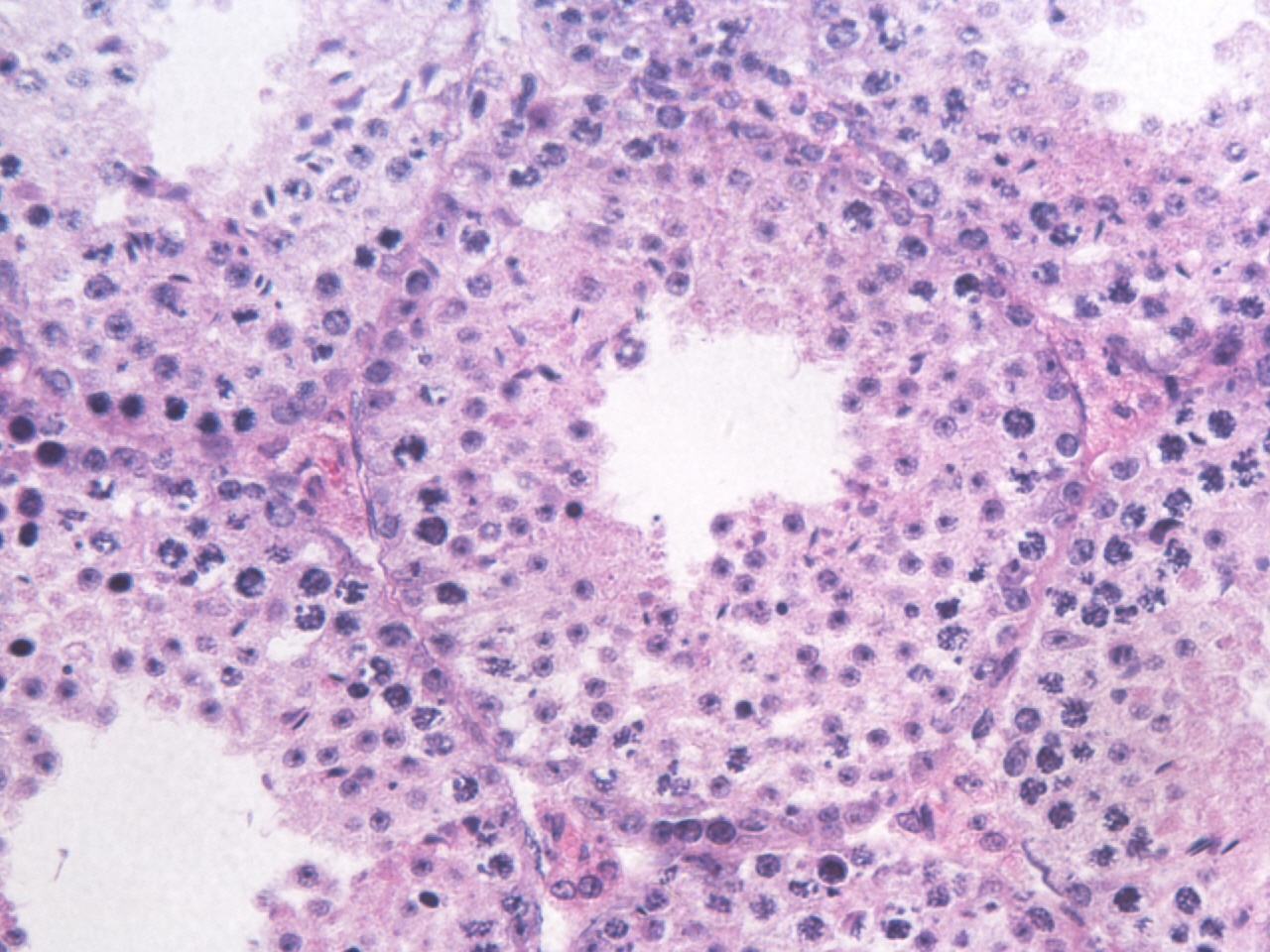

Supplement: Supplementary file 8 [file Data_Sheet_5.ZIP › Fig4A/ko 40.jpg]

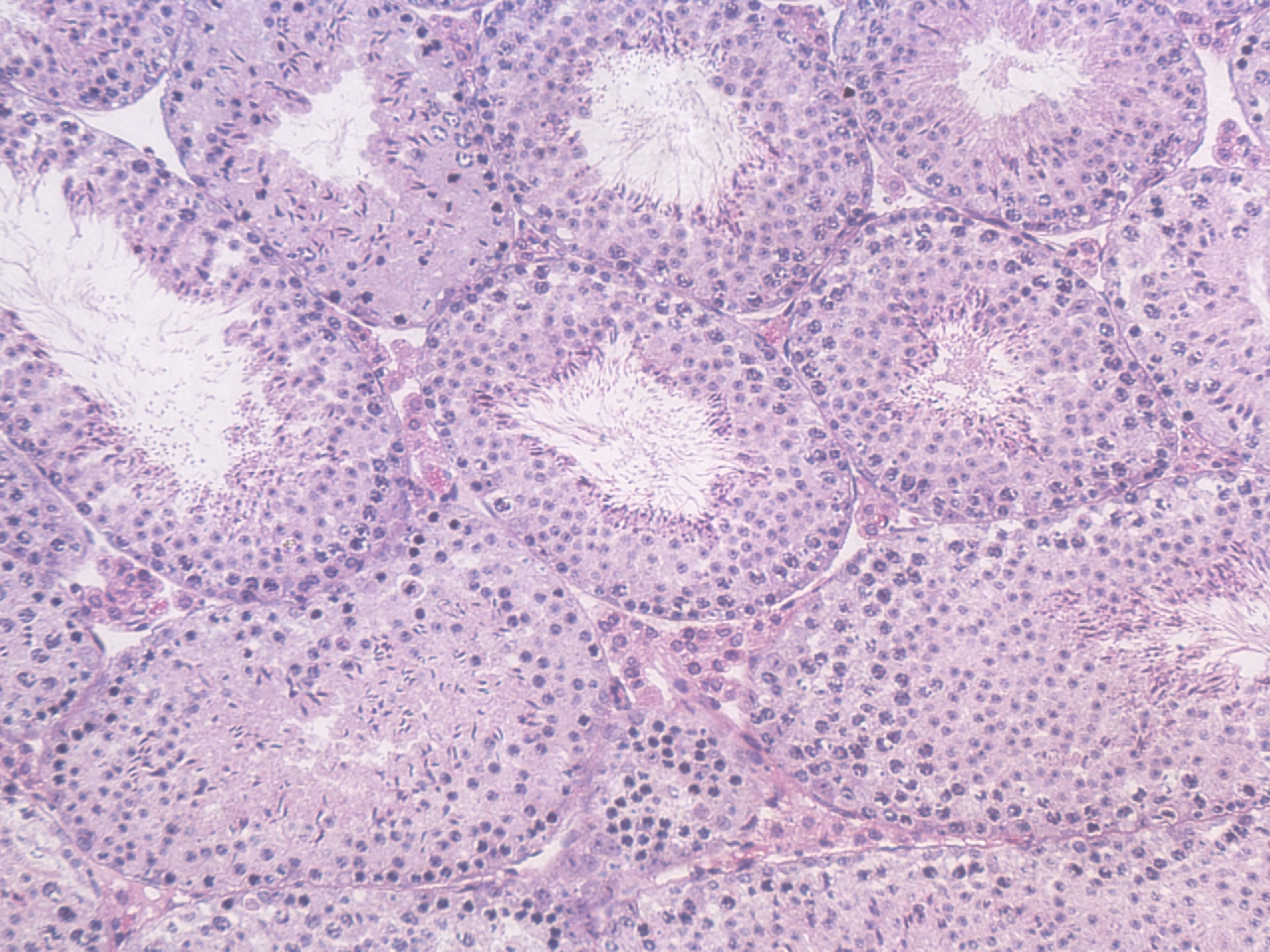

Supplement: Supplementary file 8 [file Data_Sheet_5.ZIP › Fig4A/wt 20.jpg]

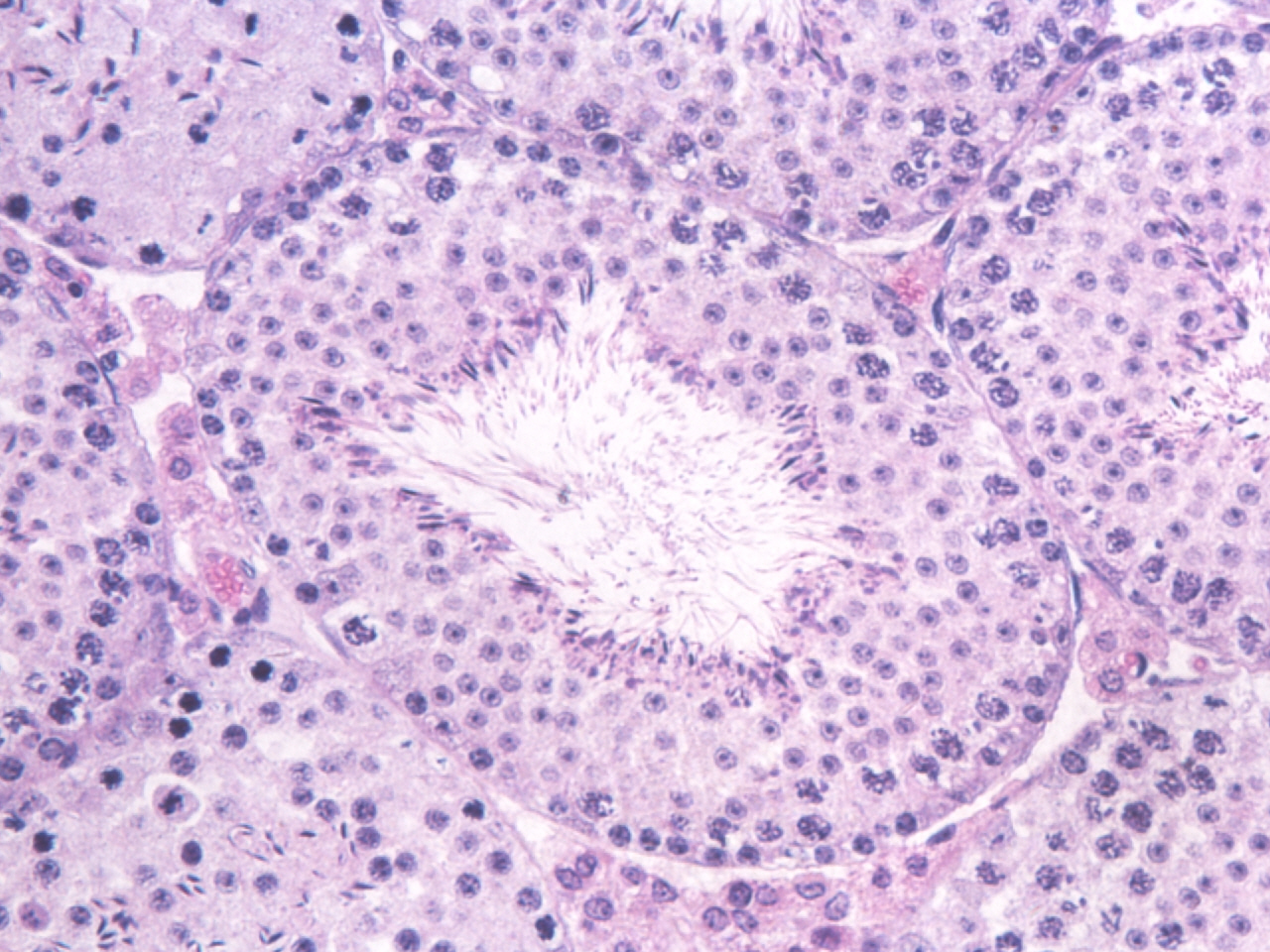

Supplement: Supplementary file 8 [file Data_Sheet_5.ZIP › Fig4A/wt 40.jpg]

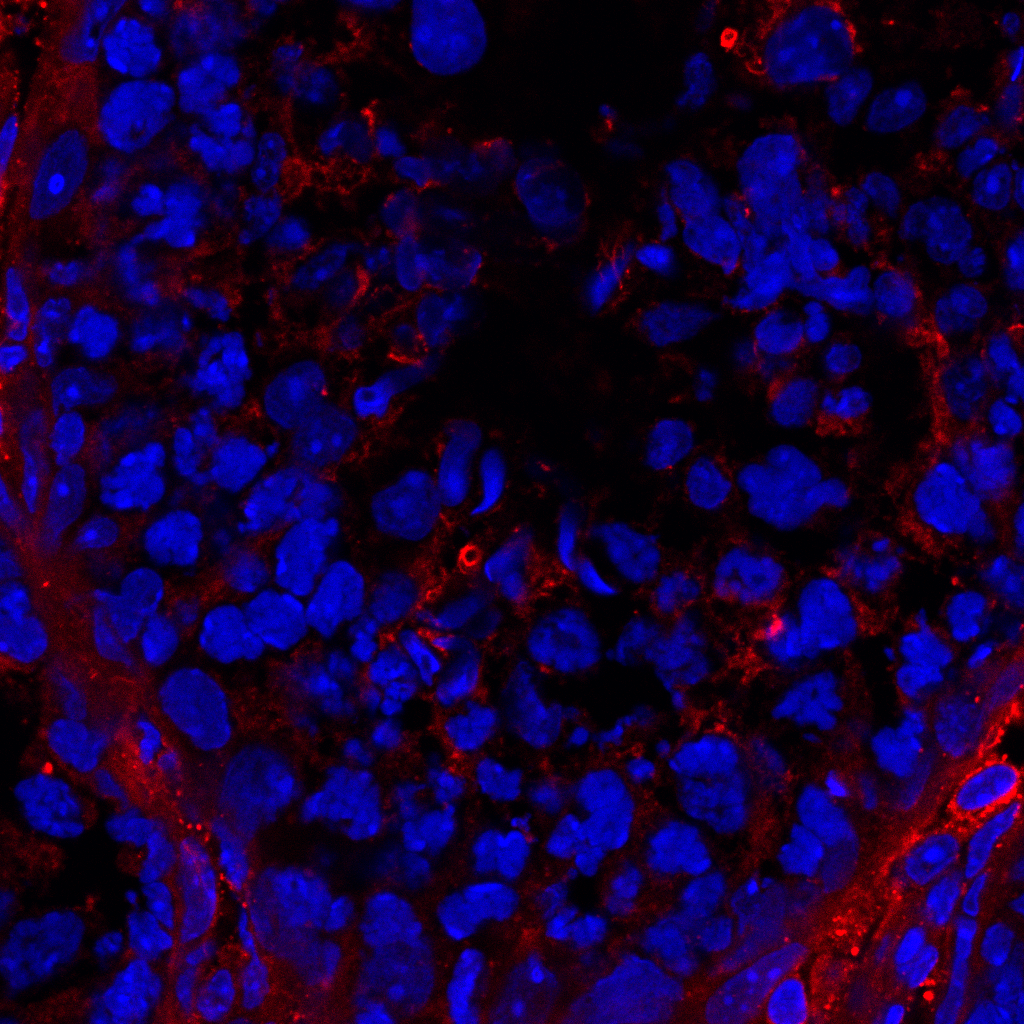

Supplement: Supplementary file 9 [file Data_Sheet_6.ZIP › Fig4B/cf ko tub.lif_Series012_z0.tif]

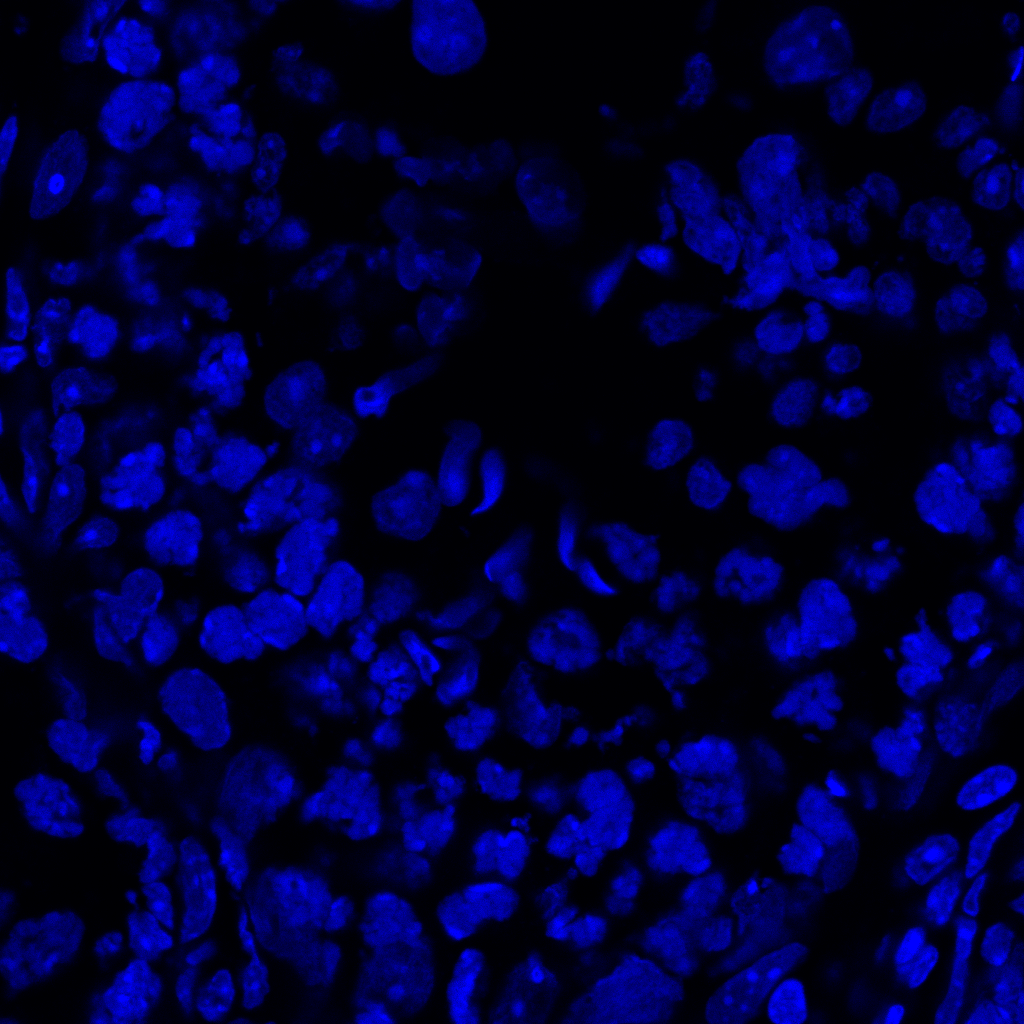

Supplement: Supplementary file 9 [file Data_Sheet_6.ZIP › Fig4B/cf ko tub.lif_Series012_z0_ch00.tif]

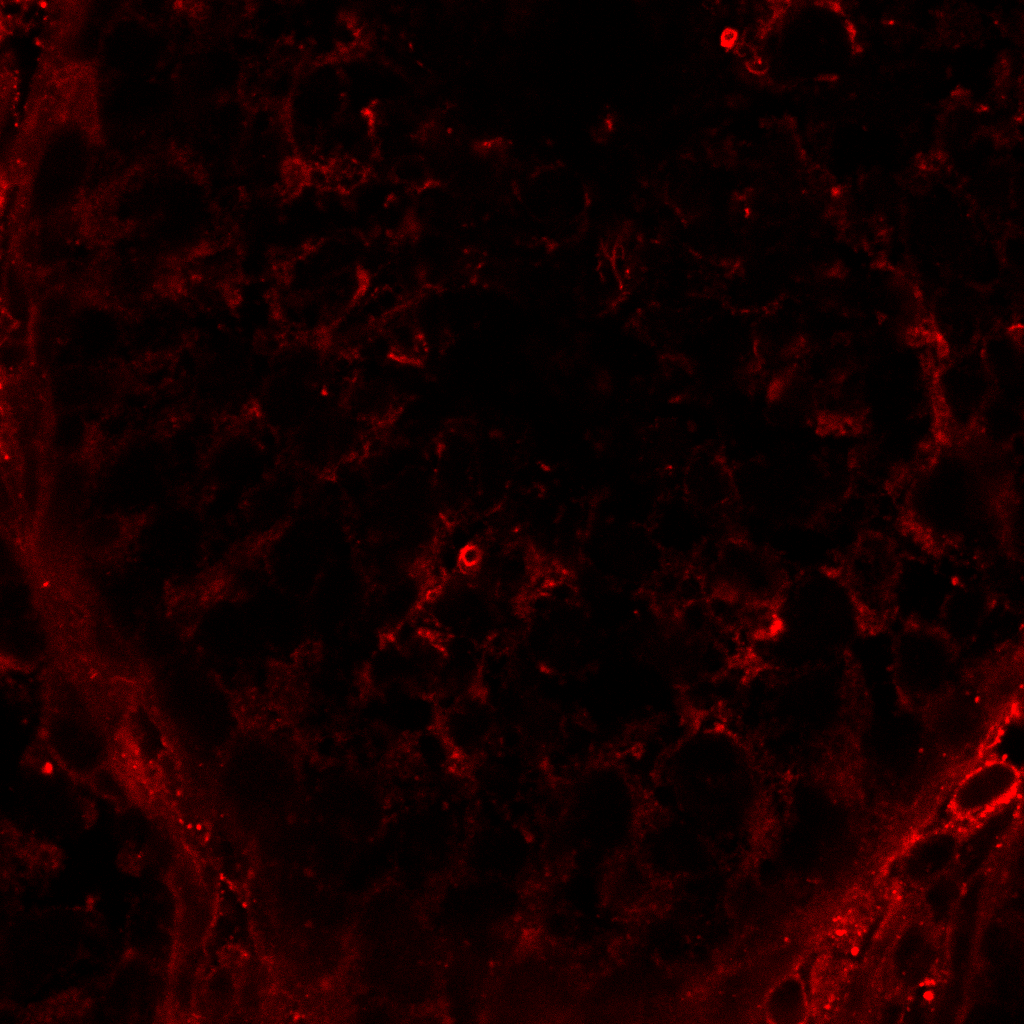

Supplement: Supplementary file 9 [file Data_Sheet_6.ZIP › Fig4B/cf ko tub.lif_Series012_z0_ch02.tif]

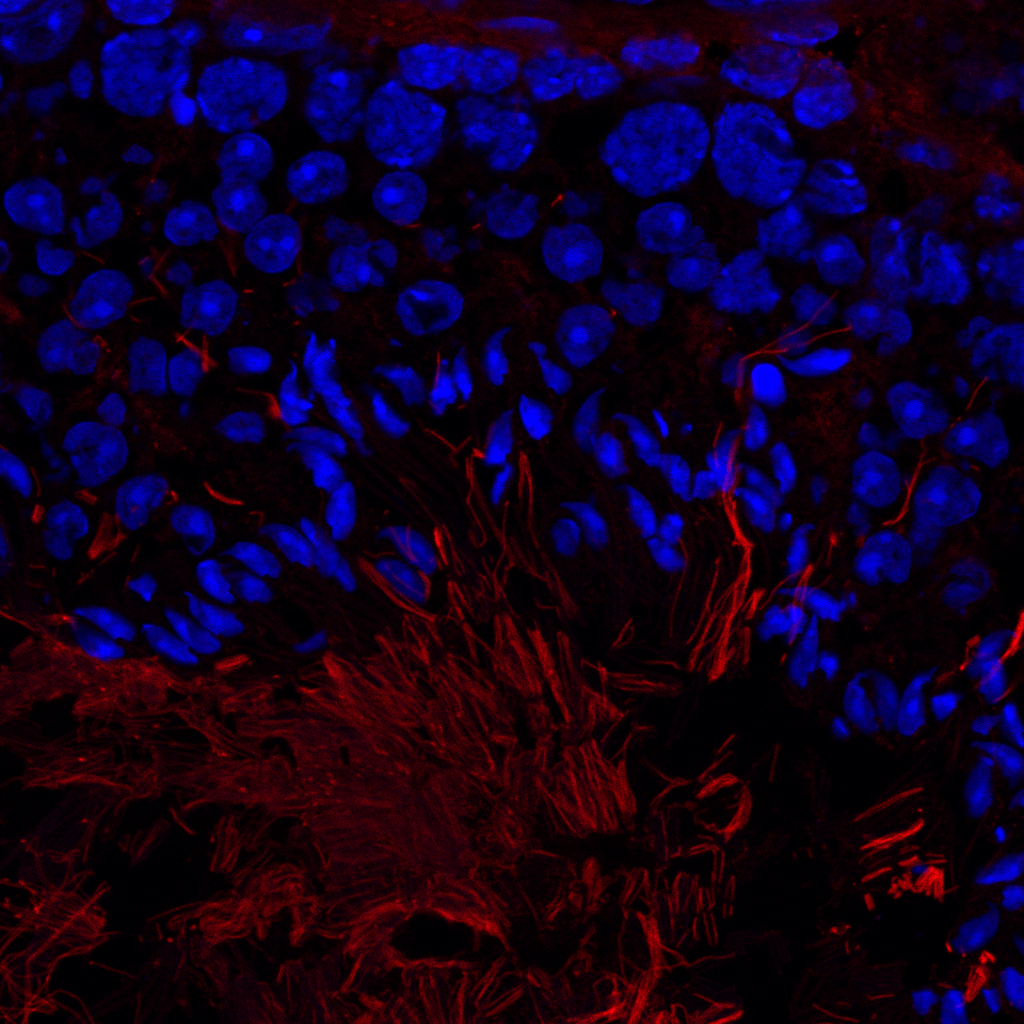

Supplement: Supplementary file 9 [file Data_Sheet_6.ZIP › Fig4B/cf wt tub .lif_Series045_z0.tif]

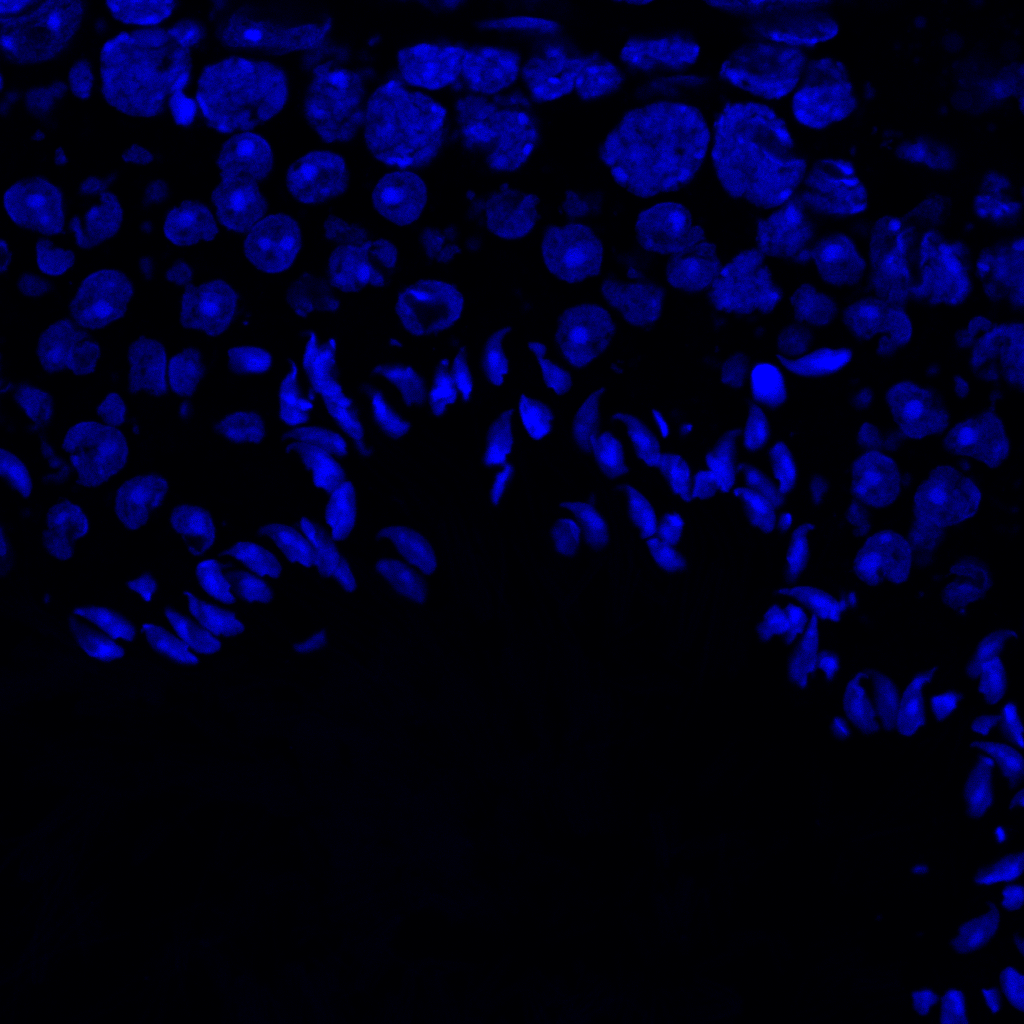

Supplement: Supplementary file 9 [file Data_Sheet_6.ZIP › Fig4B/cf wt tub .lif_Series045_z0_ch00.tif]

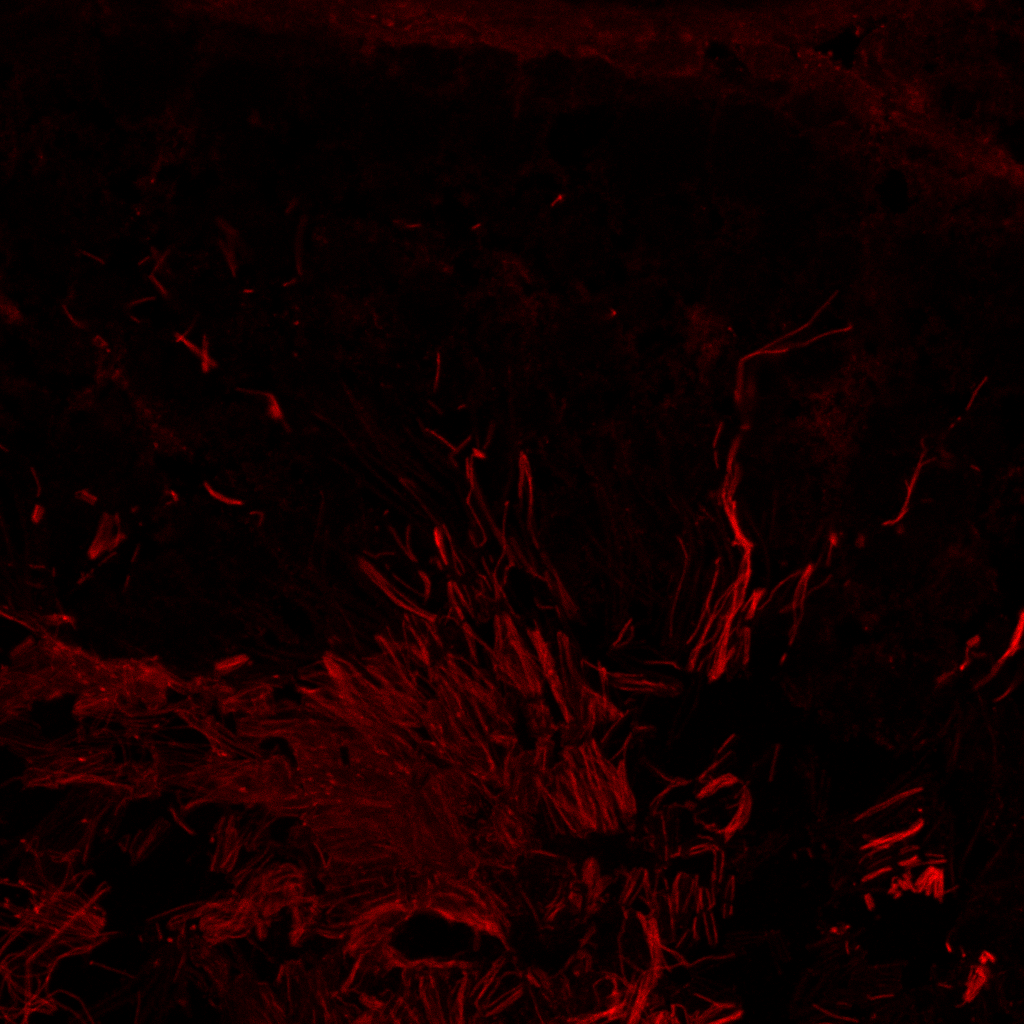

Supplement: Supplementary file 9 [file Data_Sheet_6.ZIP › Fig4B/cf wt tub .lif_Series045_z0_ch02.tif]

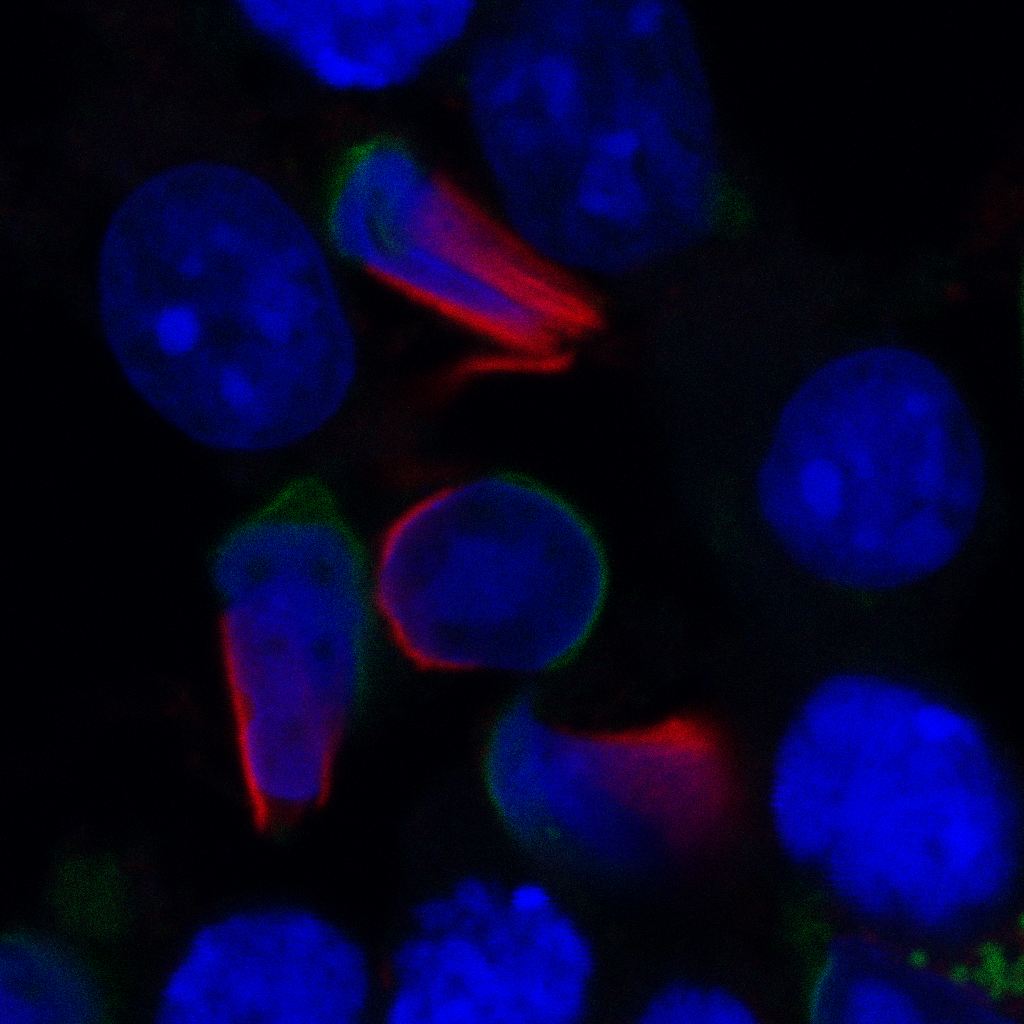

Supplement: Supplementary file 10 [file Data_Sheet_7.ZIP › Fig4D/KO/cfap53 ko.lif_11-12_z0.tif]

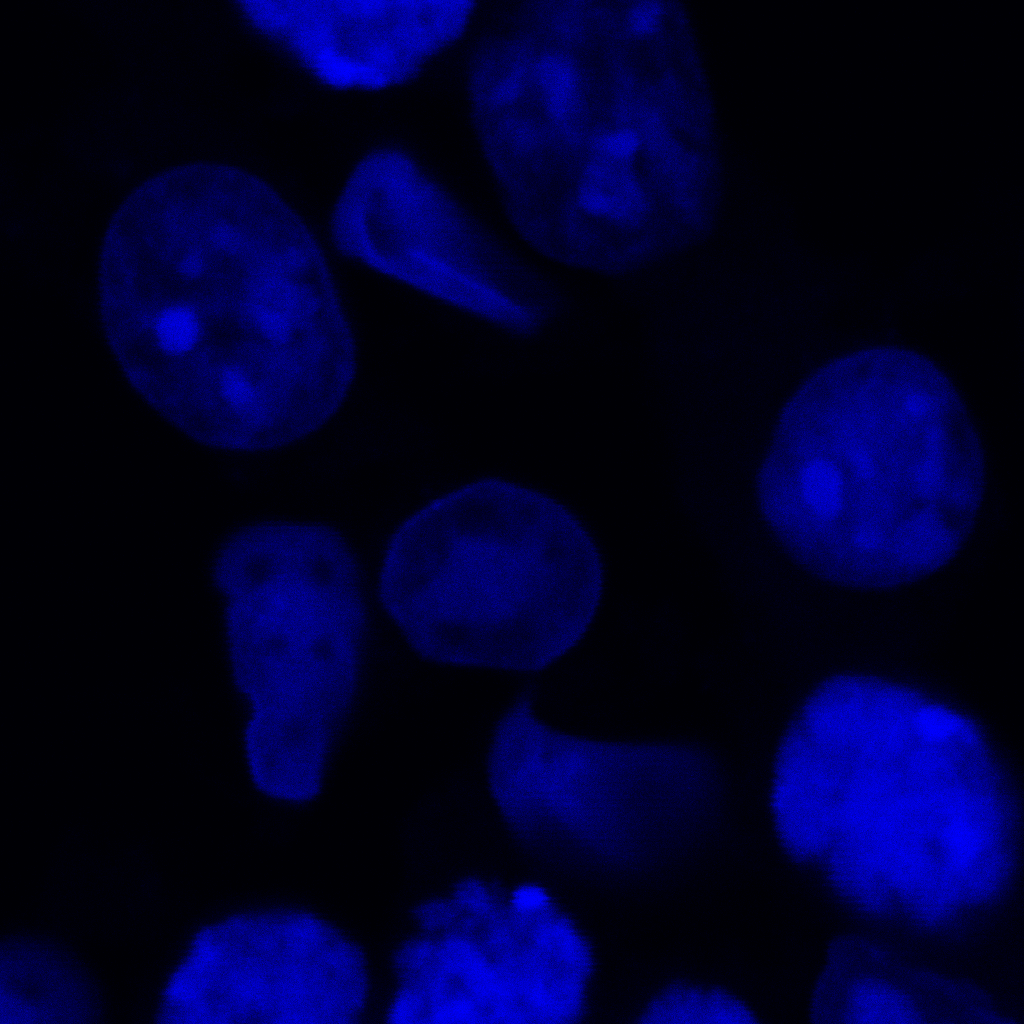

Supplement: Supplementary file 10 [file Data_Sheet_7.ZIP › Fig4D/KO/cfap53 ko.lif_11-12_z0_ch00.tif]

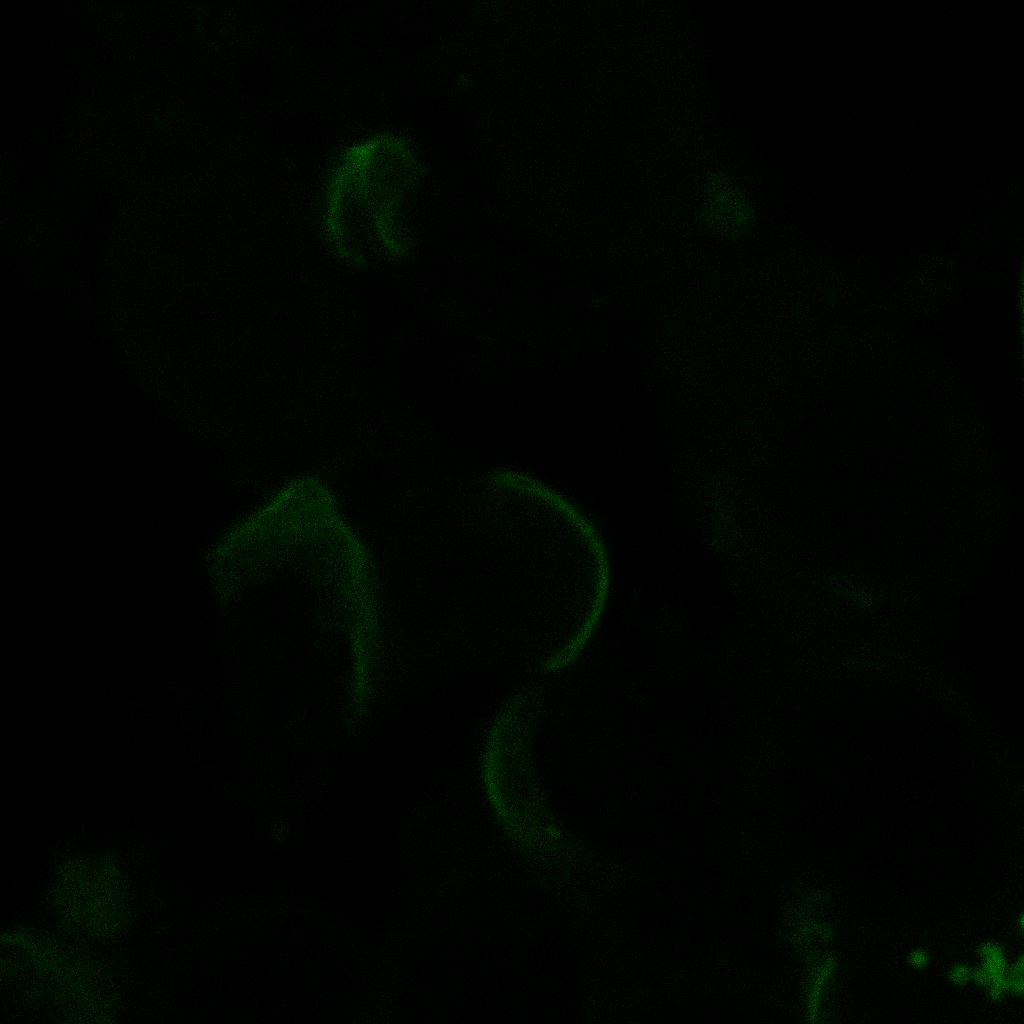

Supplement: Supplementary file 10 [file Data_Sheet_7.ZIP › Fig4D/KO/cfap53 ko.lif_11-12_z0_ch01.tif]

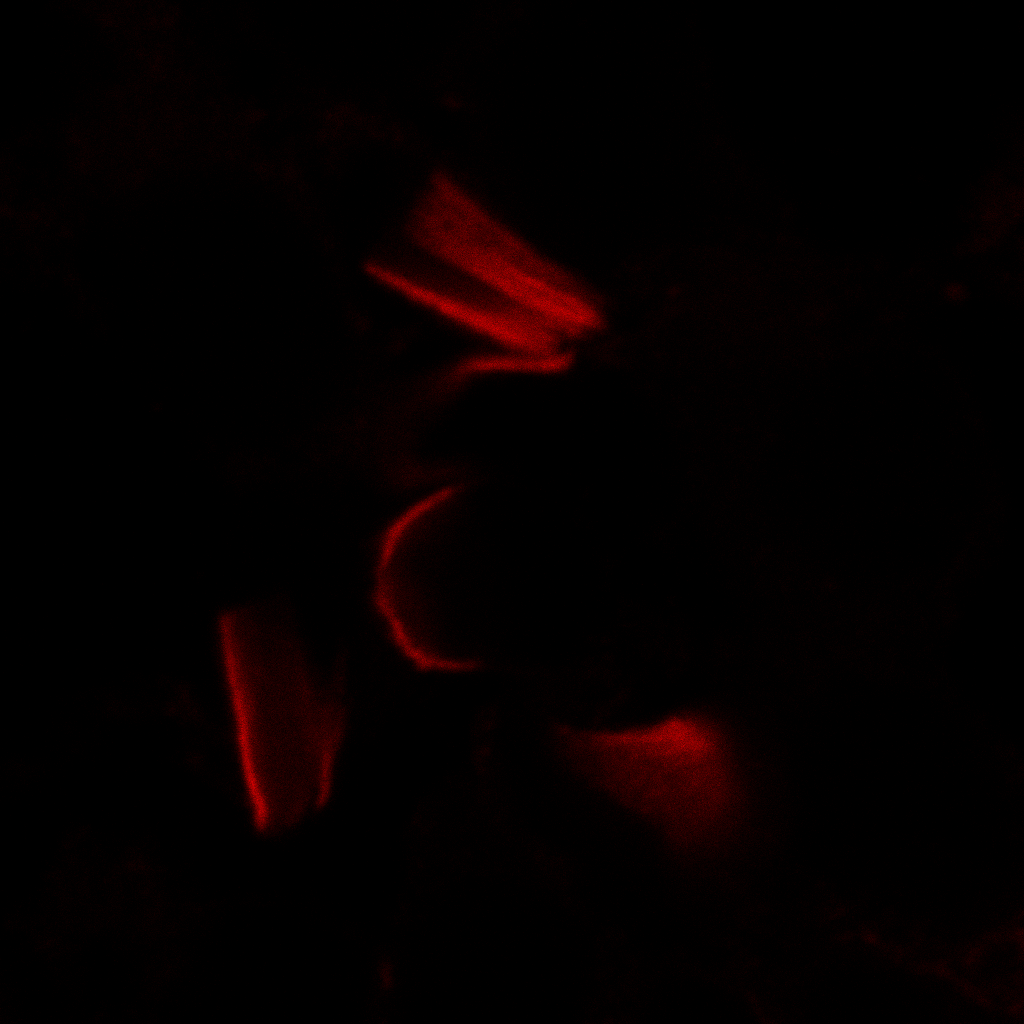

Supplement: Supplementary file 10 [file Data_Sheet_7.ZIP › Fig4D/KO/cfap53 ko.lif_11-12_z0_ch02.tif]

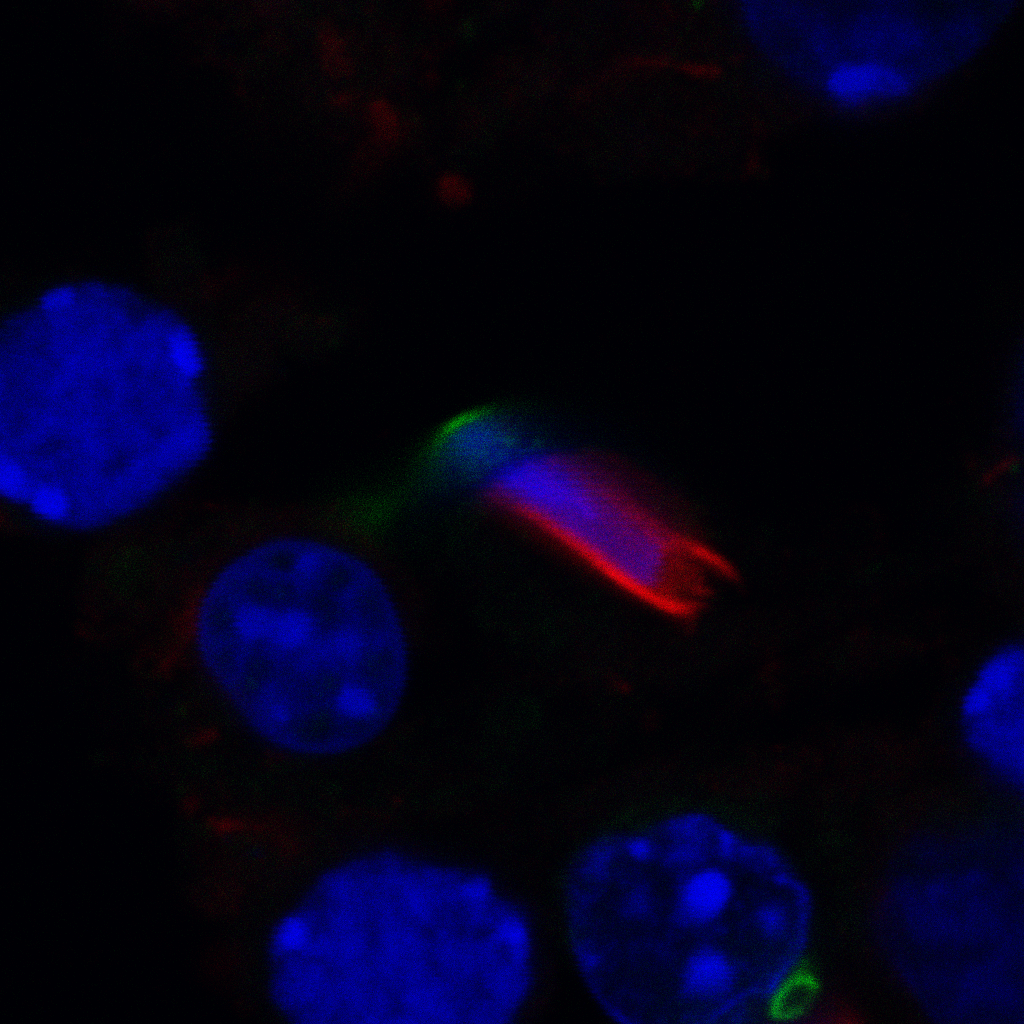

Supplement: Supplementary file 10 [file Data_Sheet_7.ZIP › Fig4D/KO/cfap53 ko.lif_12-13_z0.tif]

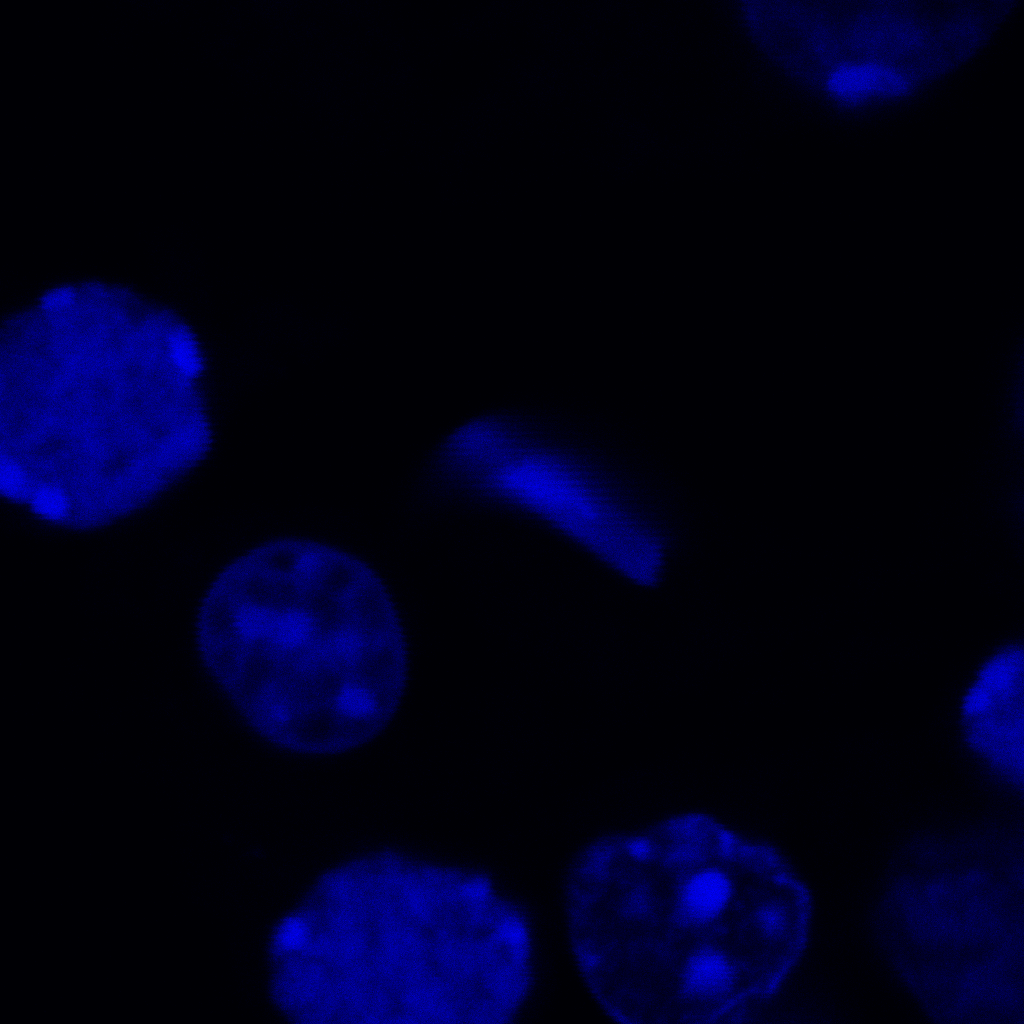

Supplement: Supplementary file 10 [file Data_Sheet_7.ZIP › Fig4D/KO/cfap53 ko.lif_12-13_z0_ch00.tif]

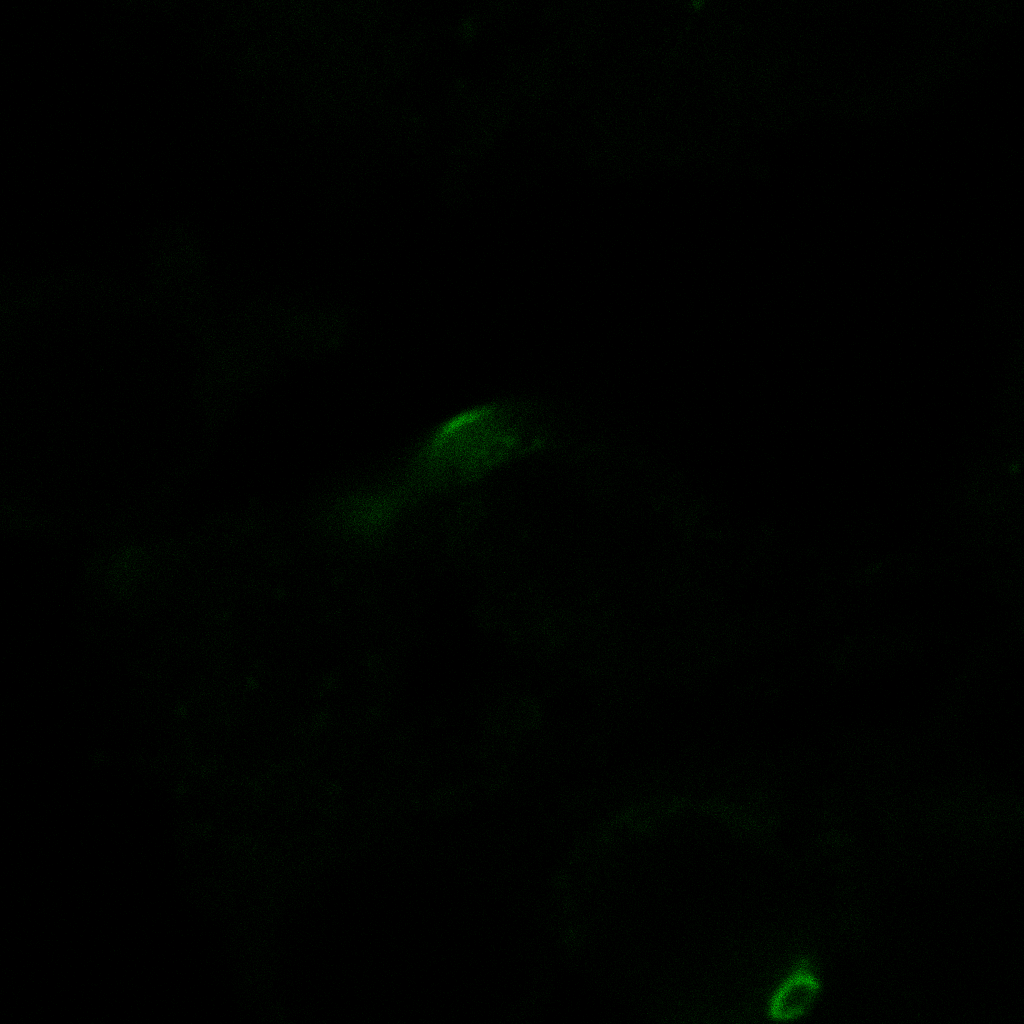

Supplement: Supplementary file 10 [file Data_Sheet_7.ZIP › Fig4D/KO/cfap53 ko.lif_12-13_z0_ch01.tif]

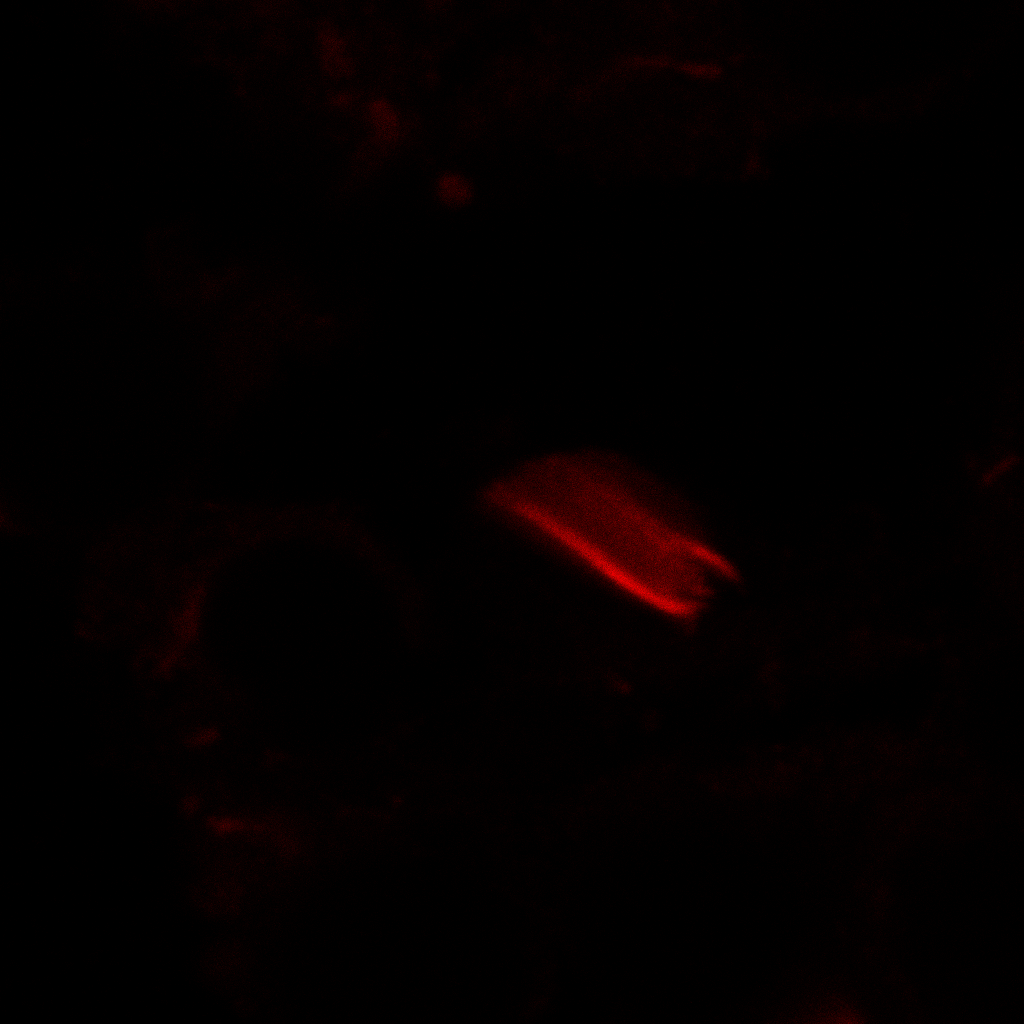

Supplement: Supplementary file 10 [file Data_Sheet_7.ZIP › Fig4D/KO/cfap53 ko.lif_12-13_z0_ch02.tif]

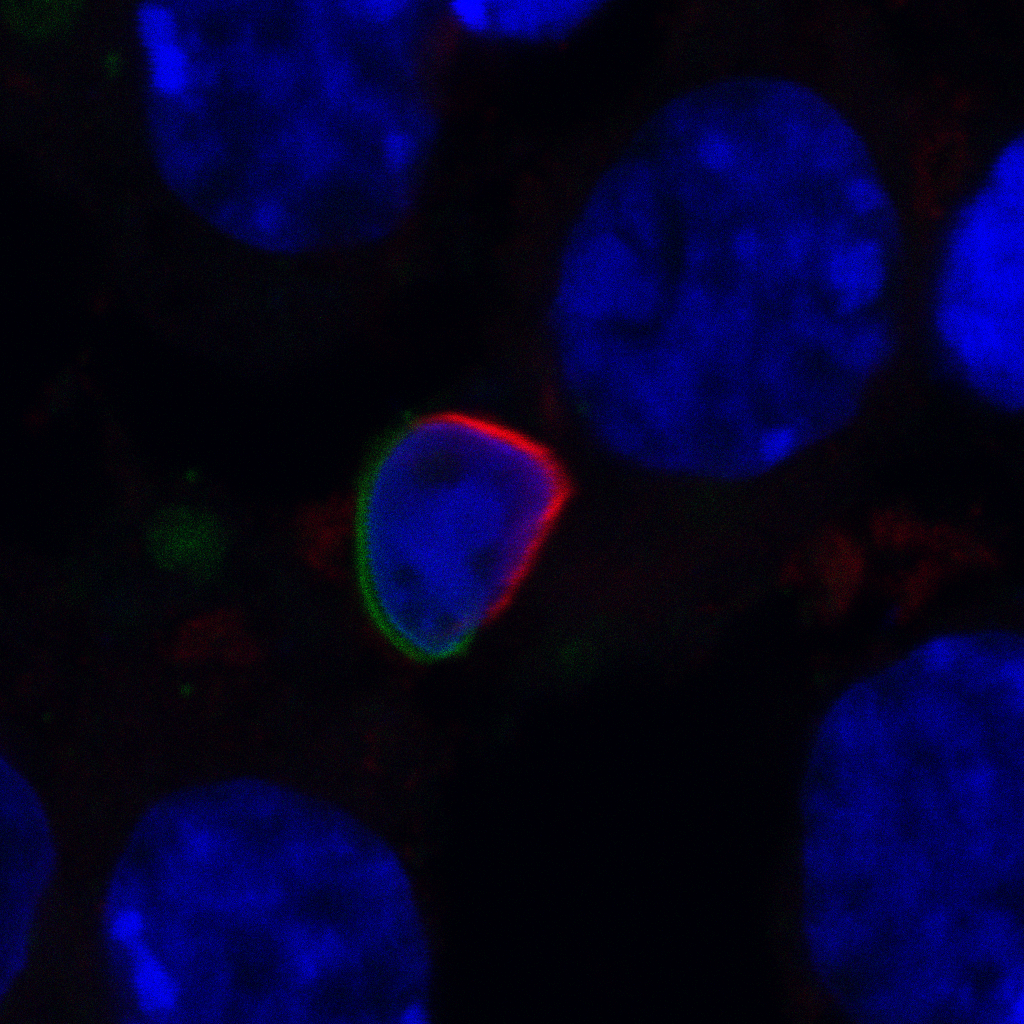

Supplement: Supplementary file 10 [file Data_Sheet_7.ZIP › Fig4D/KO/cfap53 ko.lif_8-9_z0.tif]

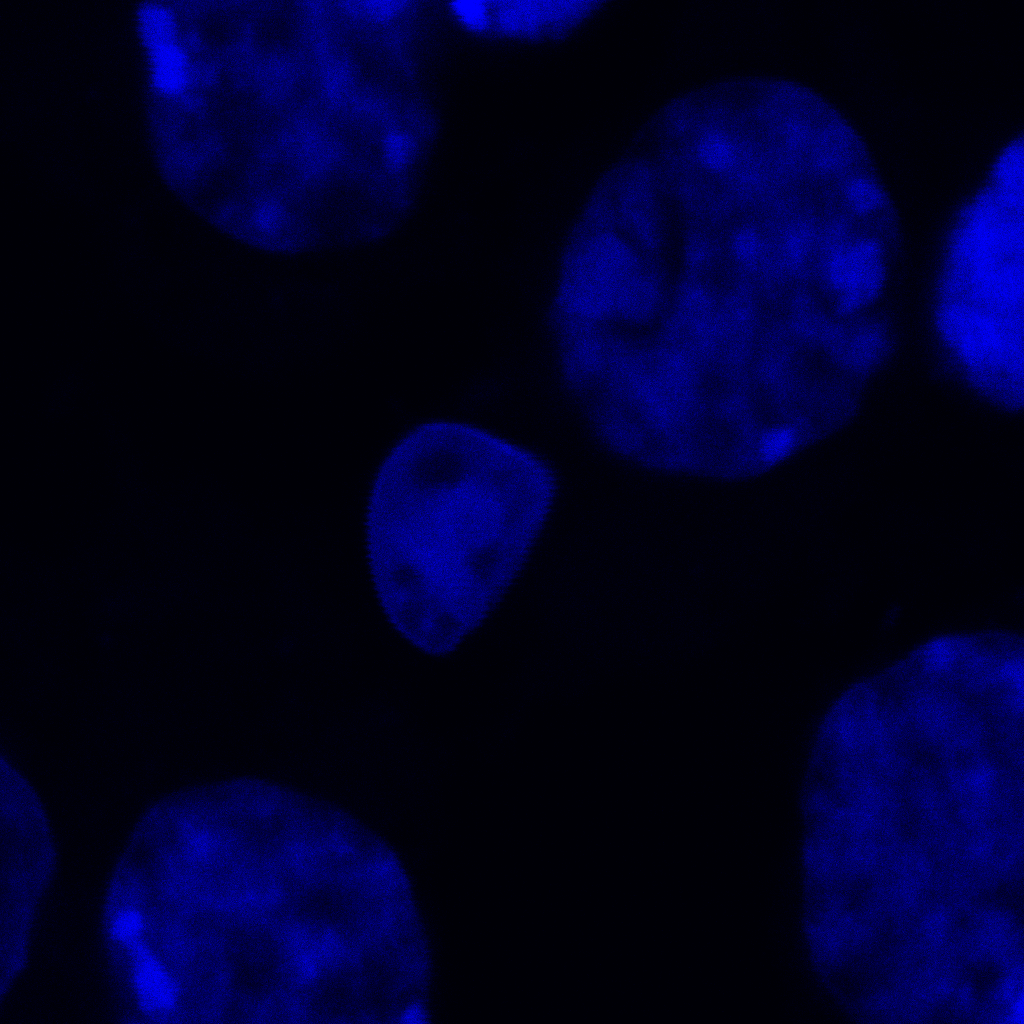

Supplement: Supplementary file 10 [file Data_Sheet_7.ZIP › Fig4D/KO/cfap53 ko.lif_8-9_z0_ch00.tif]

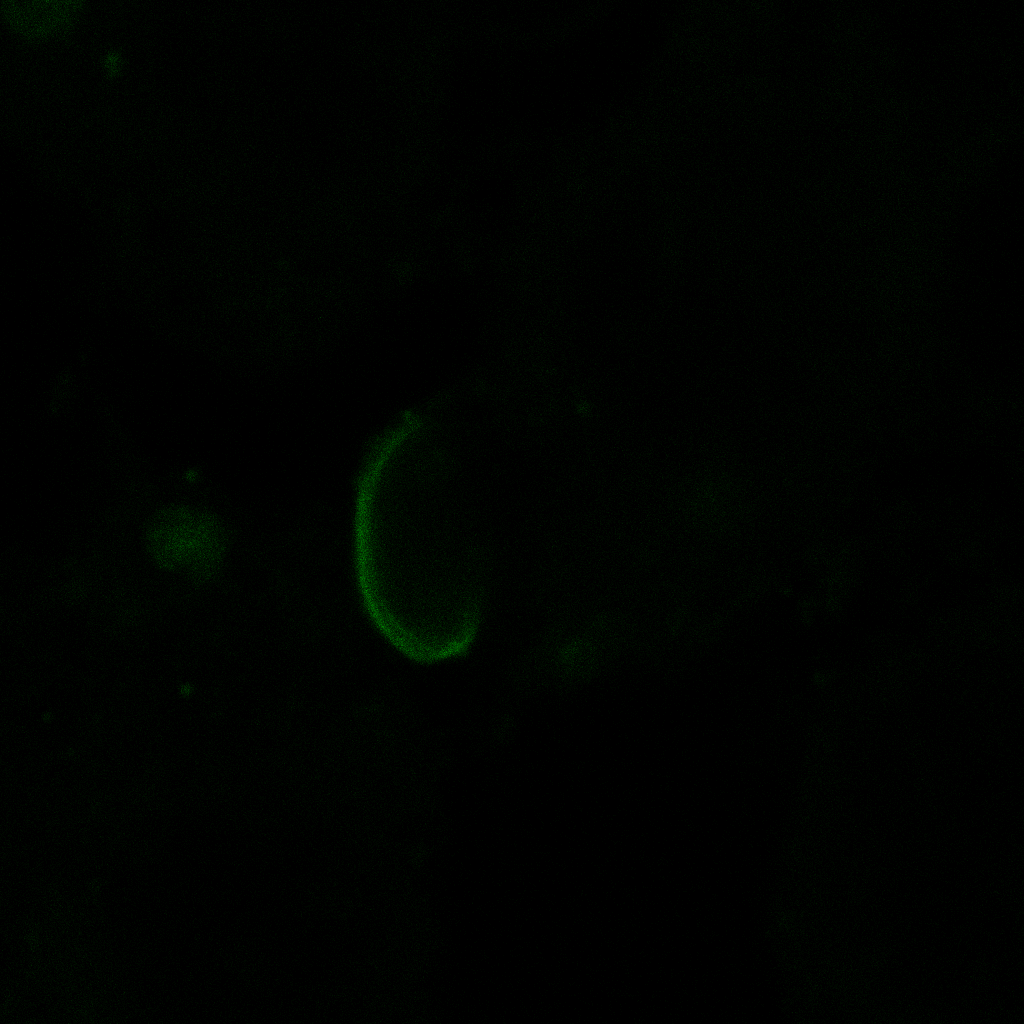

Supplement: Supplementary file 10 [file Data_Sheet_7.ZIP › Fig4D/KO/cfap53 ko.lif_8-9_z0_ch01.tif]

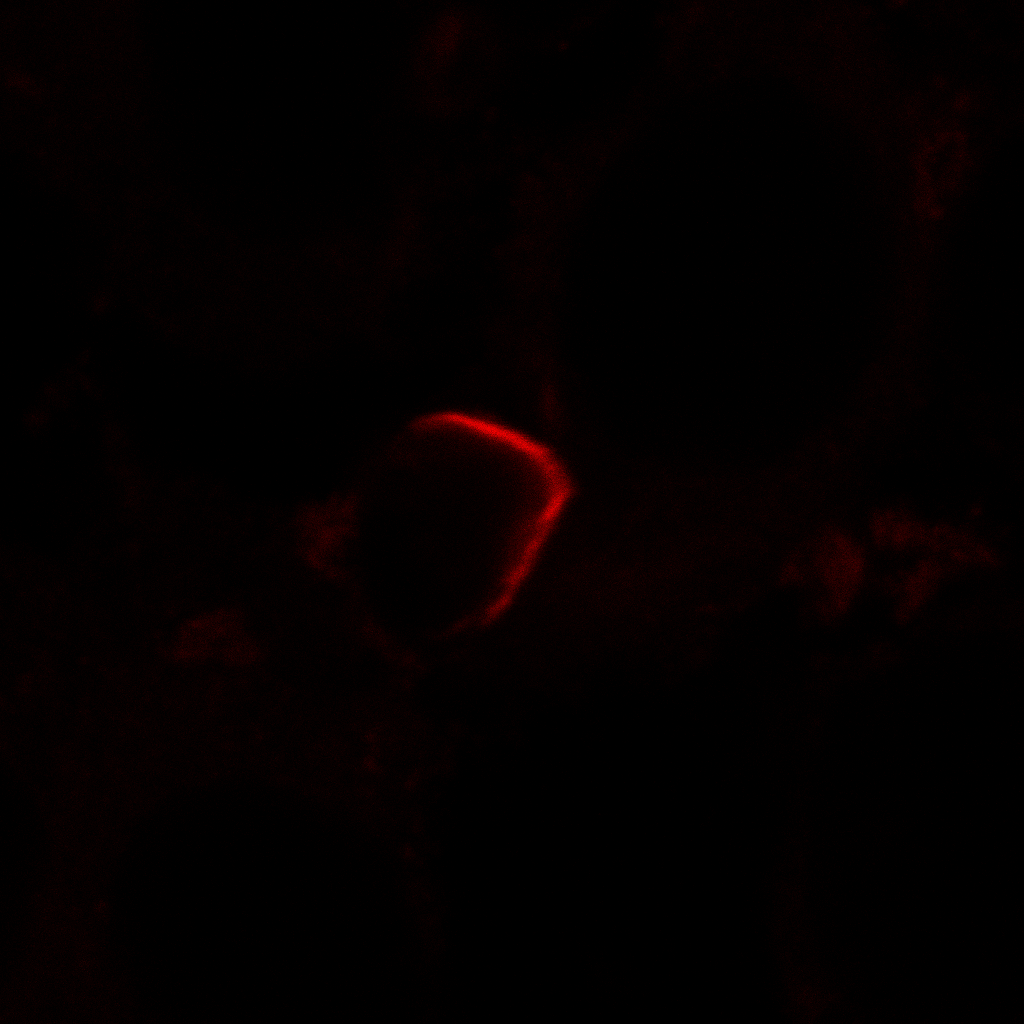

Supplement: Supplementary file 10 [file Data_Sheet_7.ZIP › Fig4D/KO/cfap53 ko.lif_8-9_z0_ch02.tif]

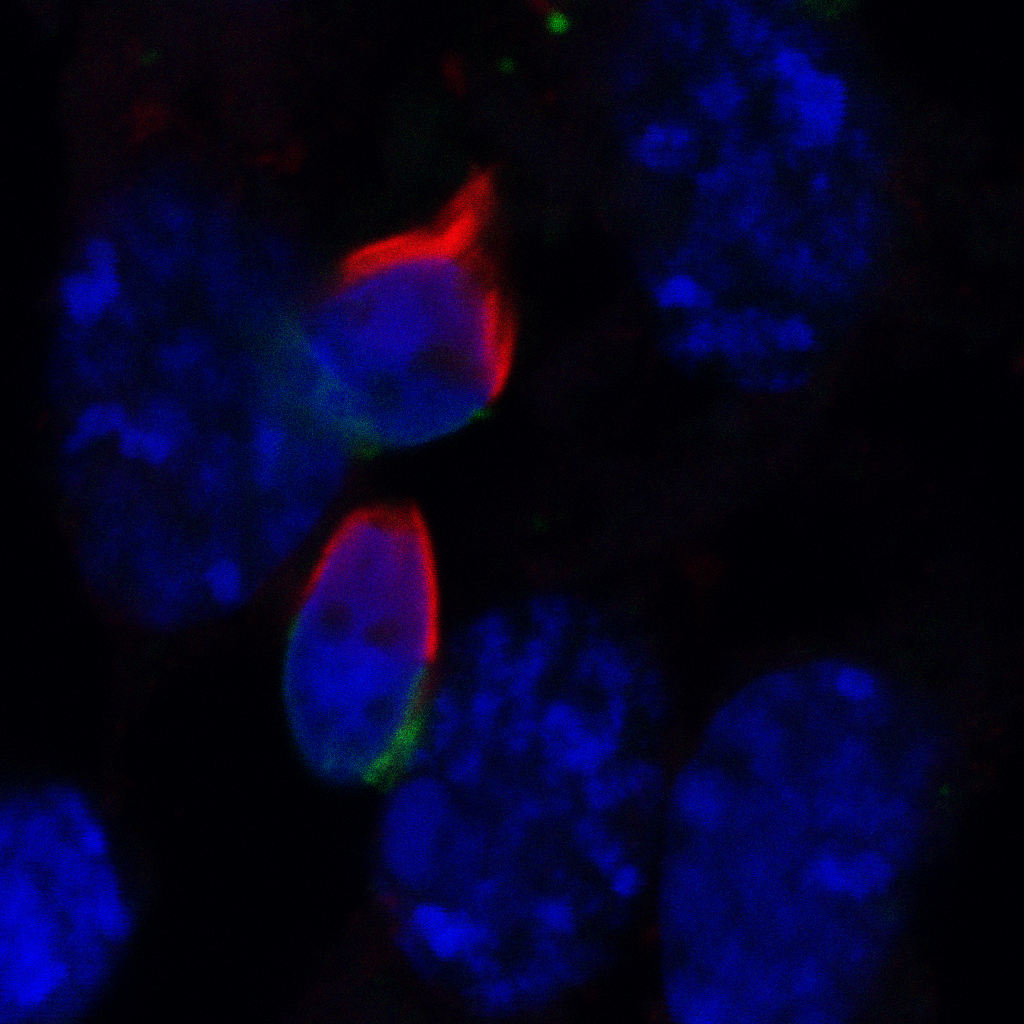

Supplement: Supplementary file 10 [file Data_Sheet_7.ZIP › Fig4D/KO/cfap53 ko.lif_9-10_z0.tif]

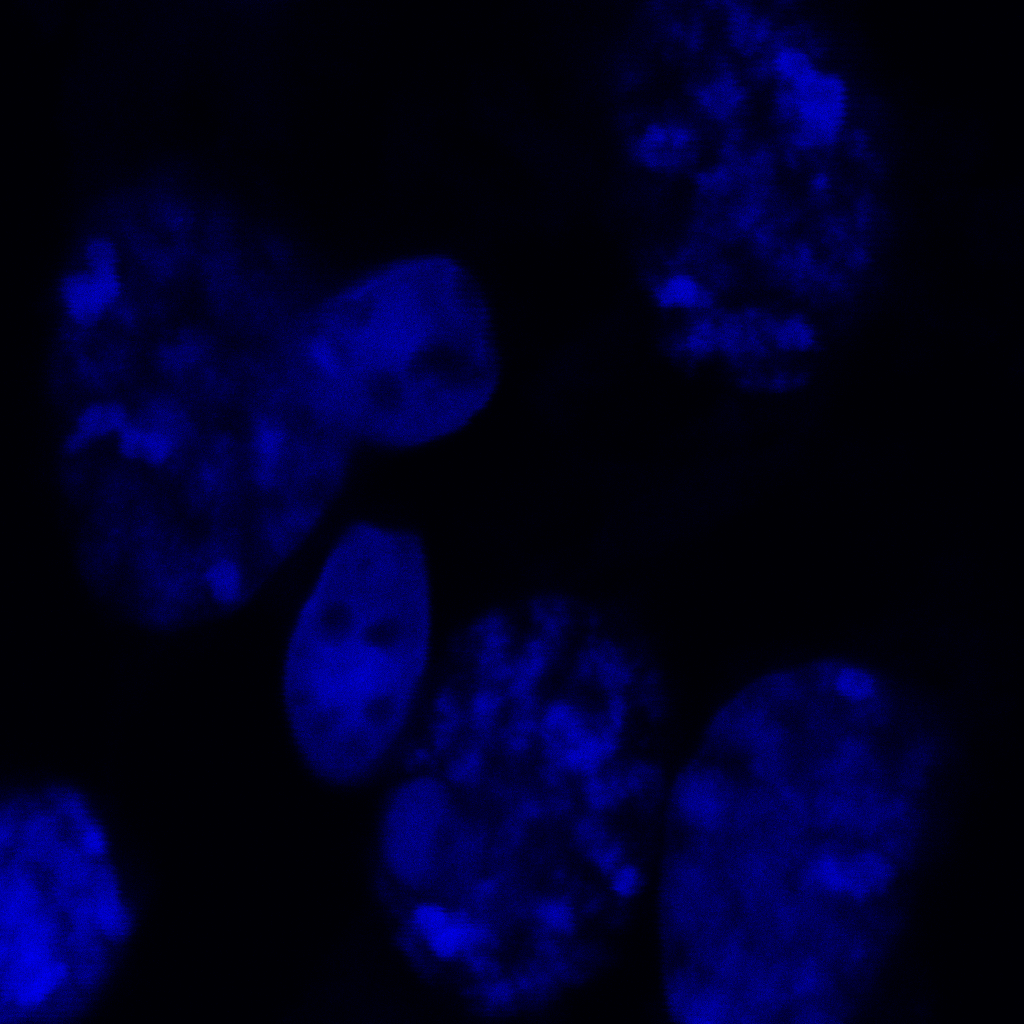

Supplement: Supplementary file 10 [file Data_Sheet_7.ZIP › Fig4D/KO/cfap53 ko.lif_9-10_z0_ch00.tif]

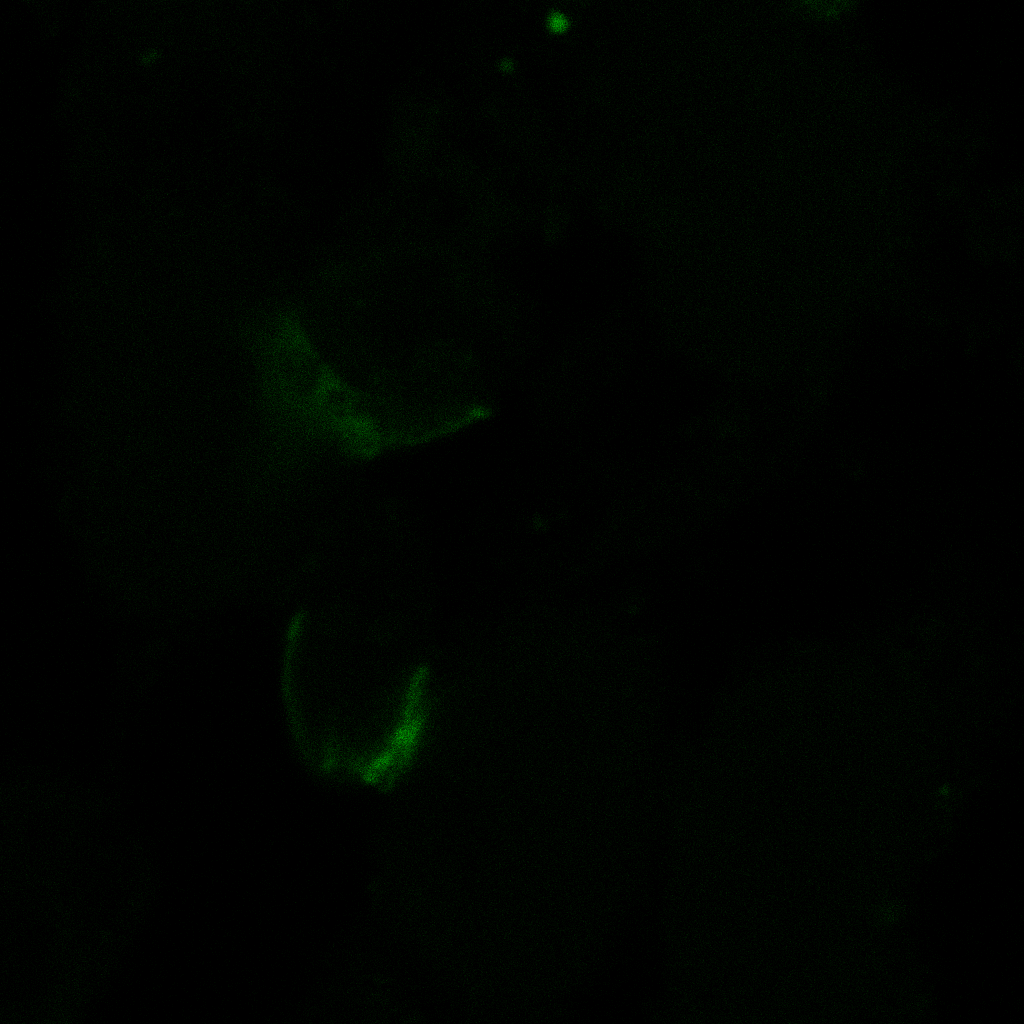

Supplement: Supplementary file 10 [file Data_Sheet_7.ZIP › Fig4D/KO/cfap53 ko.lif_9-10_z0_ch01.tif]

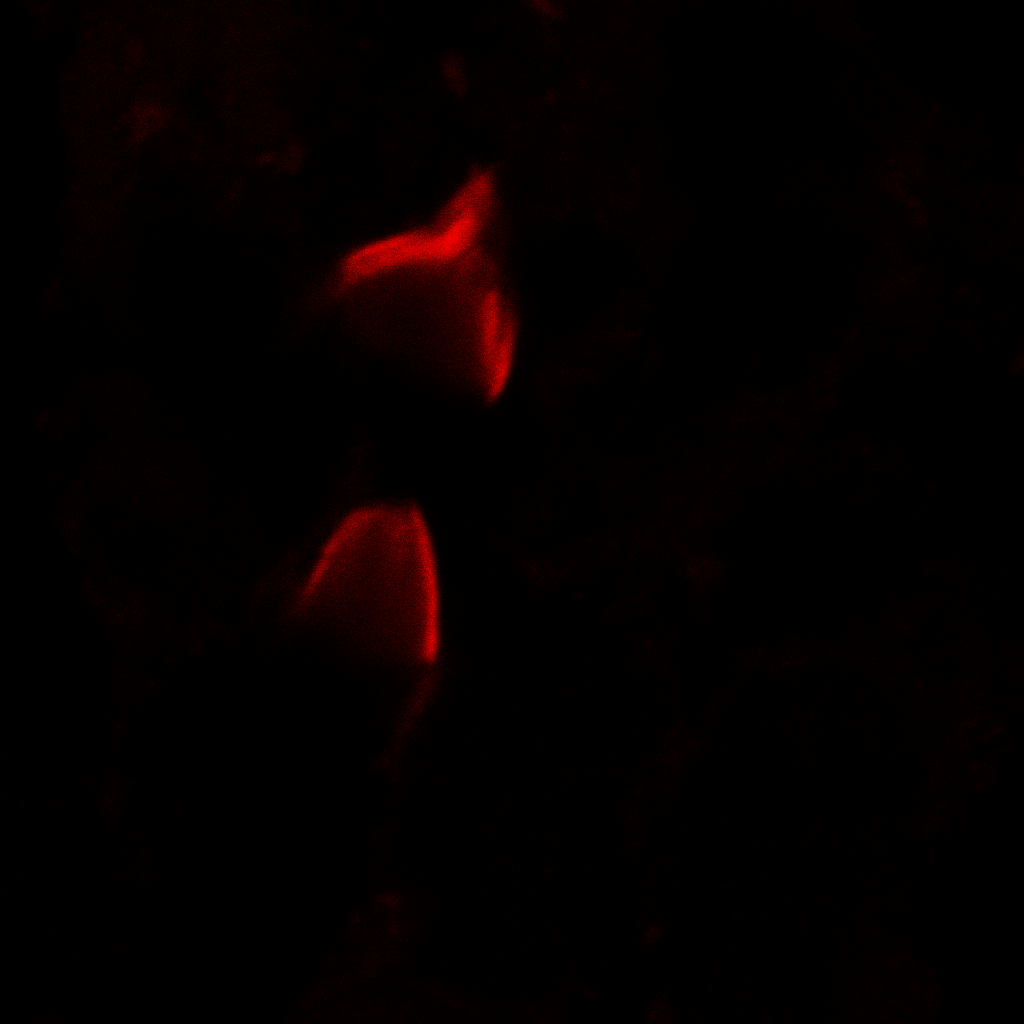

Supplement: Supplementary file 10 [file Data_Sheet_7.ZIP › Fig4D/KO/cfap53 ko.lif_9-10_z0_ch02.tif]

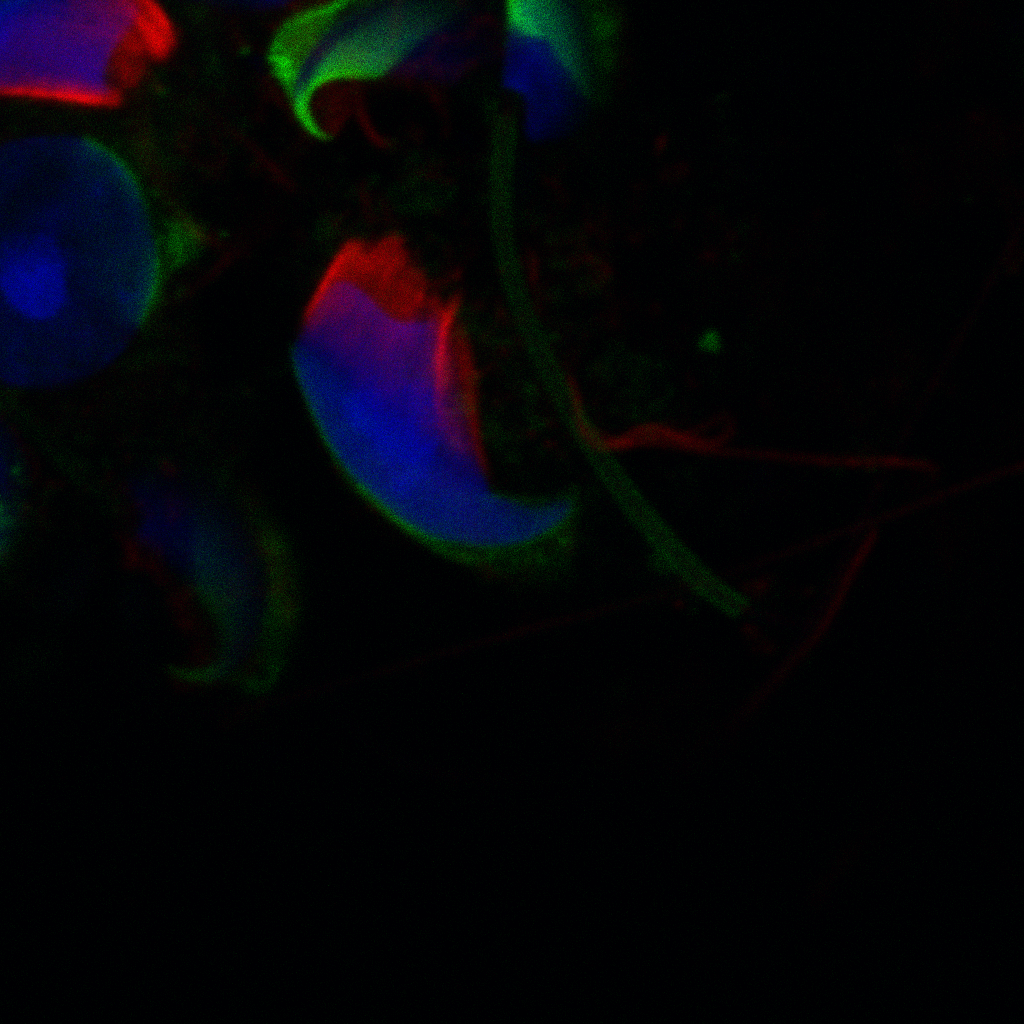

Supplement: Supplementary file 10 [file Data_Sheet_7.ZIP › Fig4D/WT/tub wt.lif_11-12 3_z0.tif]

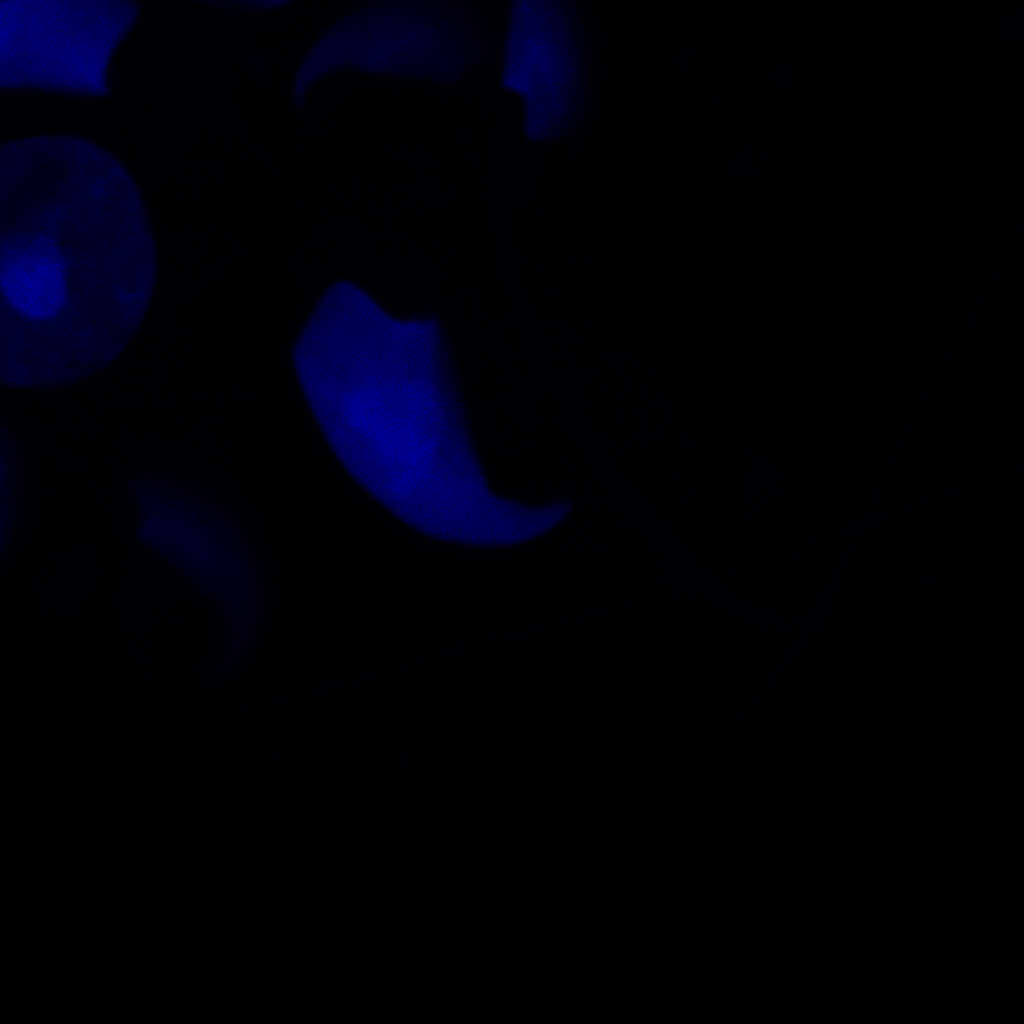

Supplement: Supplementary file 10 [file Data_Sheet_7.ZIP › Fig4D/WT/tub wt.lif_11-12 3_z0_ch00.tif]

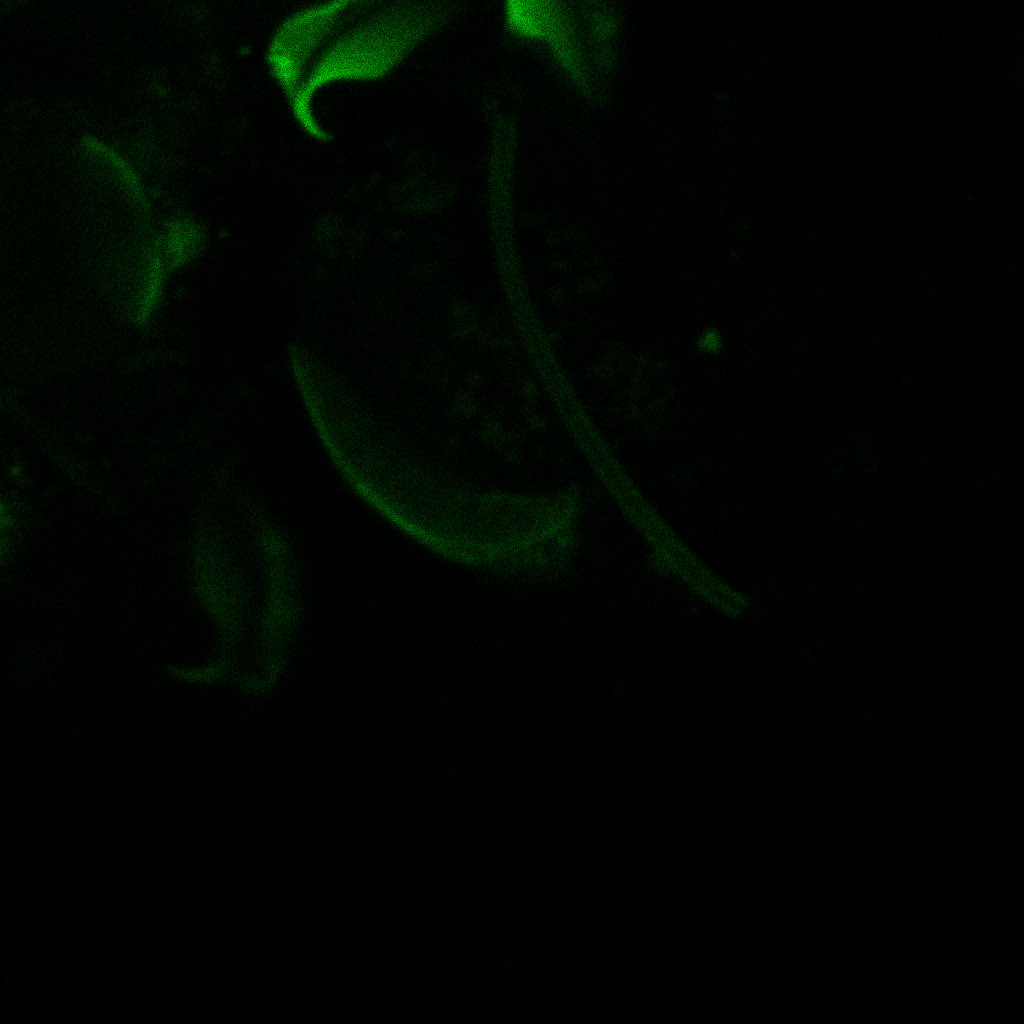

Supplement: Supplementary file 10 [file Data_Sheet_7.ZIP › Fig4D/WT/tub wt.lif_11-12 3_z0_ch01.tif]

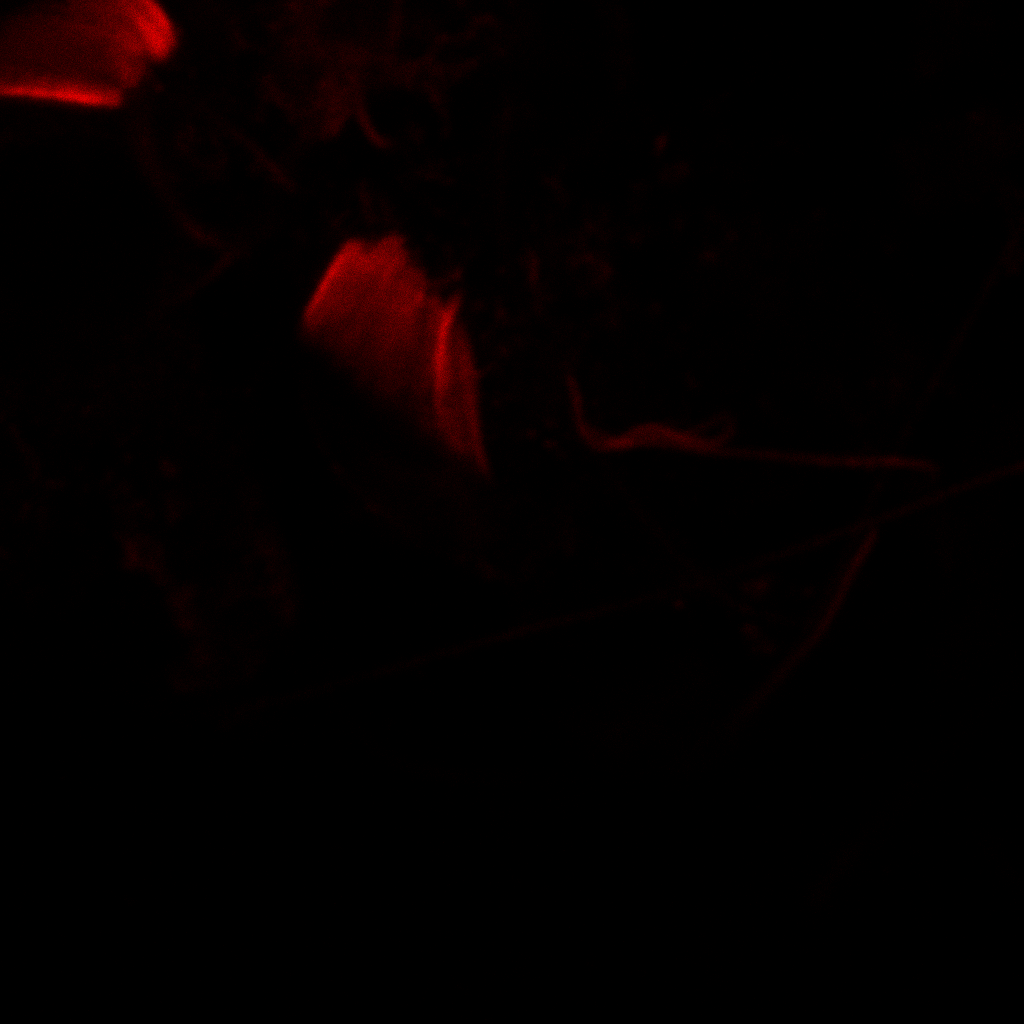

Supplement: Supplementary file 10 [file Data_Sheet_7.ZIP › Fig4D/WT/tub wt.lif_11-12 3_z0_ch02.tif]

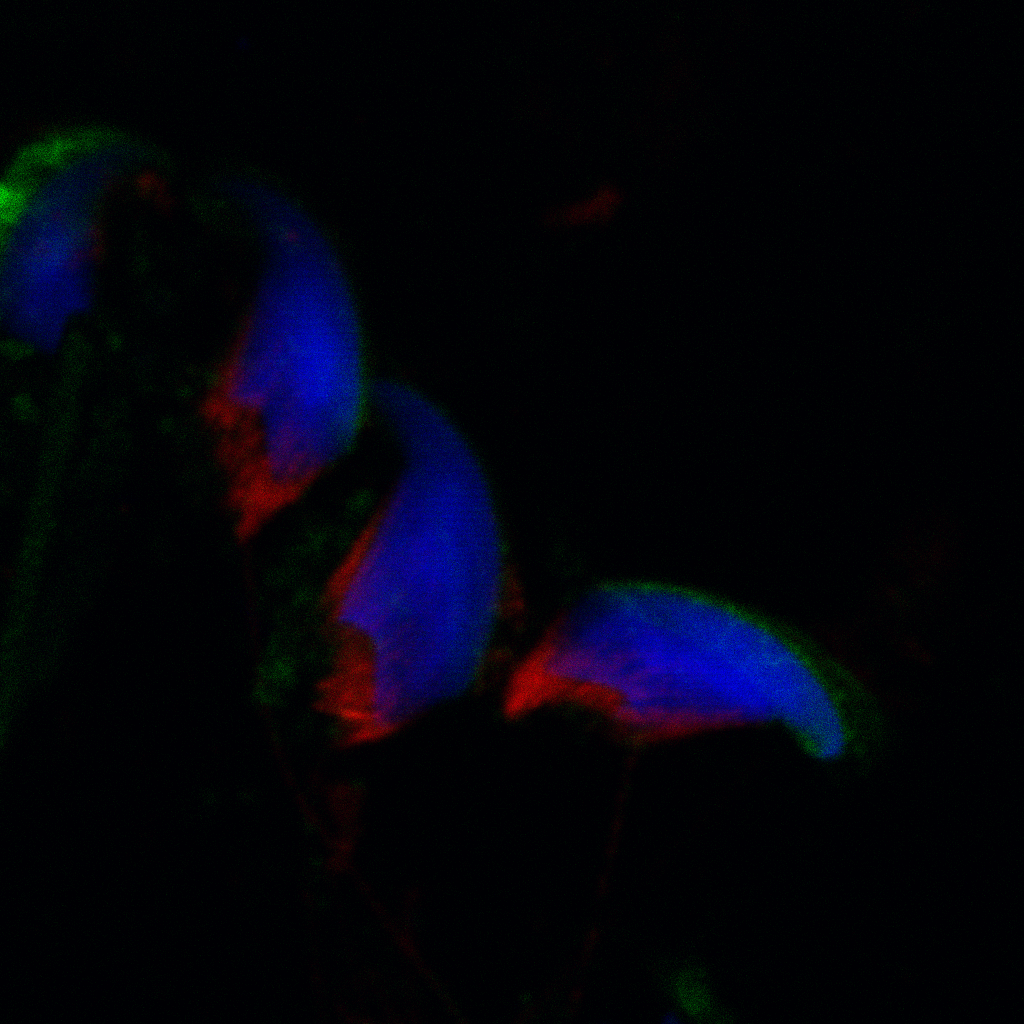

Supplement: Supplementary file 10 [file Data_Sheet_7.ZIP › Fig4D/WT/tub wt.lif_12-13_z0.tif]

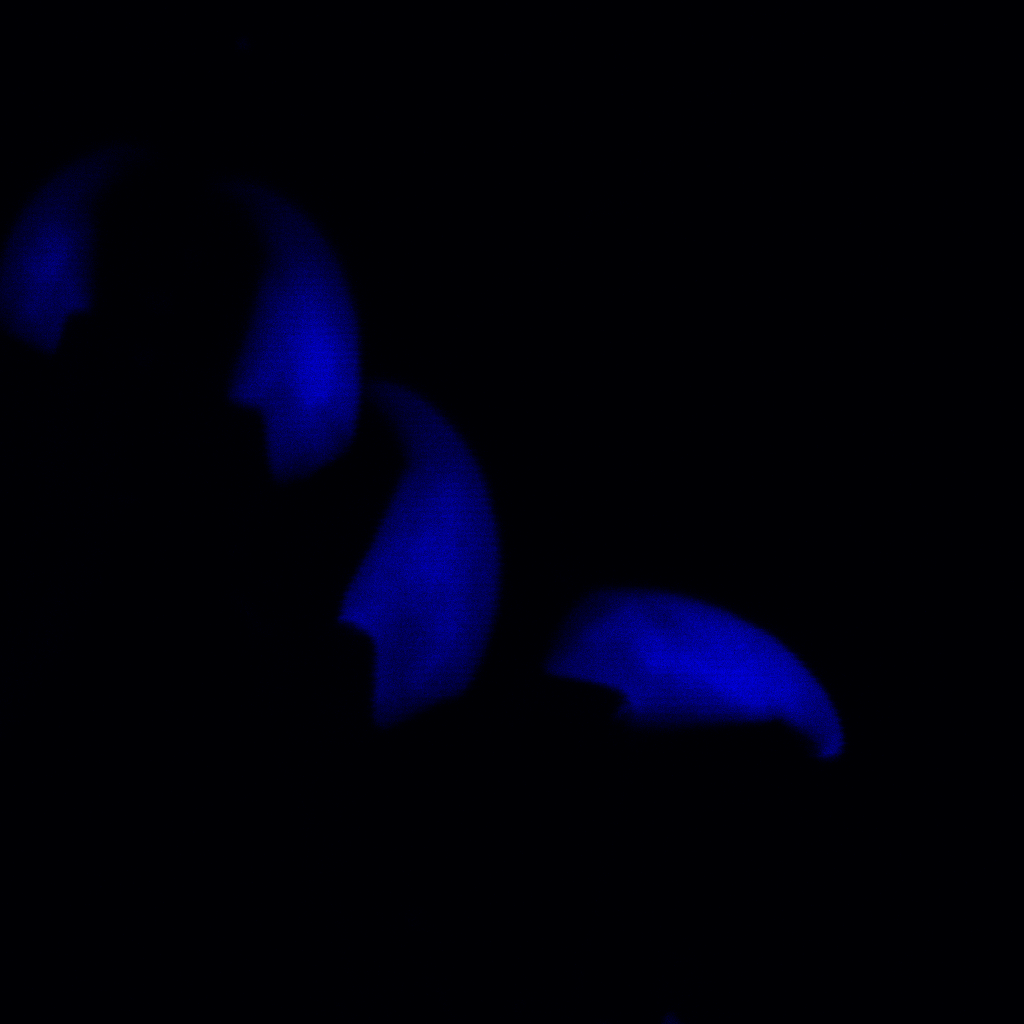

Supplement: Supplementary file 10 [file Data_Sheet_7.ZIP › Fig4D/WT/tub wt.lif_12-13_z0_ch00.tif]

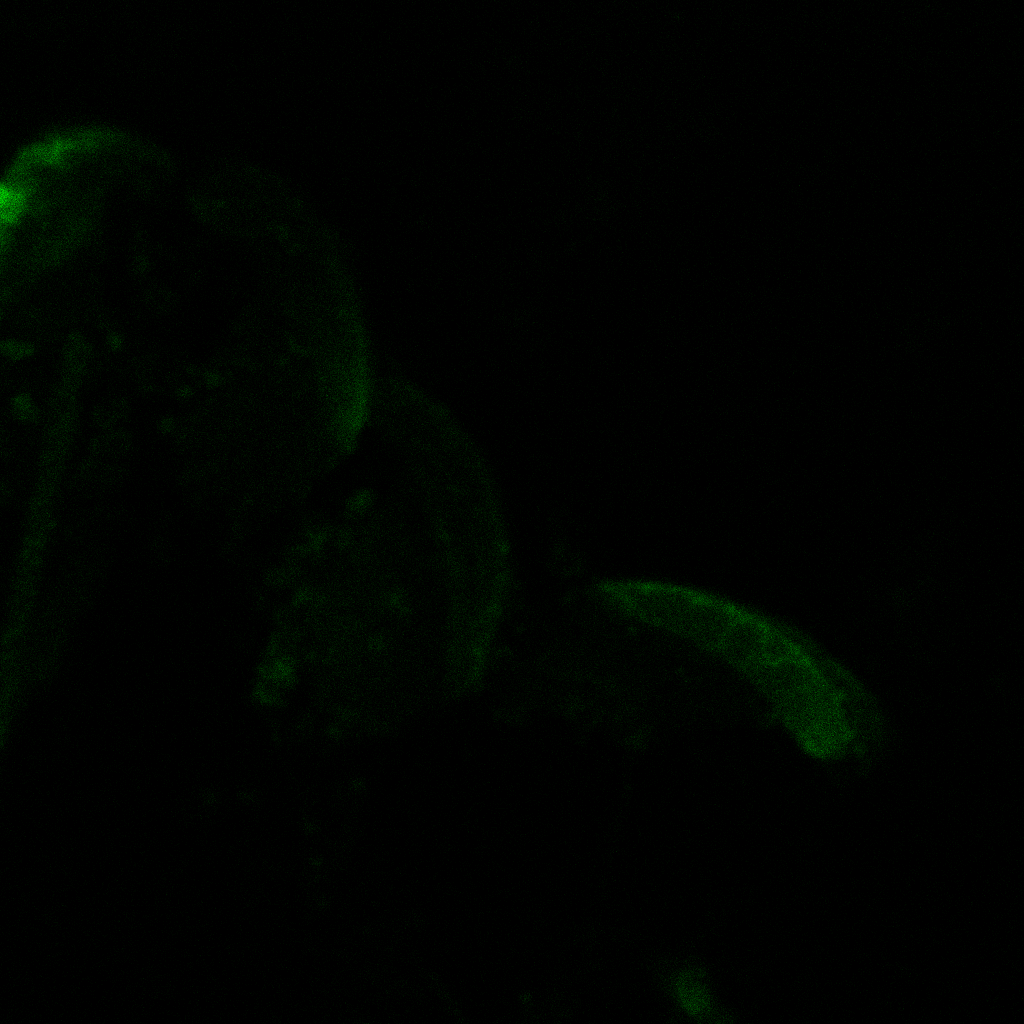

Supplement: Supplementary file 10 [file Data_Sheet_7.ZIP › Fig4D/WT/tub wt.lif_12-13_z0_ch01.tif]

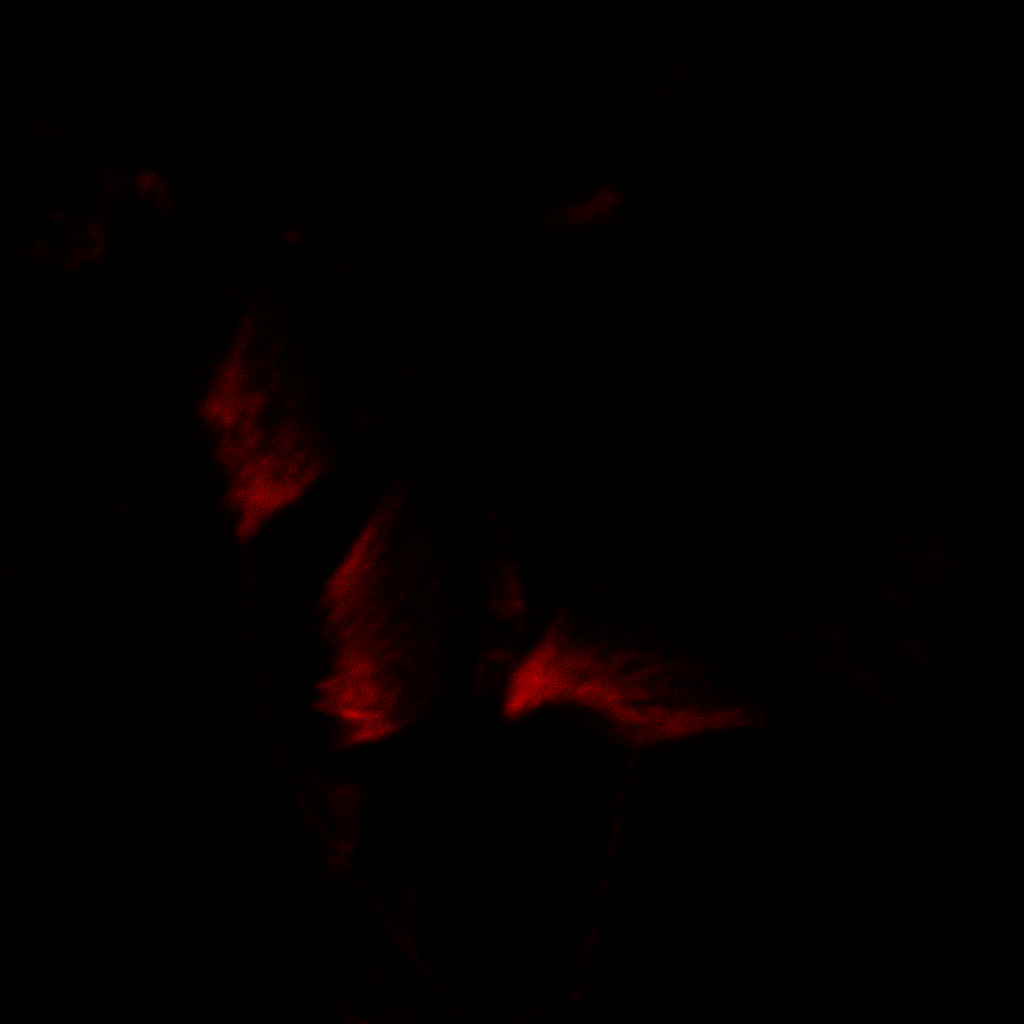

Supplement: Supplementary file 10 [file Data_Sheet_7.ZIP › Fig4D/WT/tub wt.lif_12-13_z0_ch02.tif]

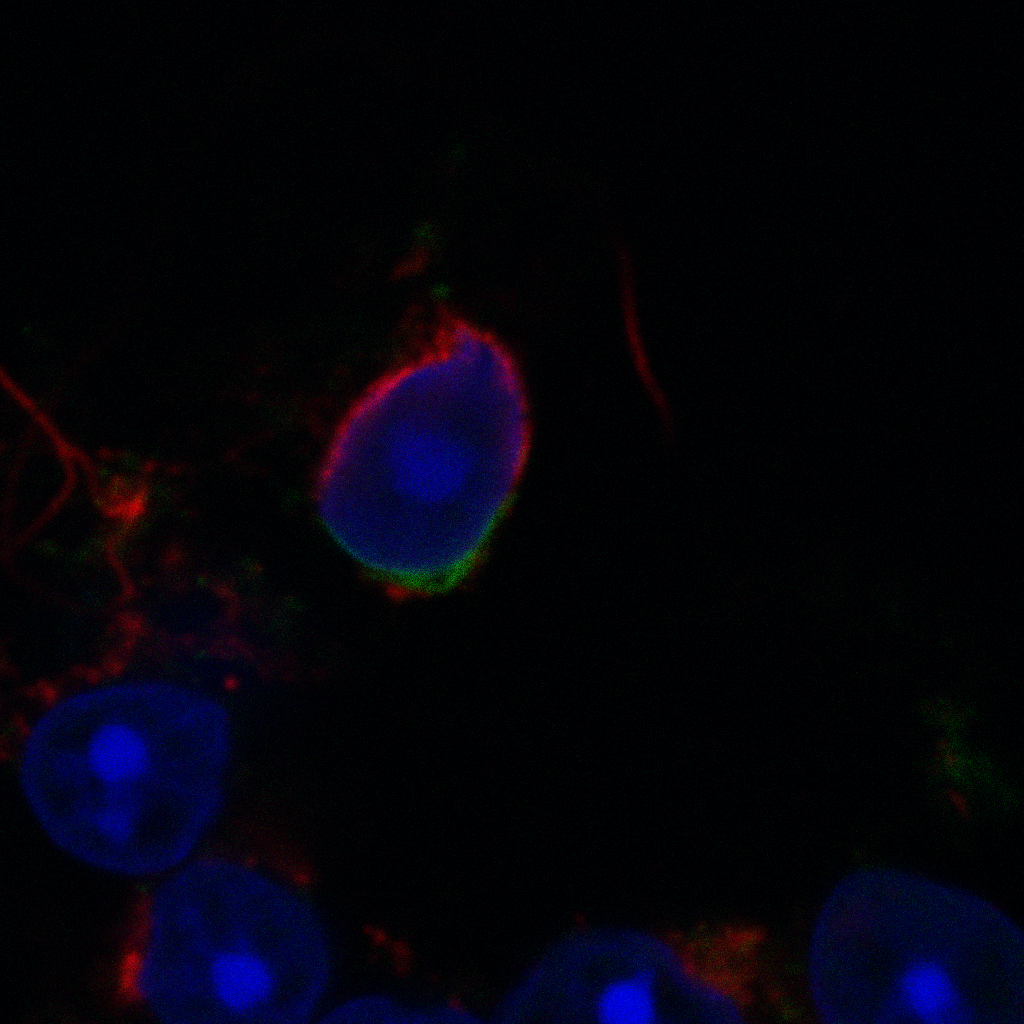

Supplement: Supplementary file 10 [file Data_Sheet_7.ZIP › Fig4D/WT/tub wt.lif_8-9_z0.tif]

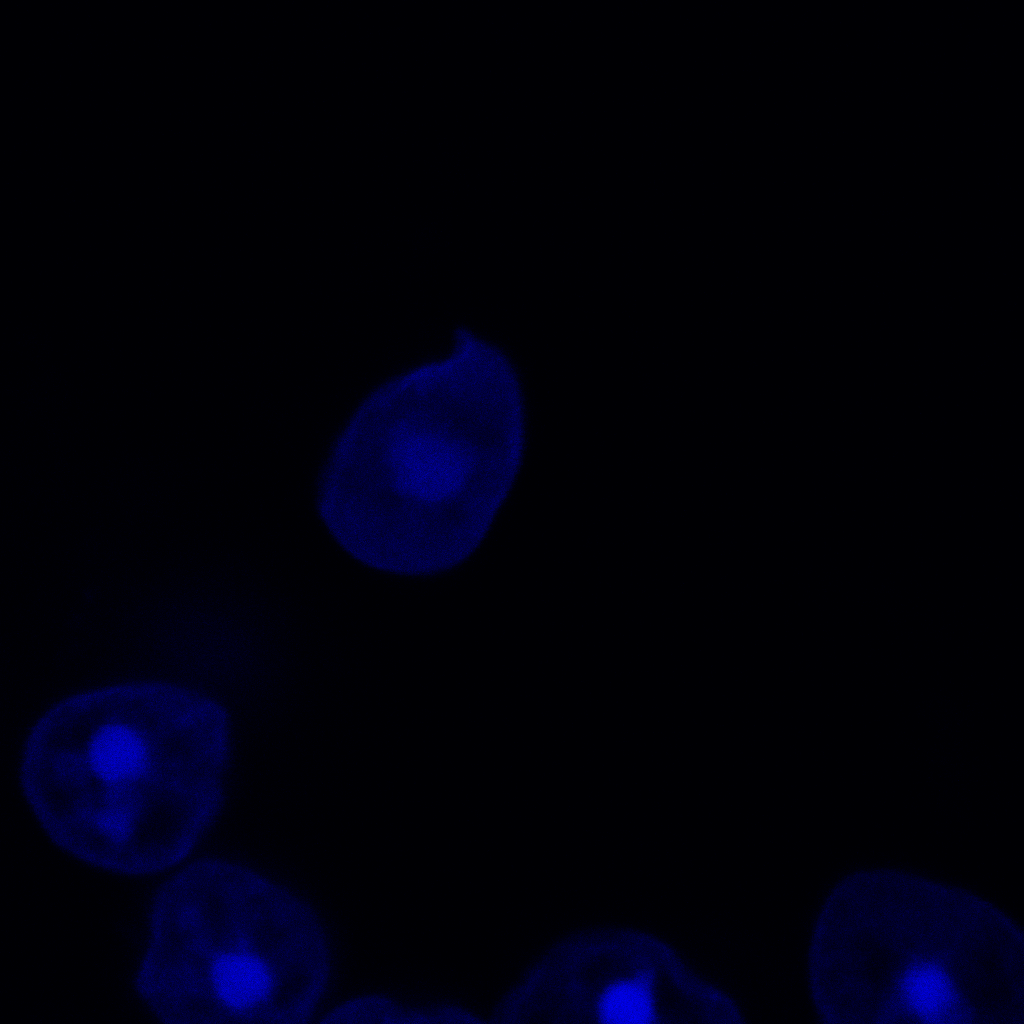

Supplement: Supplementary file 10 [file Data_Sheet_7.ZIP › Fig4D/WT/tub wt.lif_8-9_z0_ch00.tif]

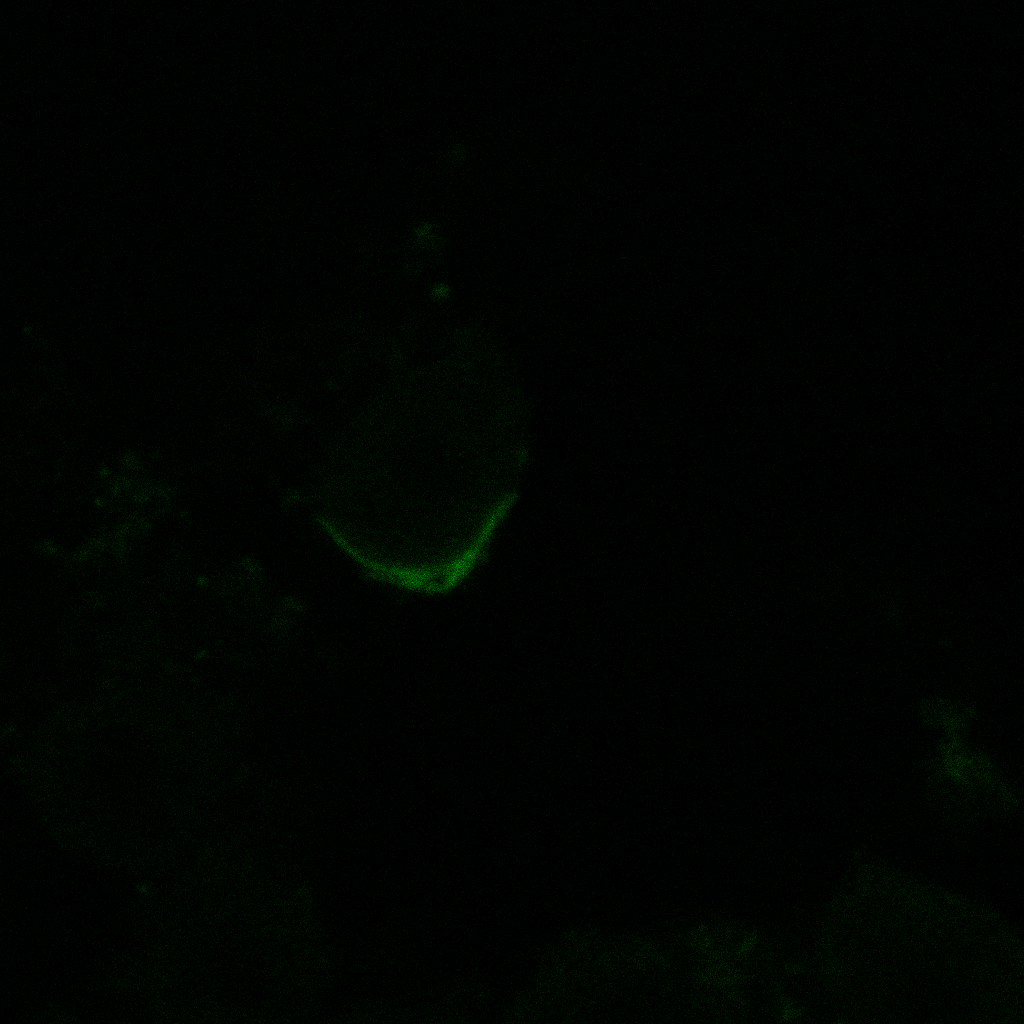

Supplement: Supplementary file 10 [file Data_Sheet_7.ZIP › Fig4D/WT/tub wt.lif_8-9_z0_ch01.tif]

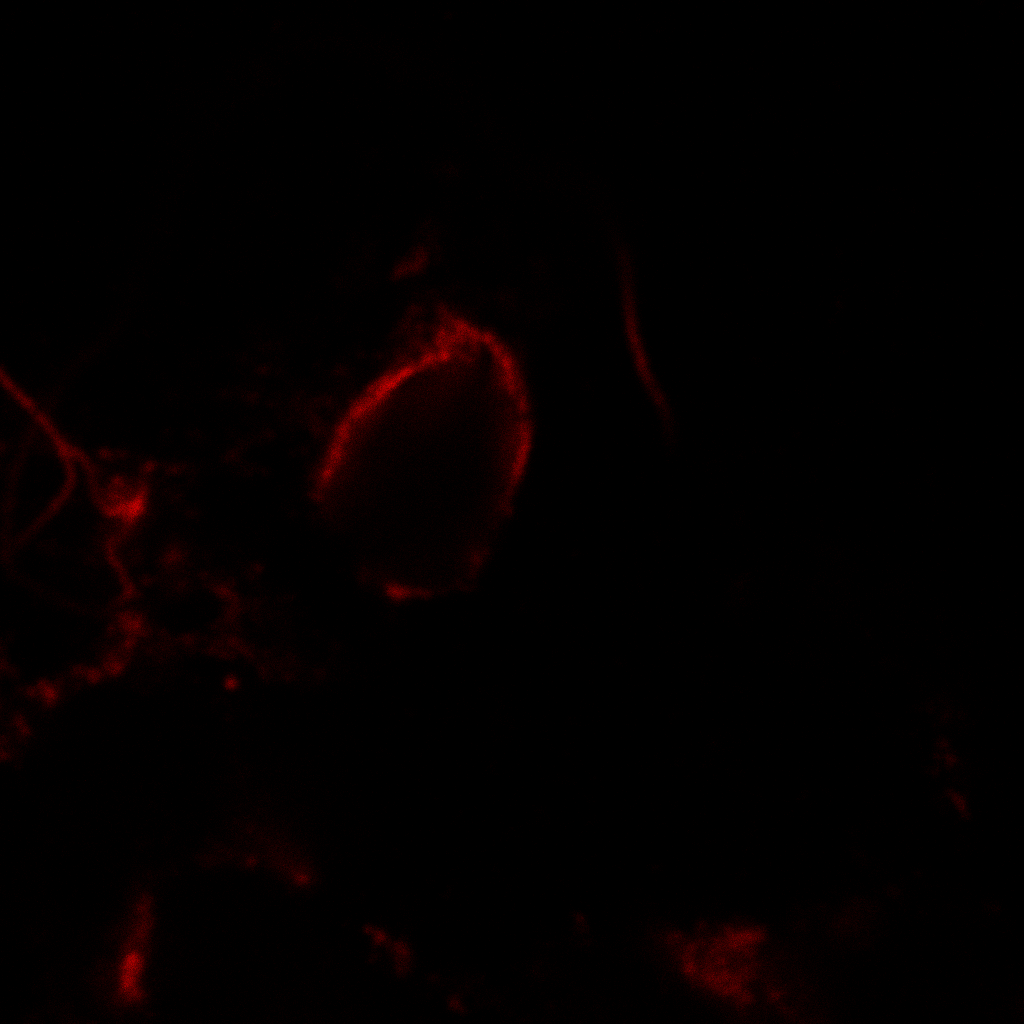

Supplement: Supplementary file 10 [file Data_Sheet_7.ZIP › Fig4D/WT/tub wt.lif_8-9_z0_ch02.tif]

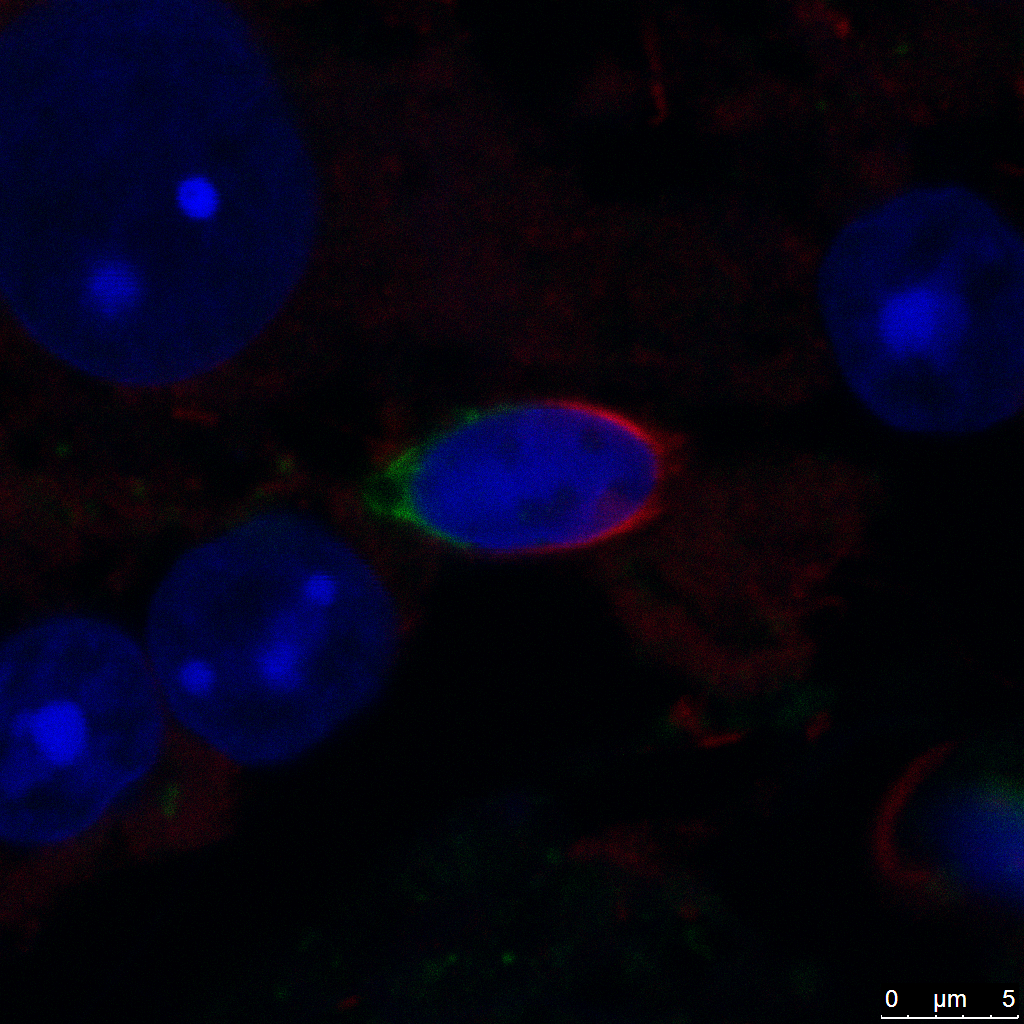

Supplement: Supplementary file 10 [file Data_Sheet_7.ZIP › Fig4D/WT/wt tub.lif_9-10_z0.tif]

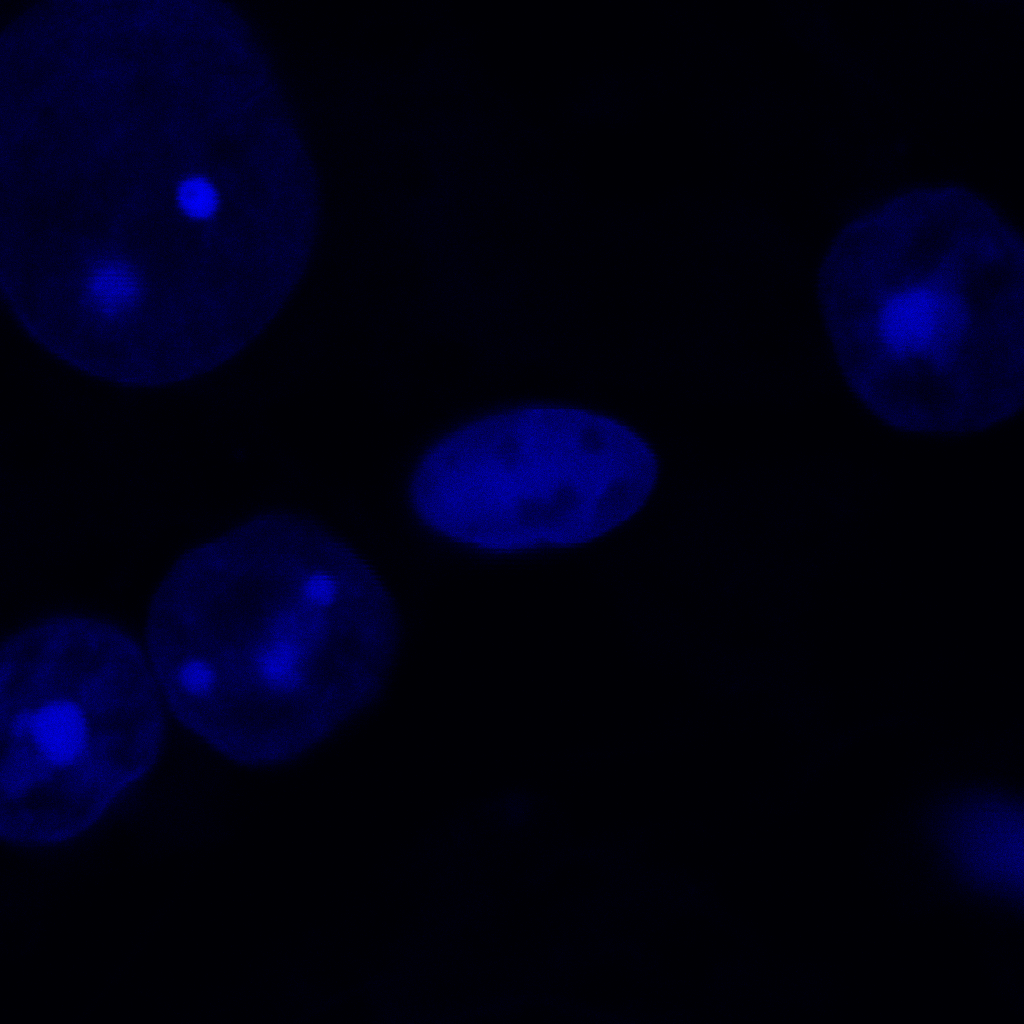

Supplement: Supplementary file 10 [file Data_Sheet_7.ZIP › Fig4D/WT/wt tub.lif_9-10_z0_ch00.tif]

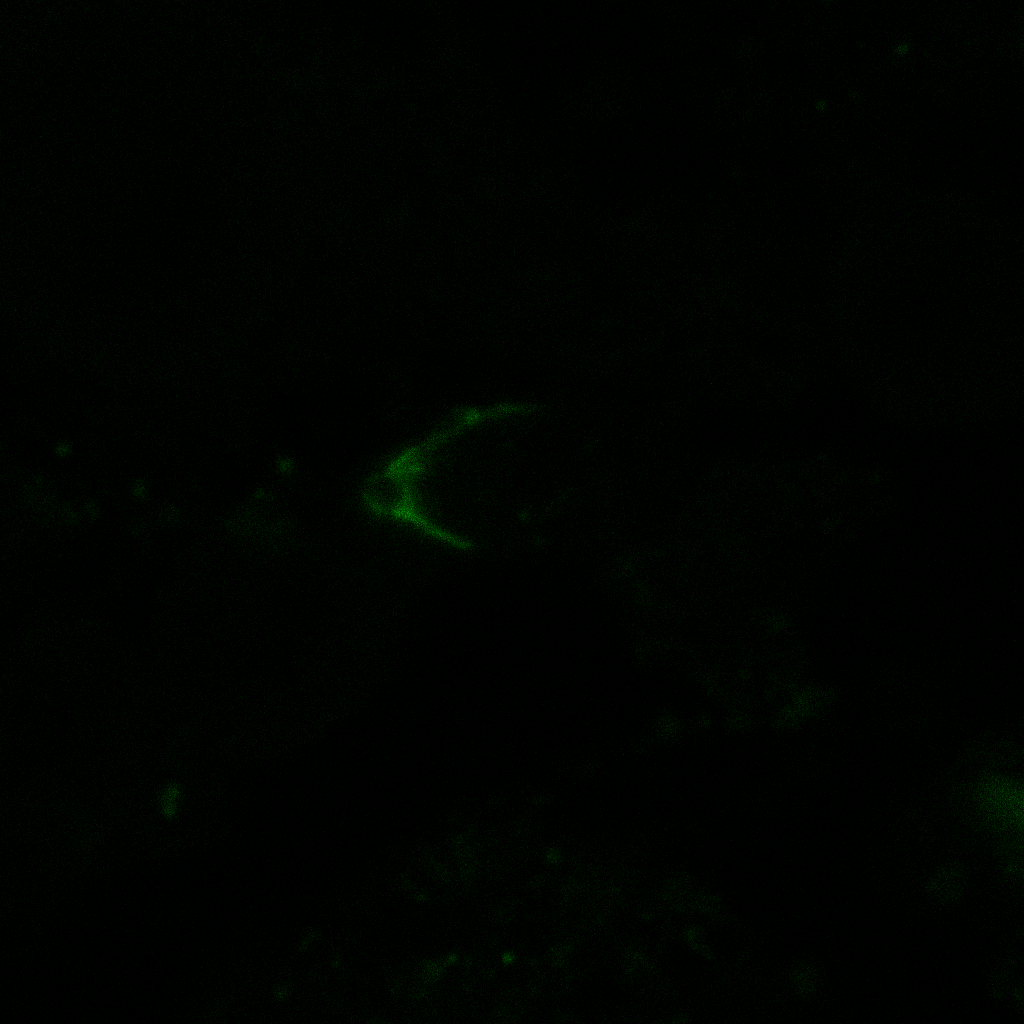

Supplement: Supplementary file 10 [file Data_Sheet_7.ZIP › Fig4D/WT/wt tub.lif_9-10_z0_ch01.tif]

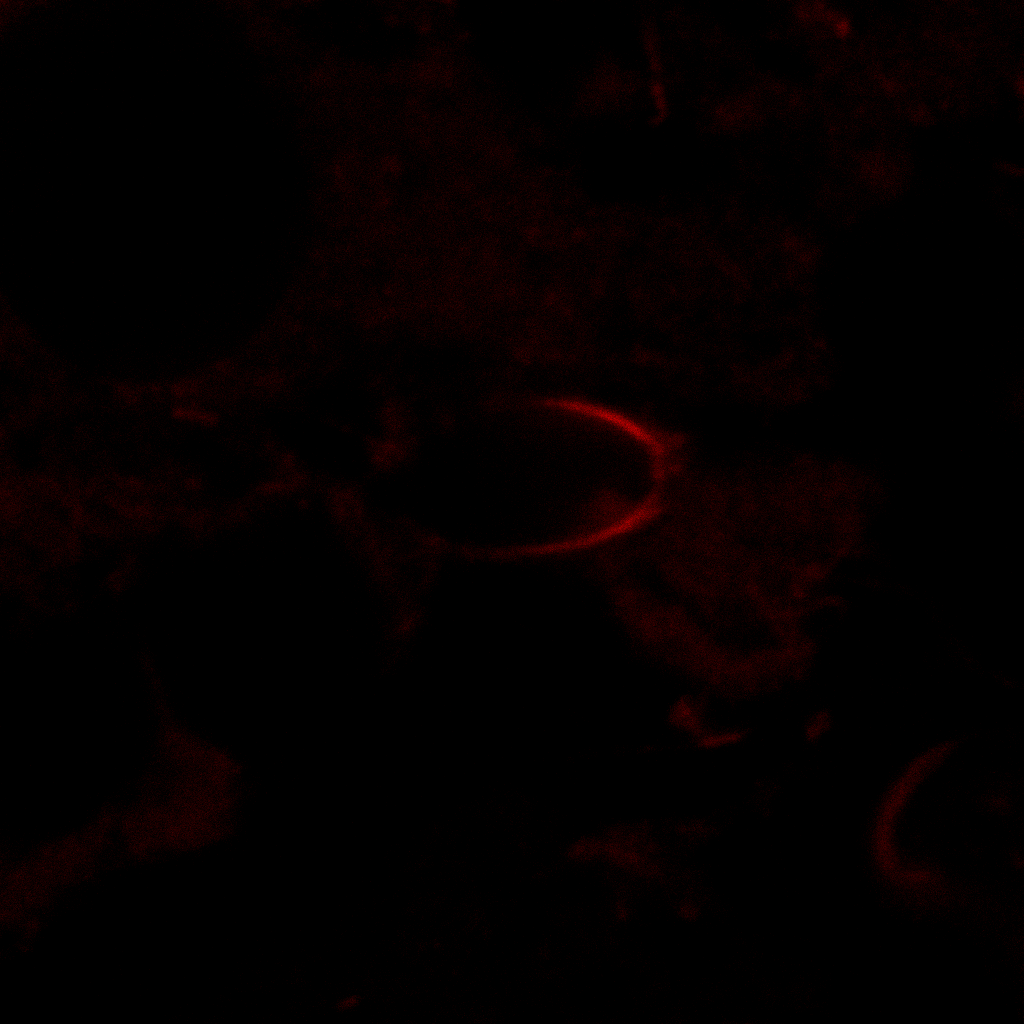

Supplement: Supplementary file 10 [file Data_Sheet_7.ZIP › Fig4D/WT/wt tub.lif_9-10_z0_ch02.tif]

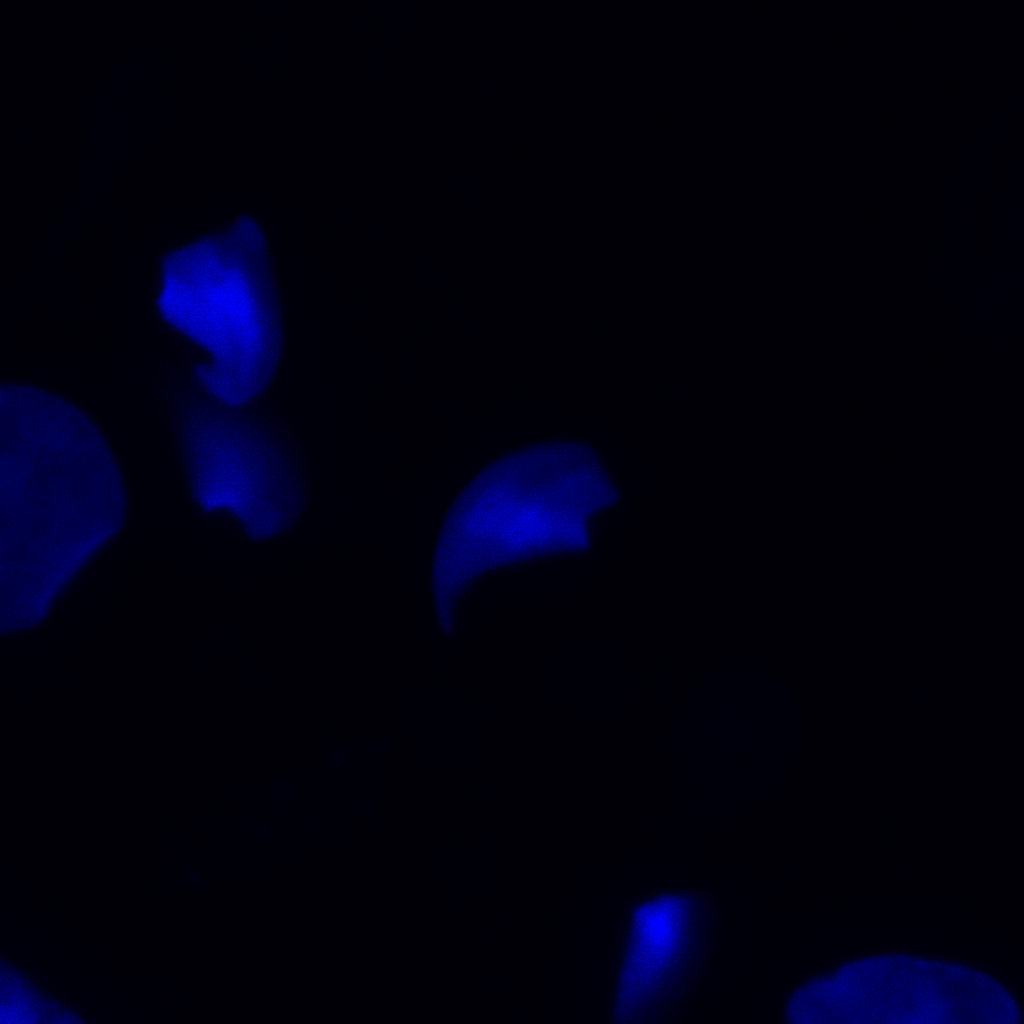

Supplement: Supplementary file 12 [file Data_Sheet_9.ZIP › Fig5A/ko/CFAP53 KO 1 10.lif_13 14_Processed001_ch00.tif]

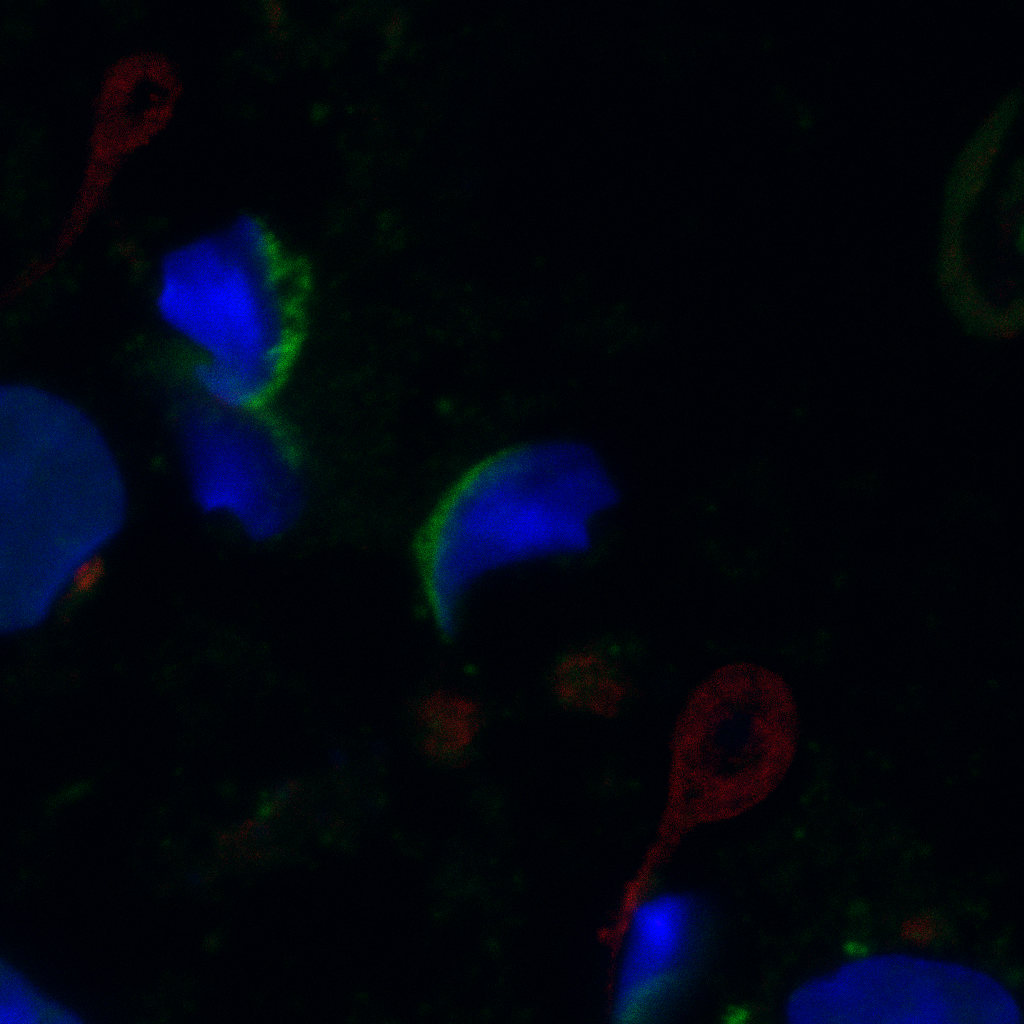

Supplement: Supplementary file 12 [file Data_Sheet_9.ZIP › Fig5A/ko/CFAP53 KO 1 10.lif_13 14_Processed001_ch01 merge.tif]
