# Supplementary figures and images for: Essential Role of CFAP53 in Sperm Flagellum Biogenesis (part 2 of 3)
Source: Front Cell Dev Biol. 2021 May 28;9:676910. doi: 10.3389/fcell.2021.676910 (PMC8195676; doi:10.3389/fcell.2021.676910)

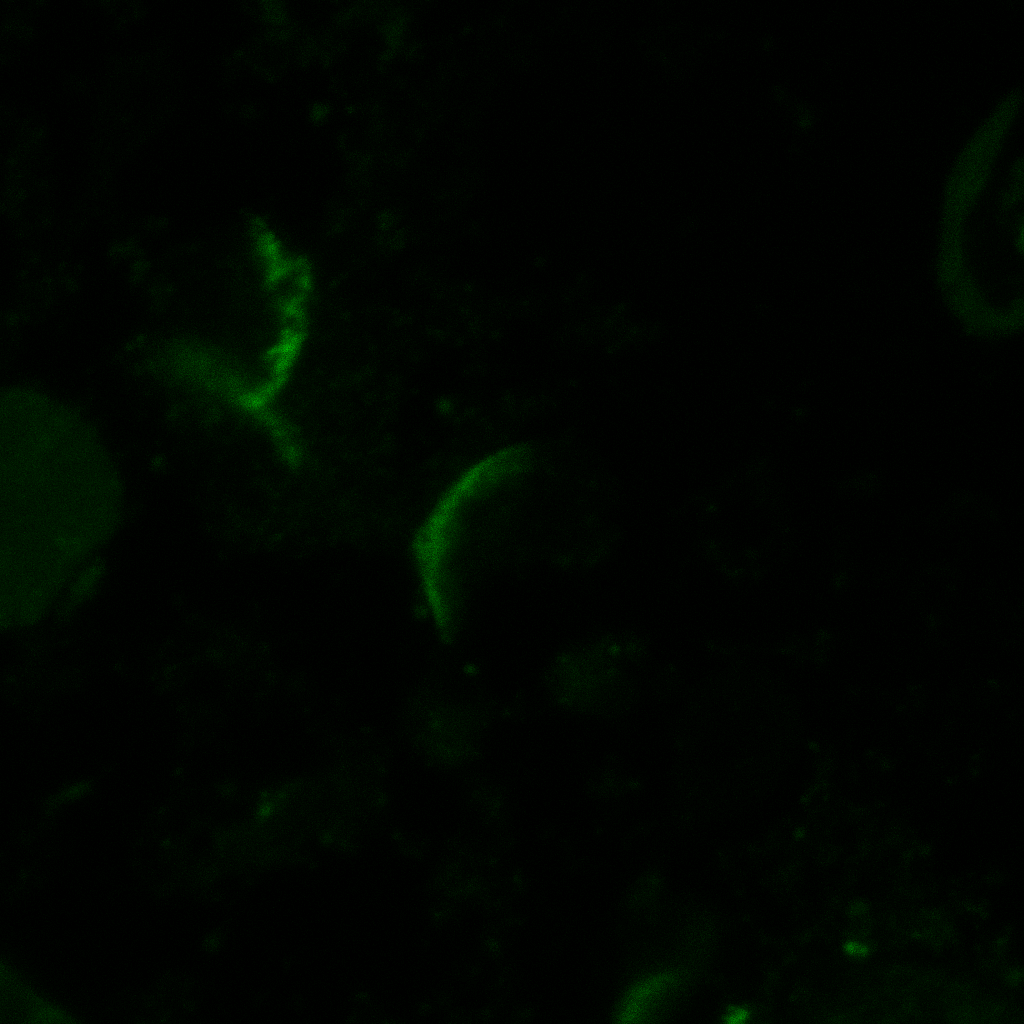

Supplement: Supplementary file 12 [file Data_Sheet_9.ZIP › Fig5A/ko/CFAP53 KO 1 10.lif_13 14_Processed001_ch01.tif]

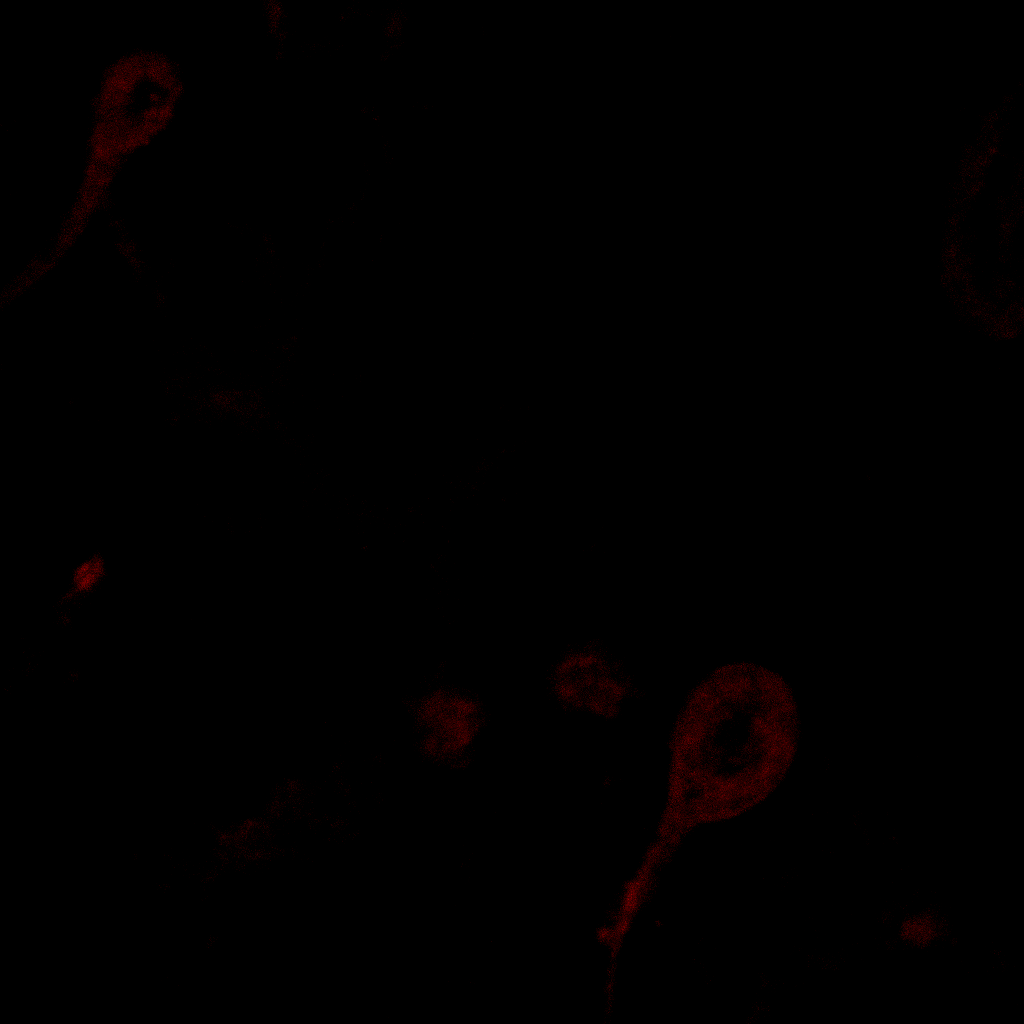

Supplement: Supplementary file 12 [file Data_Sheet_9.ZIP › Fig5A/ko/CFAP53 KO 1 10.lif_13 14_Processed001_ch02.tif]

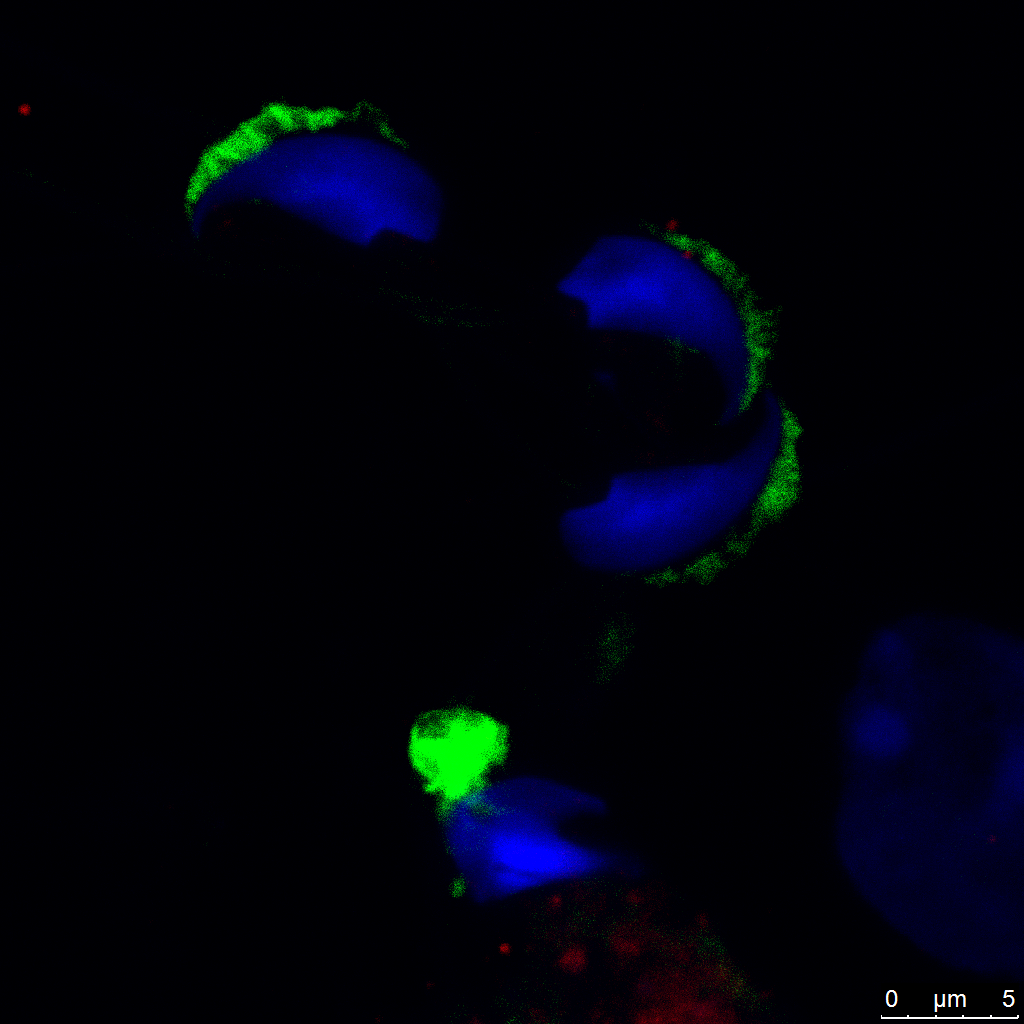

Supplement: Supplementary file 12 [file Data_Sheet_9.ZIP › Fig5A/ko/cfap53 pna m2 1 10.lif_ko 15 16_Processed001.tif]

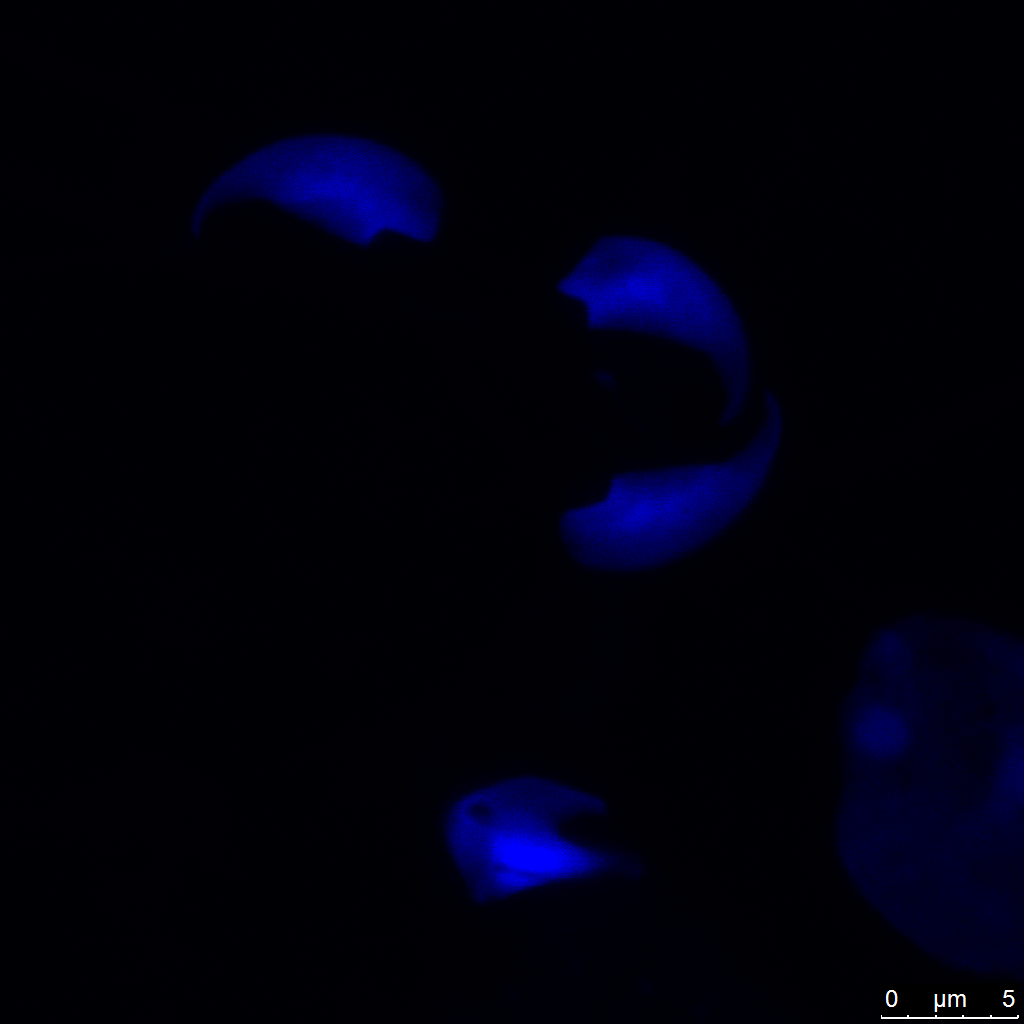

Supplement: Supplementary file 12 [file Data_Sheet_9.ZIP › Fig5A/ko/cfap53 pna m2 1 10.lif_ko 15 16_Processed001_ch00.tif]

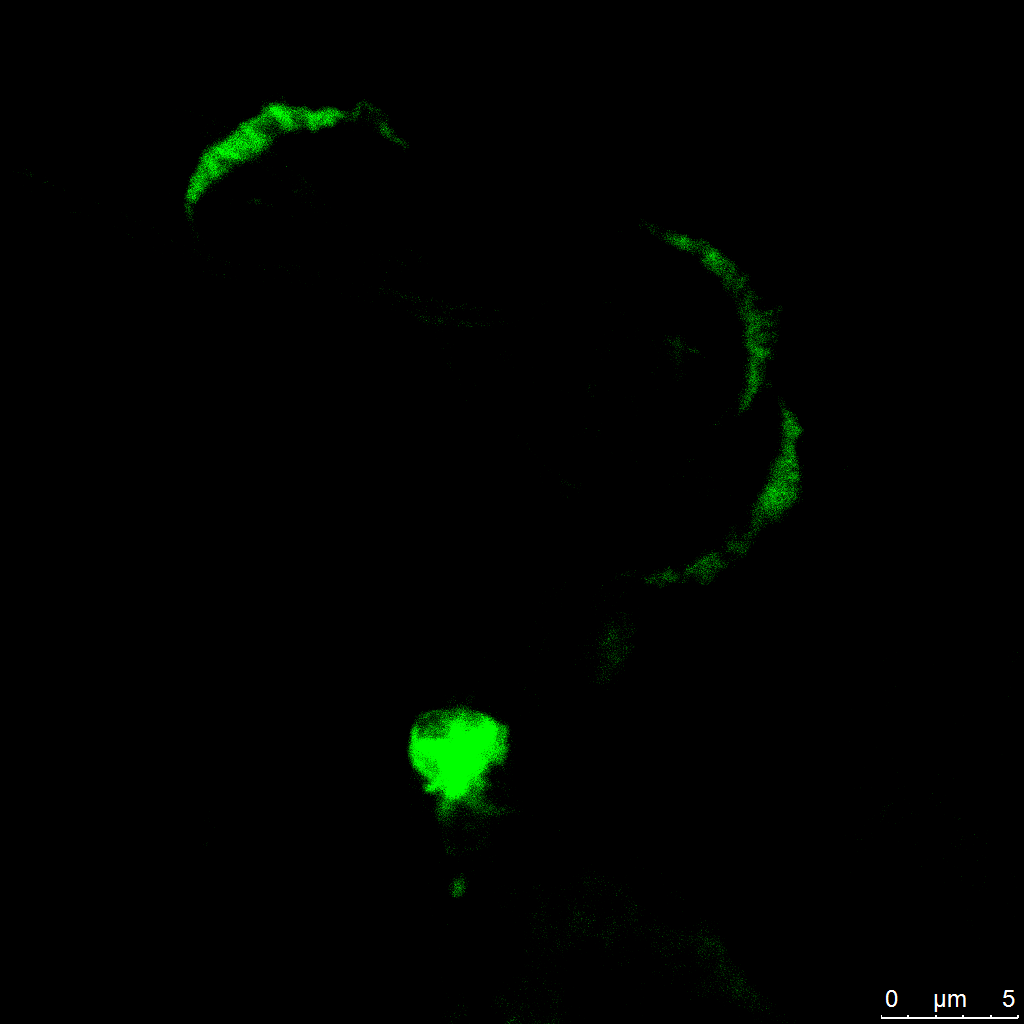

Supplement: Supplementary file 12 [file Data_Sheet_9.ZIP › Fig5A/ko/cfap53 pna m2 1 10.lif_ko 15 16_Processed001_ch01.tif]

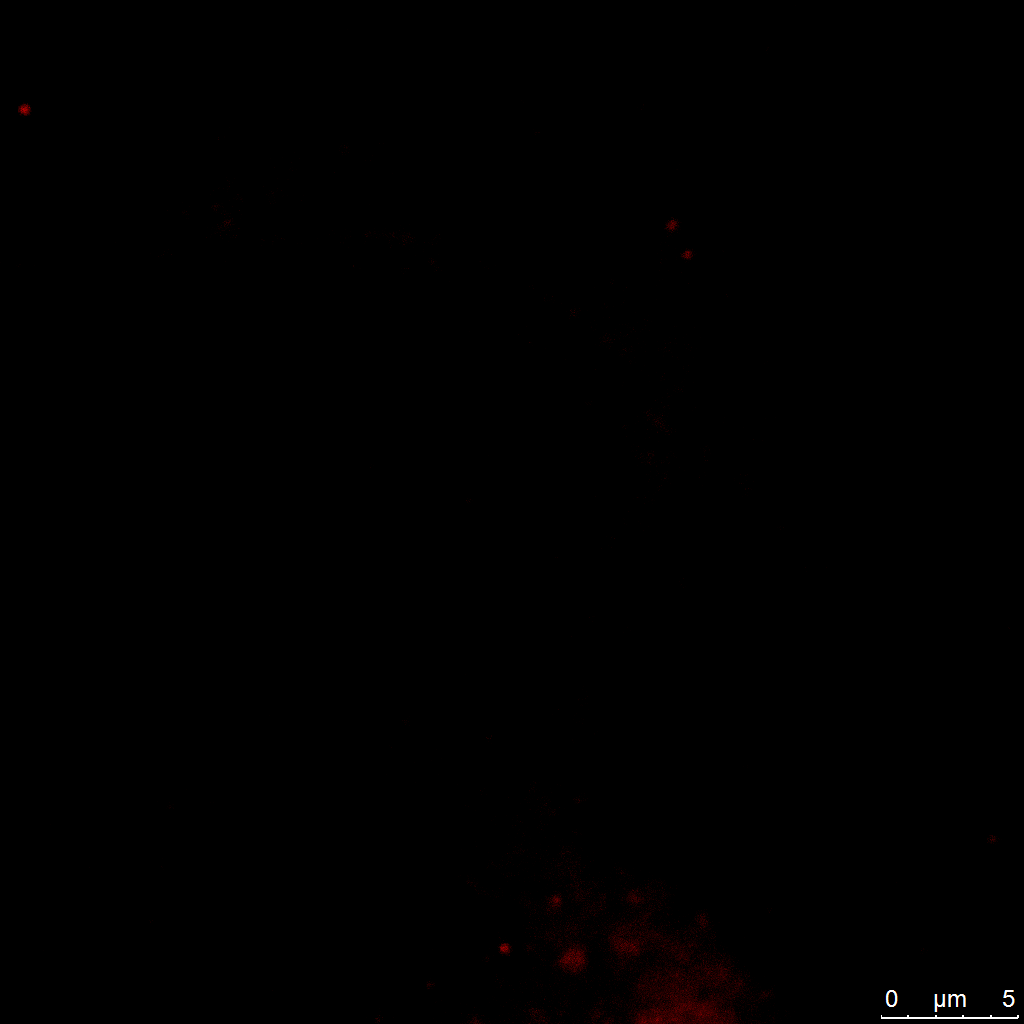

Supplement: Supplementary file 12 [file Data_Sheet_9.ZIP › Fig5A/ko/cfap53 pna m2 1 10.lif_ko 15 16_Processed001_ch02.tif]

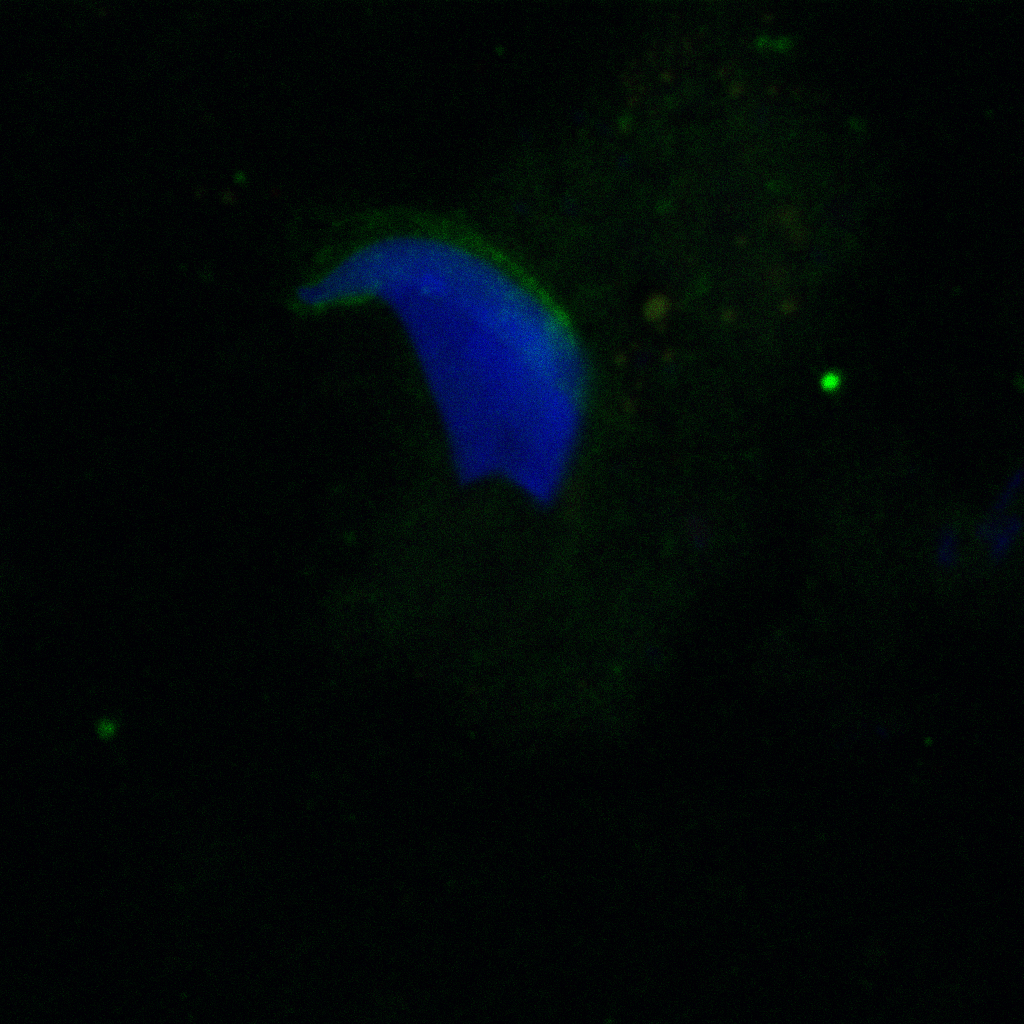

Supplement: Supplementary file 12 [file Data_Sheet_9.ZIP › Fig5A/ko/KO m5.lif_11-13 2_z0.tif]

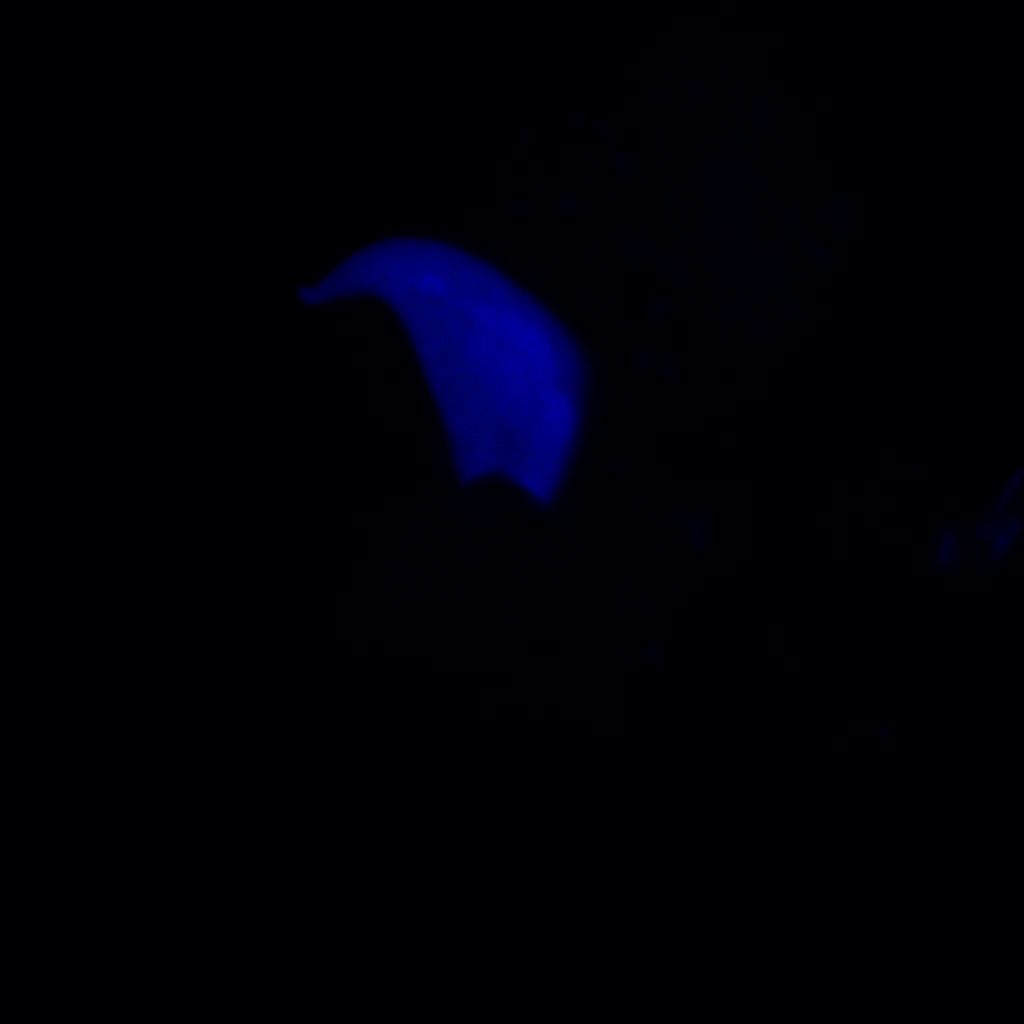

Supplement: Supplementary file 12 [file Data_Sheet_9.ZIP › Fig5A/ko/KO m5.lif_11-13 2_z0_ch00.tif]

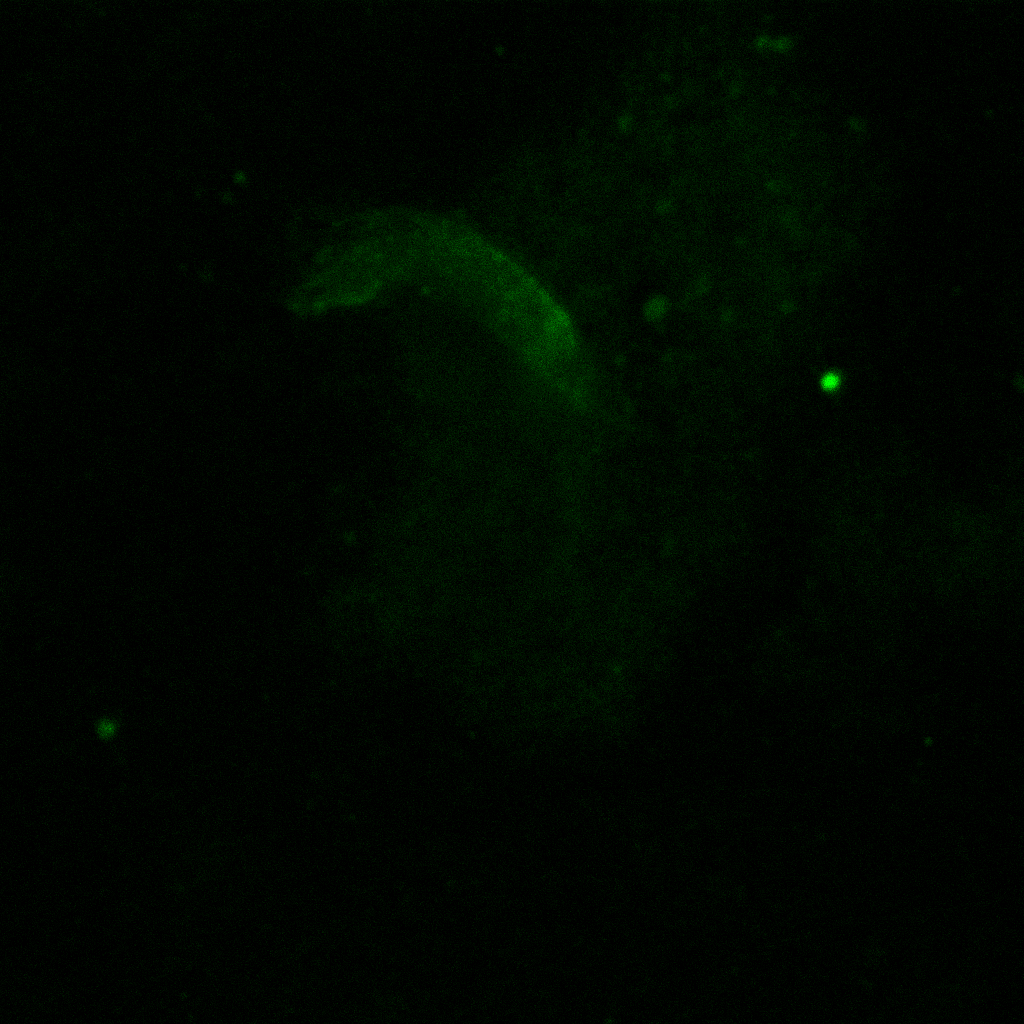

Supplement: Supplementary file 12 [file Data_Sheet_9.ZIP › Fig5A/ko/KO m5.lif_11-13 2_z0_ch01.tif]

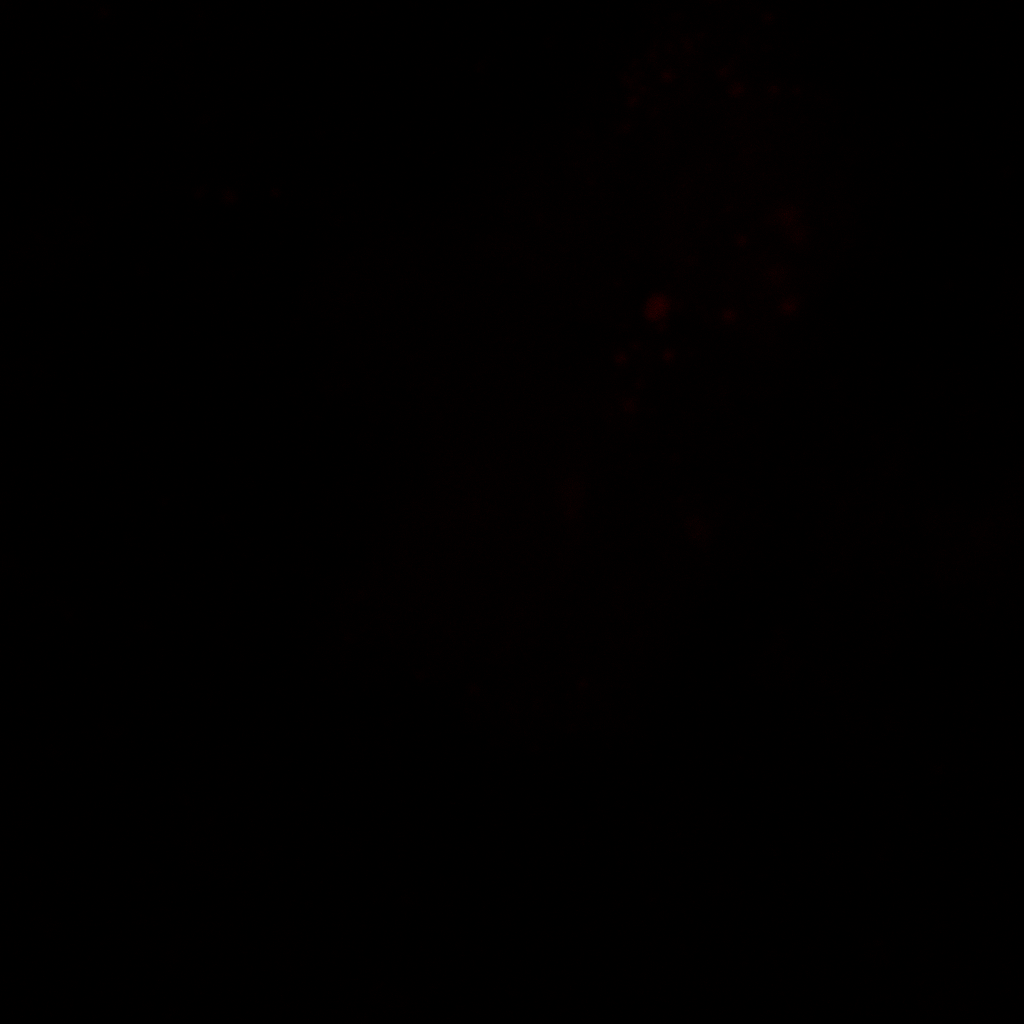

Supplement: Supplementary file 12 [file Data_Sheet_9.ZIP › Fig5A/ko/KO m5.lif_11-13 2_z0_ch02.tif]

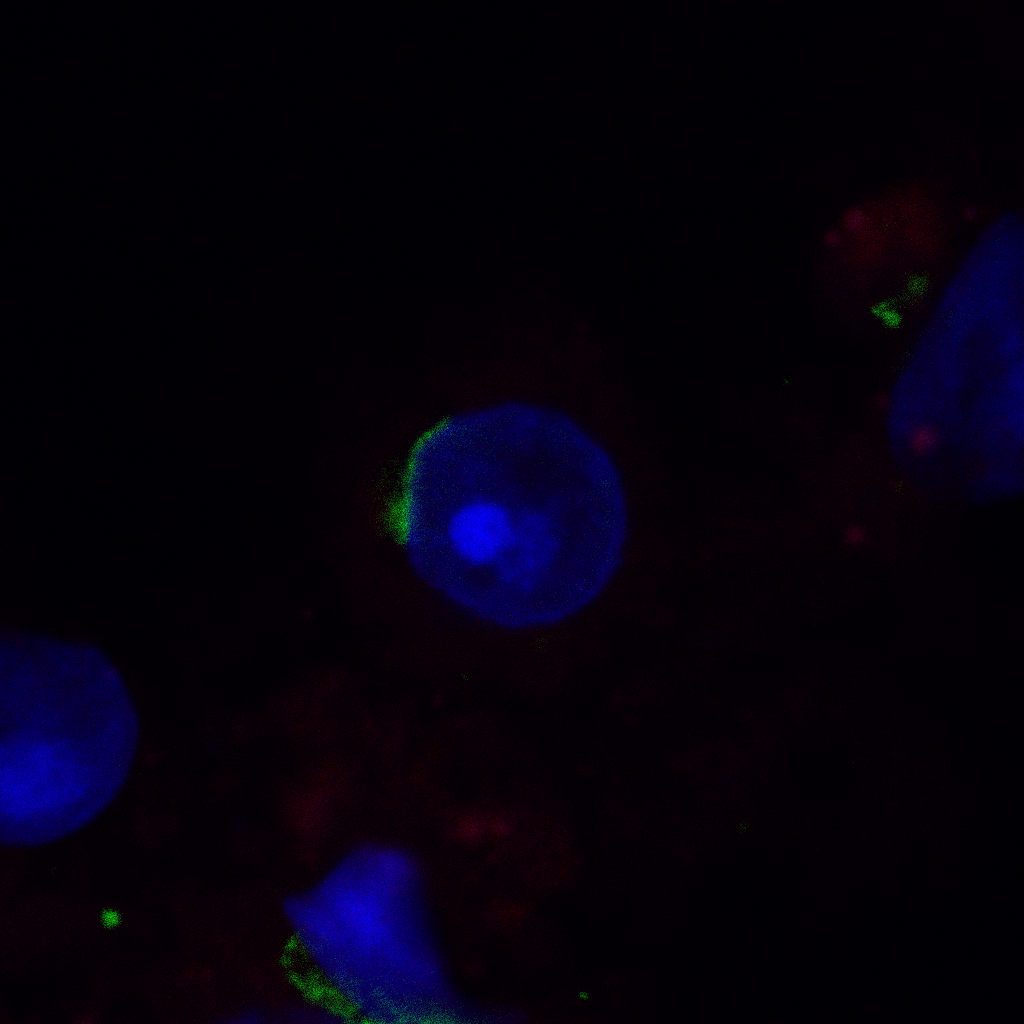

Supplement: Supplementary file 12 [file Data_Sheet_9.ZIP › Fig5A/ko/KO m5.lif_5-6_z3.tif]

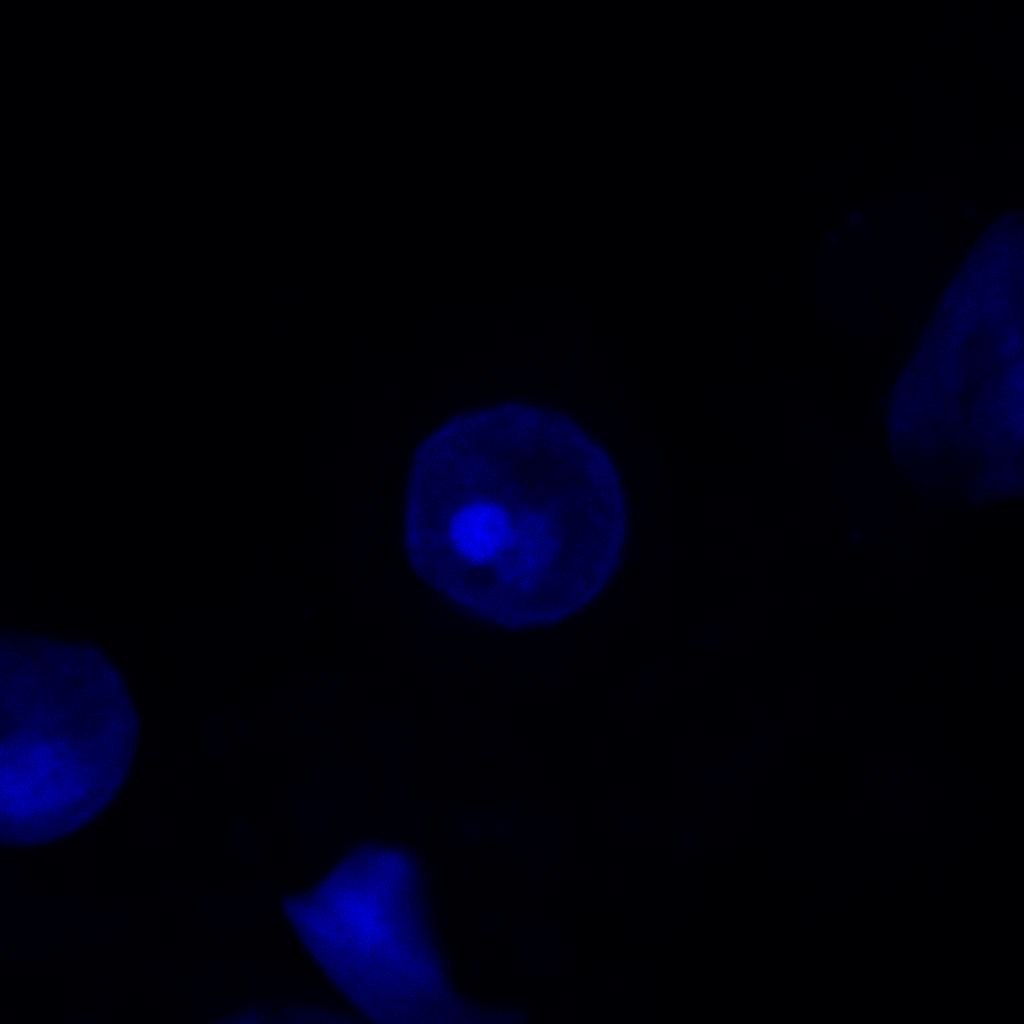

Supplement: Supplementary file 12 [file Data_Sheet_9.ZIP › Fig5A/ko/KO m5.lif_5-6_z3_ch00.tif]

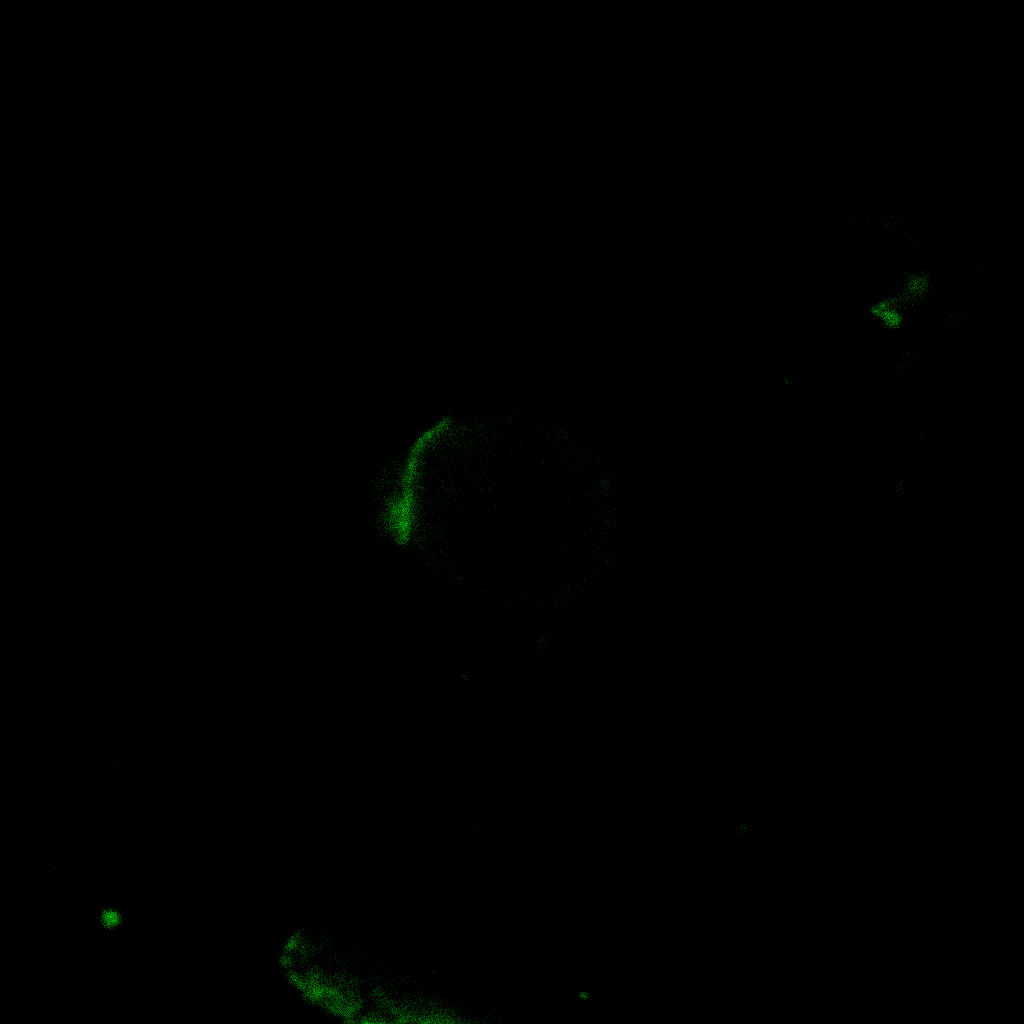

Supplement: Supplementary file 12 [file Data_Sheet_9.ZIP › Fig5A/ko/KO m5.lif_5-6_z3_ch01.tif]

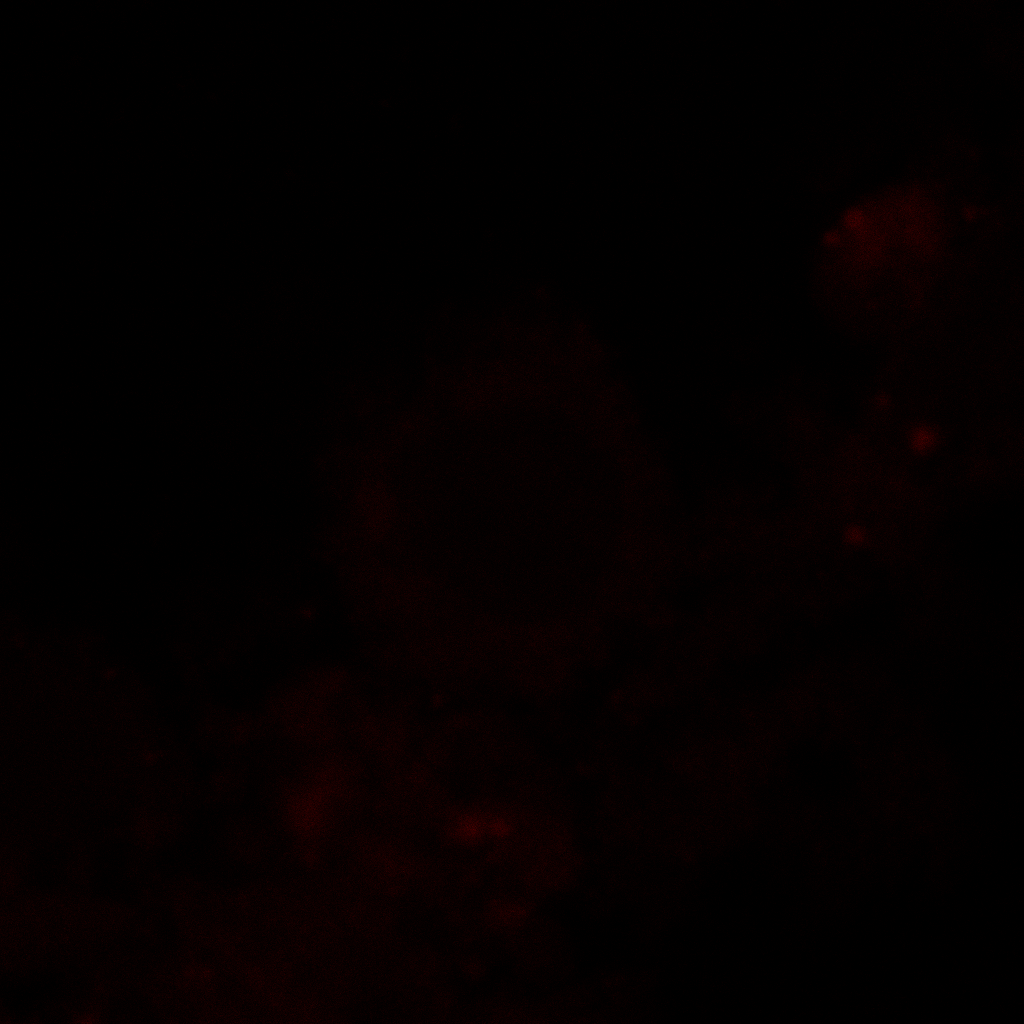

Supplement: Supplementary file 12 [file Data_Sheet_9.ZIP › Fig5A/ko/KO m5.lif_5-6_z3_ch02.tif]

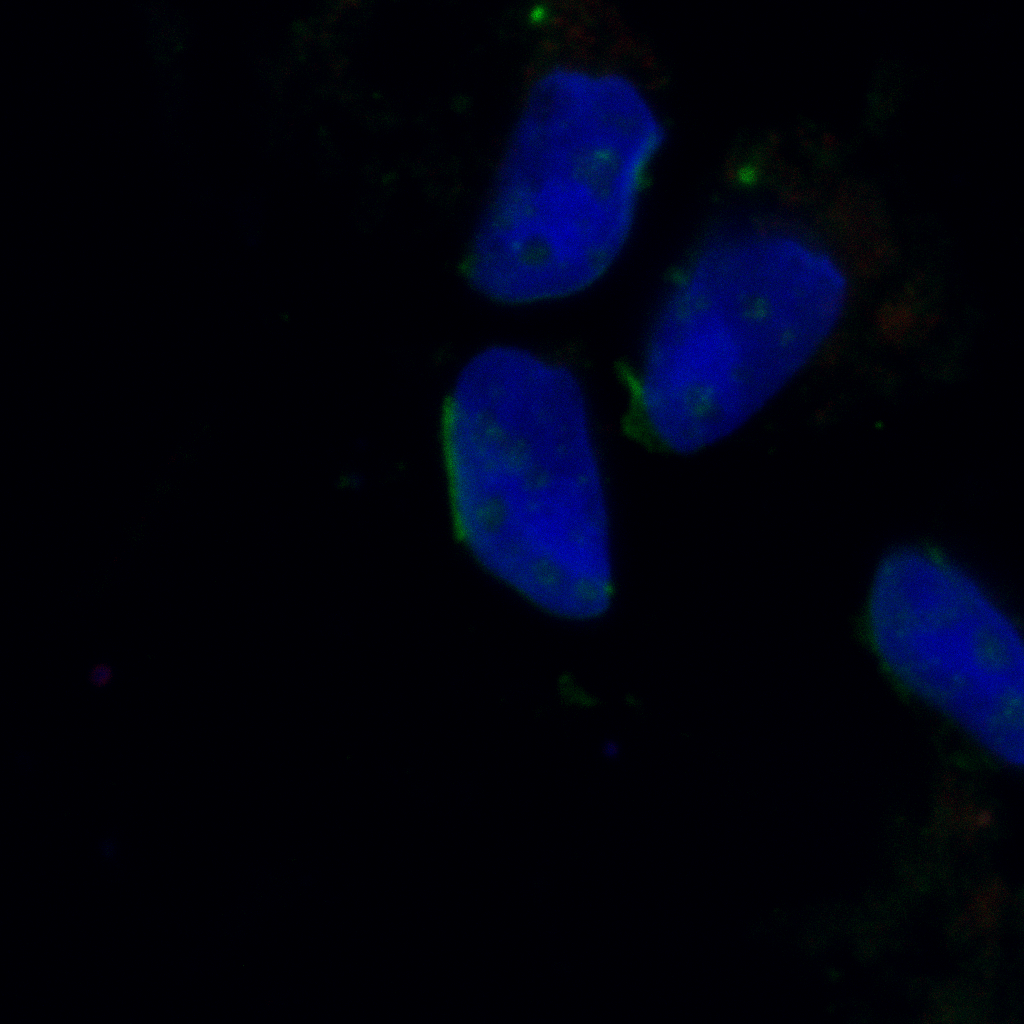

Supplement: Supplementary file 12 [file Data_Sheet_9.ZIP › Fig5A/ko/KO m5.lif_9 10_Processed001.tif]

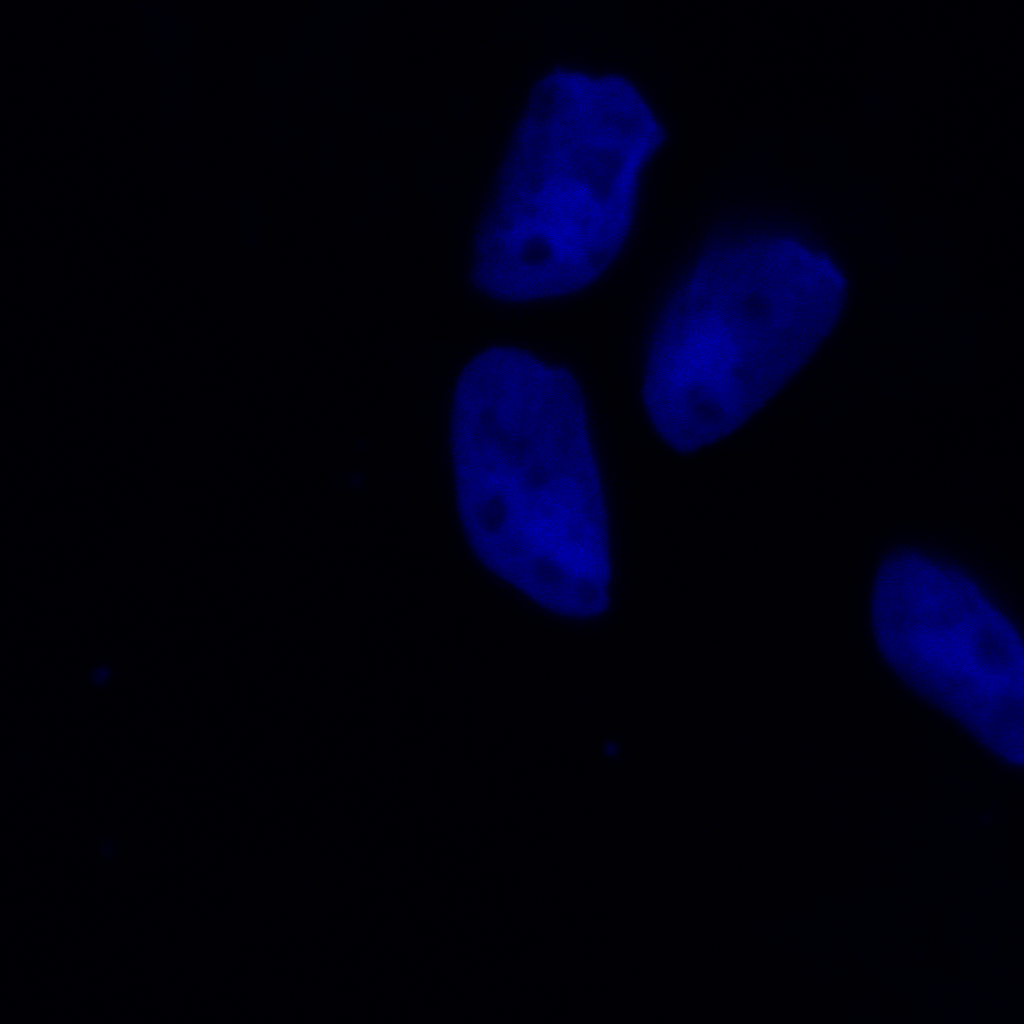

Supplement: Supplementary file 12 [file Data_Sheet_9.ZIP › Fig5A/ko/KO m5.lif_9 10_Processed001_ch00.tif]

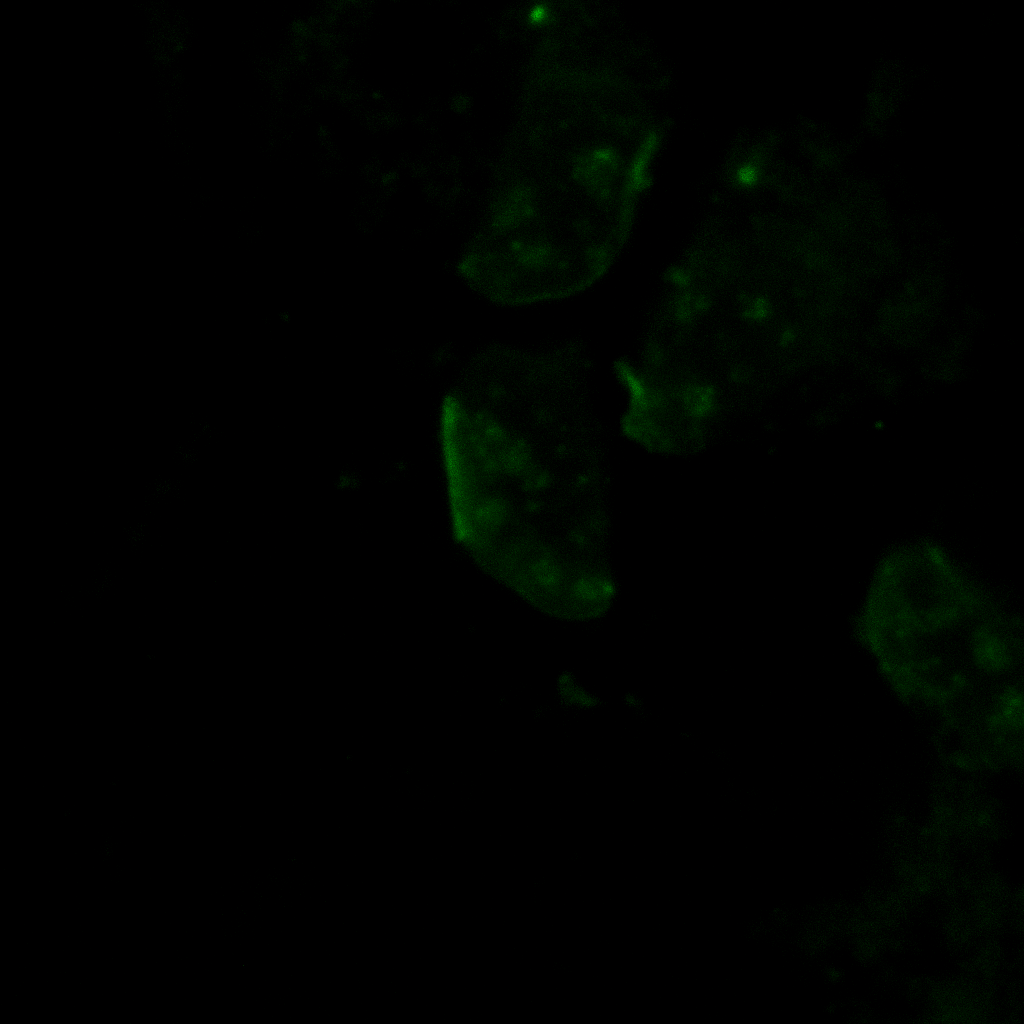

Supplement: Supplementary file 12 [file Data_Sheet_9.ZIP › Fig5A/ko/KO m5.lif_9 10_Processed001_ch01.tif]

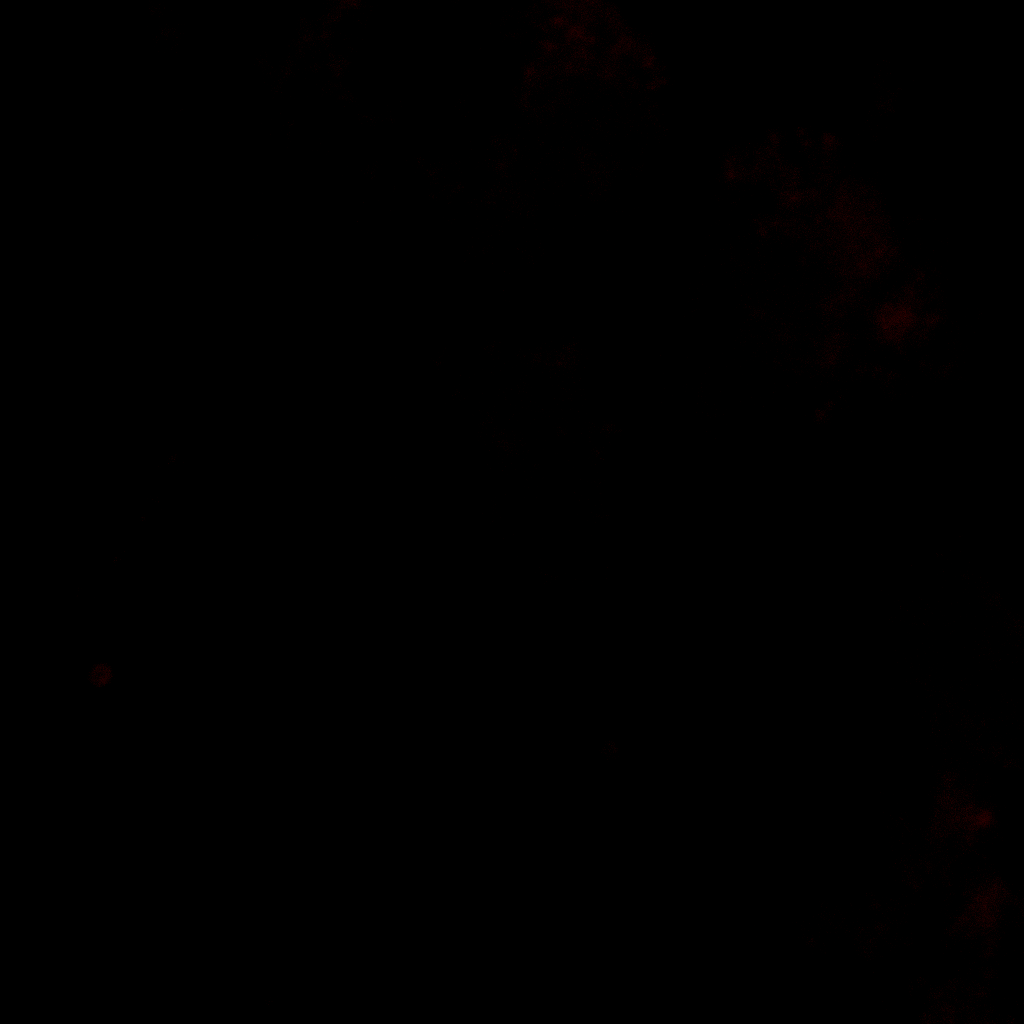

Supplement: Supplementary file 12 [file Data_Sheet_9.ZIP › Fig5A/ko/KO m5.lif_9 10_Processed001_ch02.tif]

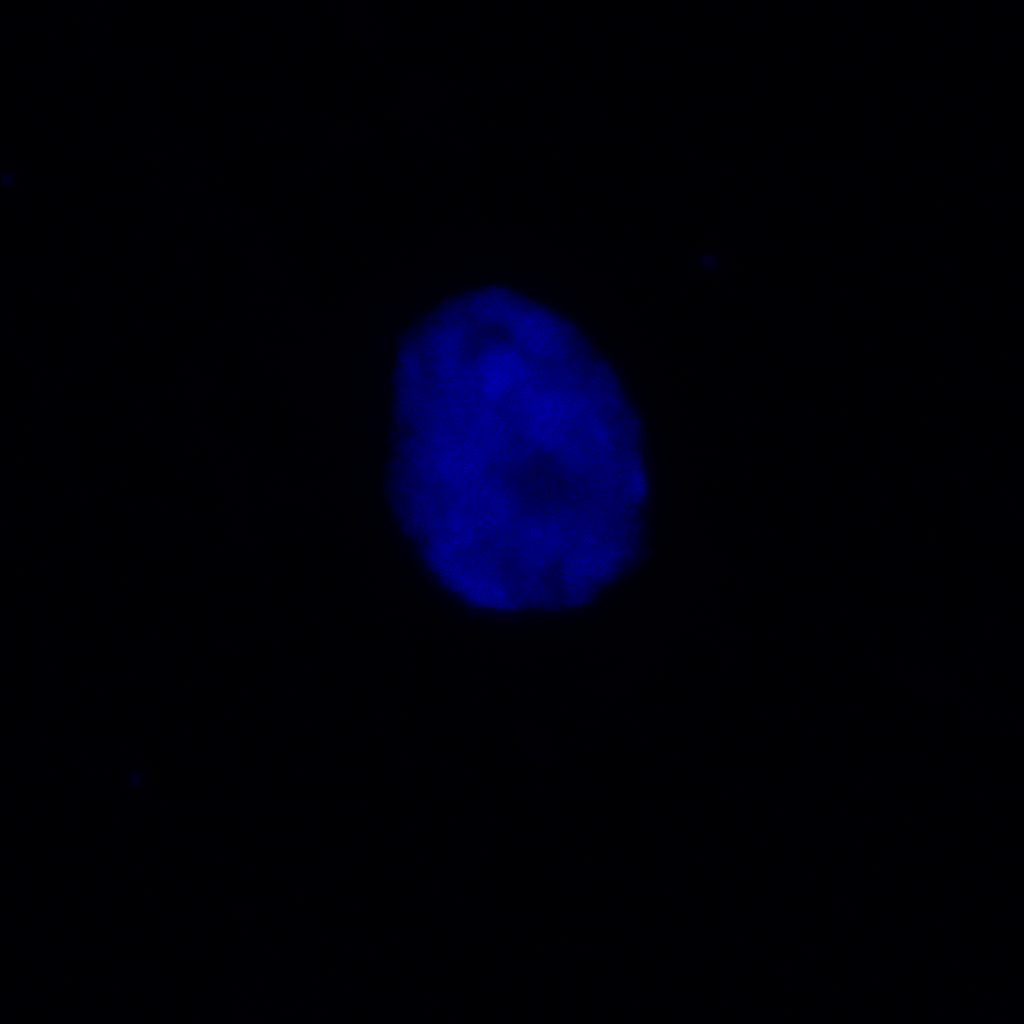

Supplement: Supplementary file 12 [file Data_Sheet_9.ZIP › Fig5A/ko/KO m5.lif_SPERMATOCYTE_Processed001.tif]

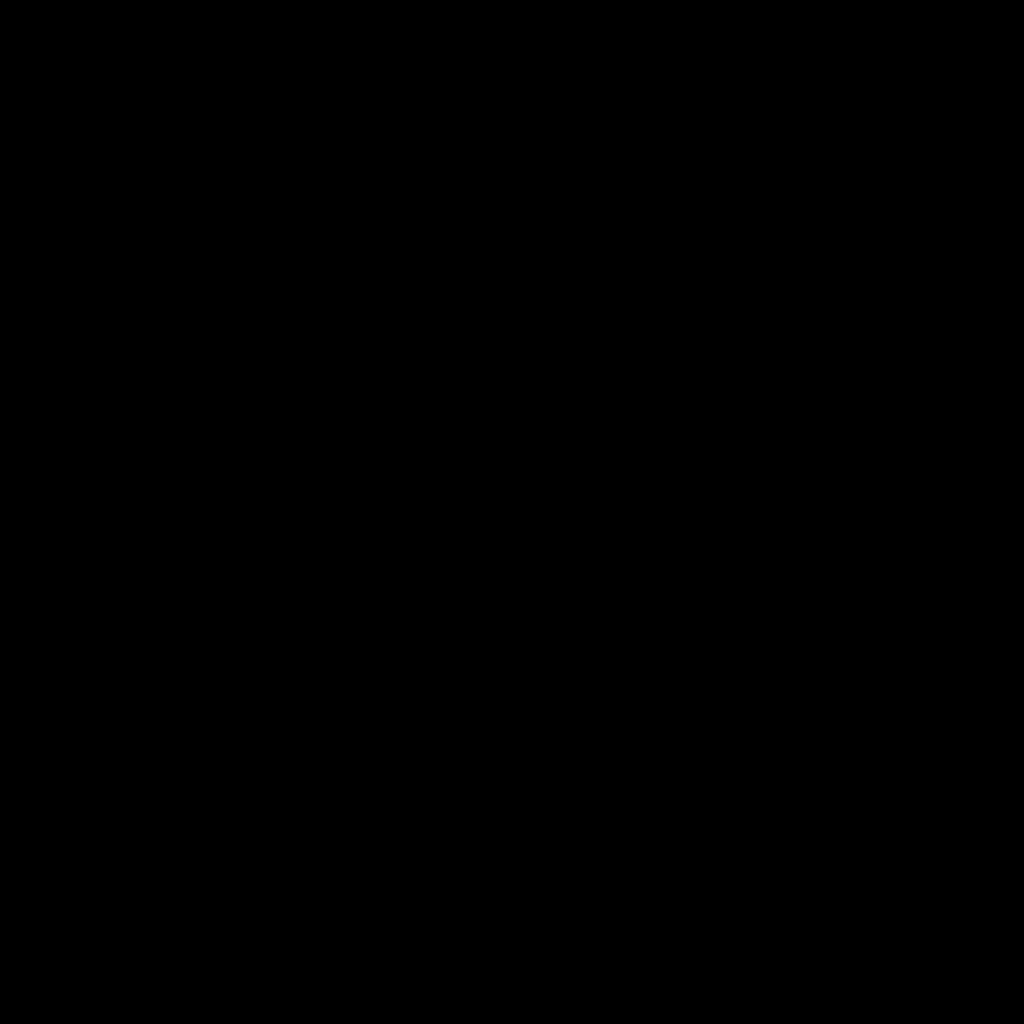

Supplement: Supplementary file 12 [file Data_Sheet_9.ZIP › Fig5A/ko/KO m5.lif_SPERMATOCYTE_Processed001_ch01.tif]

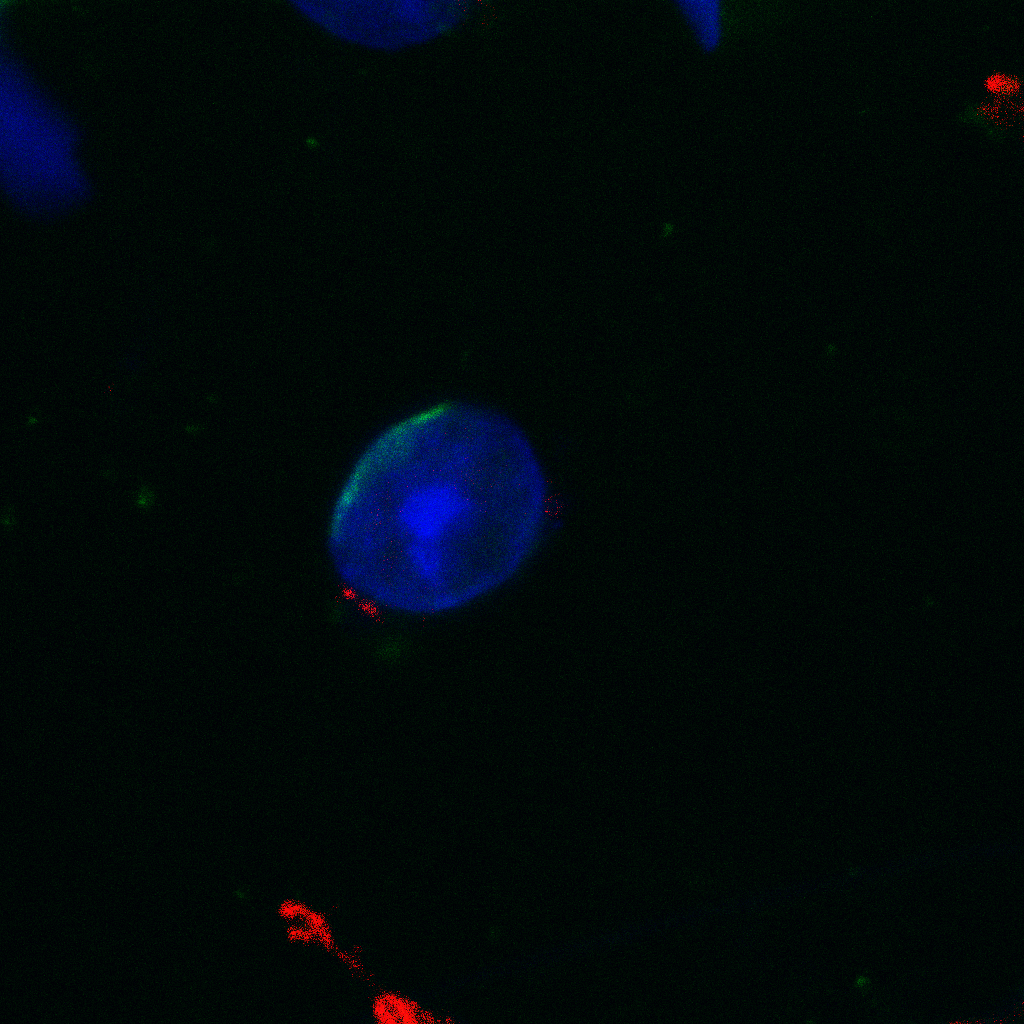

Supplement: Supplementary file 12 [file Data_Sheet_9.ZIP › Fig5A/wt/CCDC11 M5 1 10 48H.lif_Series052mergech00.tif]

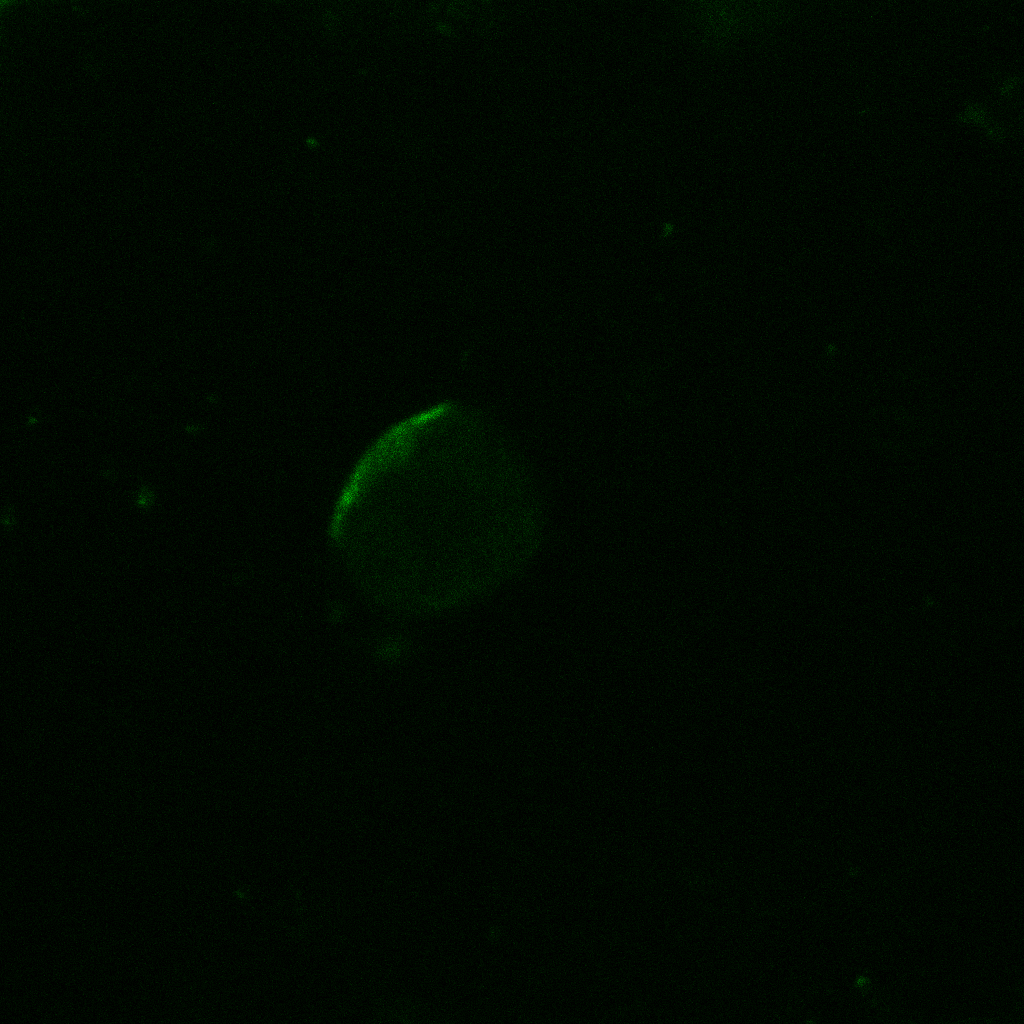

Supplement: Supplementary file 12 [file Data_Sheet_9.ZIP › Fig5A/wt/CCDC11 M5 1 10 48H.lif_Series052_z2_ch01.tif]

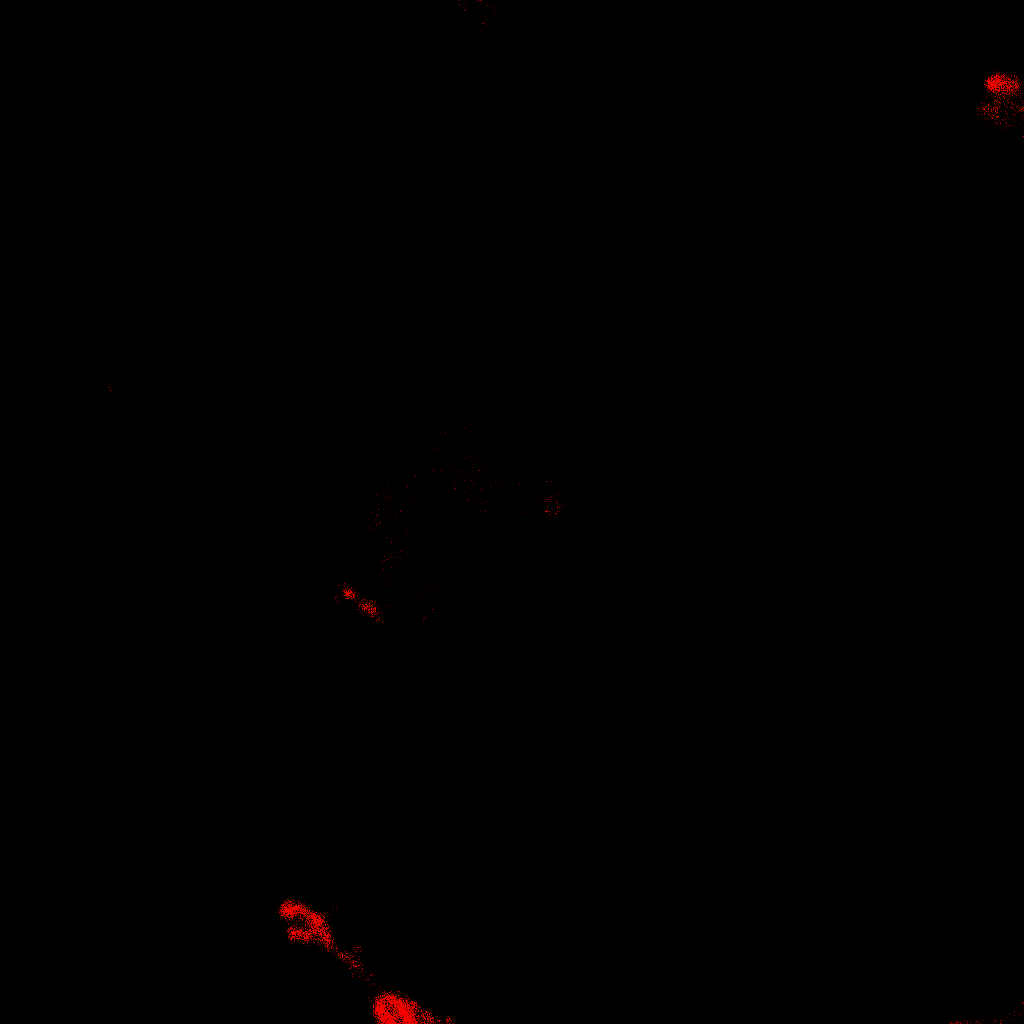

Supplement: Supplementary file 12 [file Data_Sheet_9.ZIP › Fig5A/wt/CCDC11 M5 1 10 48H.lif_Series052_z3_ch02.tif]

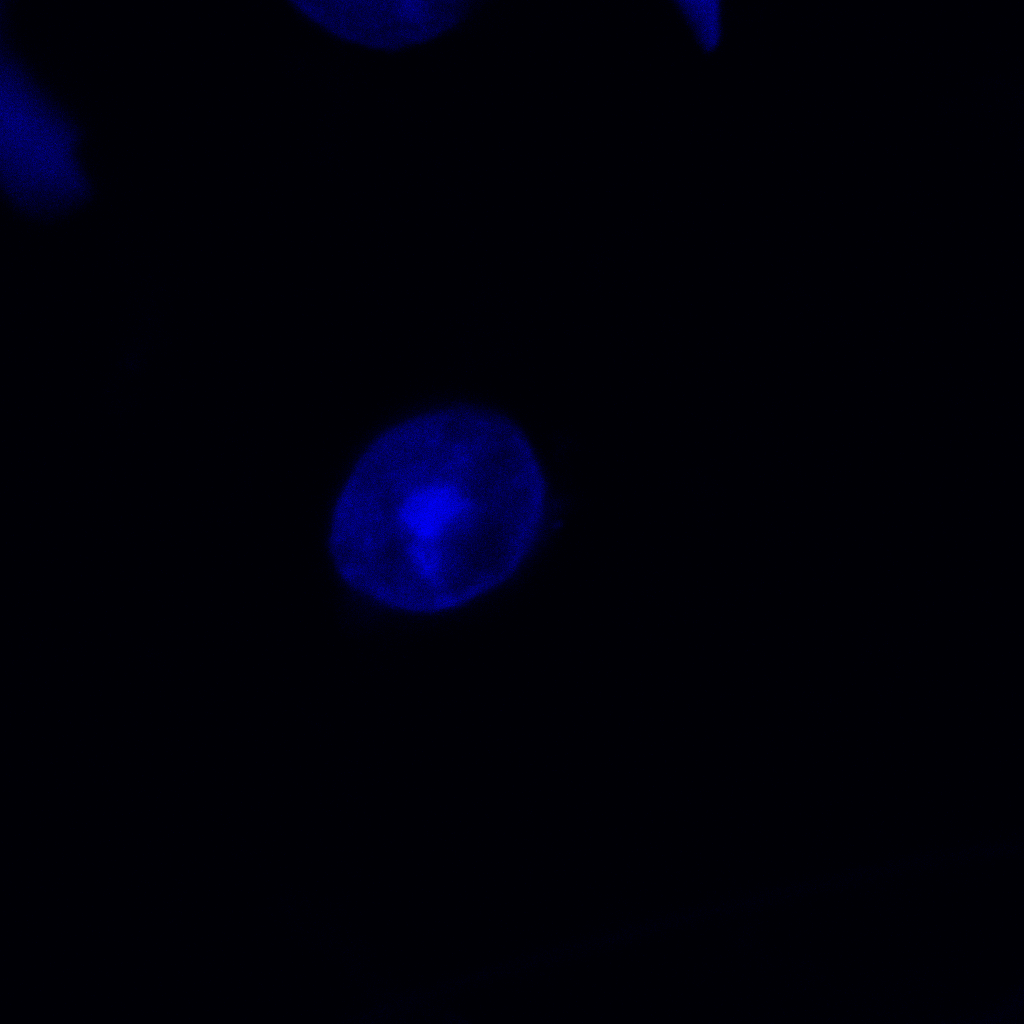

Supplement: Supplementary file 12 [file Data_Sheet_9.ZIP › Fig5A/wt/CCDC11 M5 1 10 48H.lif_Series052_z4_ch00.tif]

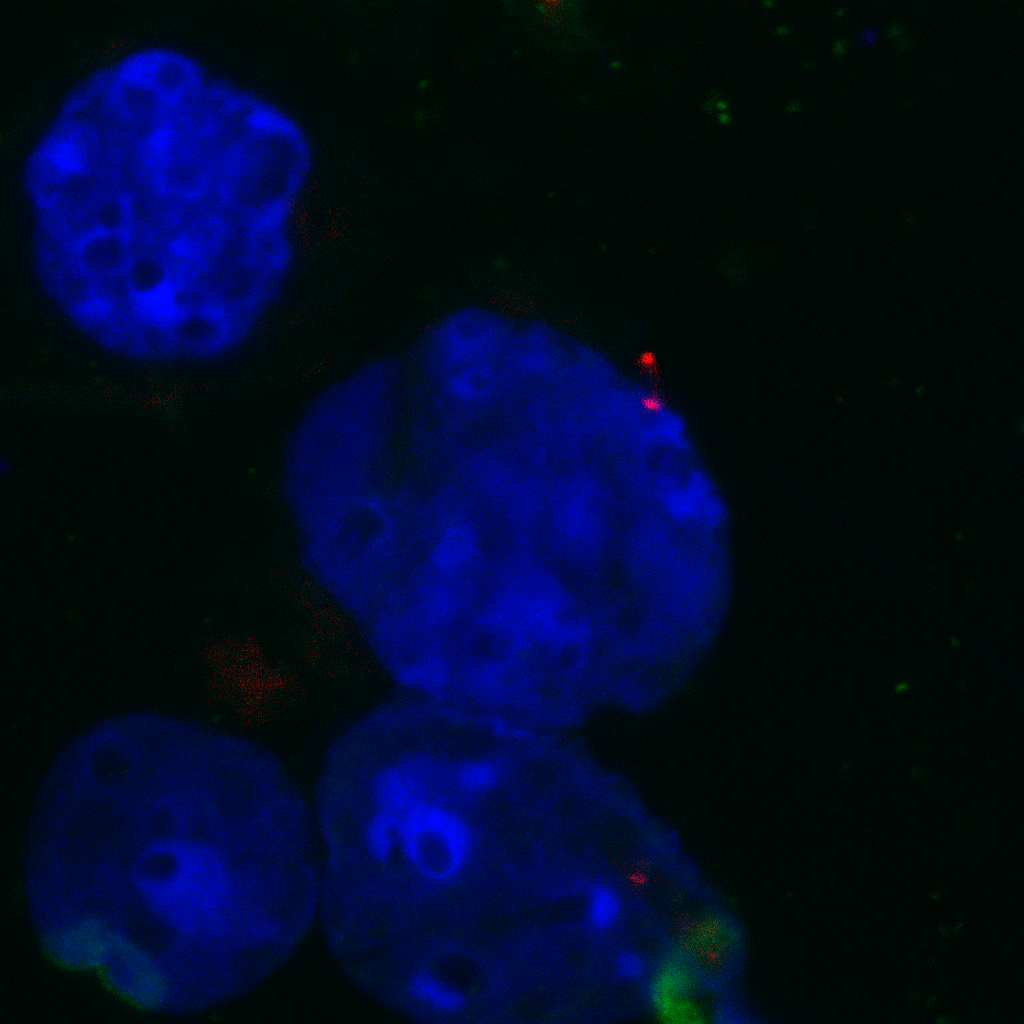

Supplement: Supplementary file 12 [file Data_Sheet_9.ZIP › Fig5A/wt/CCDC11 M5 1 10 48H.lif_SPERMATOCYTE_Processed001.tif]

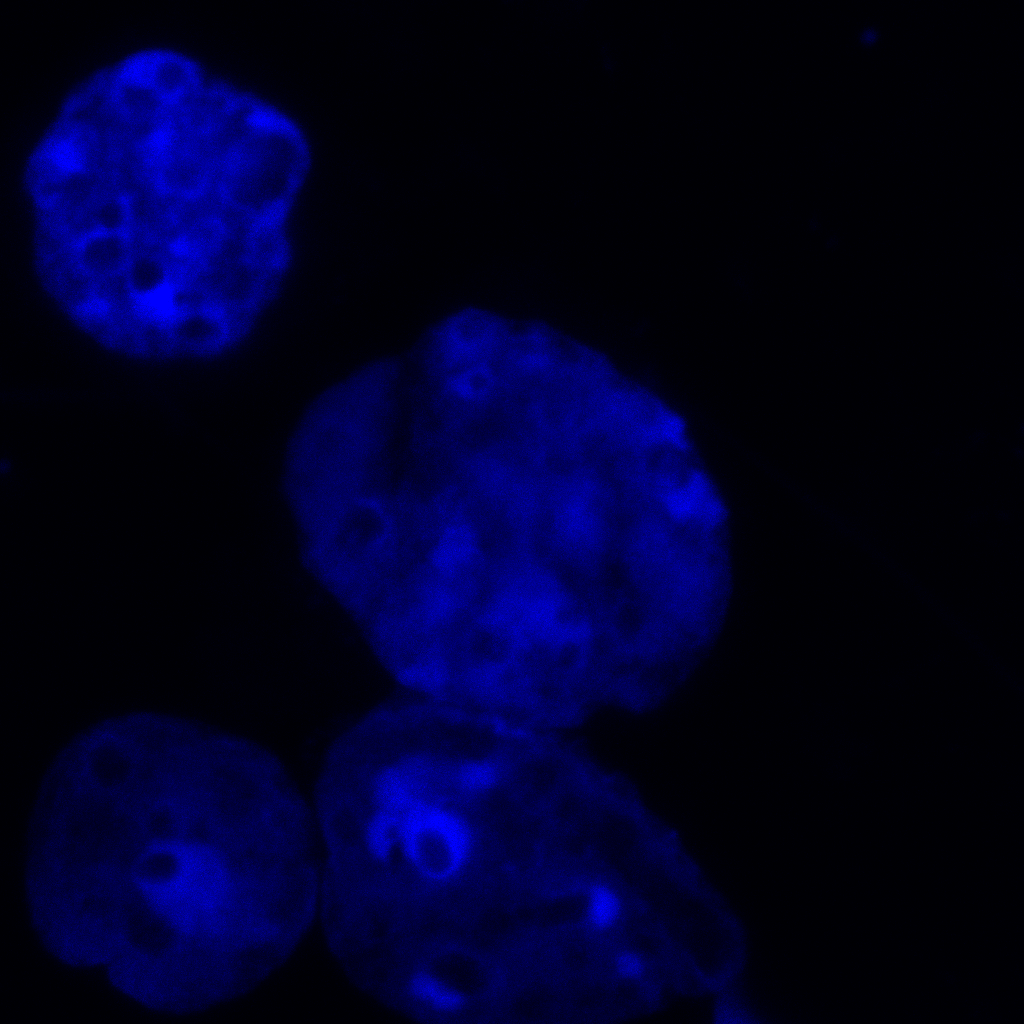

Supplement: Supplementary file 12 [file Data_Sheet_9.ZIP › Fig5A/wt/CCDC11 M5 1 10 48H.lif_SPERMATOCYTE_Processed001_ch00.tif]

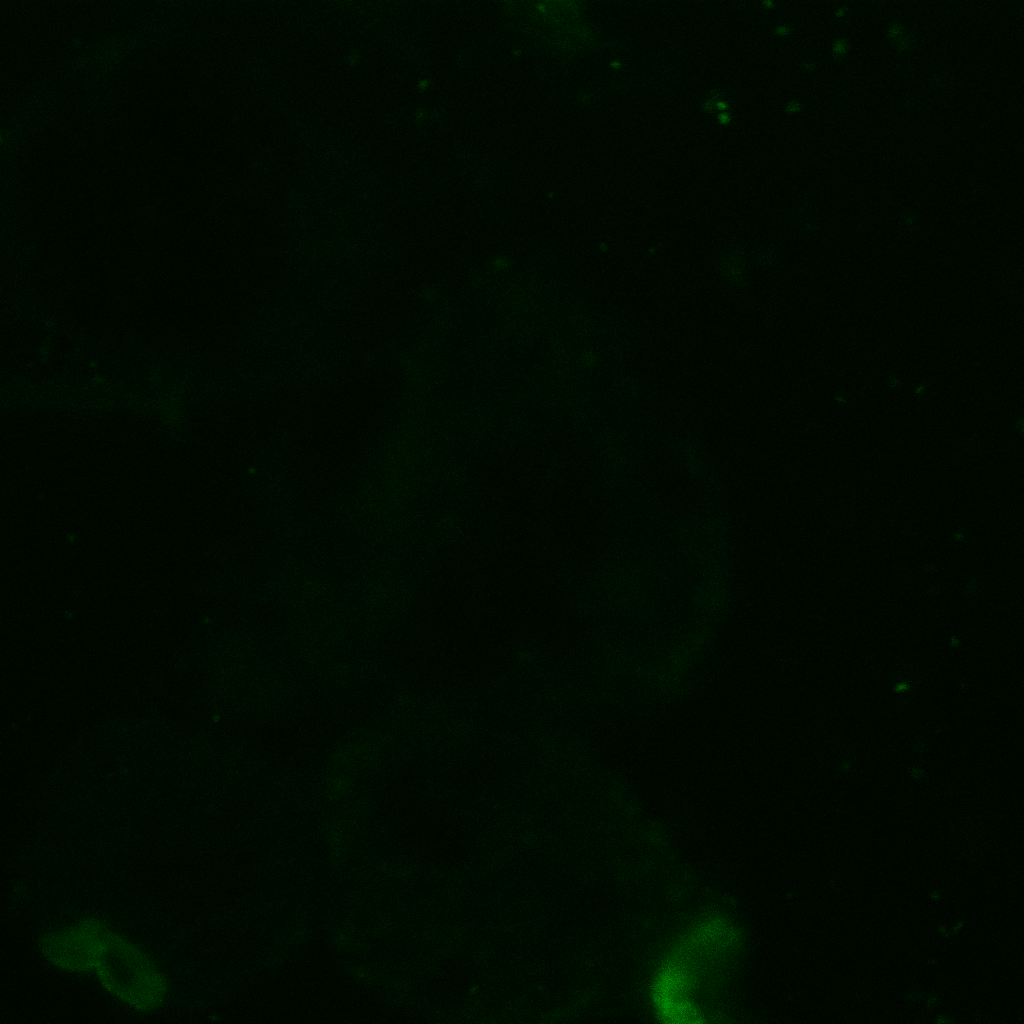

Supplement: Supplementary file 12 [file Data_Sheet_9.ZIP › Fig5A/wt/CCDC11 M5 1 10 48H.lif_SPERMATOCYTE_Processed001_ch01.tif]

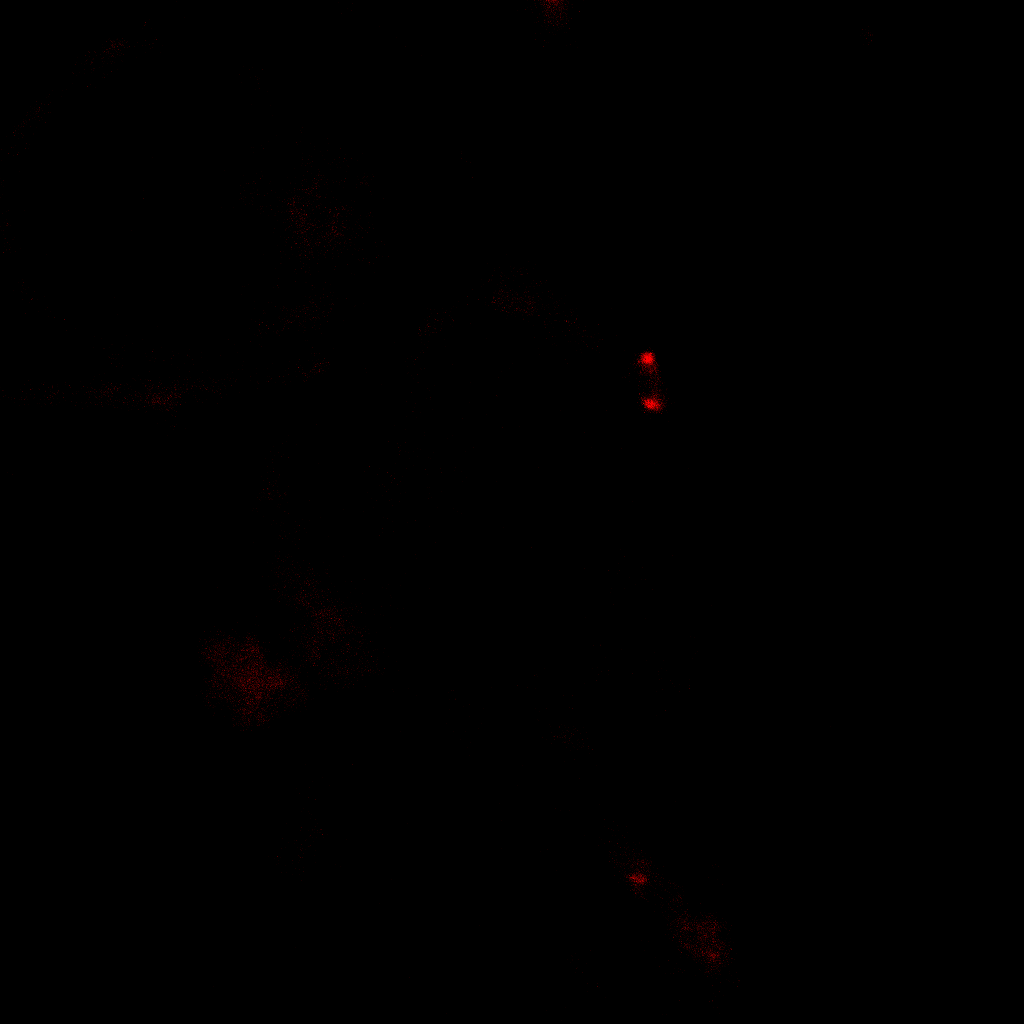

Supplement: Supplementary file 12 [file Data_Sheet_9.ZIP › Fig5A/wt/CCDC11 M5 1 10 48H.lif_SPERMATOCYTE_Processed001_ch02.tif]

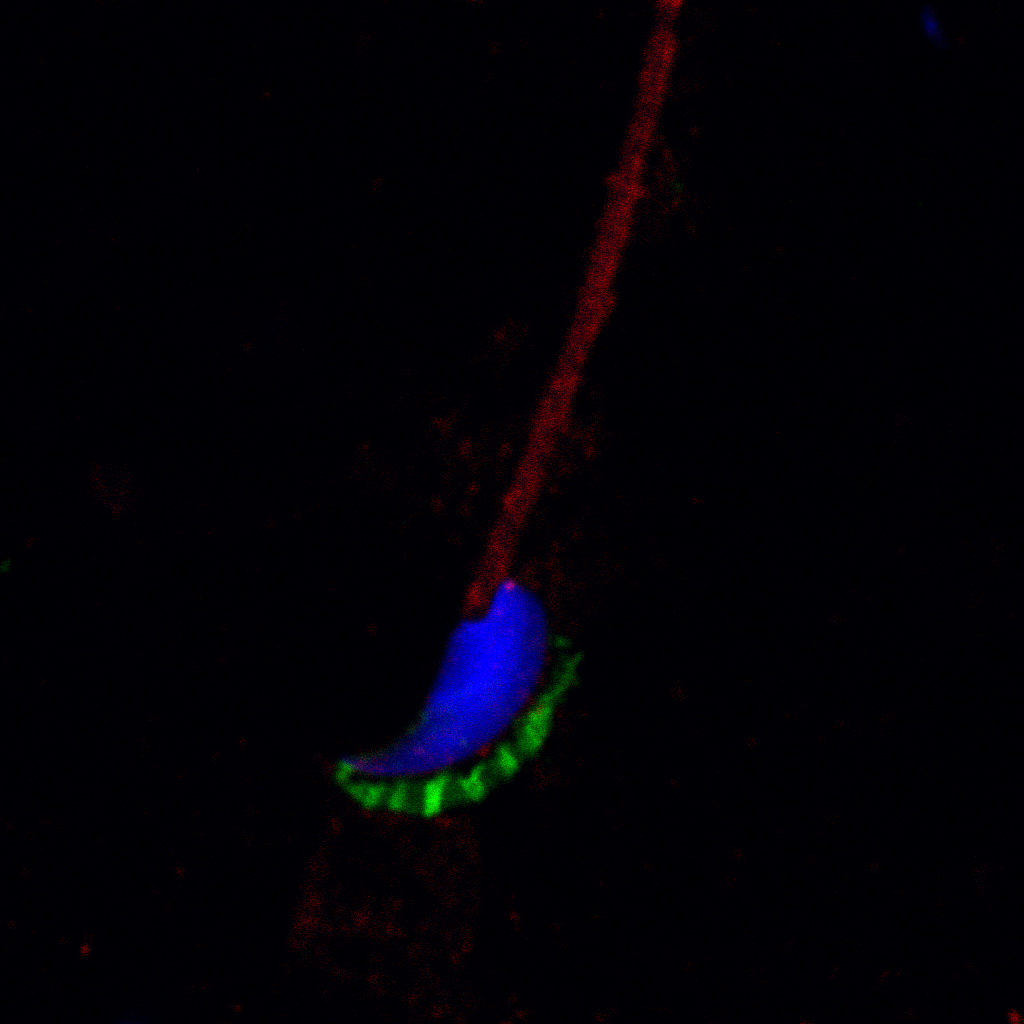

Supplement: Supplementary file 12 [file Data_Sheet_9.ZIP › Fig5A/wt/cfap53 pna m2 1 10.lif_Series009_z2.tif]

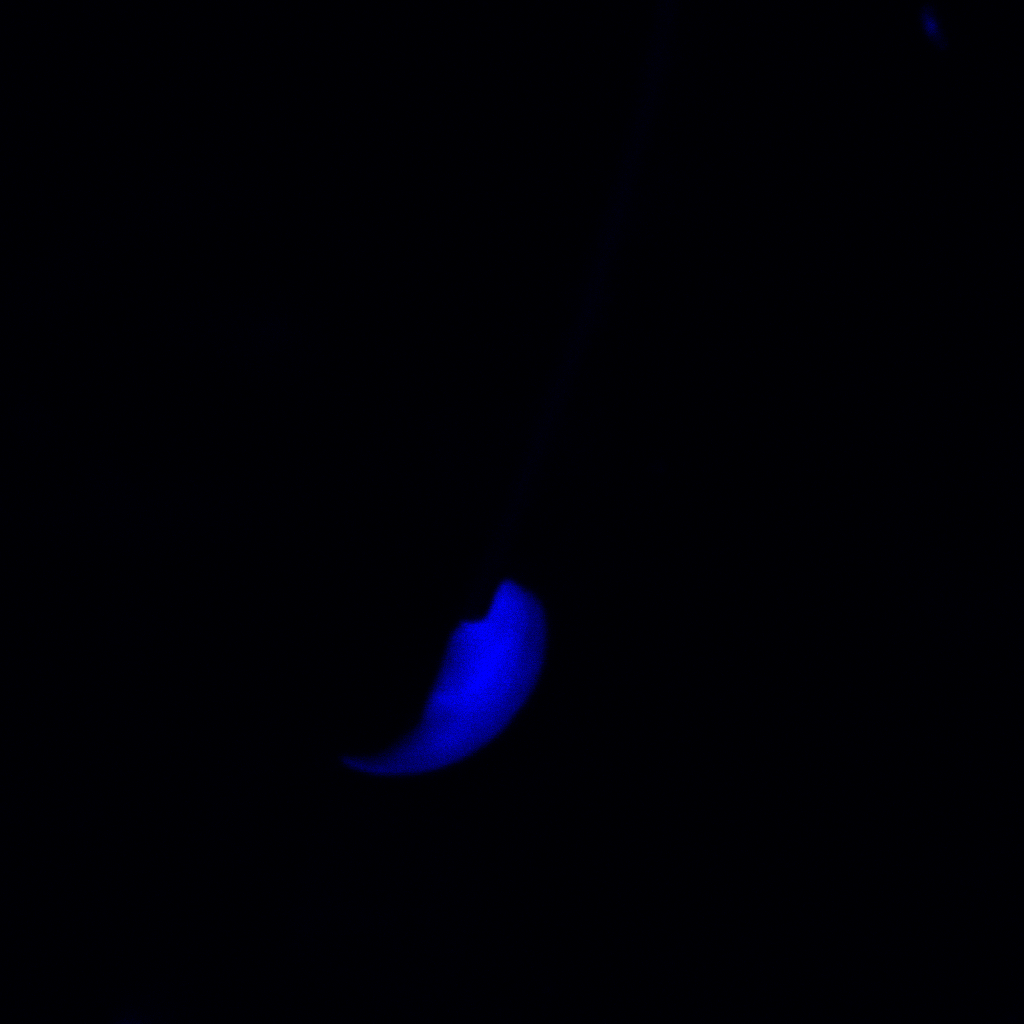

Supplement: Supplementary file 12 [file Data_Sheet_9.ZIP › Fig5A/wt/cfap53 pna m2 1 10.lif_Series009_z2_ch00.tif]

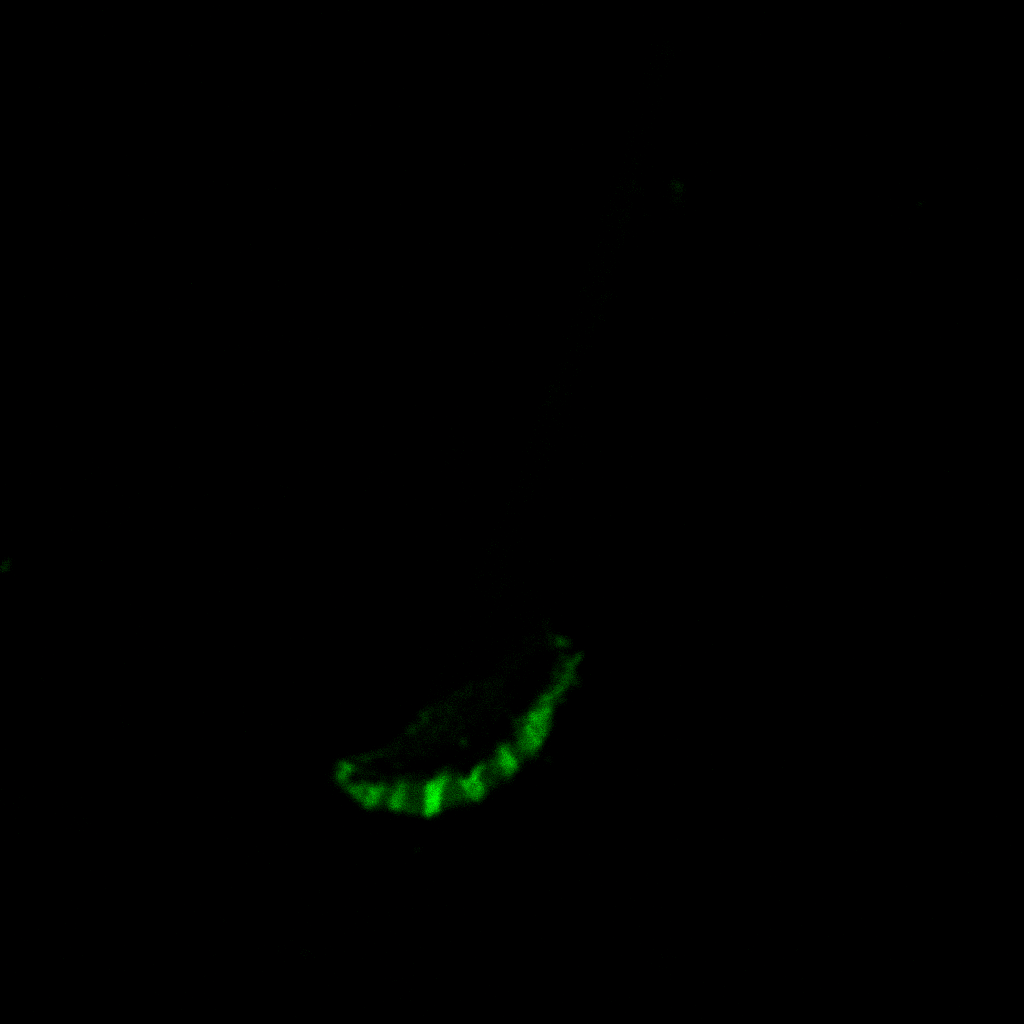

Supplement: Supplementary file 12 [file Data_Sheet_9.ZIP › Fig5A/wt/cfap53 pna m2 1 10.lif_Series009_z2_ch01.tif]

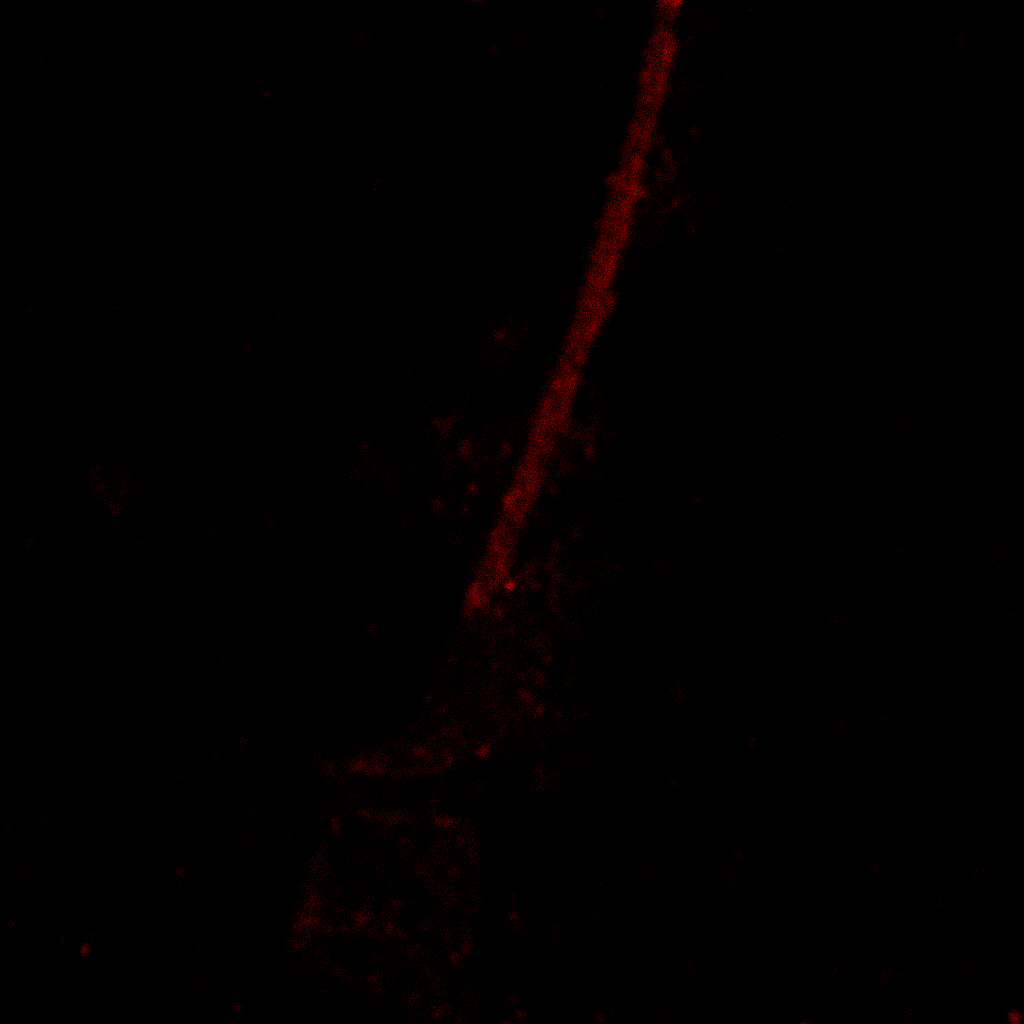

Supplement: Supplementary file 12 [file Data_Sheet_9.ZIP › Fig5A/wt/cfap53 pna m2 1 10.lif_Series009_z2_ch02.tif]

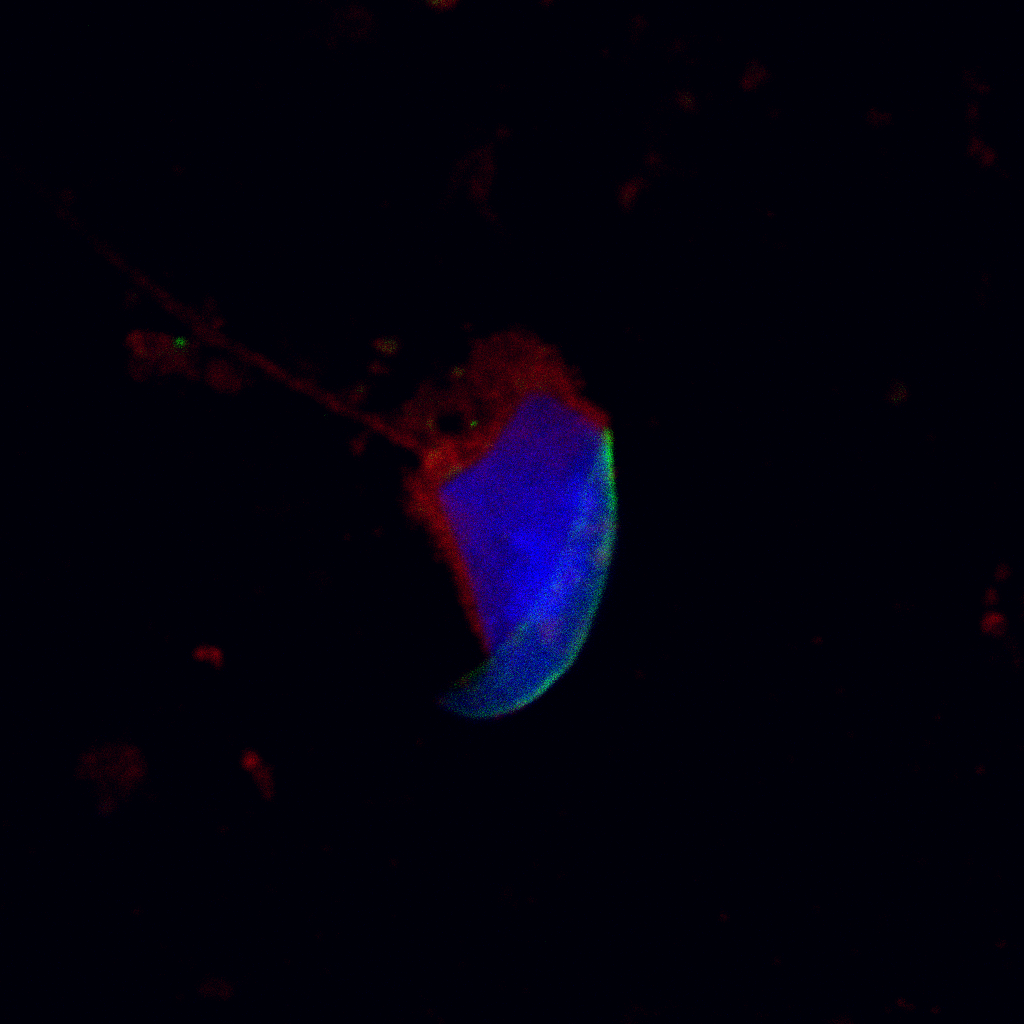

Supplement: Supplementary file 12 [file Data_Sheet_9.ZIP › Fig5A/wt/cfap53 tritc pna.lif_11-13_z3.tif]

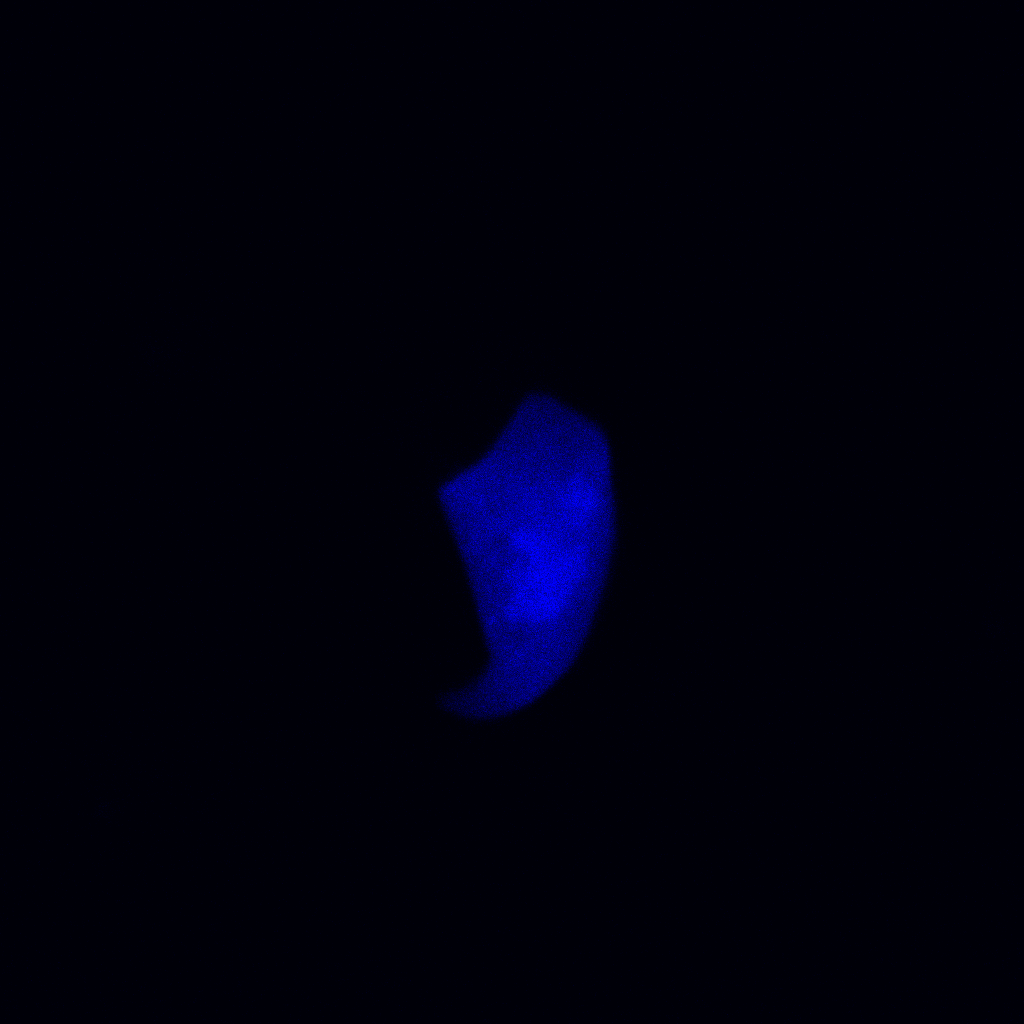

Supplement: Supplementary file 12 [file Data_Sheet_9.ZIP › Fig5A/wt/cfap53 tritc pna.lif_11-13_z3_ch00.tif]

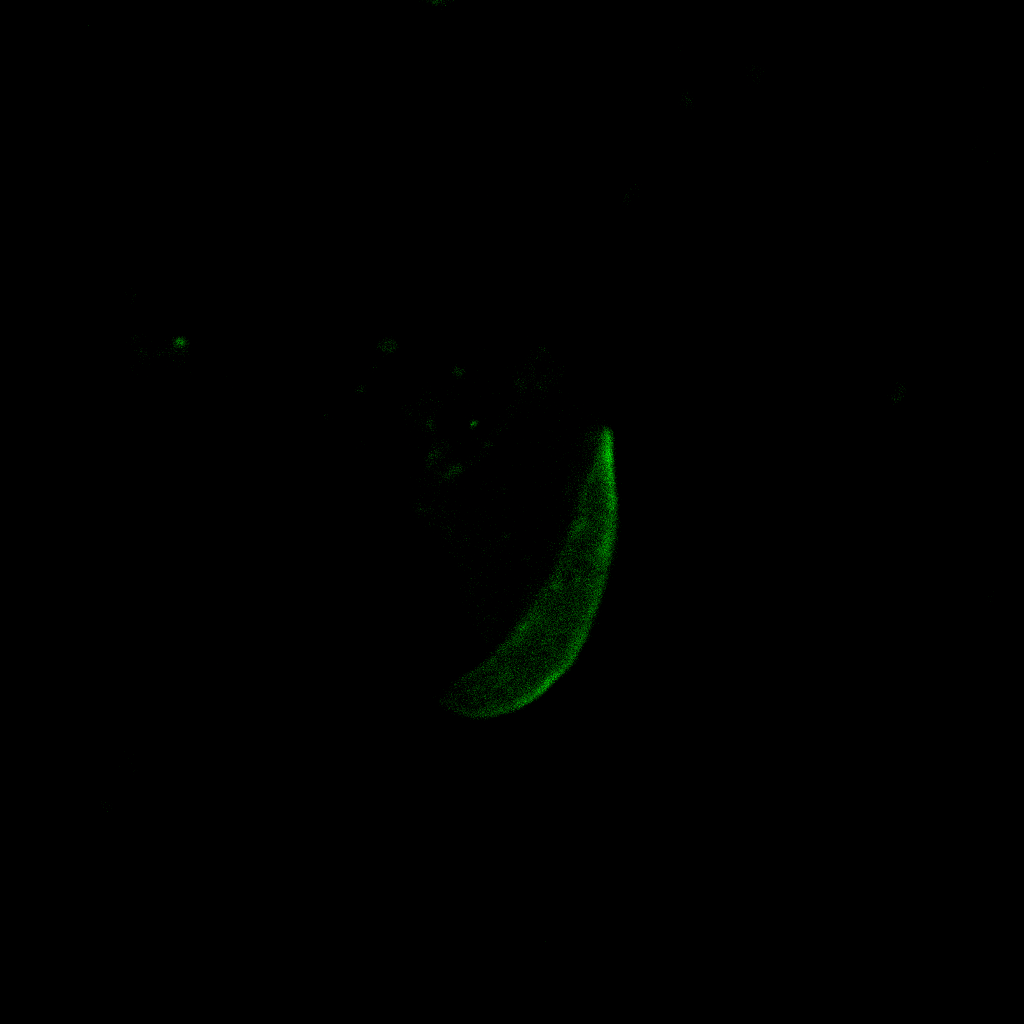

Supplement: Supplementary file 12 [file Data_Sheet_9.ZIP › Fig5A/wt/cfap53 tritc pna.lif_11-13_z3_ch01.tif]

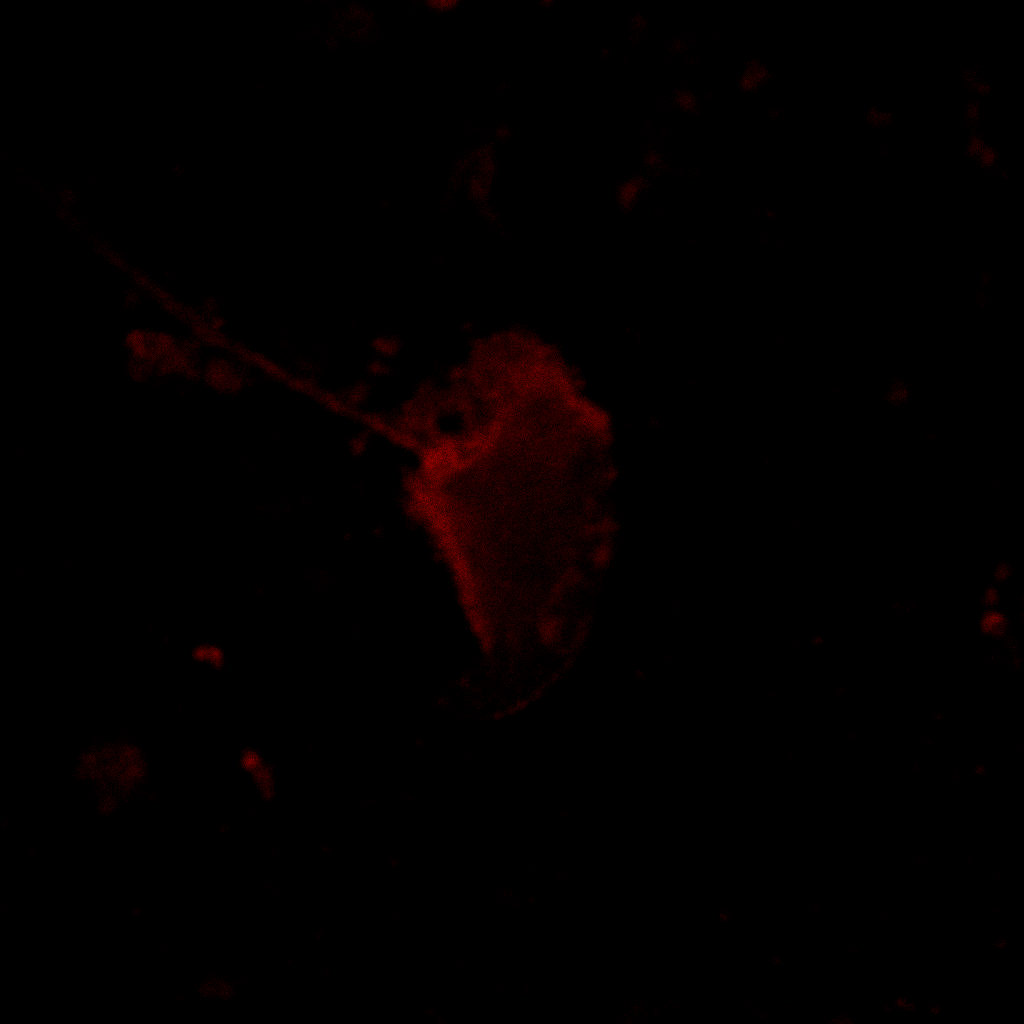

Supplement: Supplementary file 12 [file Data_Sheet_9.ZIP › Fig5A/wt/cfap53 tritc pna.lif_11-13_z3_ch02.tif]

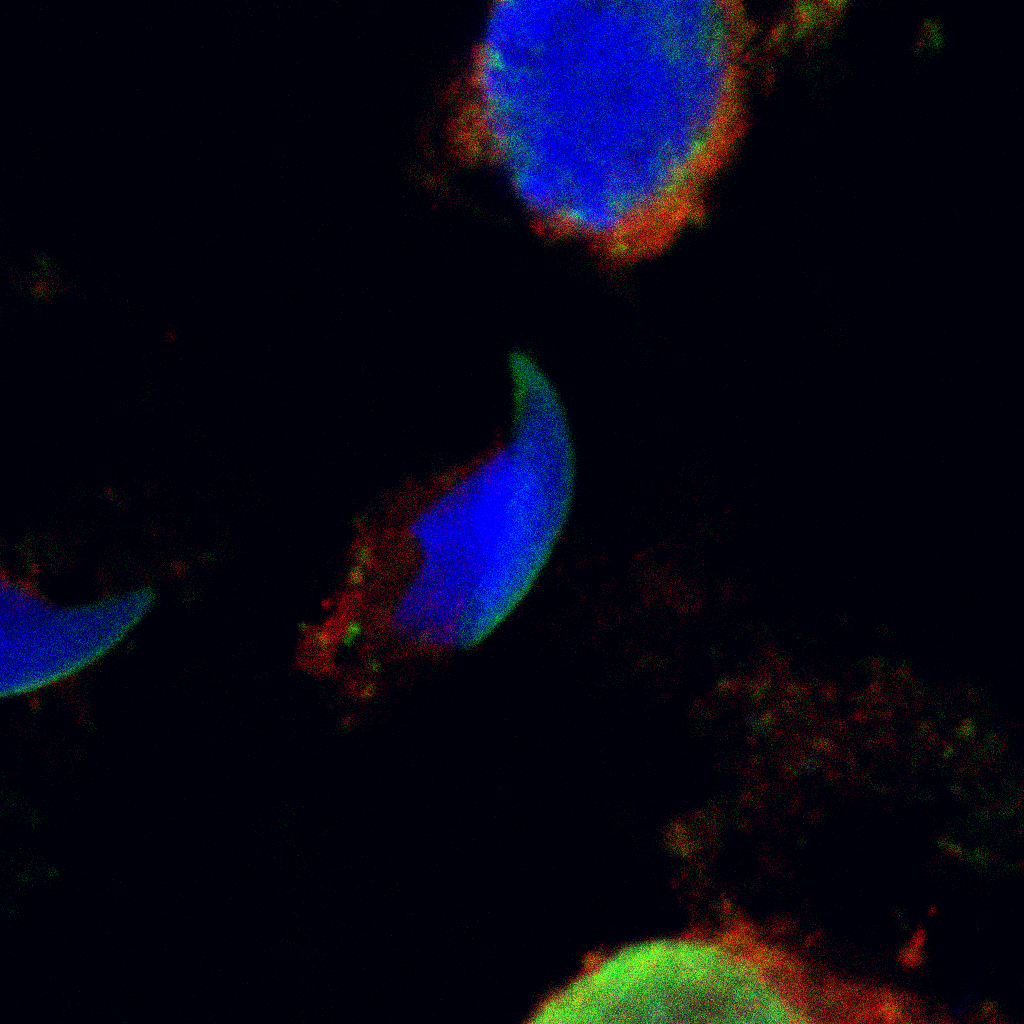

Supplement: Supplementary file 12 [file Data_Sheet_9.ZIP › Fig5A/wt/cfap53 tritc pna.lif_13-14_z2.tif]

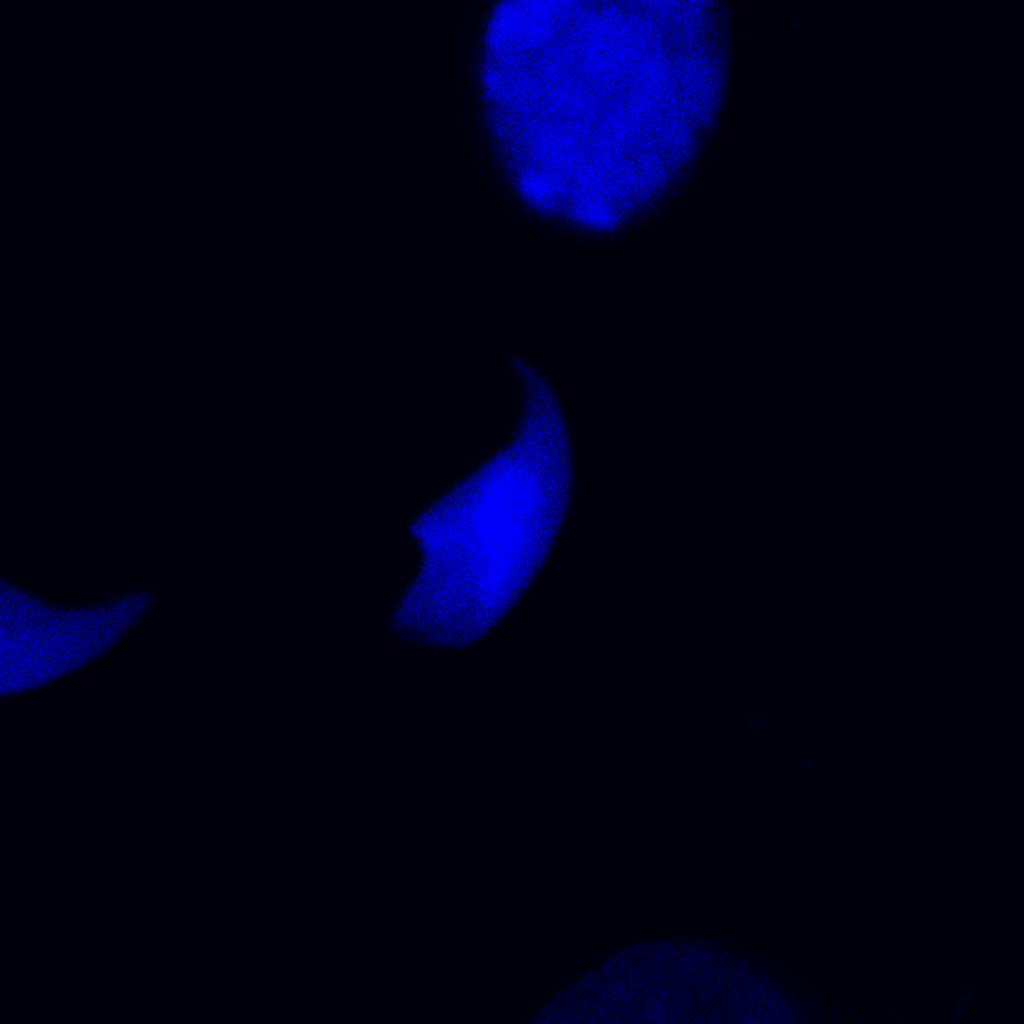

Supplement: Supplementary file 12 [file Data_Sheet_9.ZIP › Fig5A/wt/cfap53 tritc pna.lif_13-14_z2_ch00.tif]

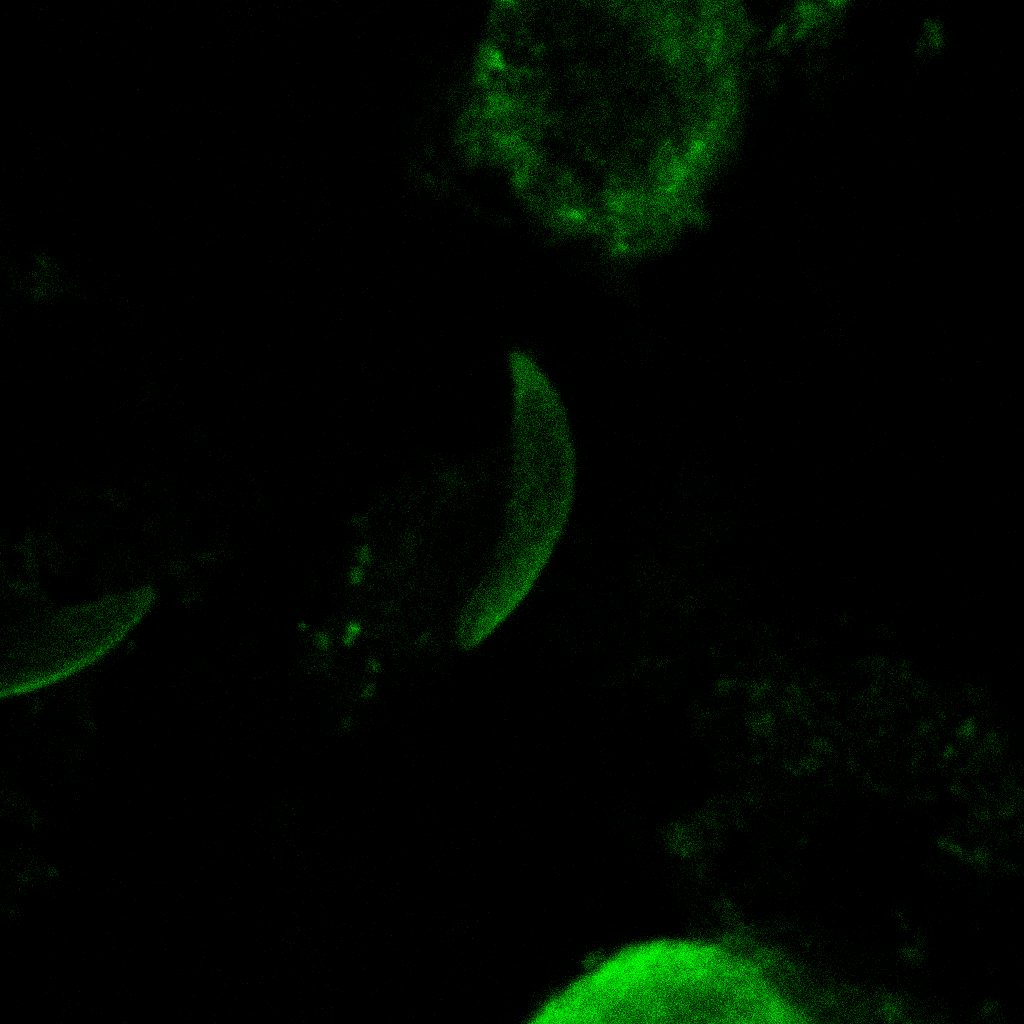

Supplement: Supplementary file 12 [file Data_Sheet_9.ZIP › Fig5A/wt/cfap53 tritc pna.lif_13-14_z2_ch01.tif]

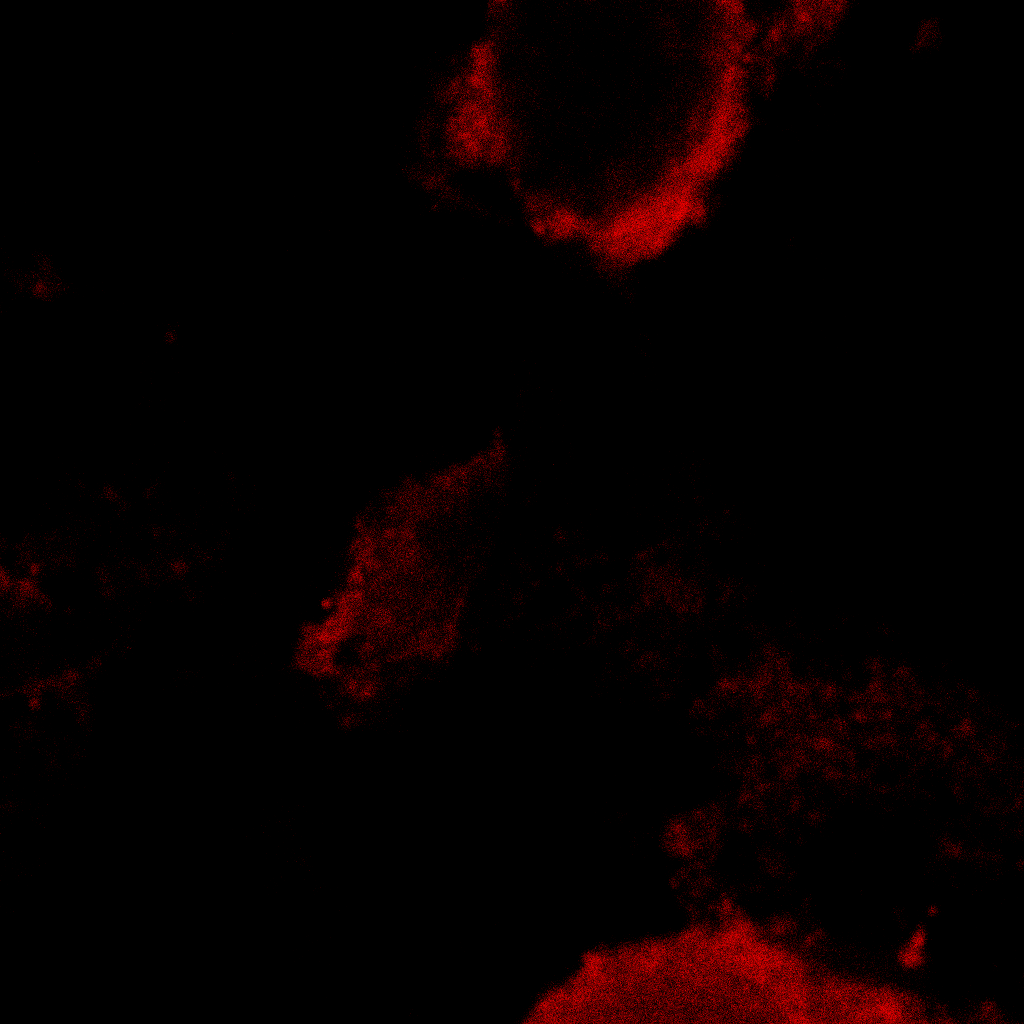

Supplement: Supplementary file 12 [file Data_Sheet_9.ZIP › Fig5A/wt/cfap53 tritc pna.lif_13-14_z2_ch02.tif]

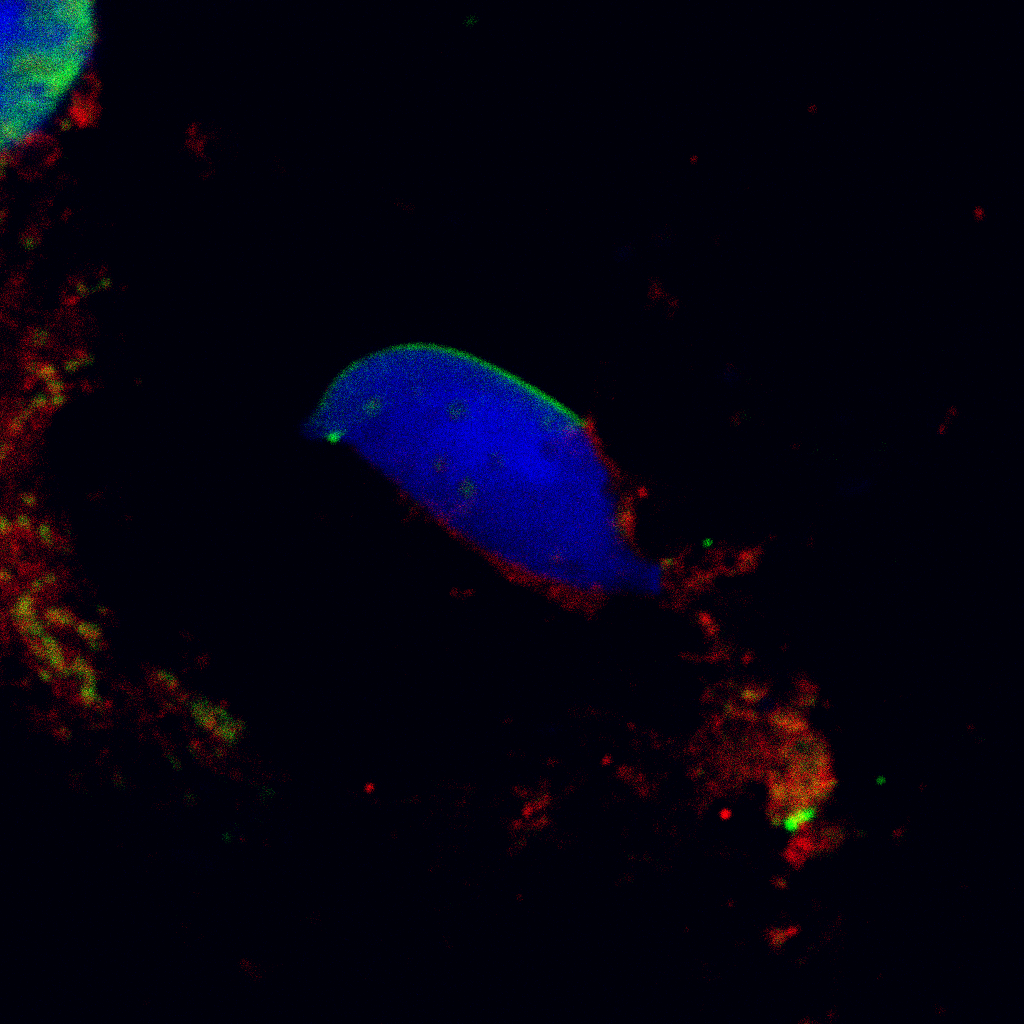

Supplement: Supplementary file 12 [file Data_Sheet_9.ZIP › Fig5A/wt/cfap53 tritc pna.lif_9-11_z3.tif]

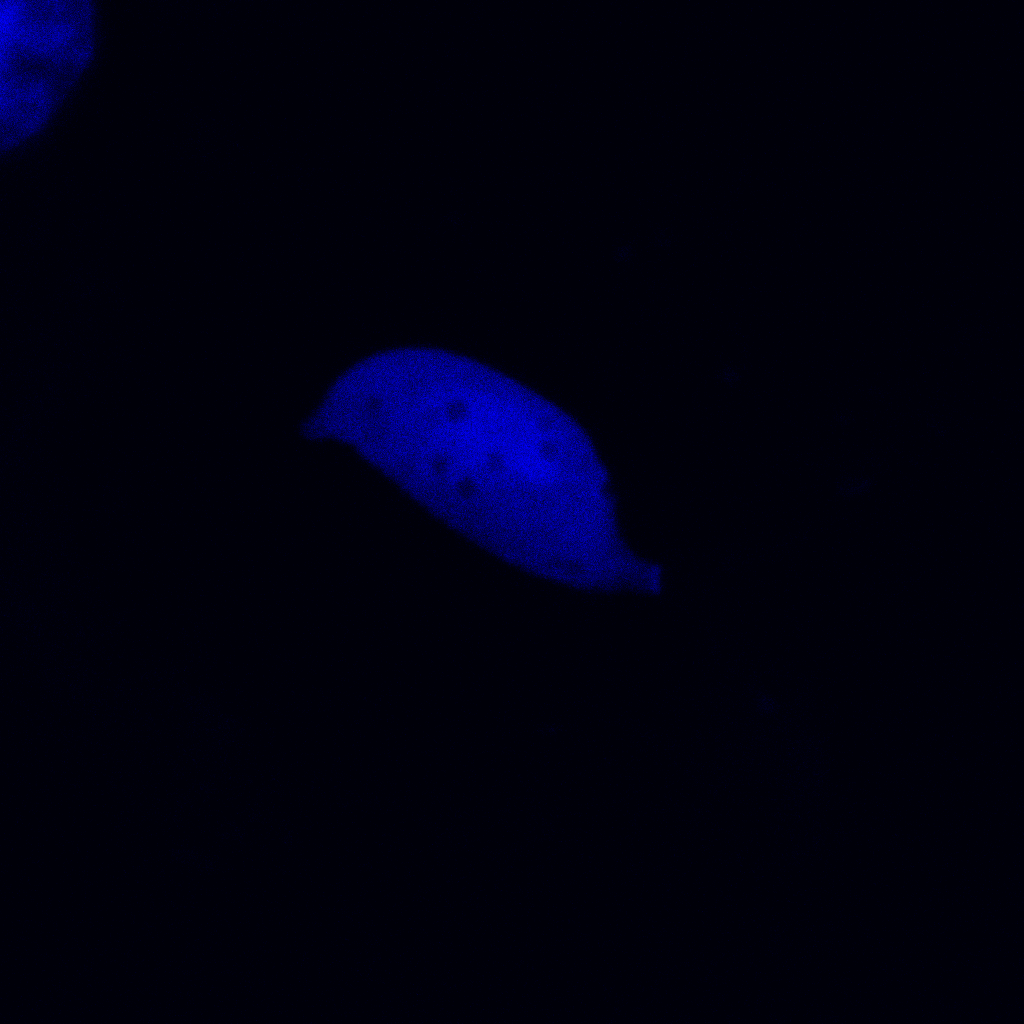

Supplement: Supplementary file 12 [file Data_Sheet_9.ZIP › Fig5A/wt/cfap53 tritc pna.lif_9-11_z3_ch00.tif]

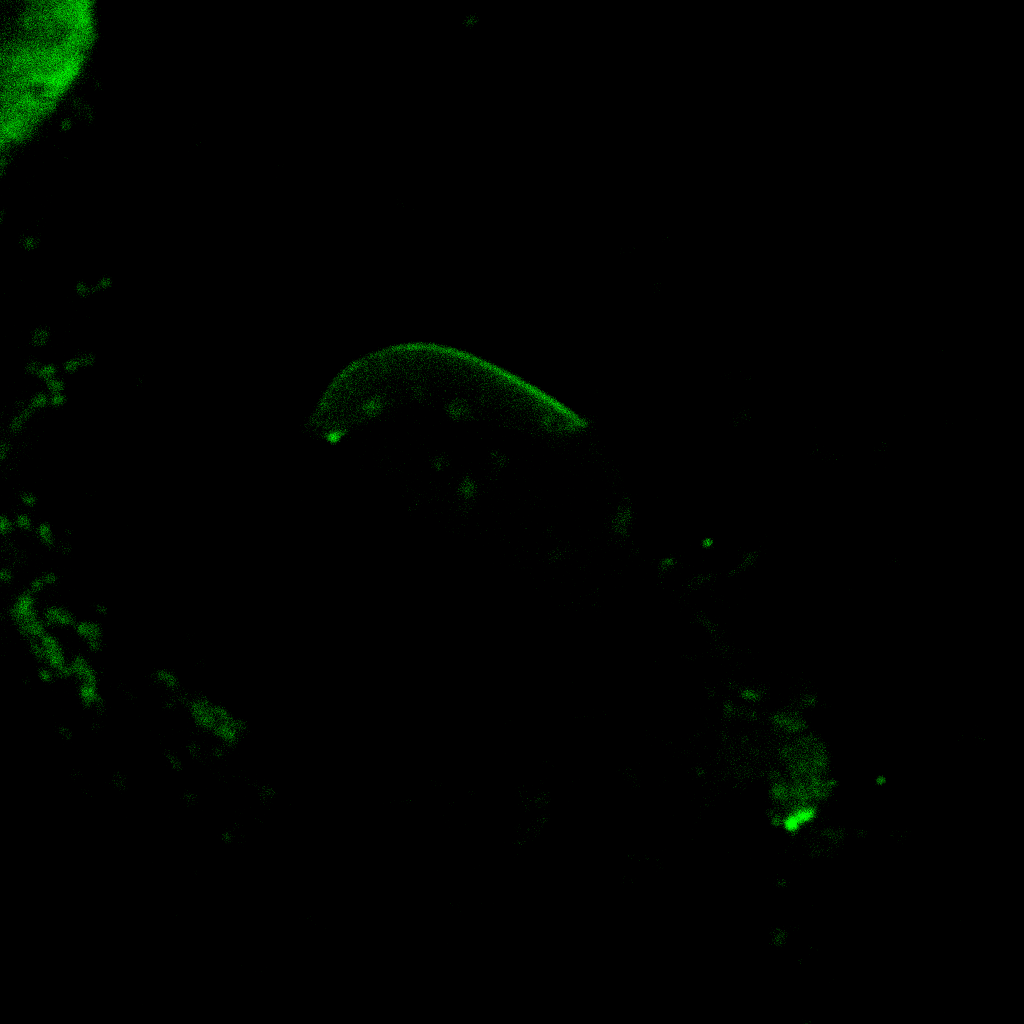

Supplement: Supplementary file 12 [file Data_Sheet_9.ZIP › Fig5A/wt/cfap53 tritc pna.lif_9-11_z3_ch01.tif]

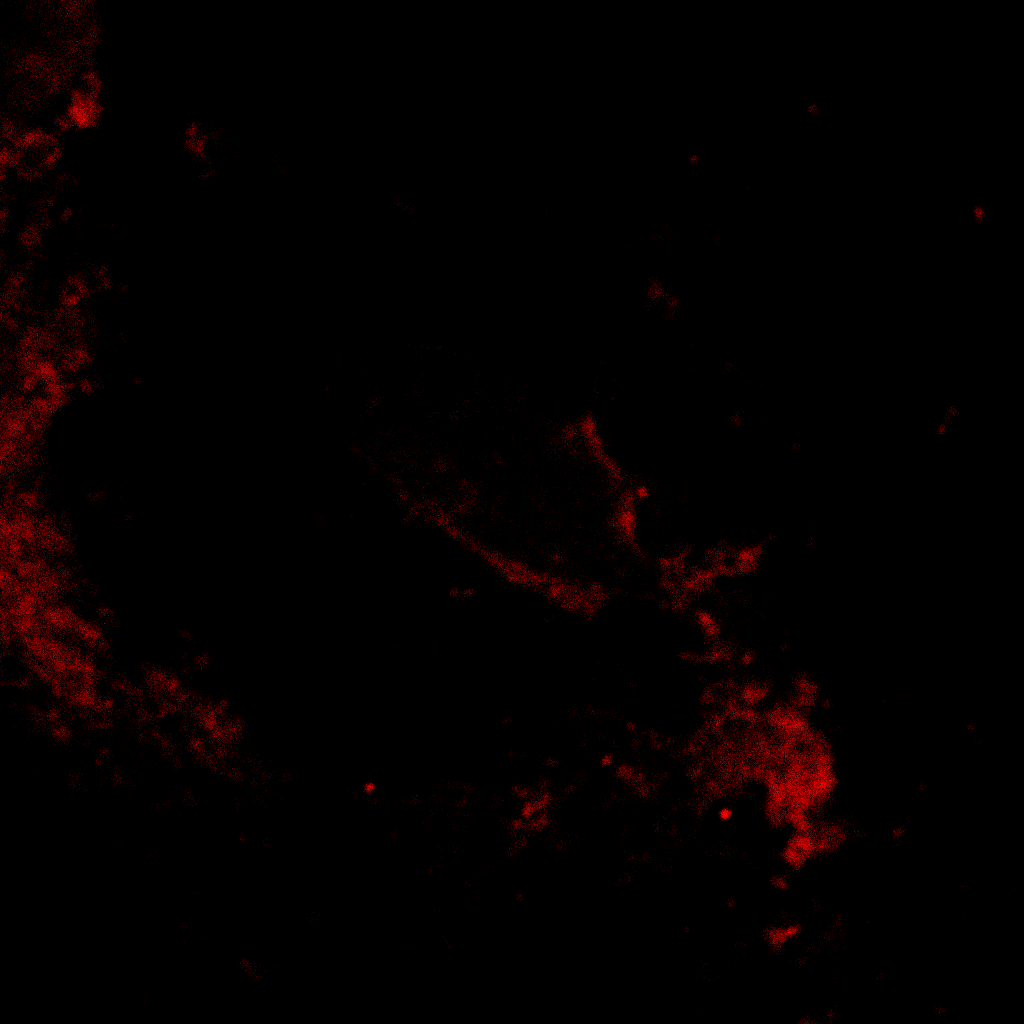

Supplement: Supplementary file 12 [file Data_Sheet_9.ZIP › Fig5A/wt/cfap53 tritc pna.lif_9-11_z3_ch02.tif]

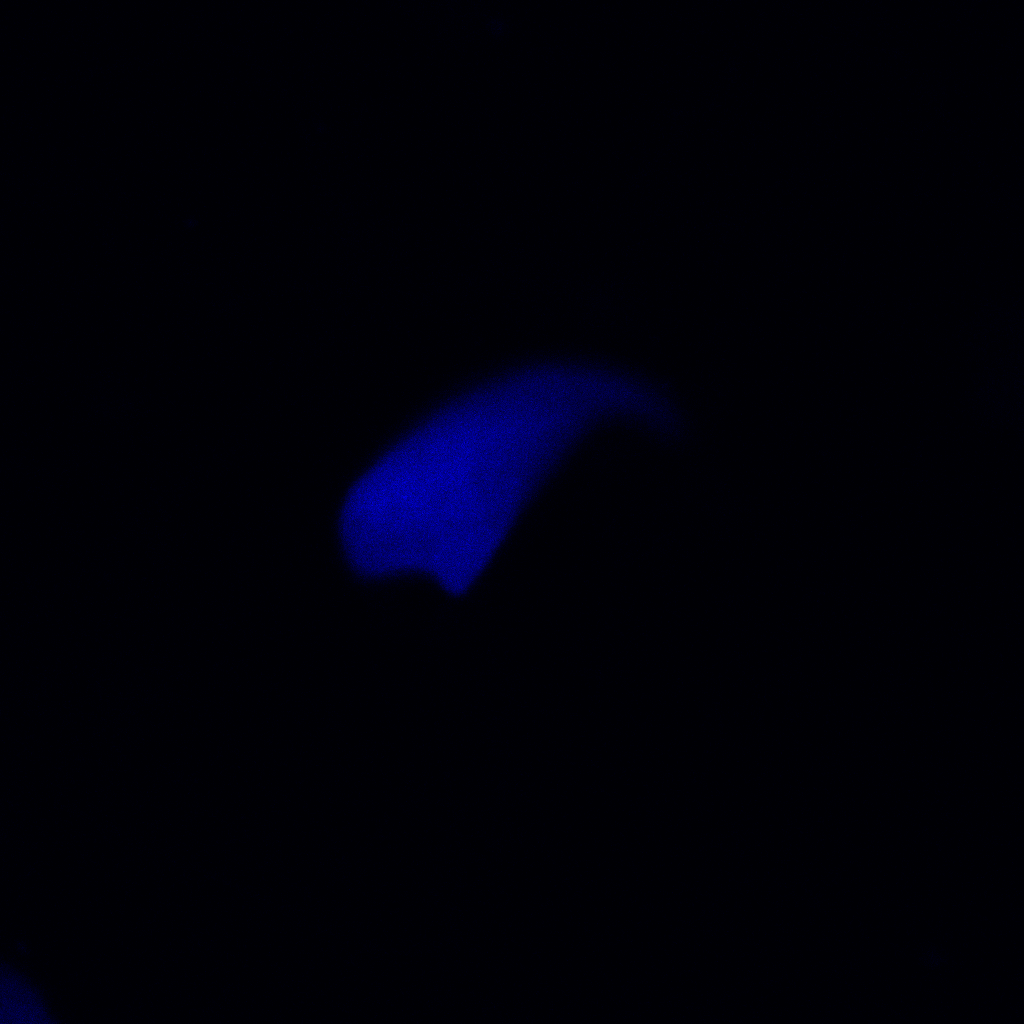

Supplement: Supplementary file 13 [file Data_Sheet_10.ZIP › Fig5B/cfap53 tritc a tubulin fitc.lif_11-12_z4_ch00.tif]

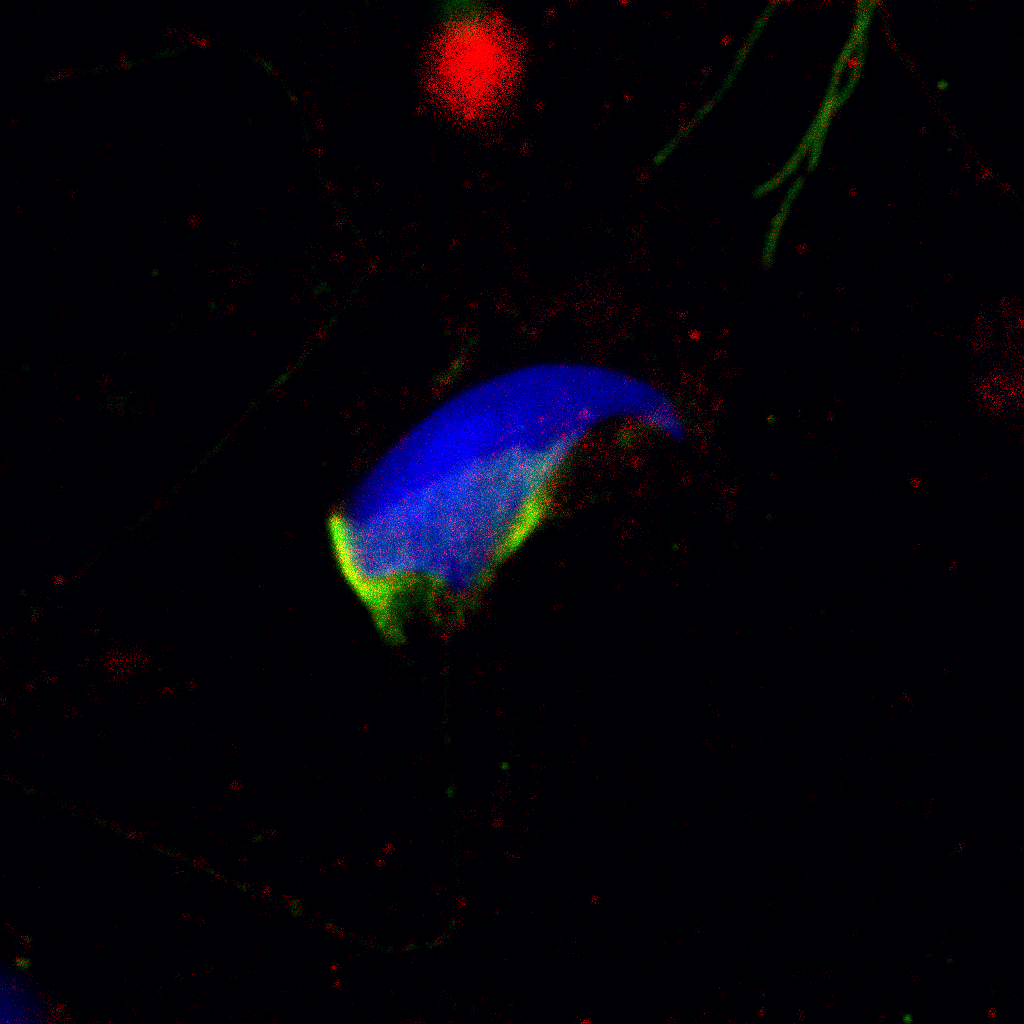

Supplement: Supplementary file 13 [file Data_Sheet_10.ZIP › Fig5B/cfap53 tritc a tubulin fitc.lif_11-12_z4_ch01 merge.tif]

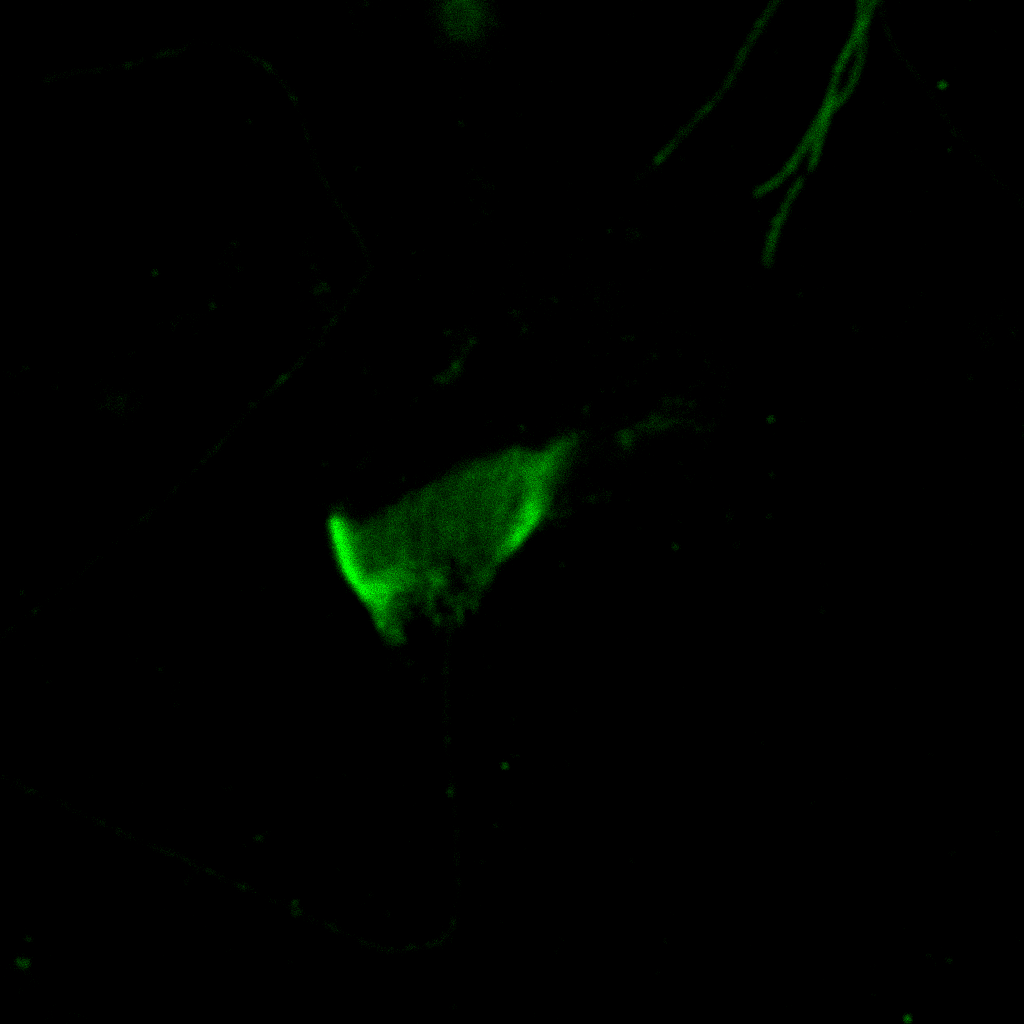

Supplement: Supplementary file 13 [file Data_Sheet_10.ZIP › Fig5B/cfap53 tritc a tubulin fitc.lif_11-12_z4_ch01.tif]

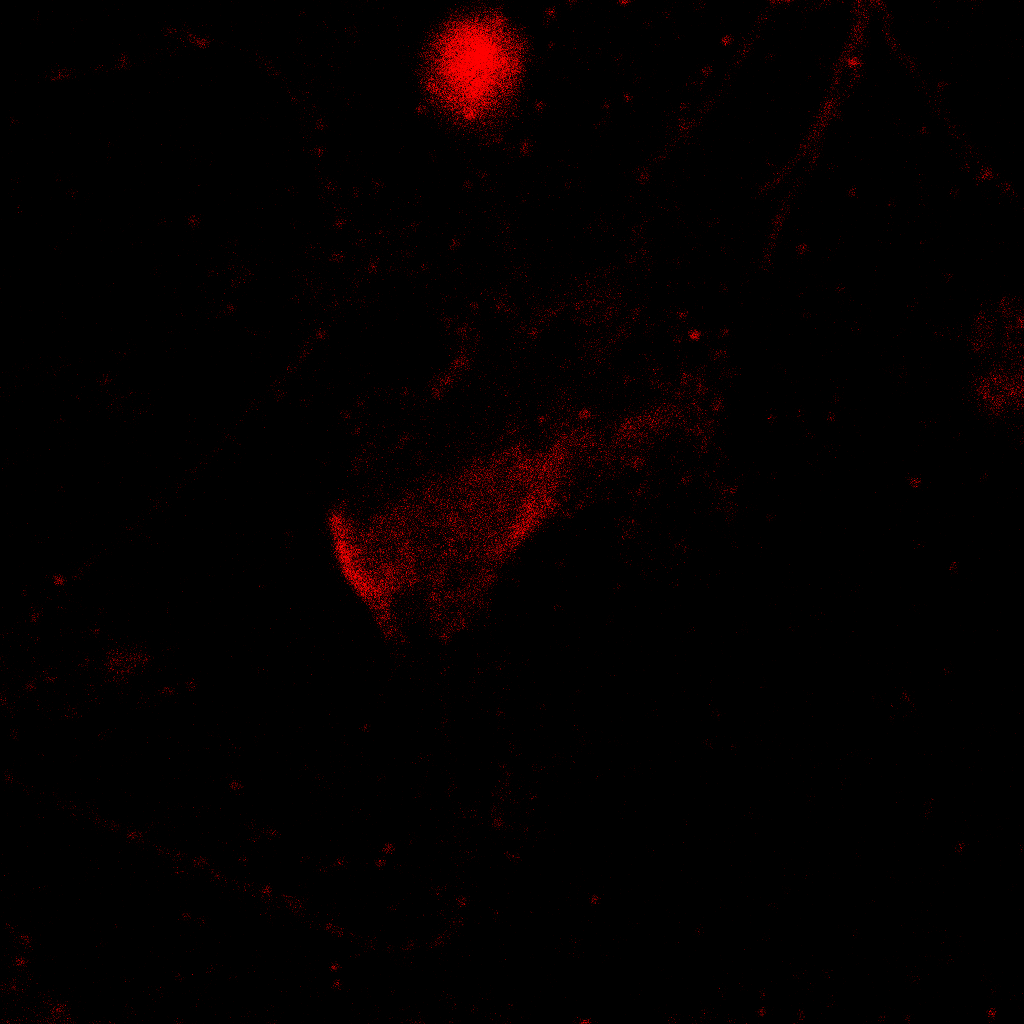

Supplement: Supplementary file 13 [file Data_Sheet_10.ZIP › Fig5B/cfap53 tritc a tubulin fitc.lif_11-12_z4_ch02.tif]

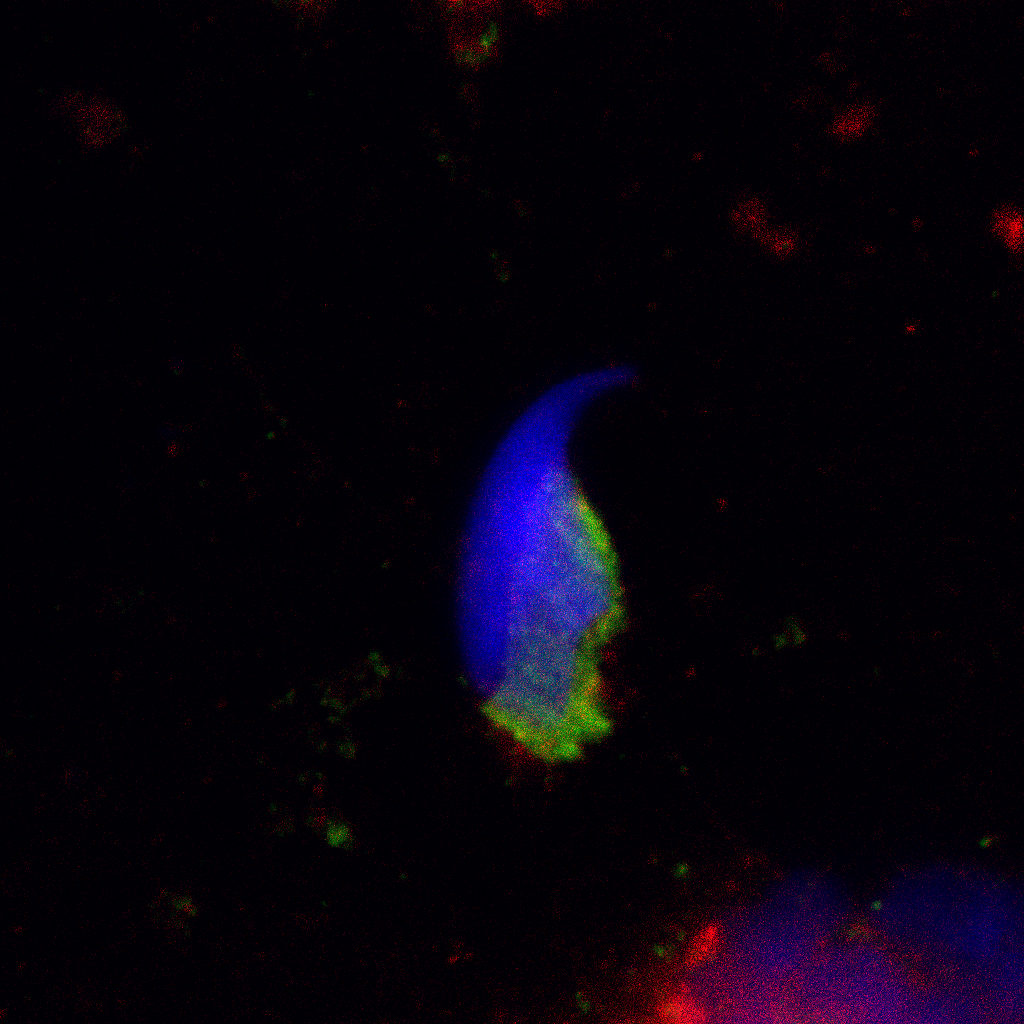

Supplement: Supplementary file 13 [file Data_Sheet_10.ZIP › Fig5B/cfap53 tritc a tubulin fitc.lif_13-14 _Processed001.tif]

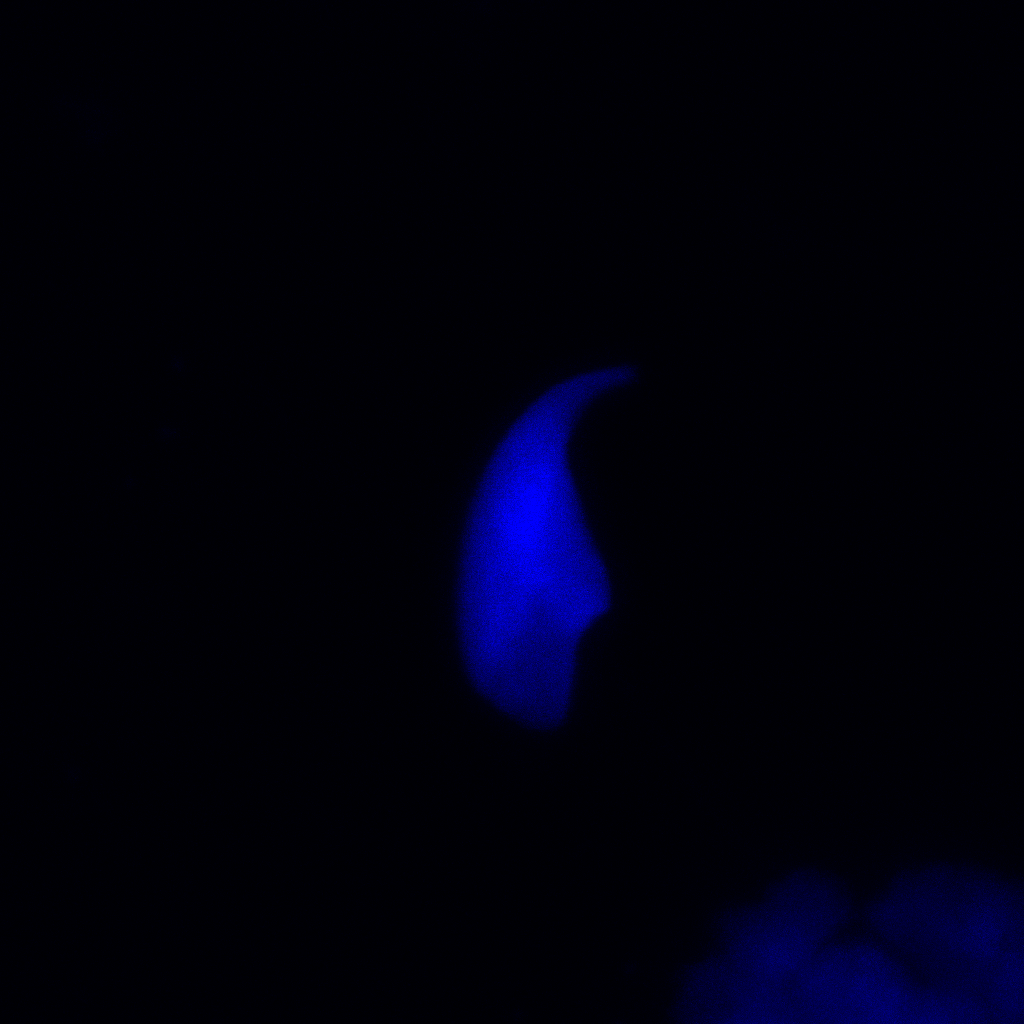

Supplement: Supplementary file 13 [file Data_Sheet_10.ZIP › Fig5B/cfap53 tritc a tubulin fitc.lif_13-14 _Processed001_ch00.tif]

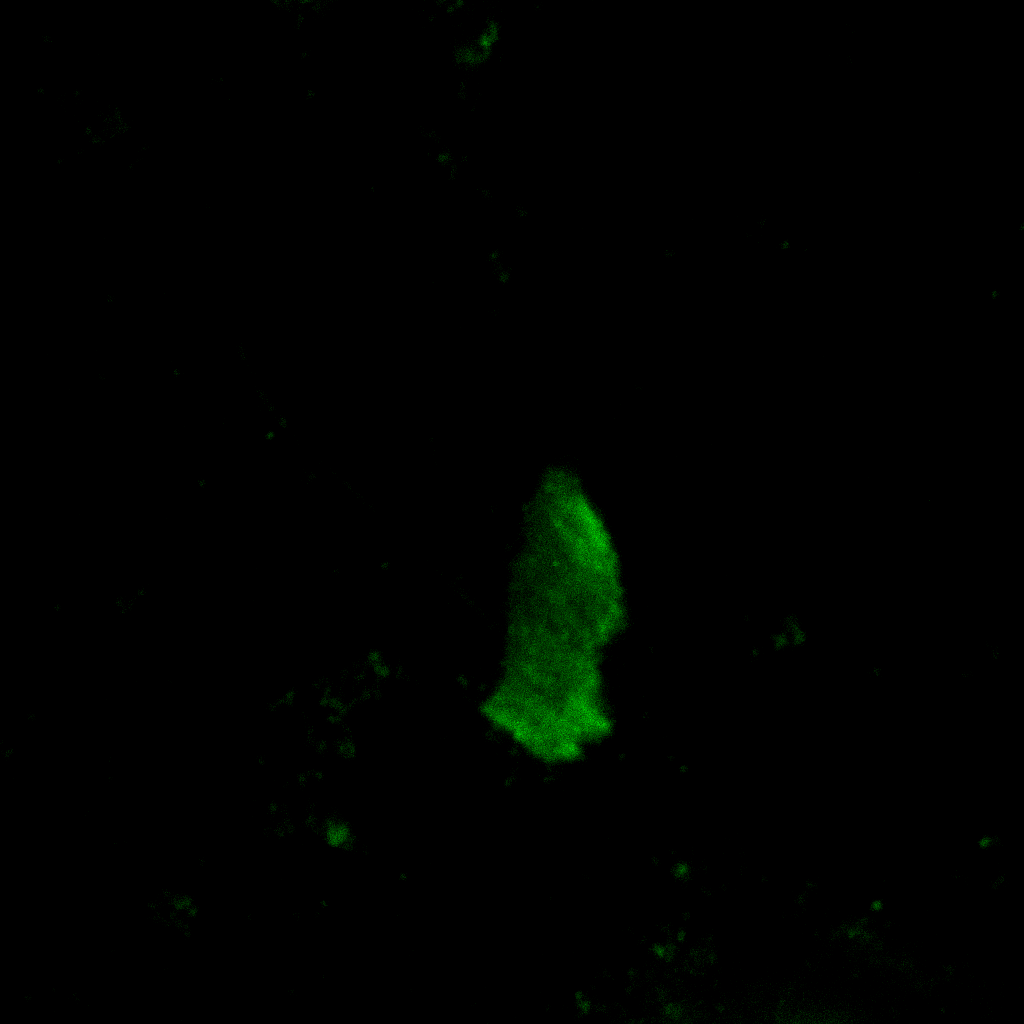

Supplement: Supplementary file 13 [file Data_Sheet_10.ZIP › Fig5B/cfap53 tritc a tubulin fitc.lif_13-14 _Processed001_ch01.tif]

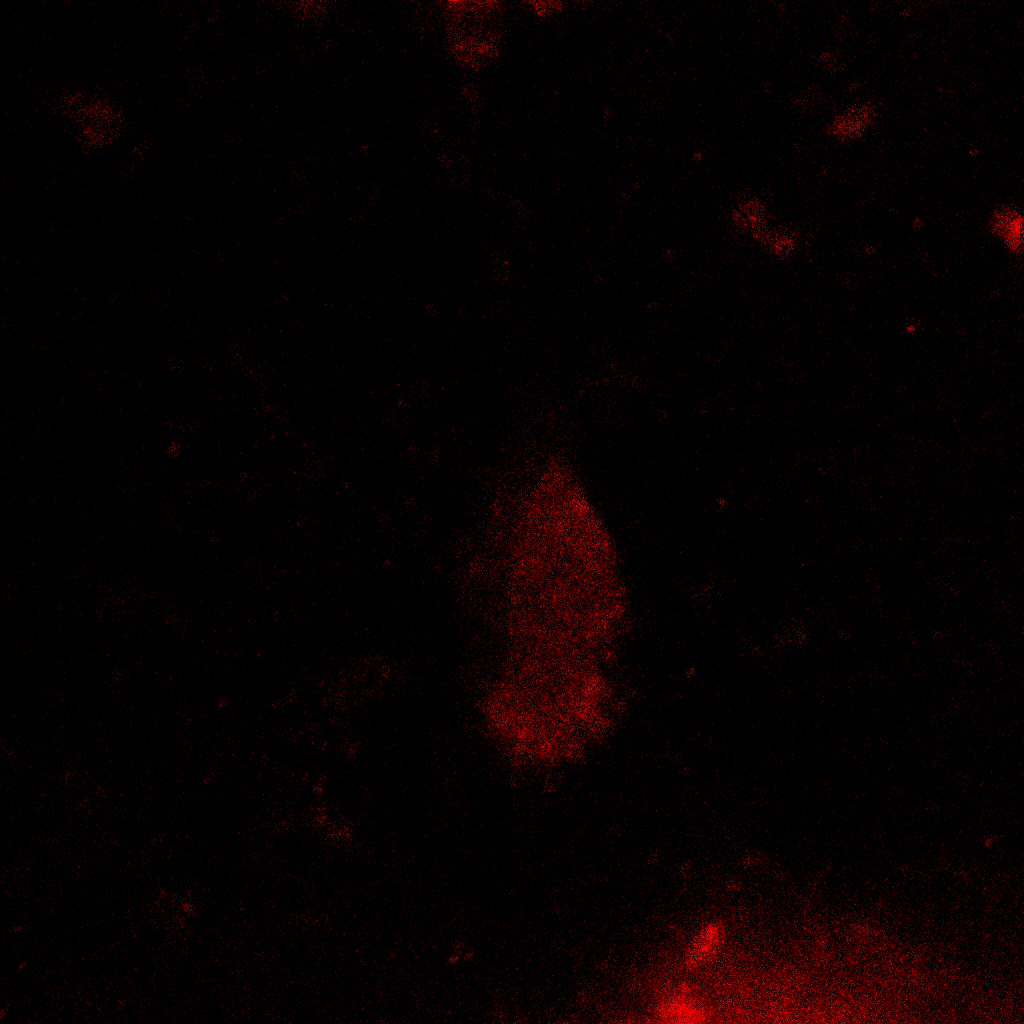

Supplement: Supplementary file 13 [file Data_Sheet_10.ZIP › Fig5B/cfap53 tritc a tubulin fitc.lif_13-14 _Processed001_ch02.tif]

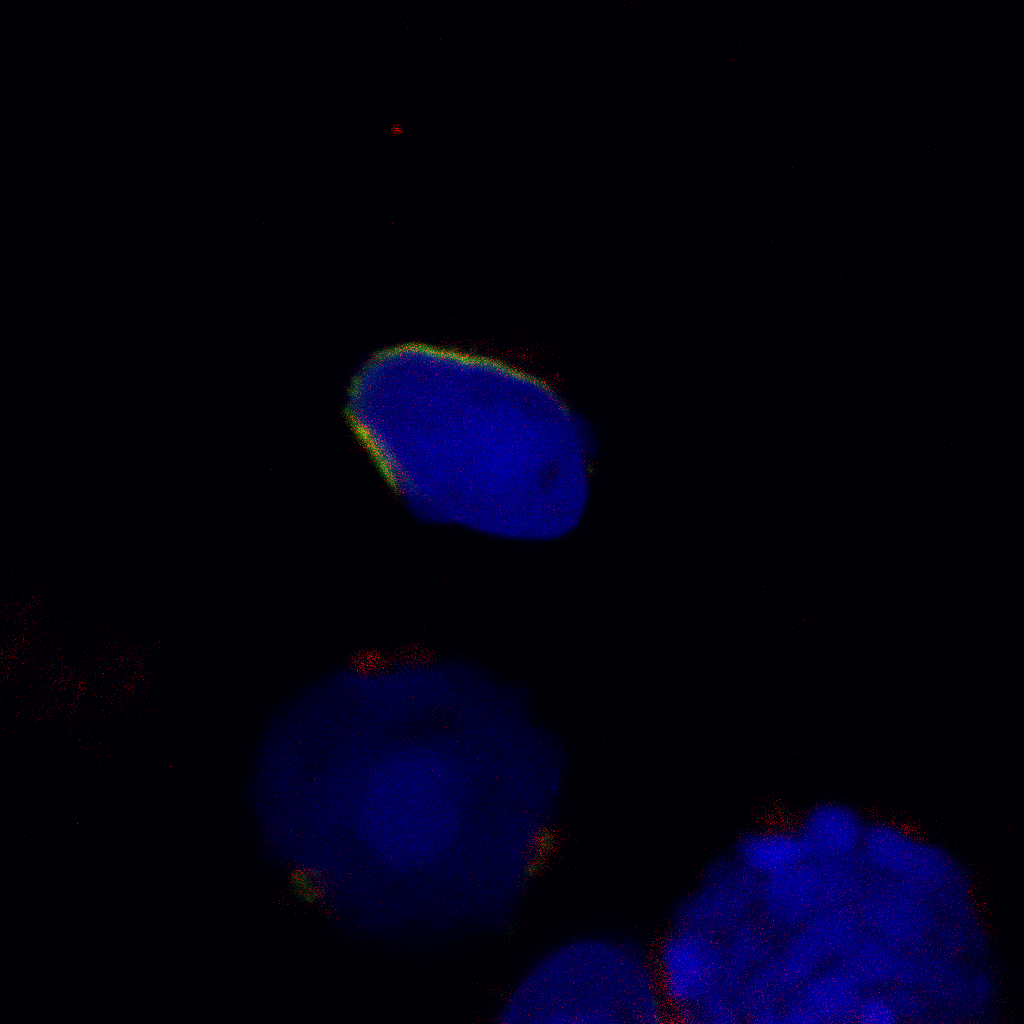

Supplement: Supplementary file 13 [file Data_Sheet_10.ZIP › Fig5B/cfap53 tritc a tubulin fitc.lif_9_z2.tif]

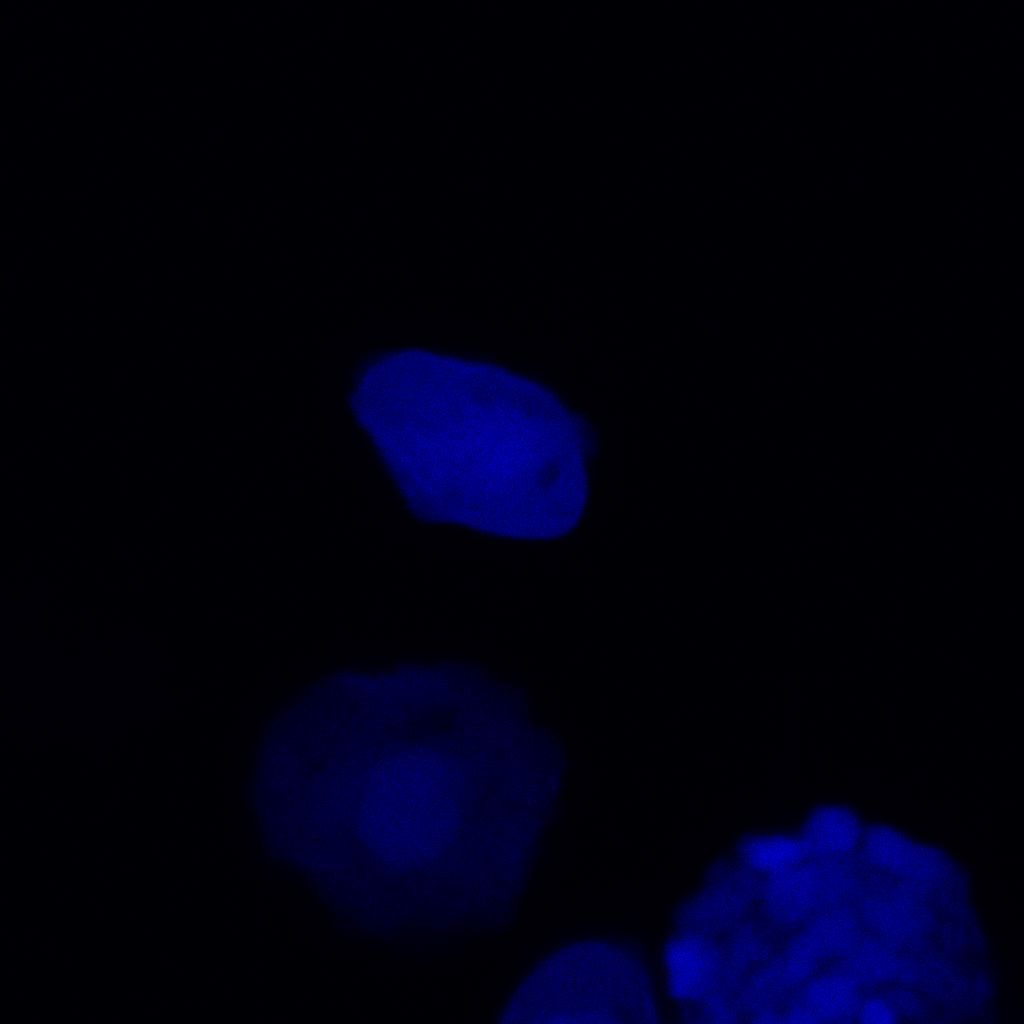

Supplement: Supplementary file 13 [file Data_Sheet_10.ZIP › Fig5B/cfap53 tritc a tubulin fitc.lif_9_z2_ch00.tif]

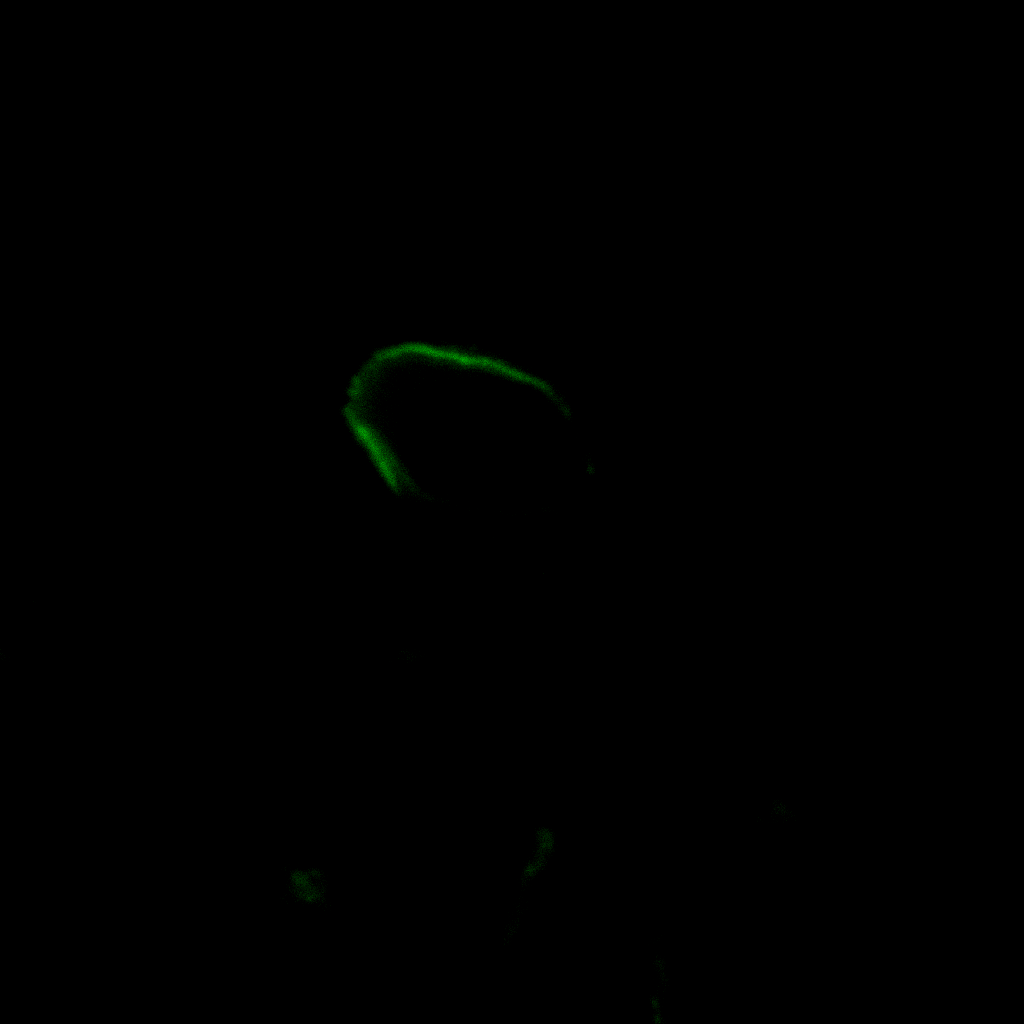

Supplement: Supplementary file 13 [file Data_Sheet_10.ZIP › Fig5B/cfap53 tritc a tubulin fitc.lif_9_z2_ch01.tif]

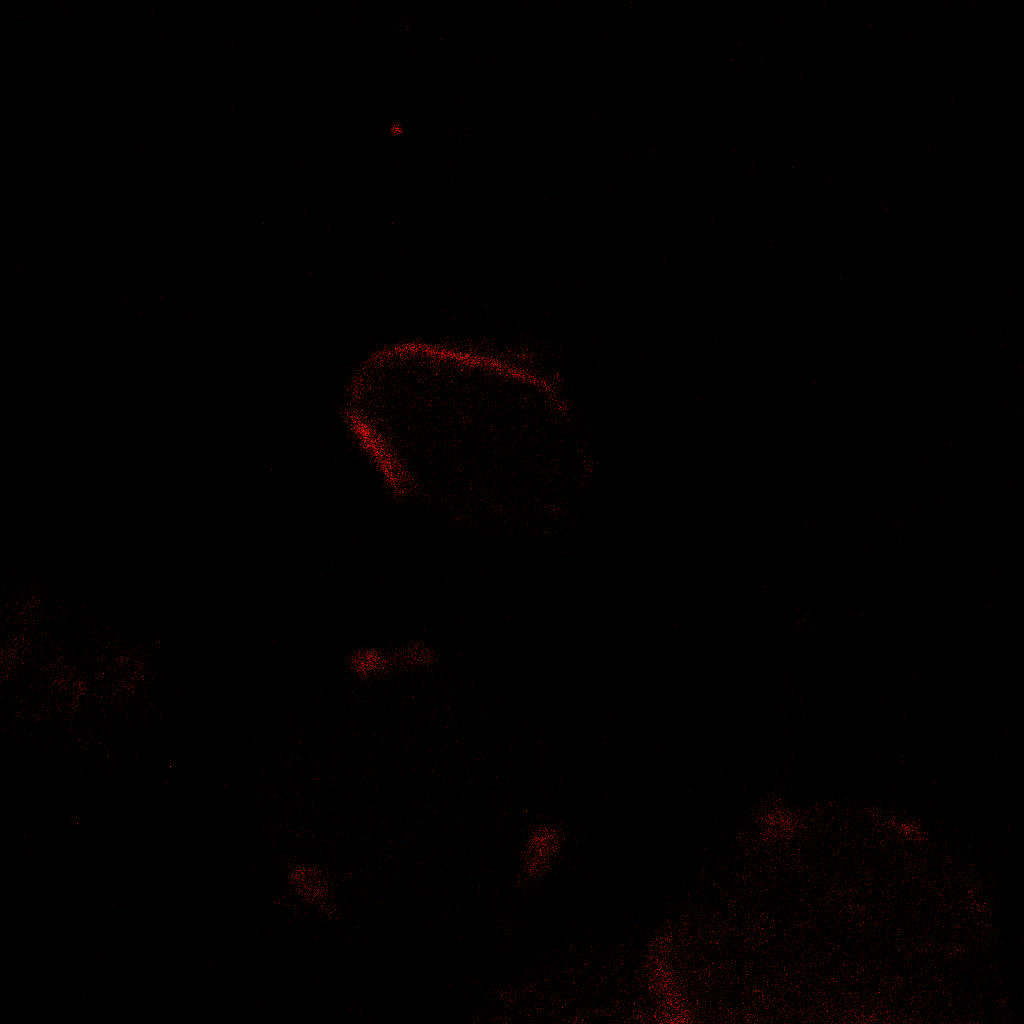

Supplement: Supplementary file 13 [file Data_Sheet_10.ZIP › Fig5B/cfap53 tritc a tubulin fitc.lif_9_z2_ch02.tif]

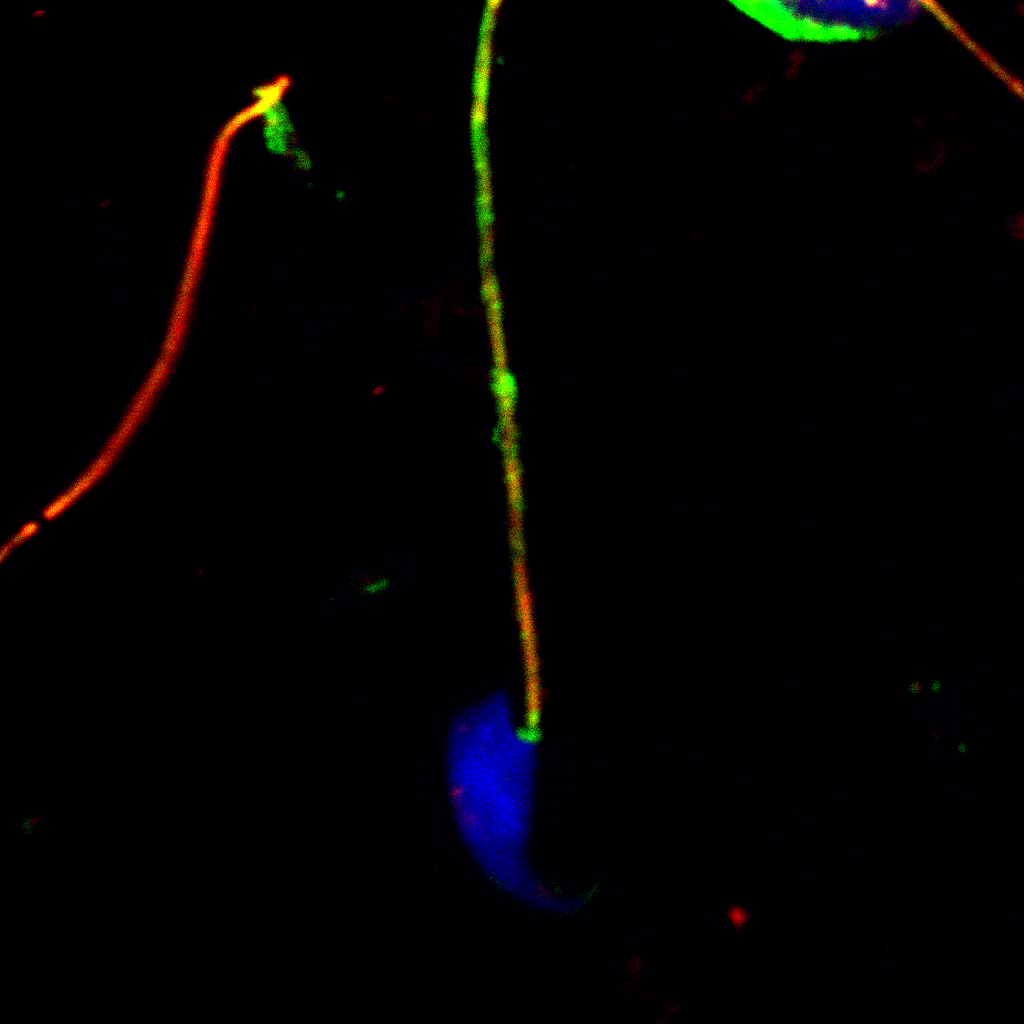

Supplement: Supplementary file 13 [file Data_Sheet_10.ZIP › Fig5B/cfap53 WT M5 10 tritc tub fitc.lif_Series001_z1.tif]

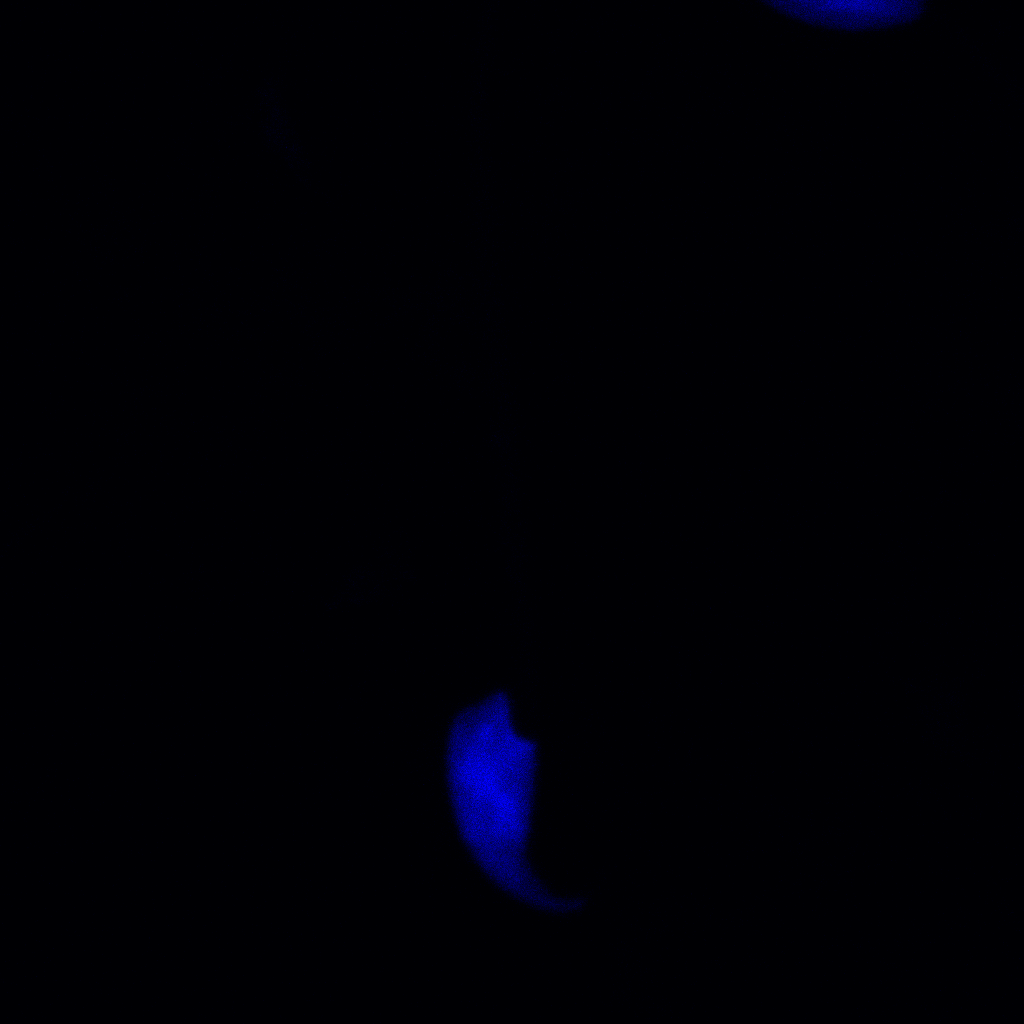

Supplement: Supplementary file 13 [file Data_Sheet_10.ZIP › Fig5B/cfap53 WT M5 10 tritc tub fitc.lif_Series001_z1_ch00.tif]

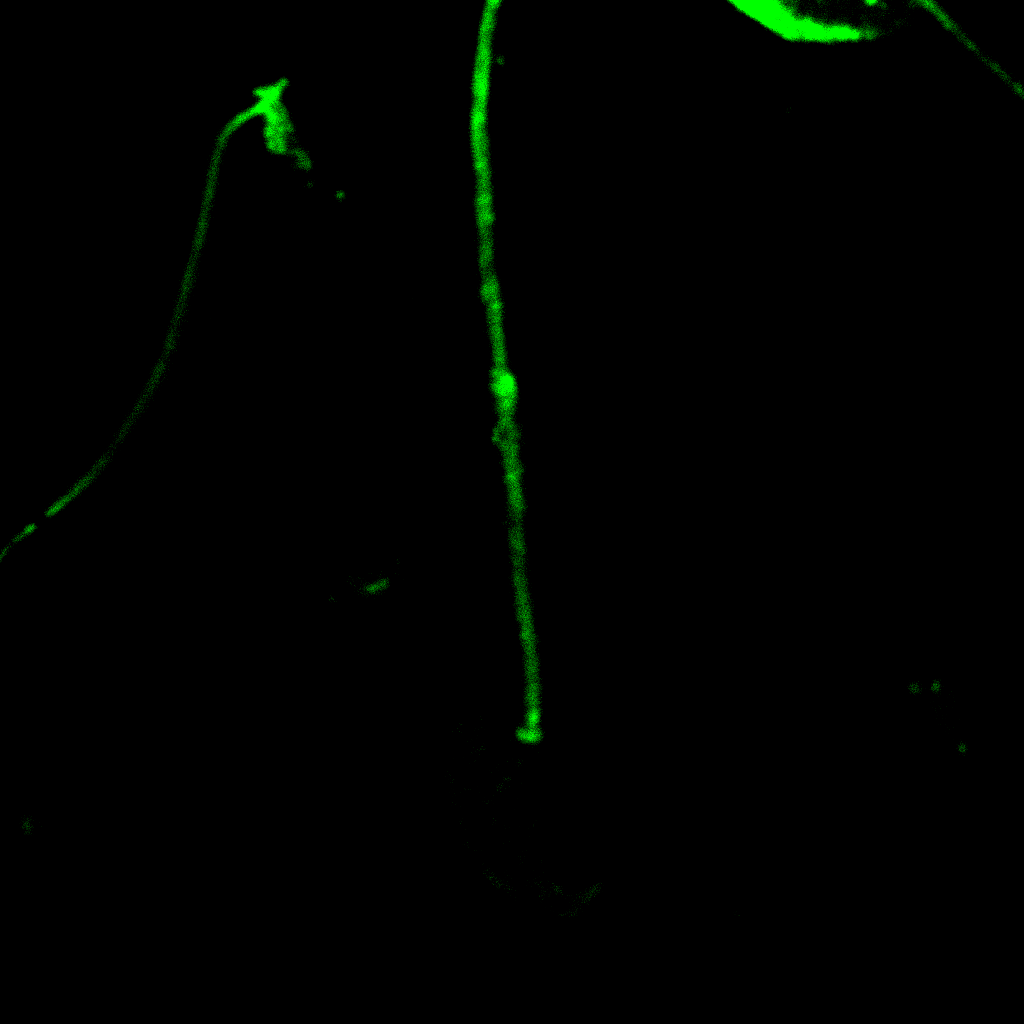

Supplement: Supplementary file 13 [file Data_Sheet_10.ZIP › Fig5B/cfap53 WT M5 10 tritc tub fitc.lif_Series001_z1_ch01.tif]

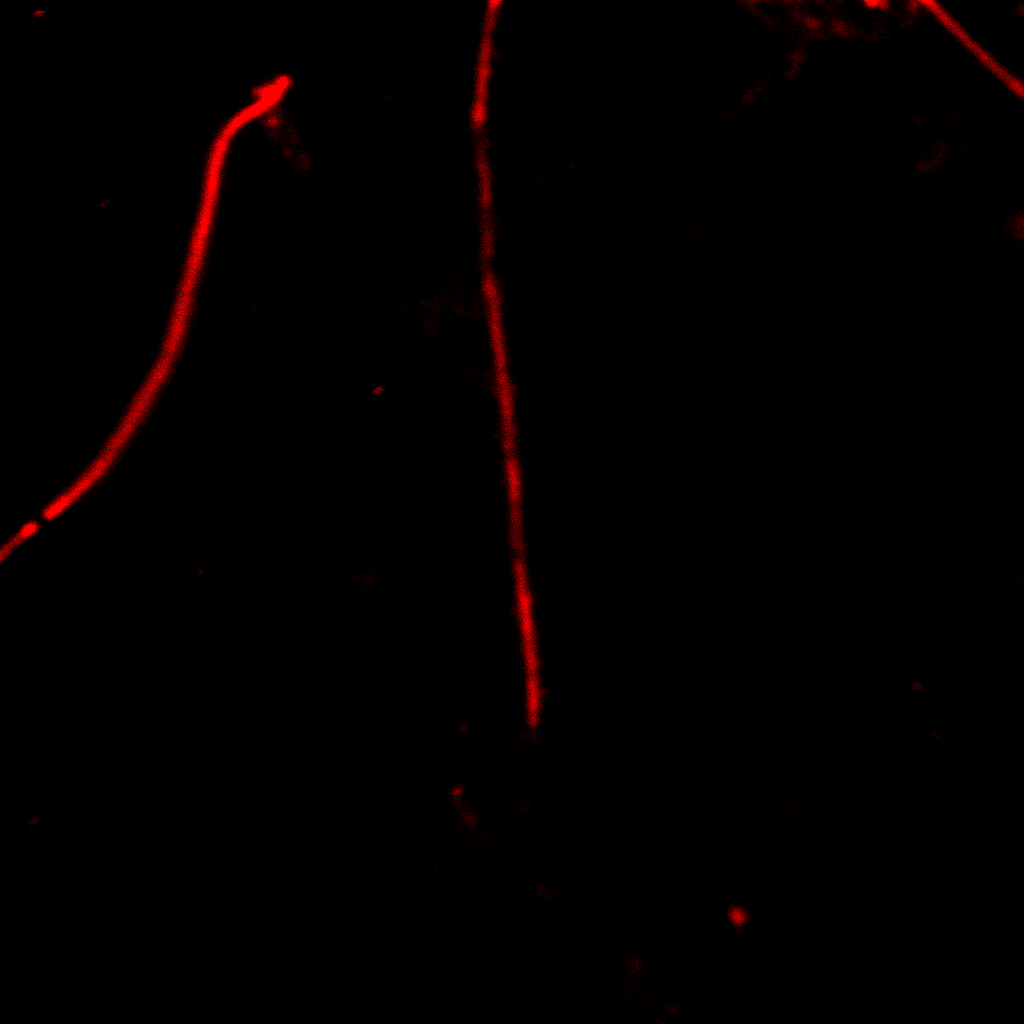

Supplement: Supplementary file 13 [file Data_Sheet_10.ZIP › Fig5B/cfap53 WT M5 10 tritc tub fitc.lif_Series001_z1_ch02.tif]

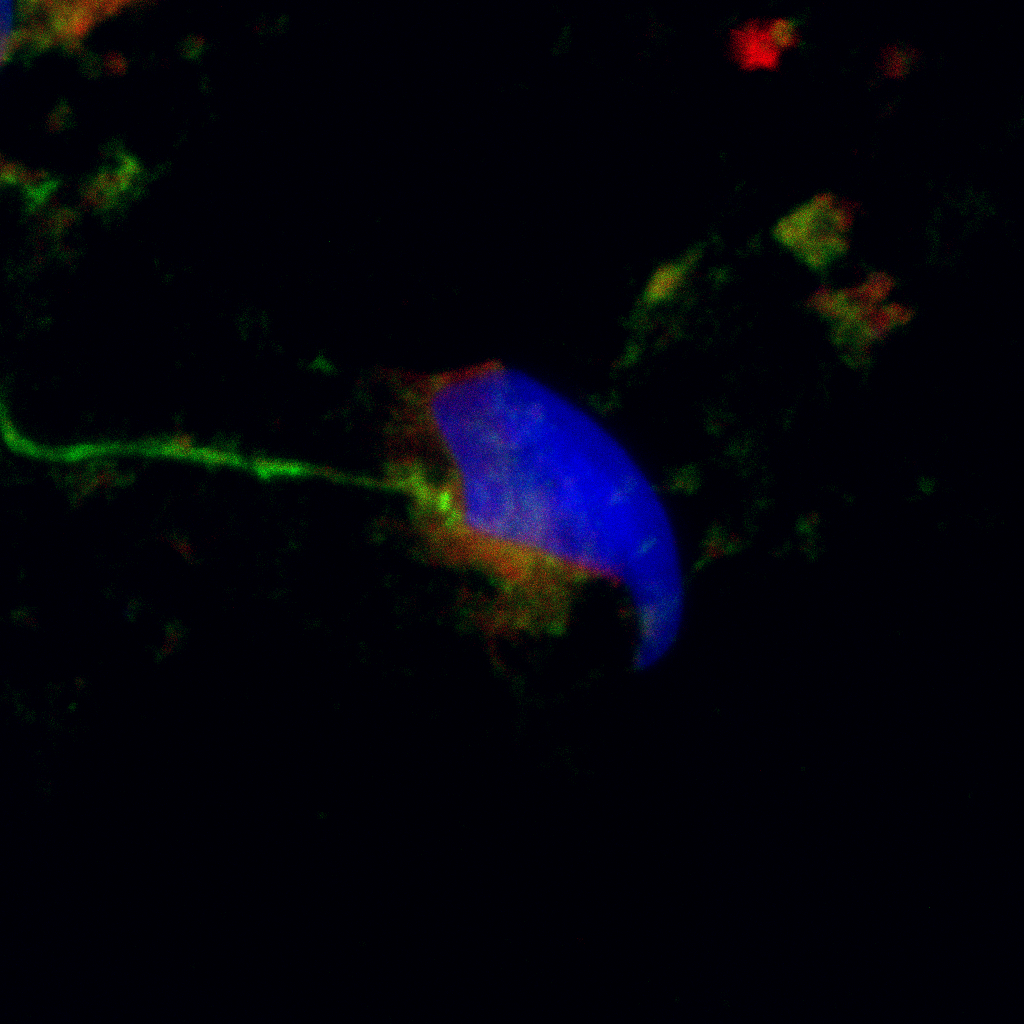

Supplement: Supplementary file 14 [file Data_Sheet_11.ZIP › Fig6G/CFAP53 TRITC IFT88 FITC.lif_11 4.tif]

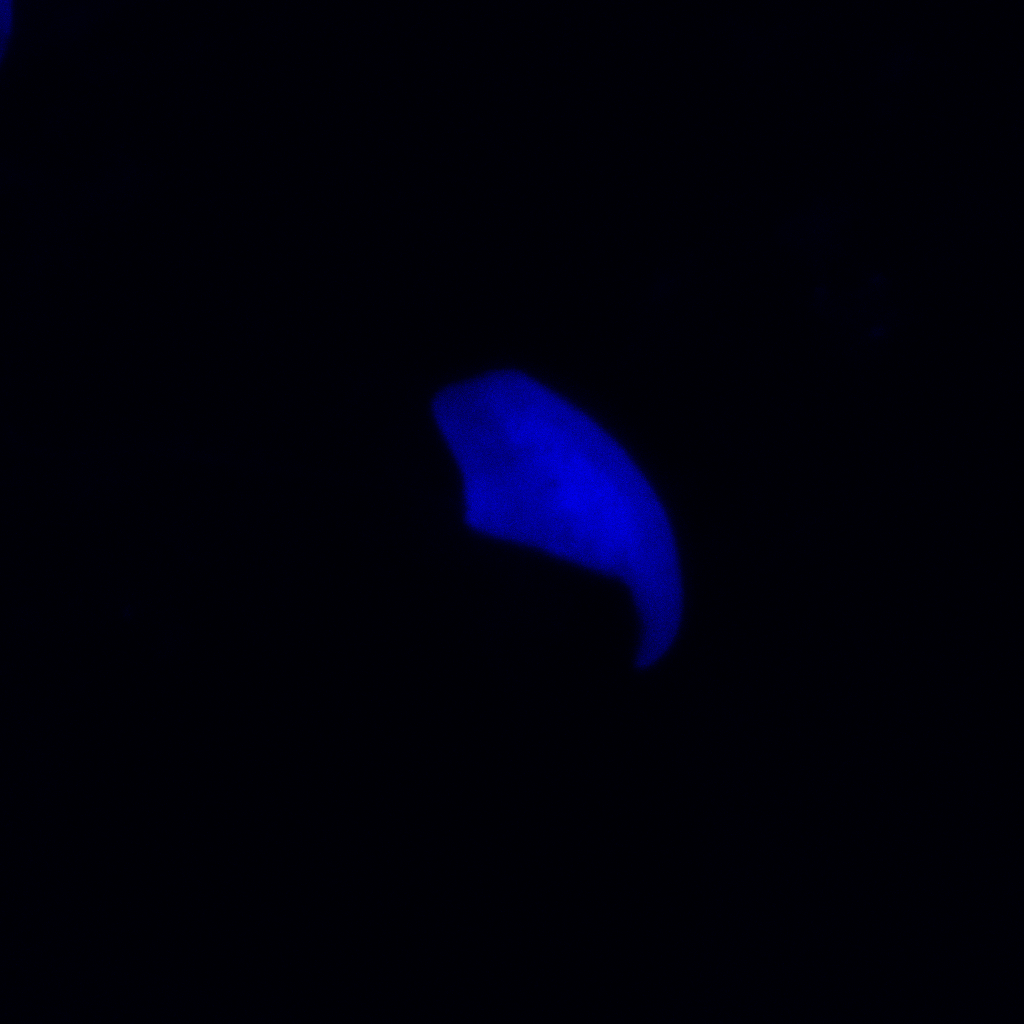

Supplement: Supplementary file 14 [file Data_Sheet_11.ZIP › Fig6G/CFAP53 TRITC IFT88 FITC.lif_11 4_ch00.tif]

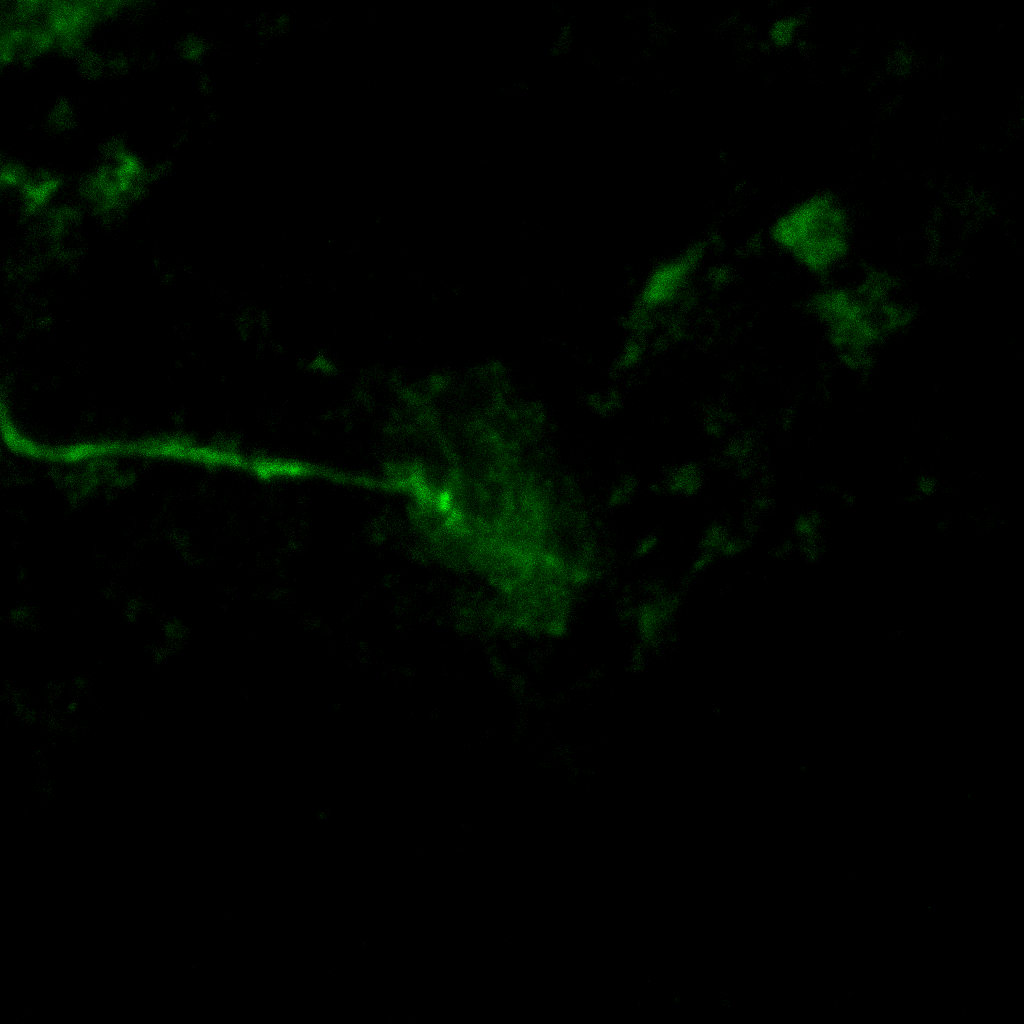

Supplement: Supplementary file 14 [file Data_Sheet_11.ZIP › Fig6G/CFAP53 TRITC IFT88 FITC.lif_11 4_ch01.tif]

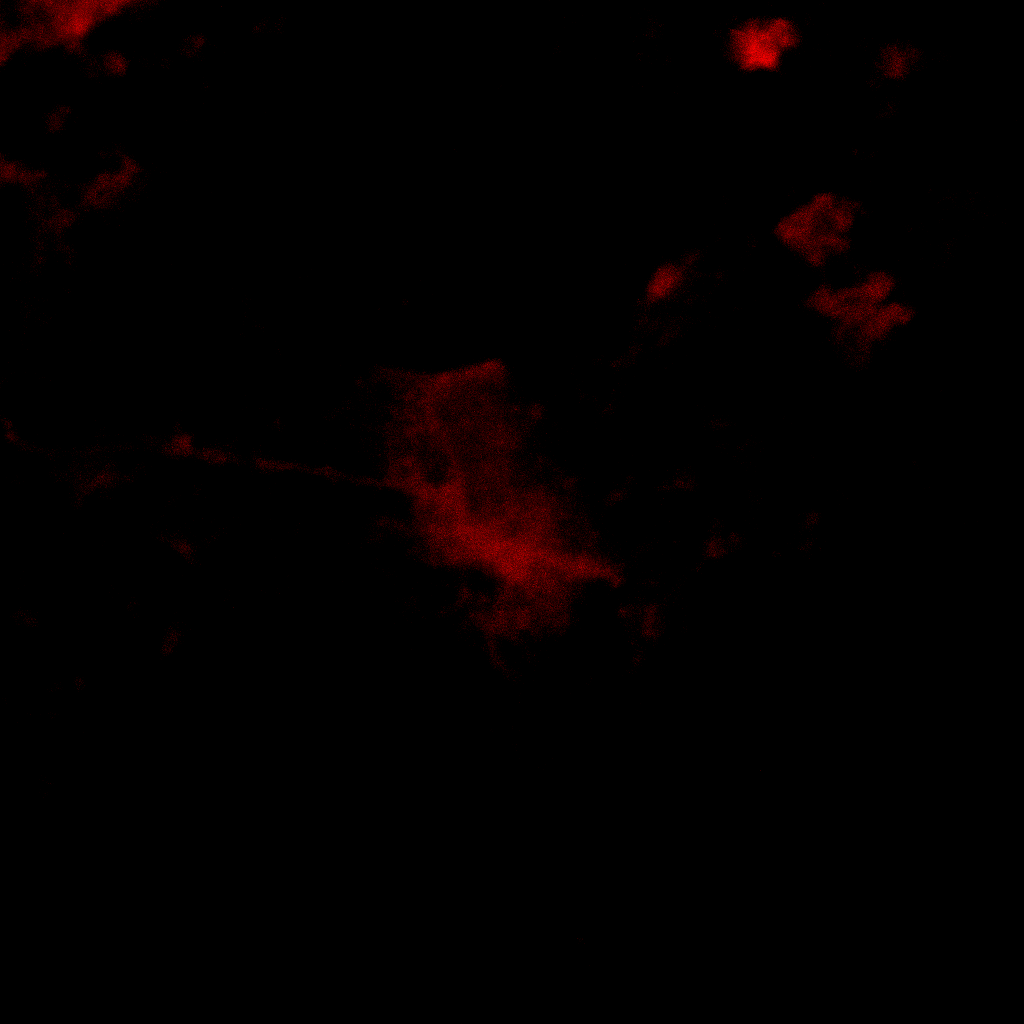

Supplement: Supplementary file 14 [file Data_Sheet_11.ZIP › Fig6G/CFAP53 TRITC IFT88 FITC.lif_11 4_ch02.tif]

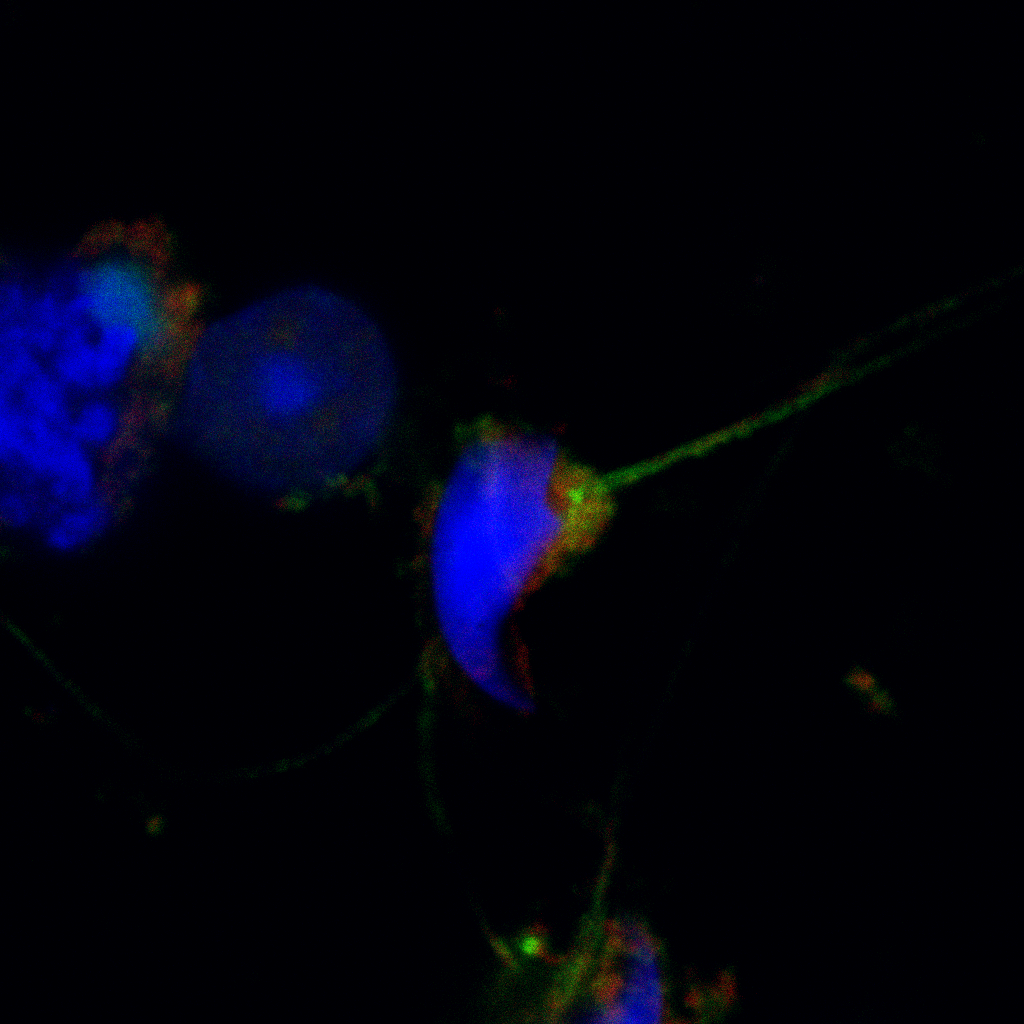

Supplement: Supplementary file 14 [file Data_Sheet_11.ZIP › Fig6G/CFAP53 TRITC IFT88 FITC.lif_13 14_z5.tif]

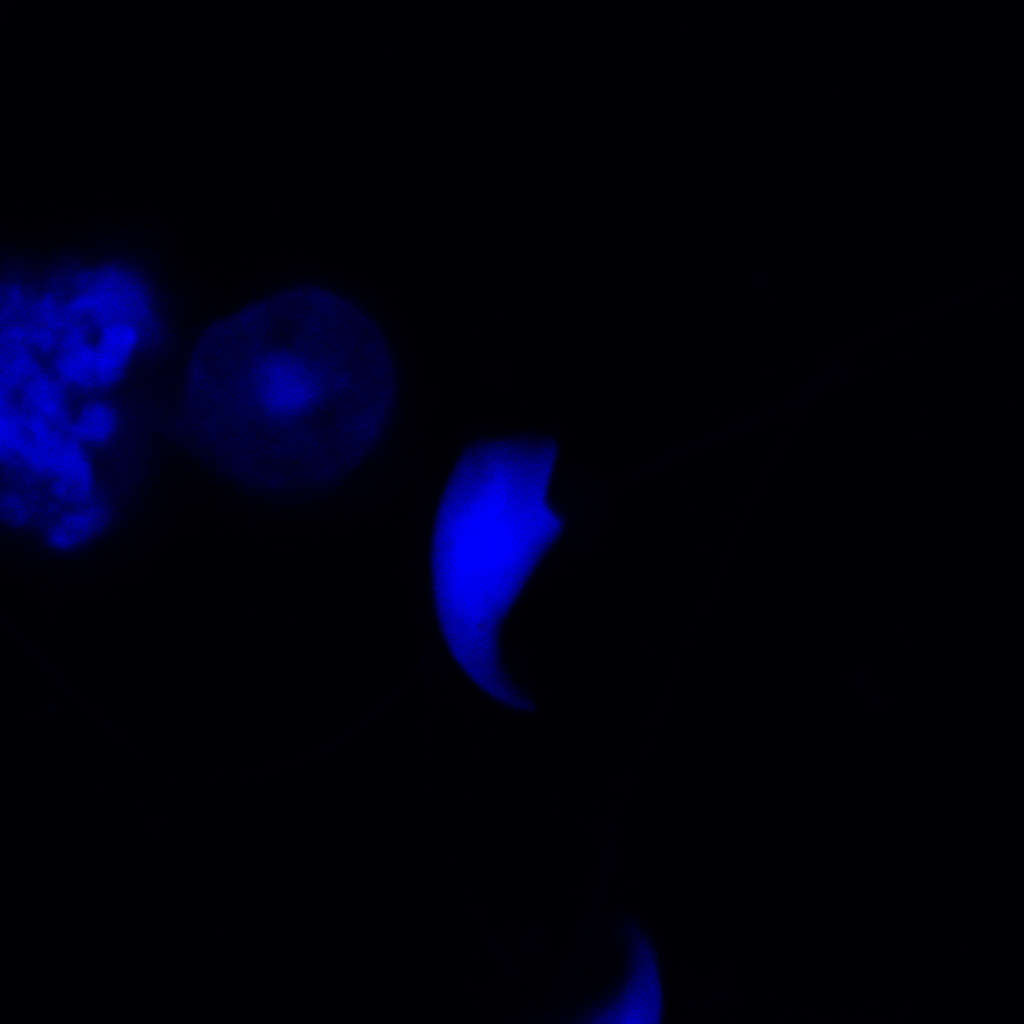

Supplement: Supplementary file 14 [file Data_Sheet_11.ZIP › Fig6G/CFAP53 TRITC IFT88 FITC.lif_13 14_z5_ch00.tif]

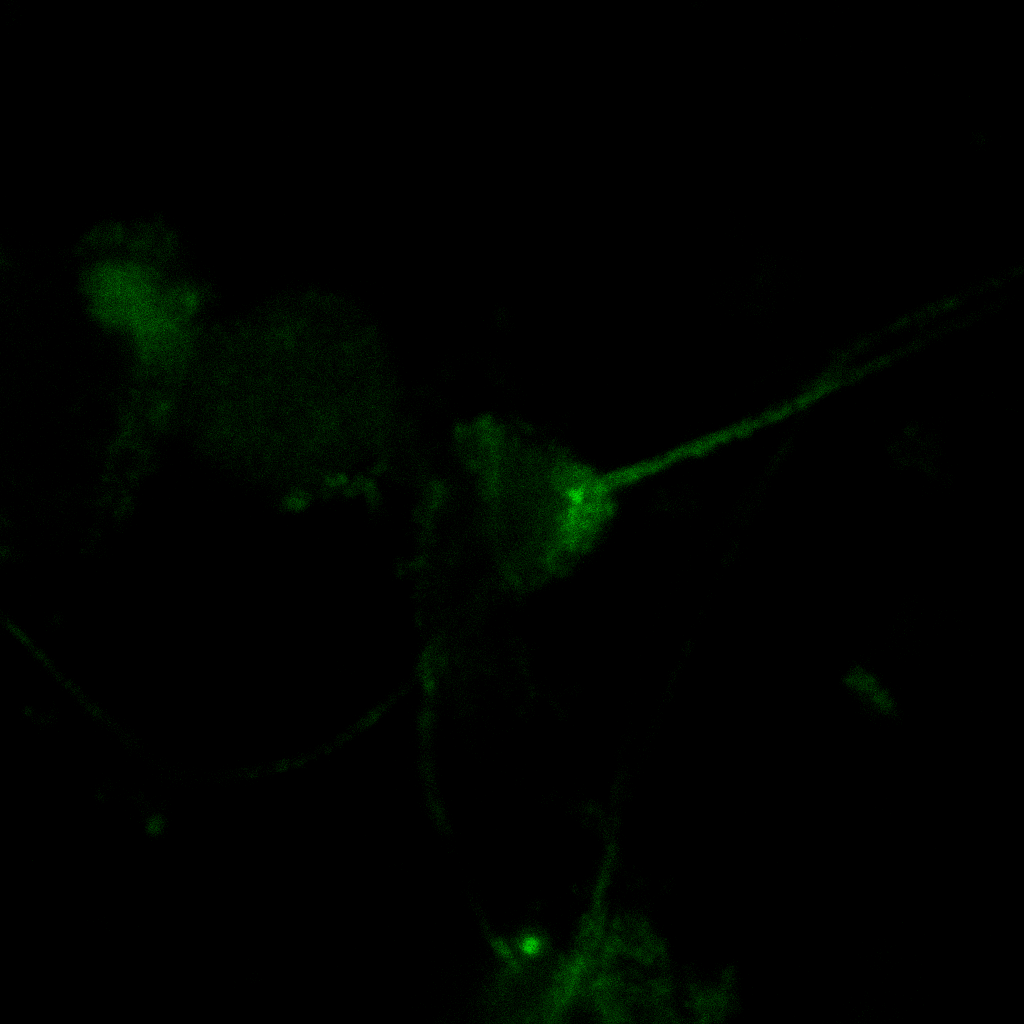

Supplement: Supplementary file 14 [file Data_Sheet_11.ZIP › Fig6G/CFAP53 TRITC IFT88 FITC.lif_13 14_z5_ch01.tif]

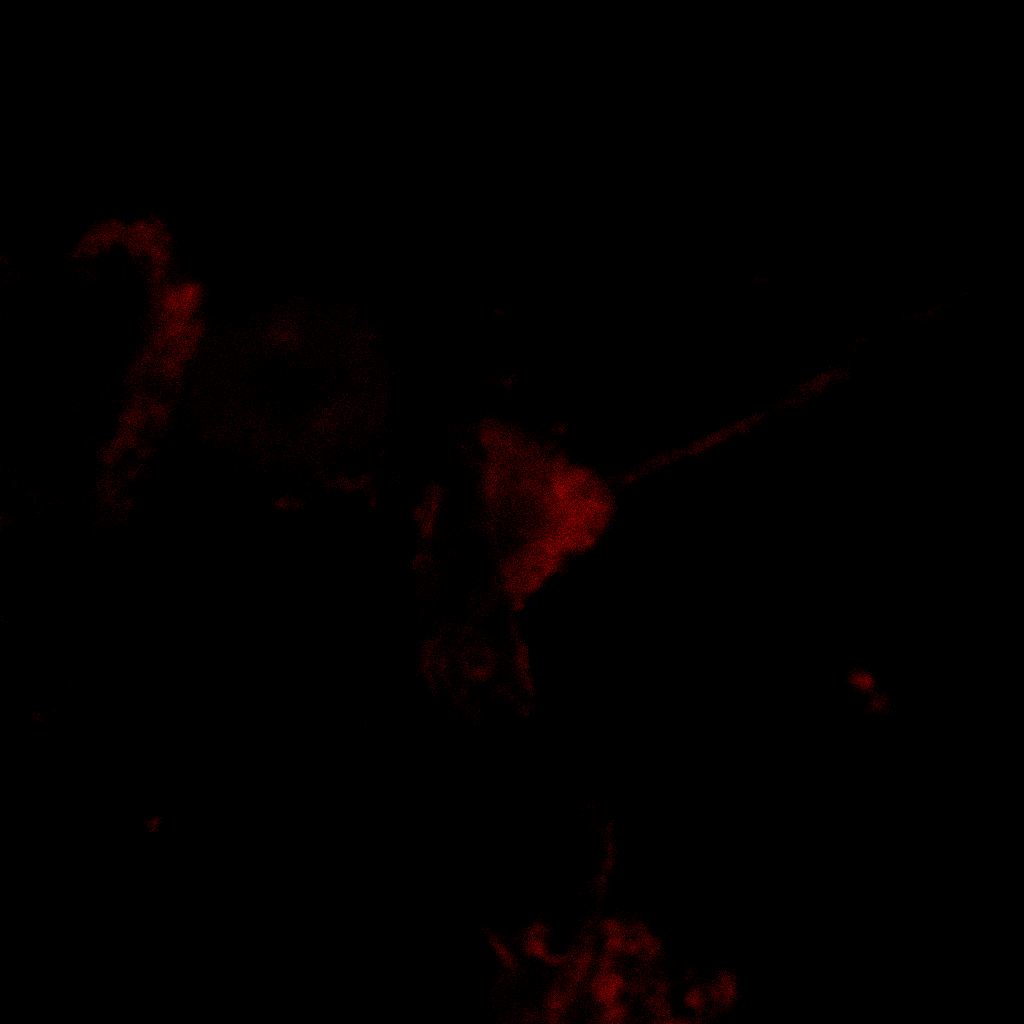

Supplement: Supplementary file 14 [file Data_Sheet_11.ZIP › Fig6G/CFAP53 TRITC IFT88 FITC.lif_13 14_z5_ch02.tif]

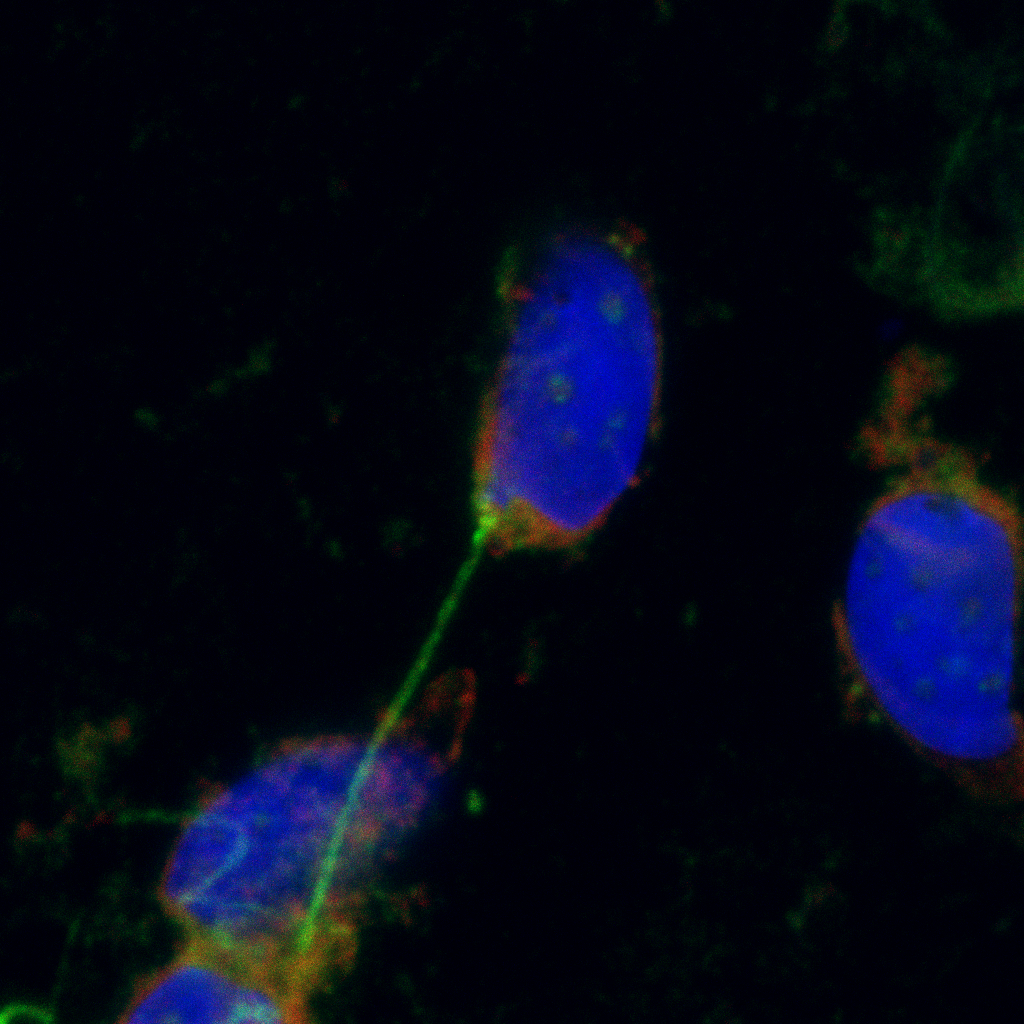

Supplement: Supplementary file 14 [file Data_Sheet_11.ZIP › Fig6G/CFAP53 TRITC IFT88 FITC.lif_9_Processed001.tif]

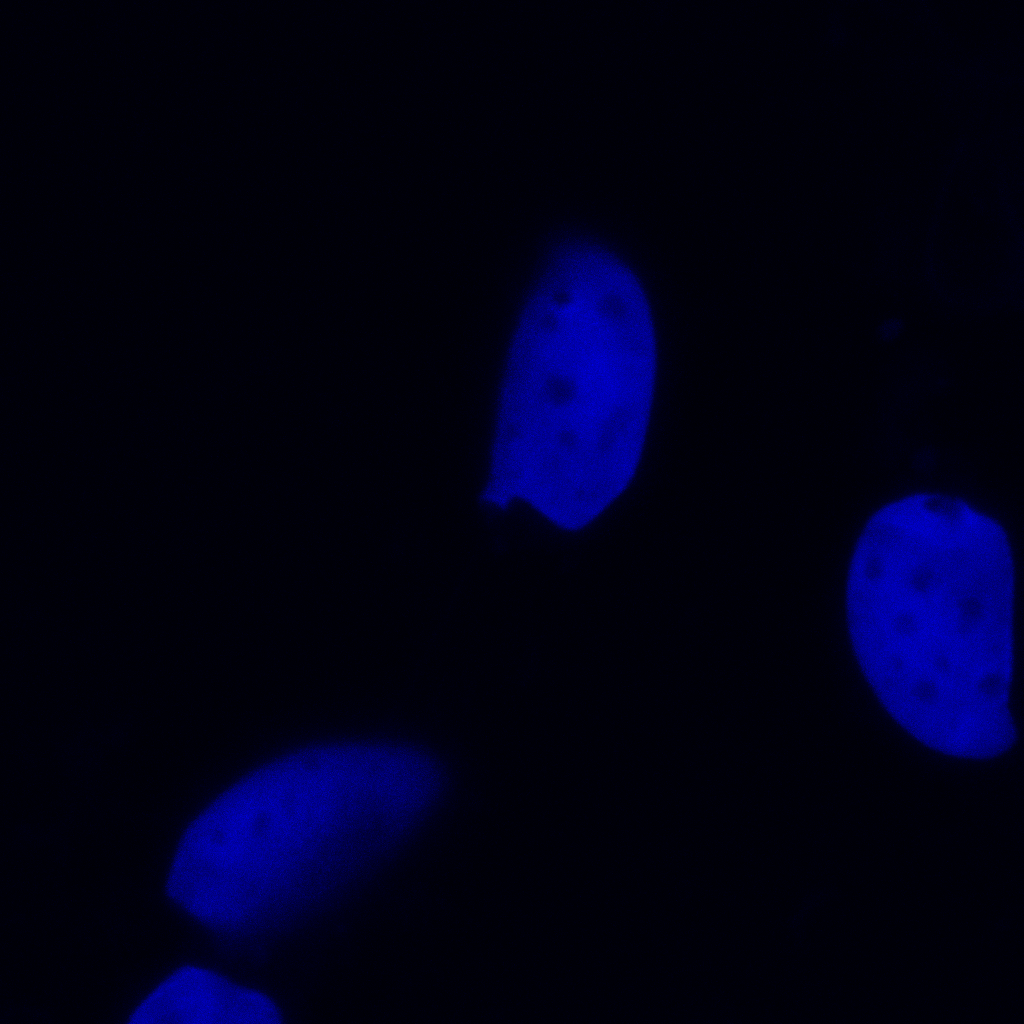

Supplement: Supplementary file 14 [file Data_Sheet_11.ZIP › Fig6G/CFAP53 TRITC IFT88 FITC.lif_9_Processed001_ch00.tif]

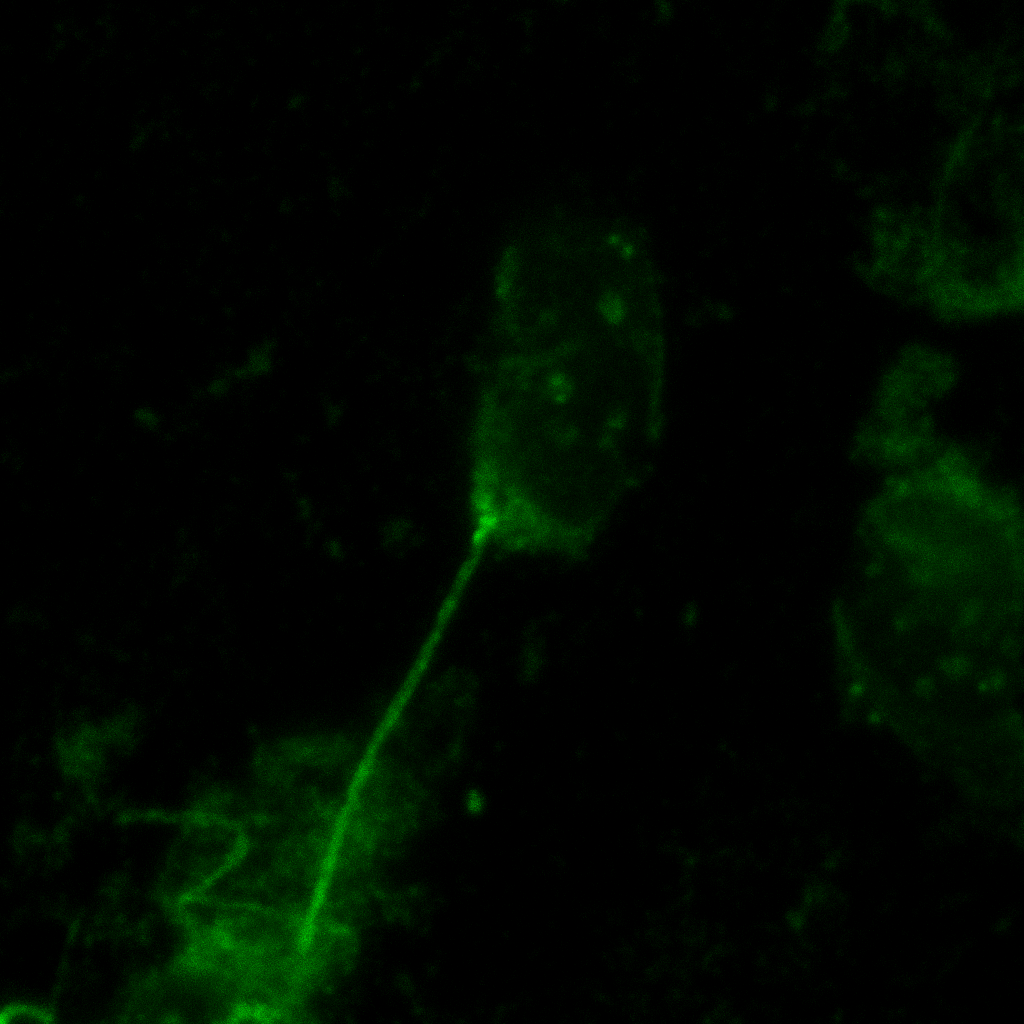

Supplement: Supplementary file 14 [file Data_Sheet_11.ZIP › Fig6G/CFAP53 TRITC IFT88 FITC.lif_9_Processed001_ch01.tif]

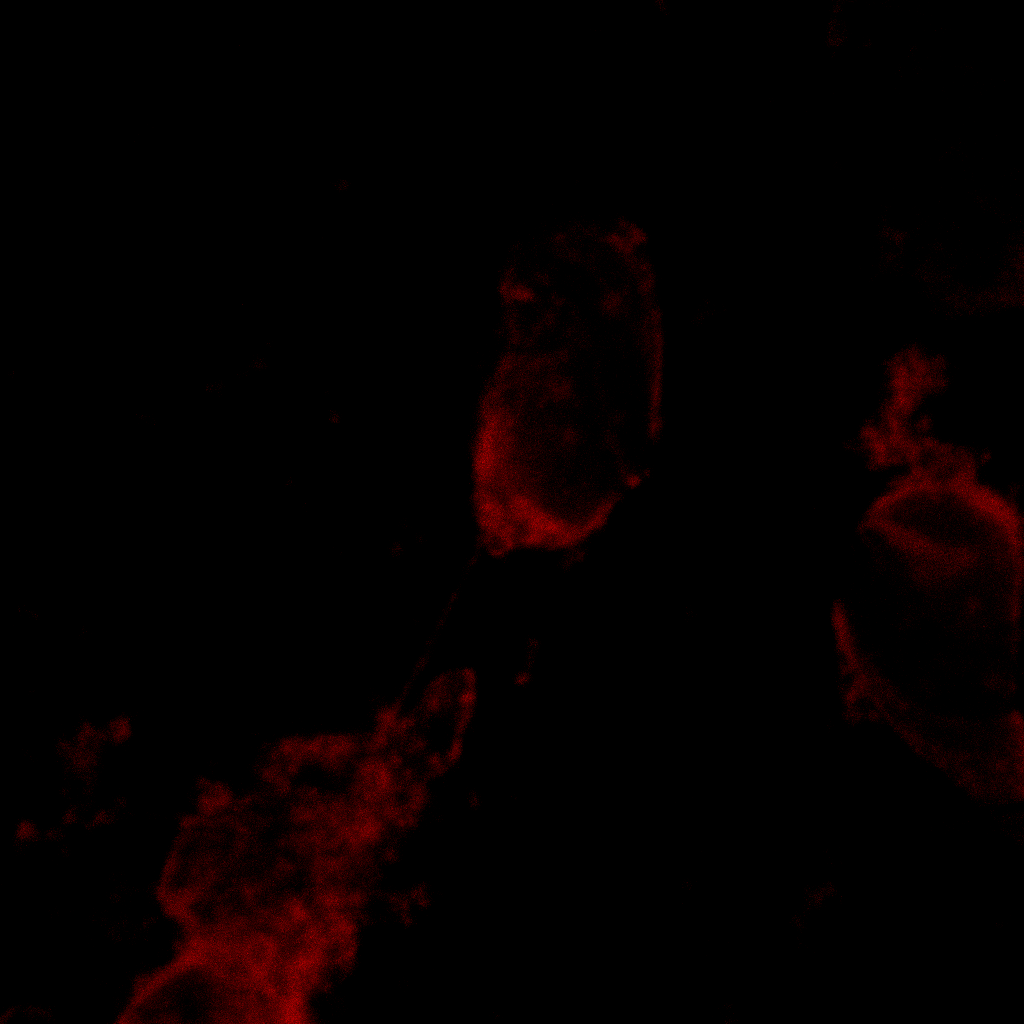

Supplement: Supplementary file 14 [file Data_Sheet_11.ZIP › Fig6G/CFAP53 TRITC IFT88 FITC.lif_9_Processed001_ch02.tif]

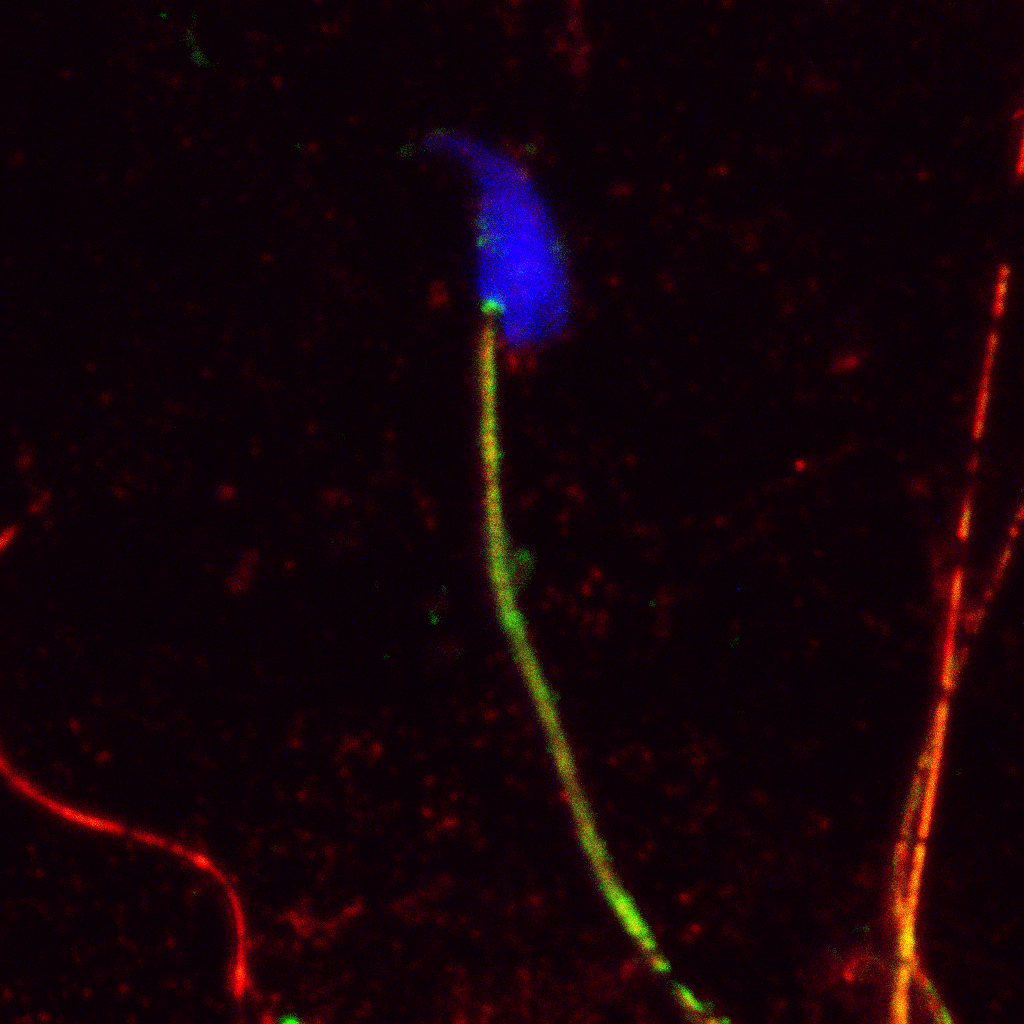

Supplement: Supplementary file 14 [file Data_Sheet_11.ZIP › Fig6G/cfap53 WT M5 15 tritc tub fitc.lif_Series006_Processed001.tif]

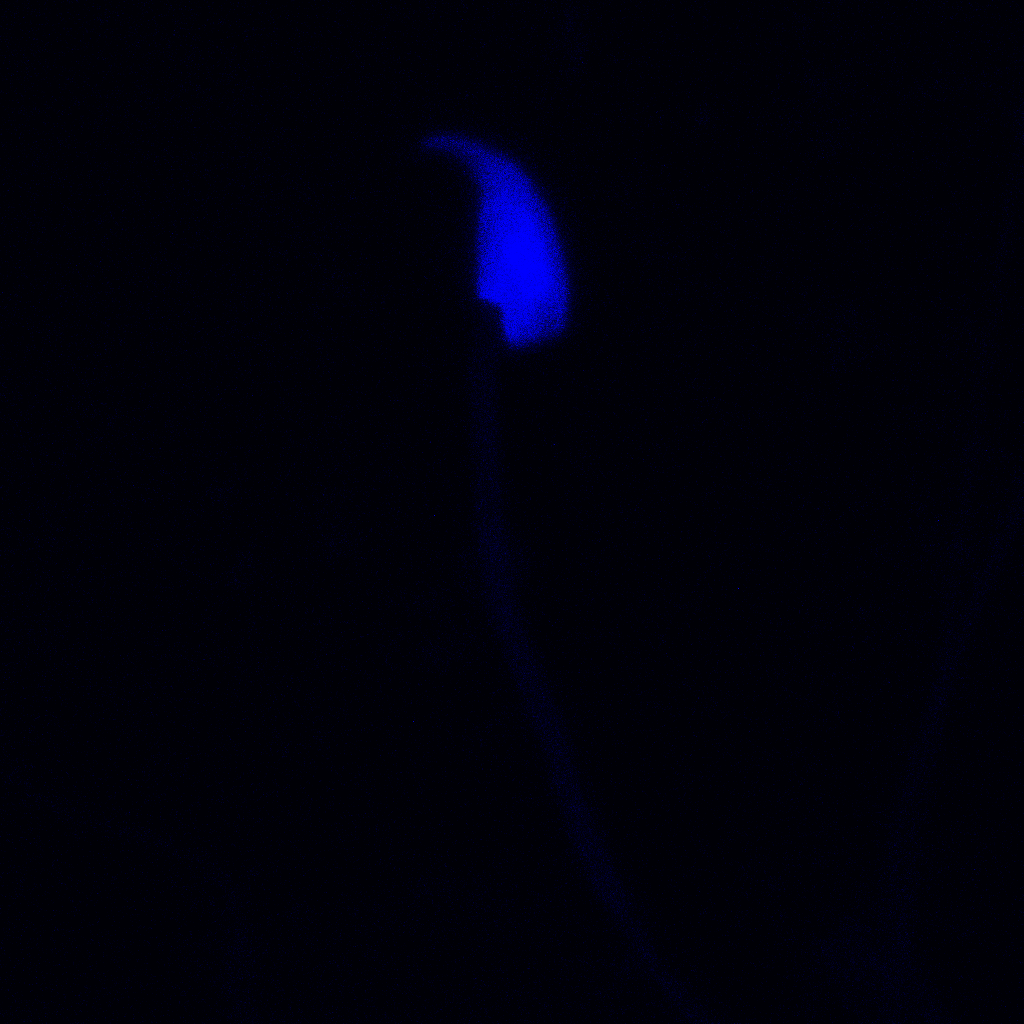

Supplement: Supplementary file 14 [file Data_Sheet_11.ZIP › Fig6G/cfap53 WT M5 15 tritc tub fitc.lif_Series006_Processed001_ch00.tif]

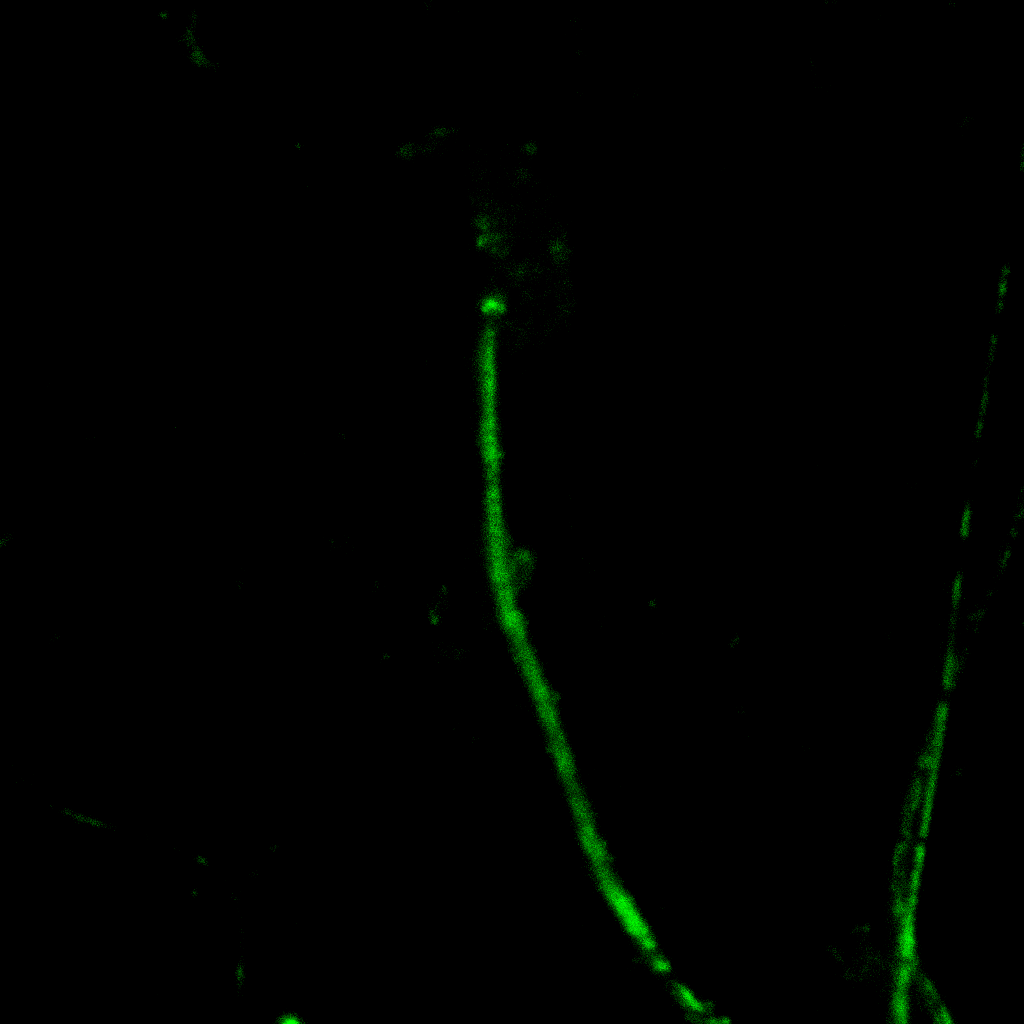

Supplement: Supplementary file 14 [file Data_Sheet_11.ZIP › Fig6G/cfap53 WT M5 15 tritc tub fitc.lif_Series006_Processed001_ch01.tif]

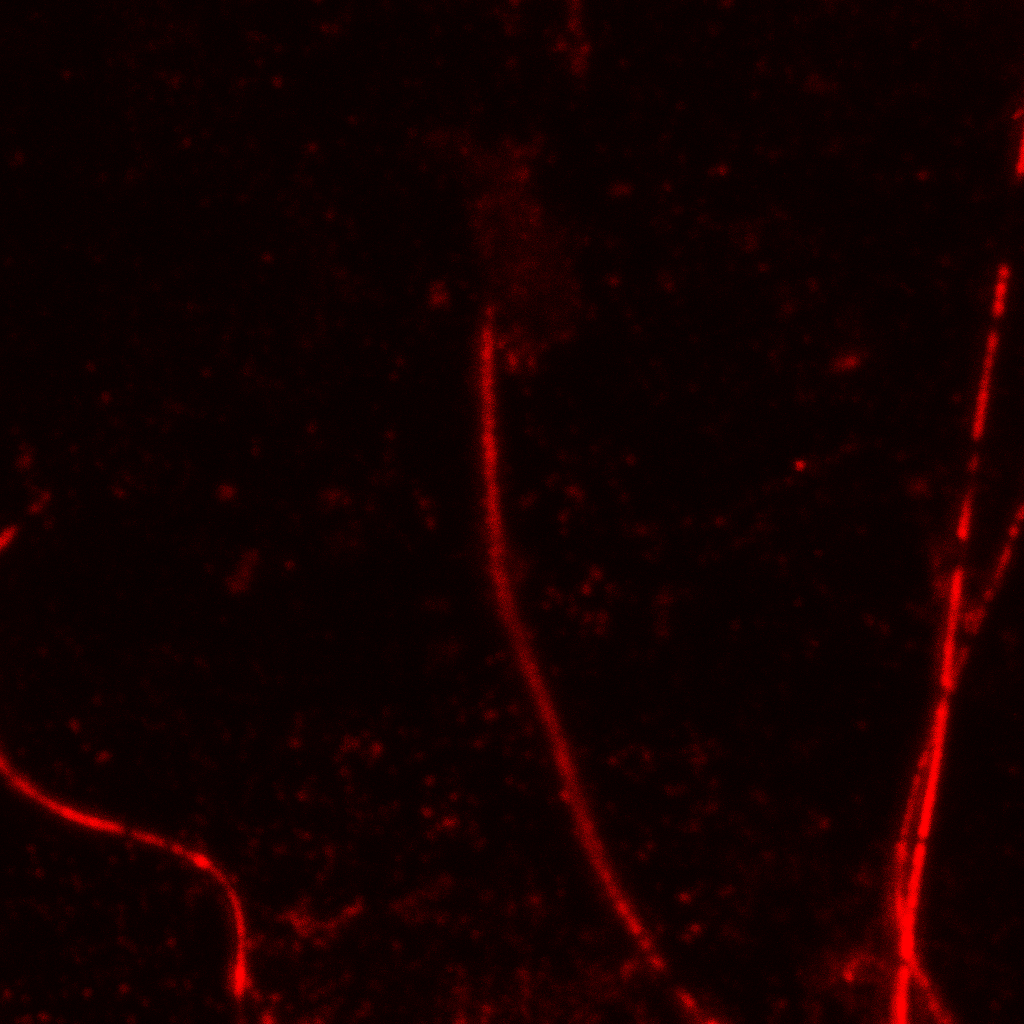

Supplement: Supplementary file 14 [file Data_Sheet_11.ZIP › Fig6G/cfap53 WT M5 15 tritc tub fitc.lif_Series006_Processed001_ch02.tif]

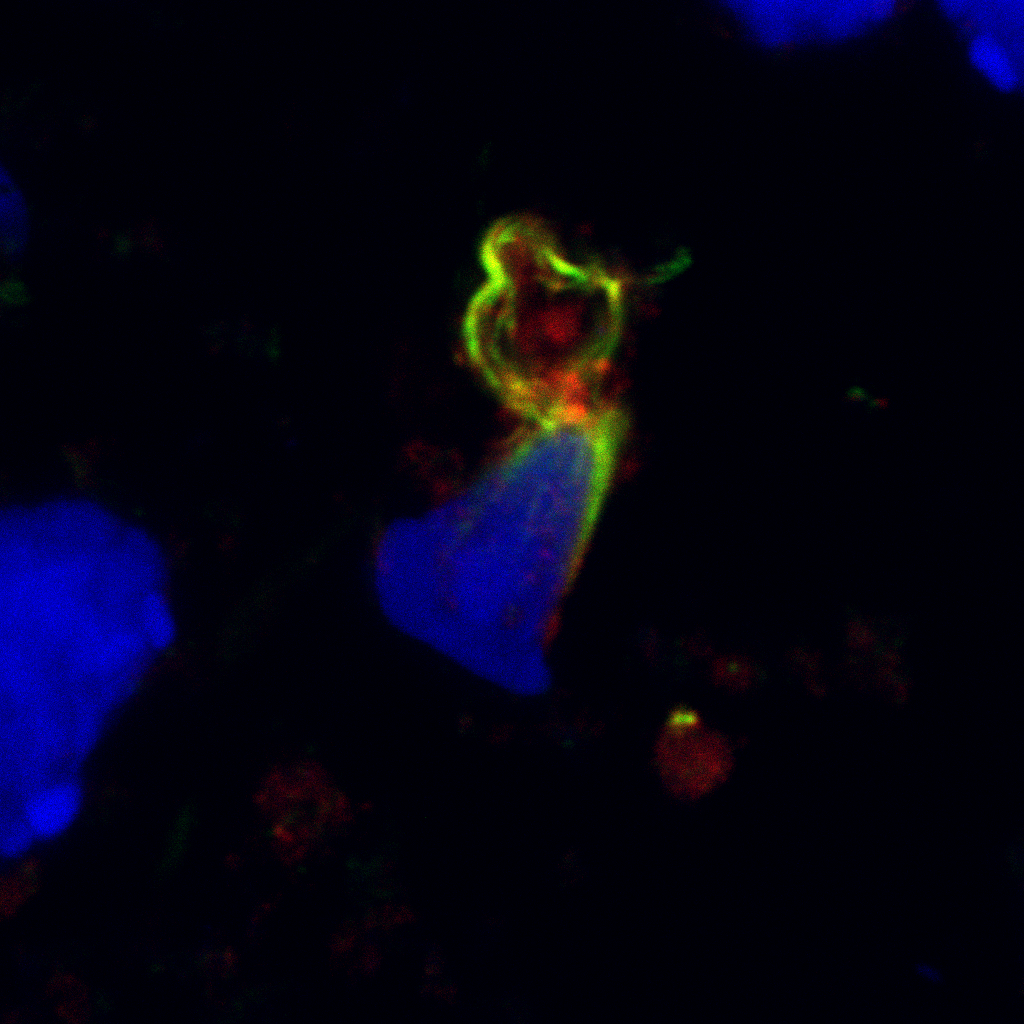

Supplement: Supplementary file 15 [file Data_Sheet_12.ZIP › Fig6H/KO/cfap53 ko IFT88 TRITC M ACTUB.lif_11_Processed001.tif]

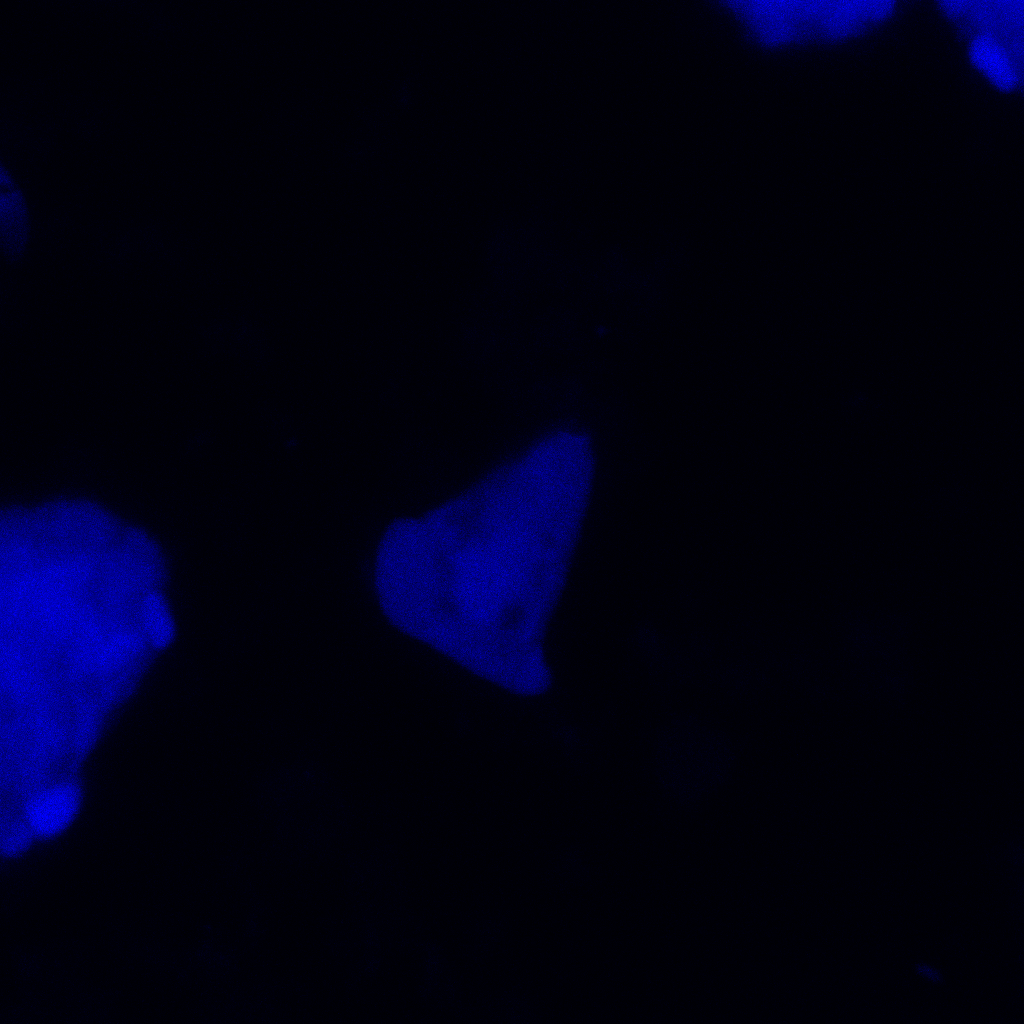

Supplement: Supplementary file 15 [file Data_Sheet_12.ZIP › Fig6H/KO/cfap53 ko IFT88 TRITC M ACTUB.lif_11_Processed001_ch00.tif]

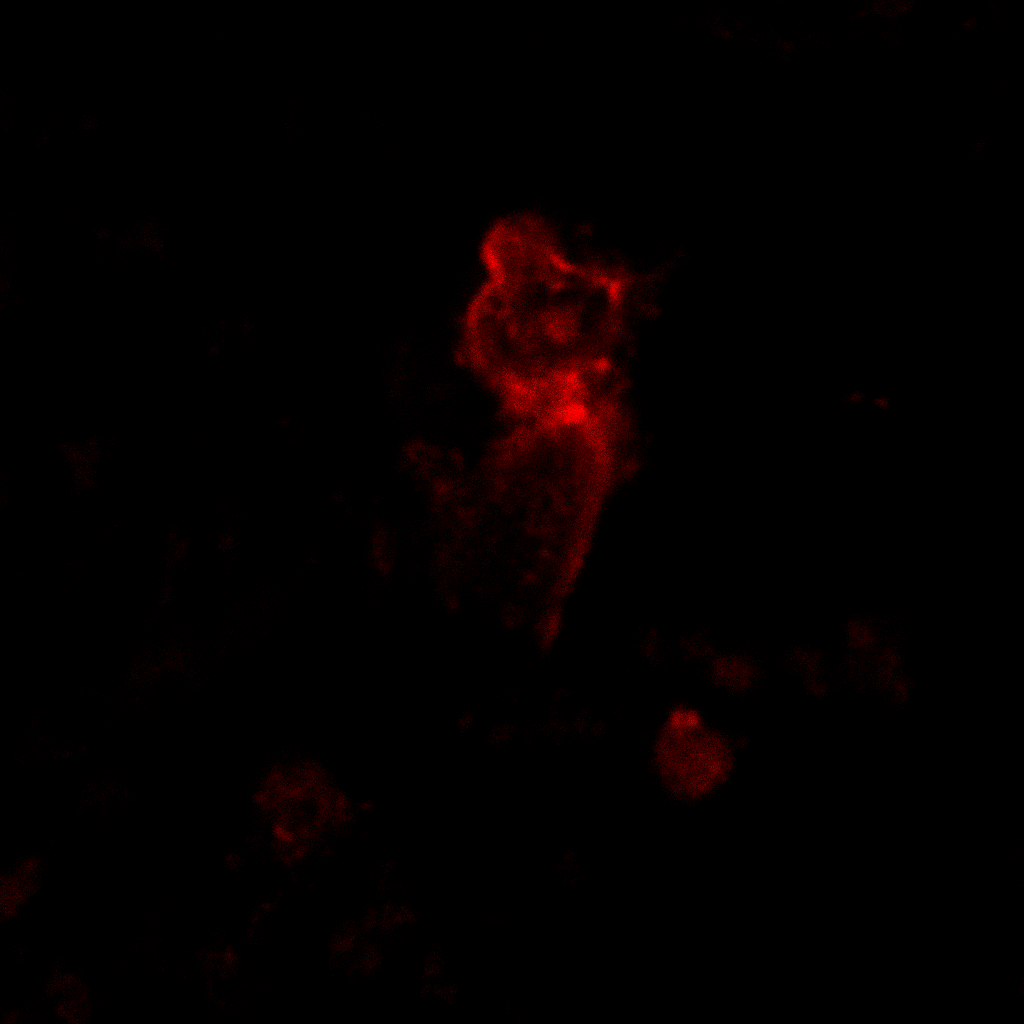

Supplement: Supplementary file 15 [file Data_Sheet_12.ZIP › Fig6H/KO/cfap53 ko IFT88 TRITC M ACTUB.lif_11_Processed001_ch02.tif]

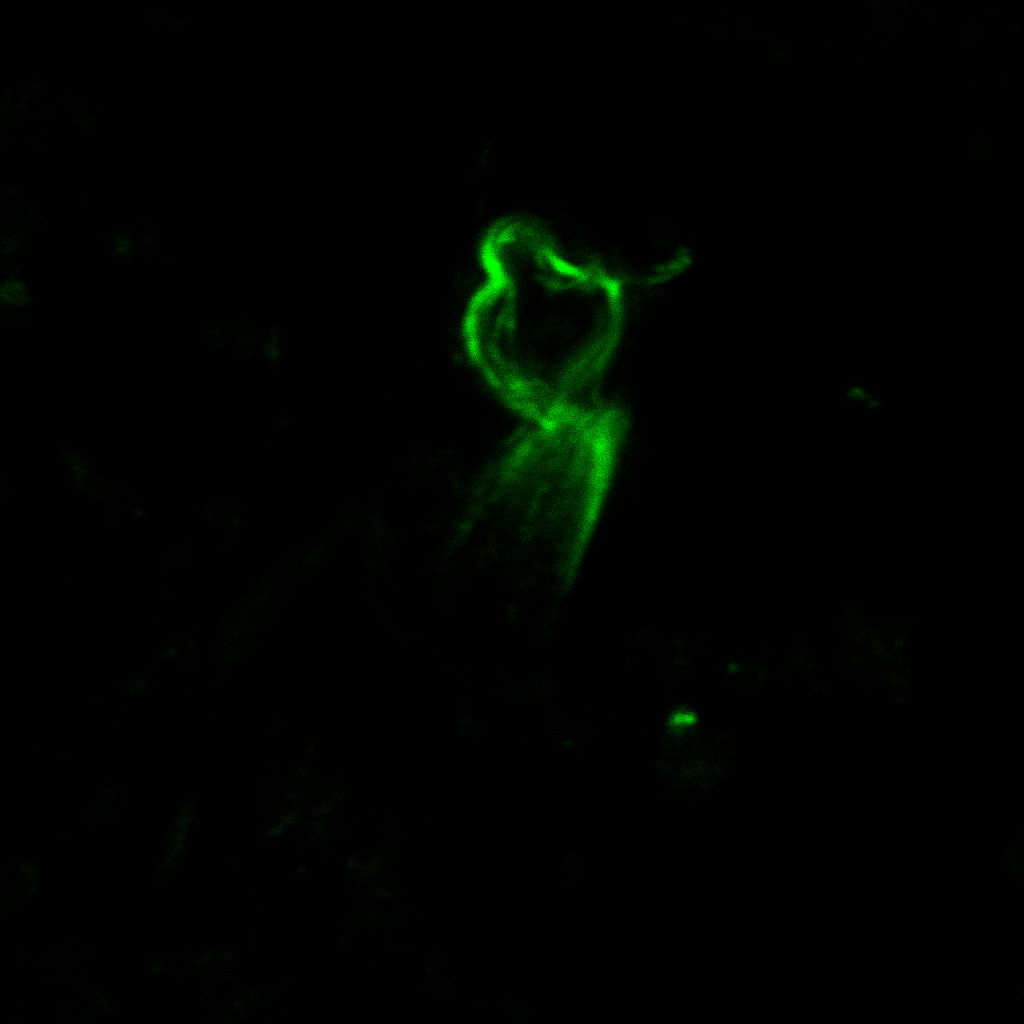

Supplement: Supplementary file 15 [file Data_Sheet_12.ZIP › Fig6H/KO/cfap53 ko IFT88 TRITC M ACTUB.lif_11_Processed001_ch03.tif]

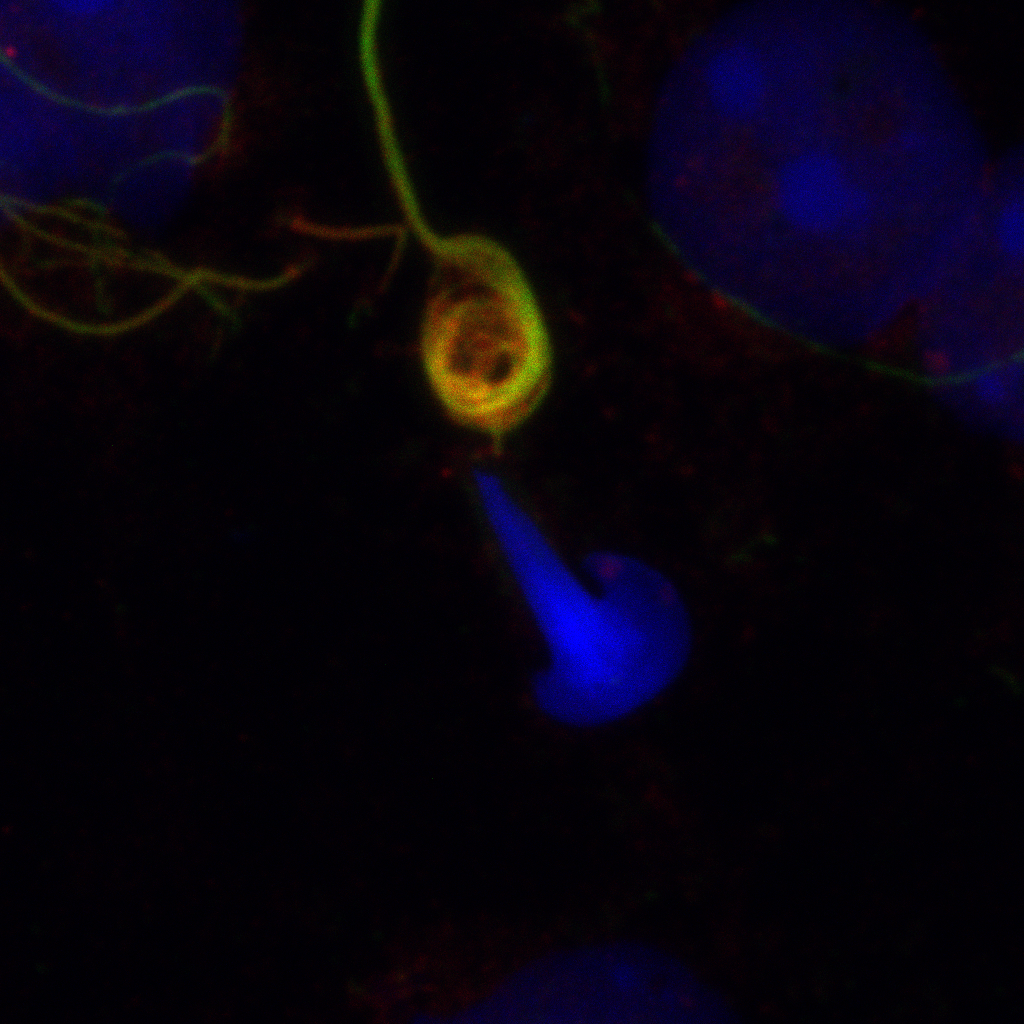

Supplement: Supplementary file 15 [file Data_Sheet_12.ZIP › Fig6H/KO/cfap53 ko IFT88 TRITC M ACTUB.lif_14-15_Processed001.tif]

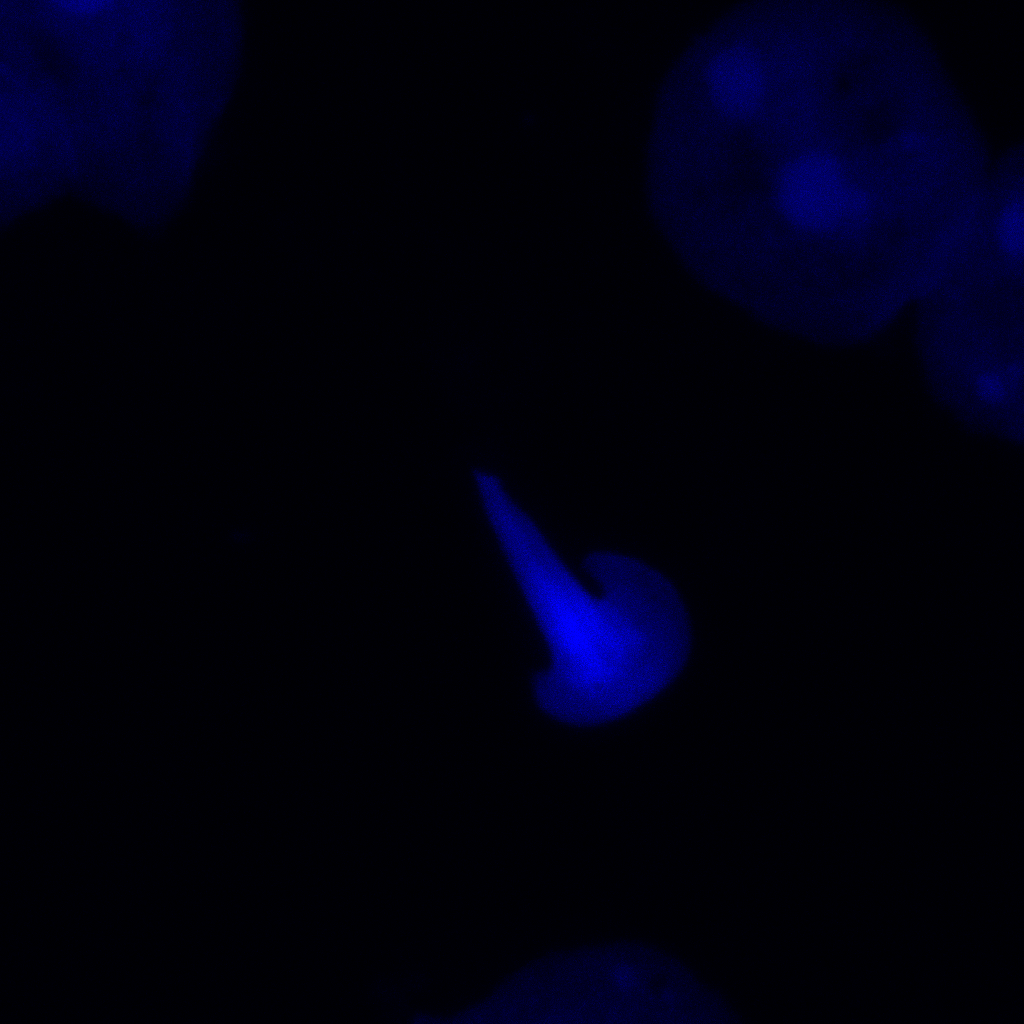

Supplement: Supplementary file 15 [file Data_Sheet_12.ZIP › Fig6H/KO/cfap53 ko IFT88 TRITC M ACTUB.lif_14-15_Processed001_ch00.tif]

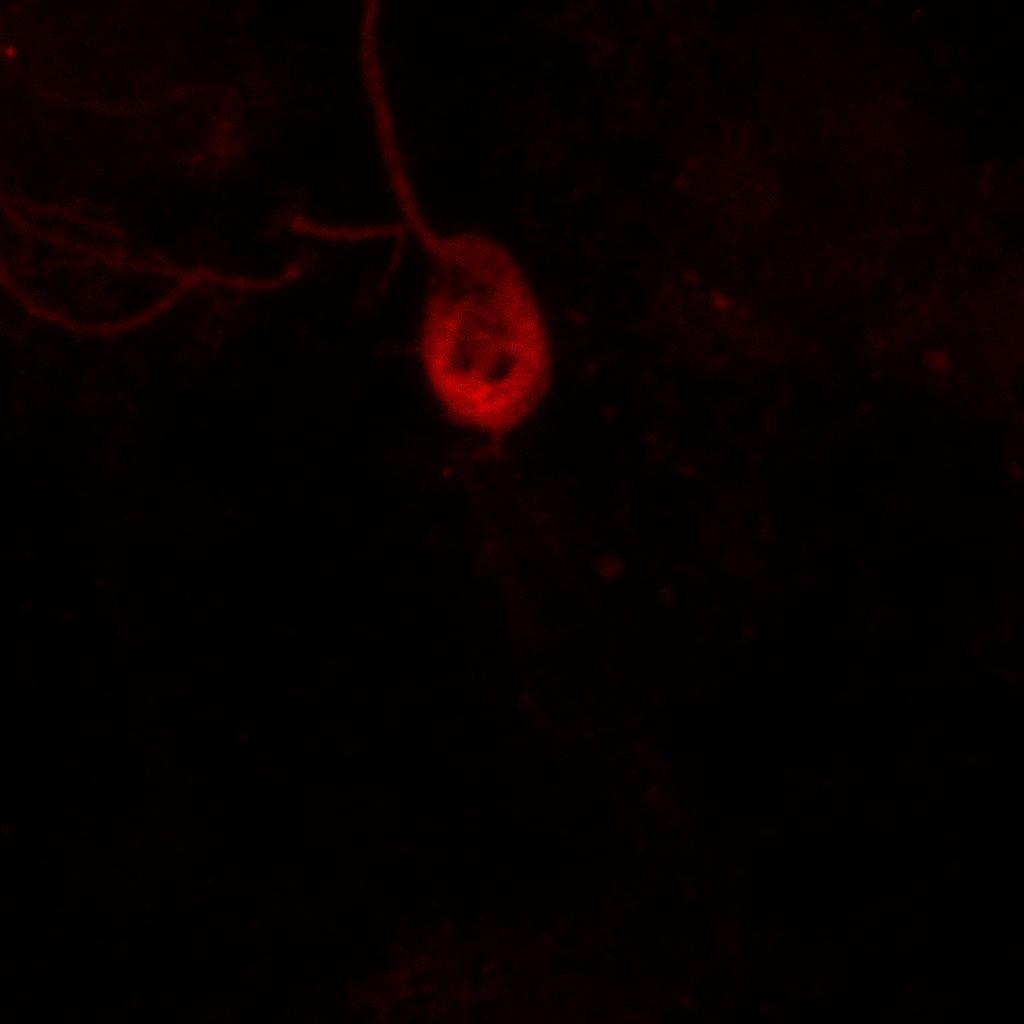

Supplement: Supplementary file 15 [file Data_Sheet_12.ZIP › Fig6H/KO/cfap53 ko IFT88 TRITC M ACTUB.lif_14-15_Processed001_ch02.tif]

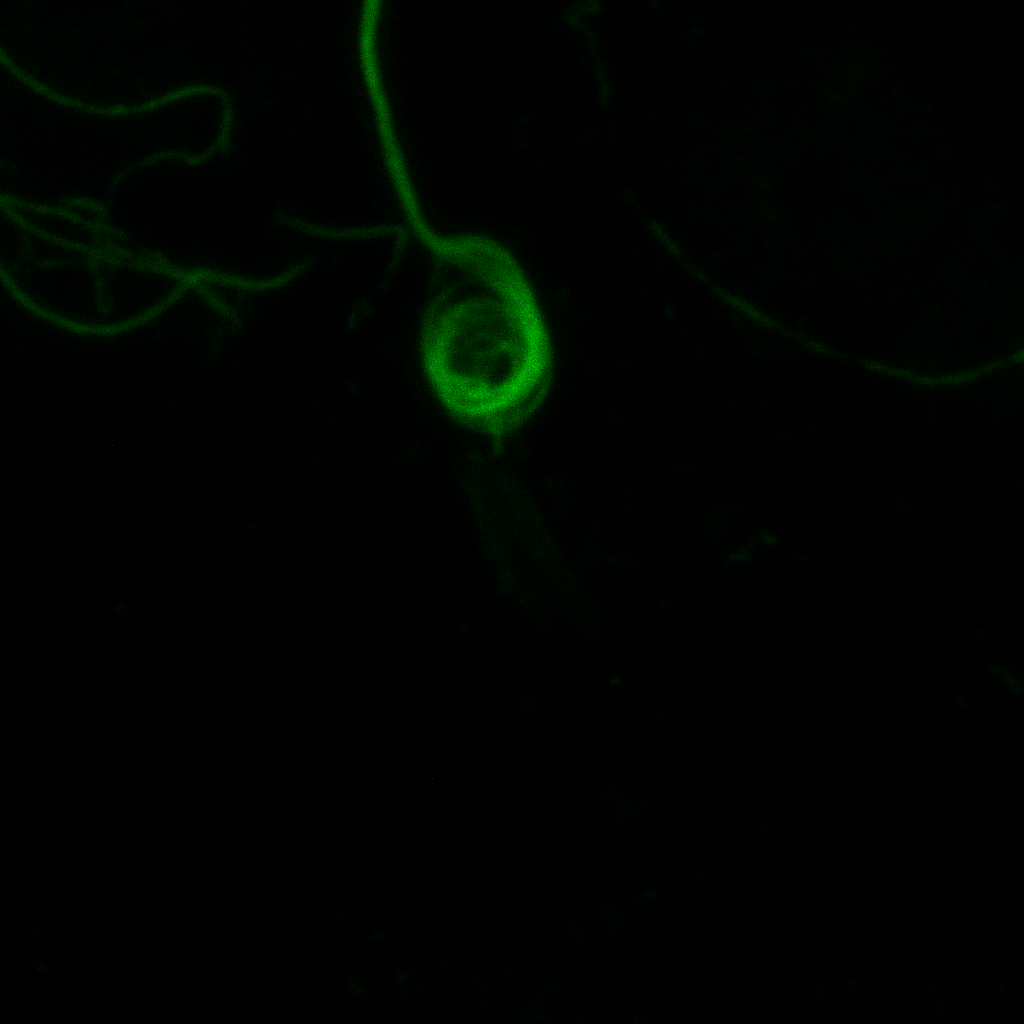

Supplement: Supplementary file 15 [file Data_Sheet_12.ZIP › Fig6H/KO/cfap53 ko IFT88 TRITC M ACTUB.lif_14-15_Processed001_ch03.tif]

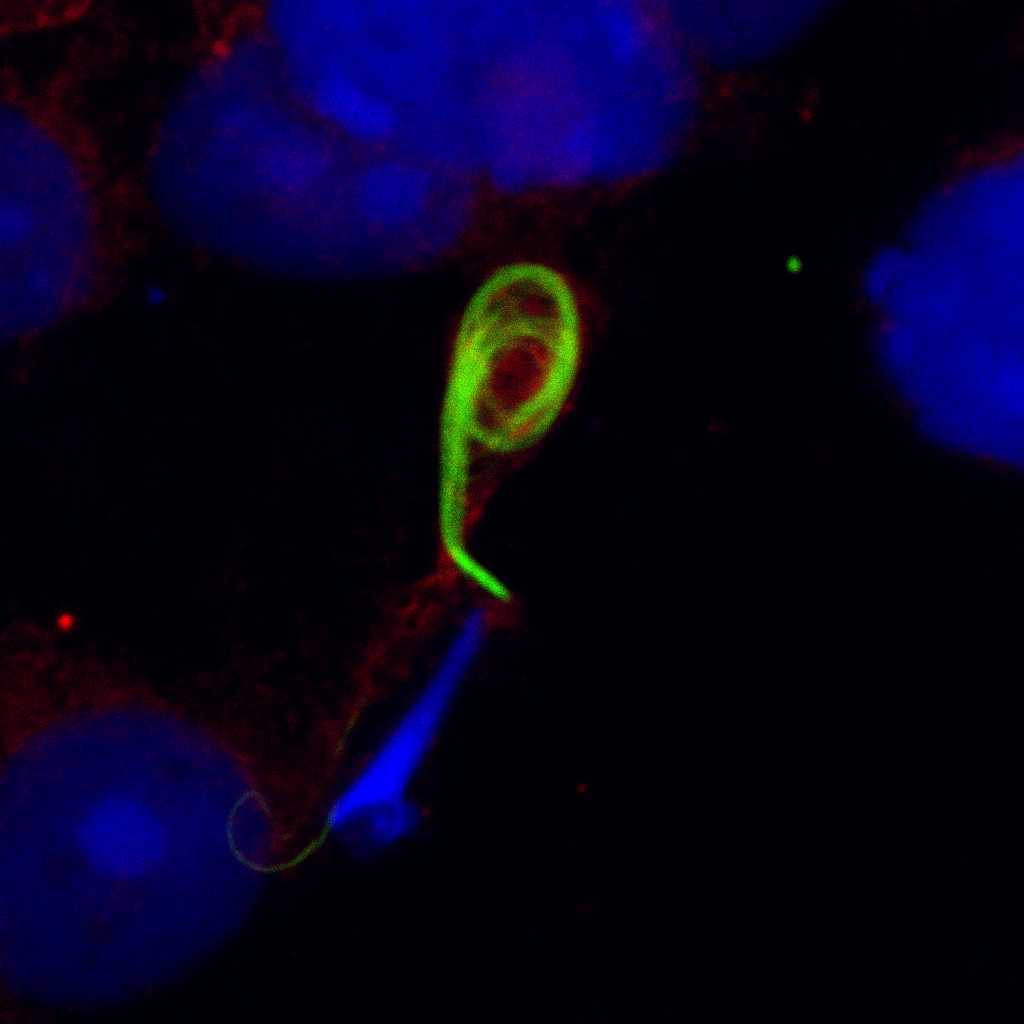

Supplement: Supplementary file 15 [file Data_Sheet_12.ZIP › Fig6H/KO/cfap53 ko IFT88 TRITC M ACTUB.lif_15_Processed001.tif]

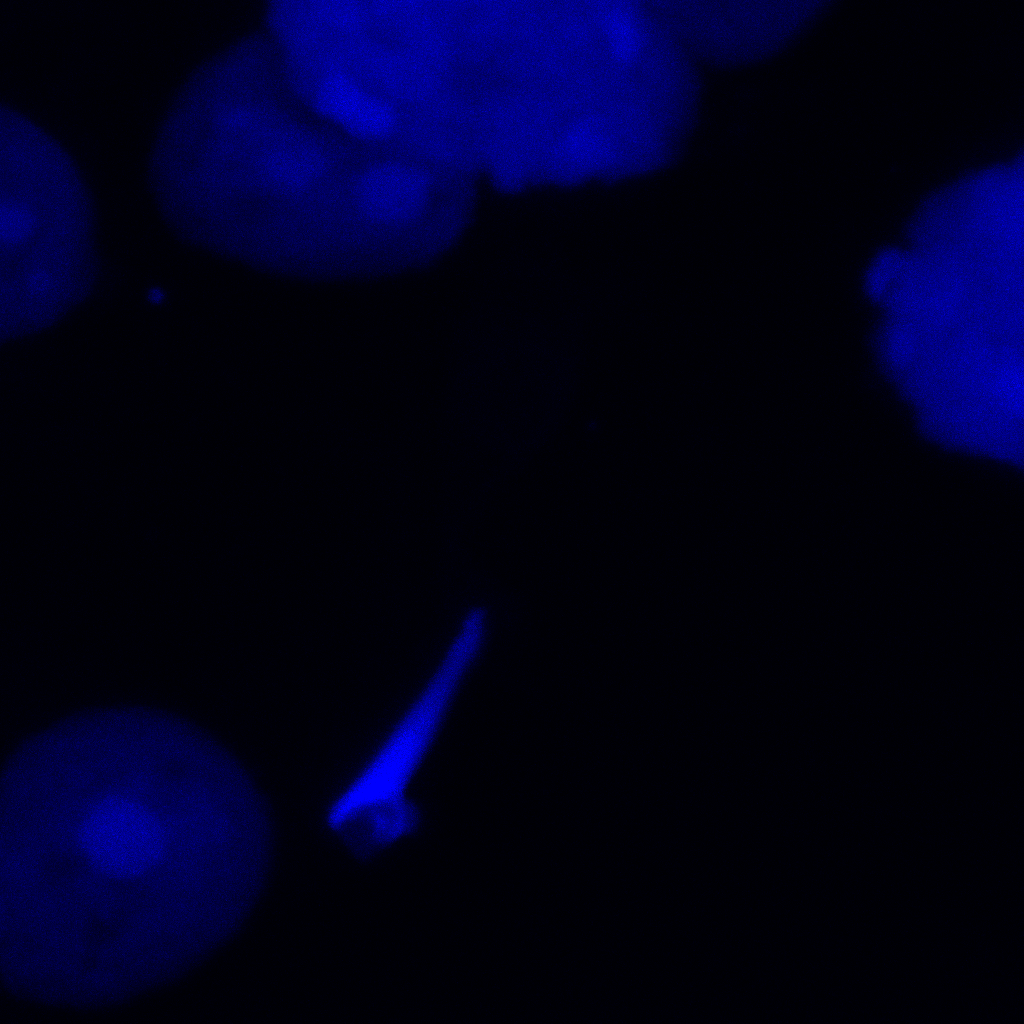

Supplement: Supplementary file 15 [file Data_Sheet_12.ZIP › Fig6H/KO/cfap53 ko IFT88 TRITC M ACTUB.lif_15_Processed001_ch00.tif]

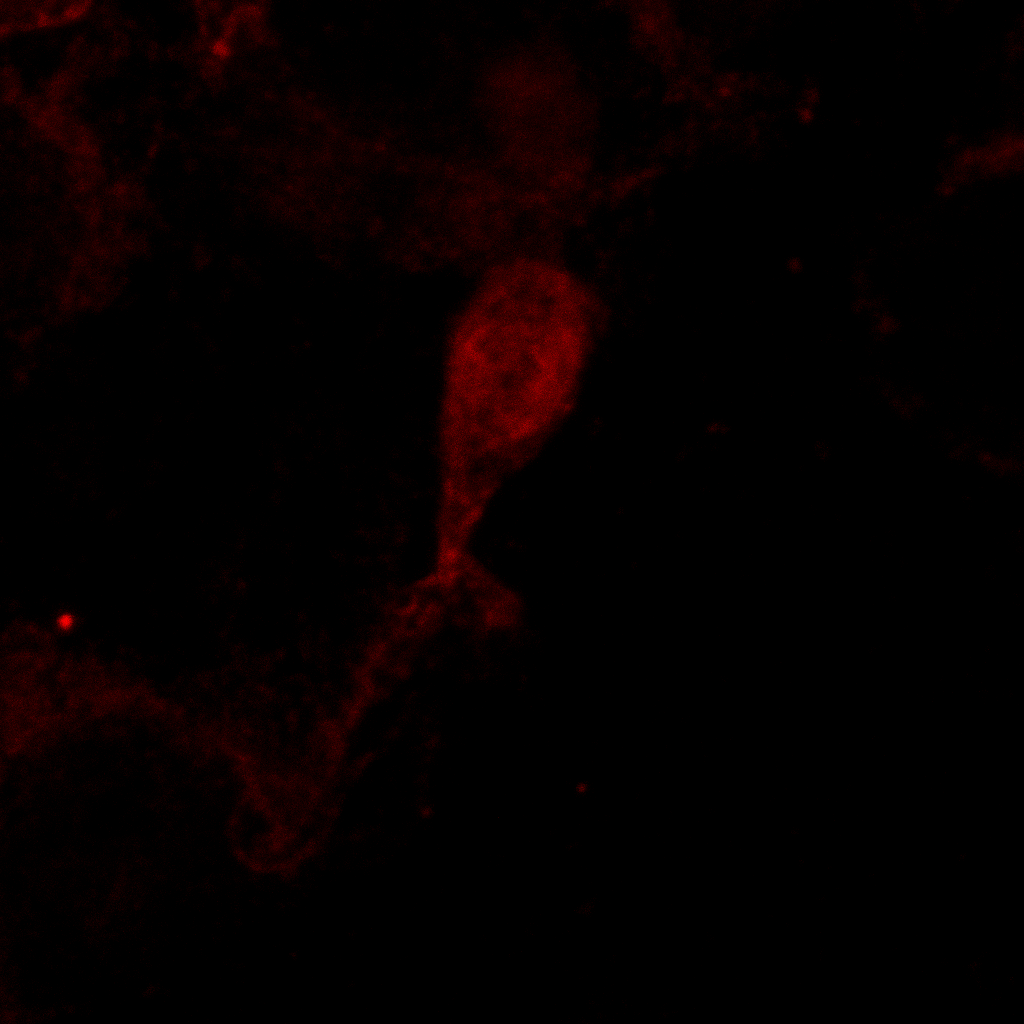

Supplement: Supplementary file 15 [file Data_Sheet_12.ZIP › Fig6H/KO/cfap53 ko IFT88 TRITC M ACTUB.lif_15_Processed001_ch02.tif]

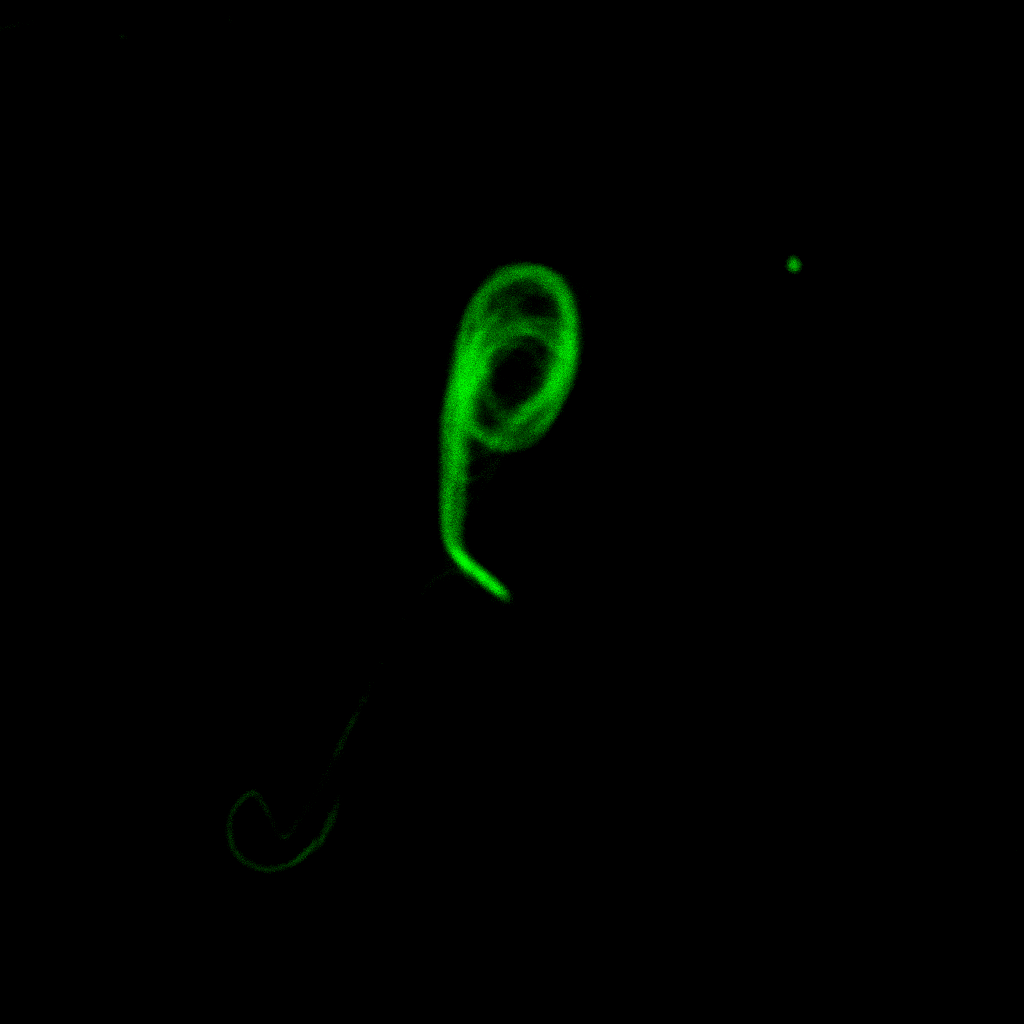

Supplement: Supplementary file 15 [file Data_Sheet_12.ZIP › Fig6H/KO/cfap53 ko IFT88 TRITC M ACTUB.lif_15_Processed001_ch03.tif]

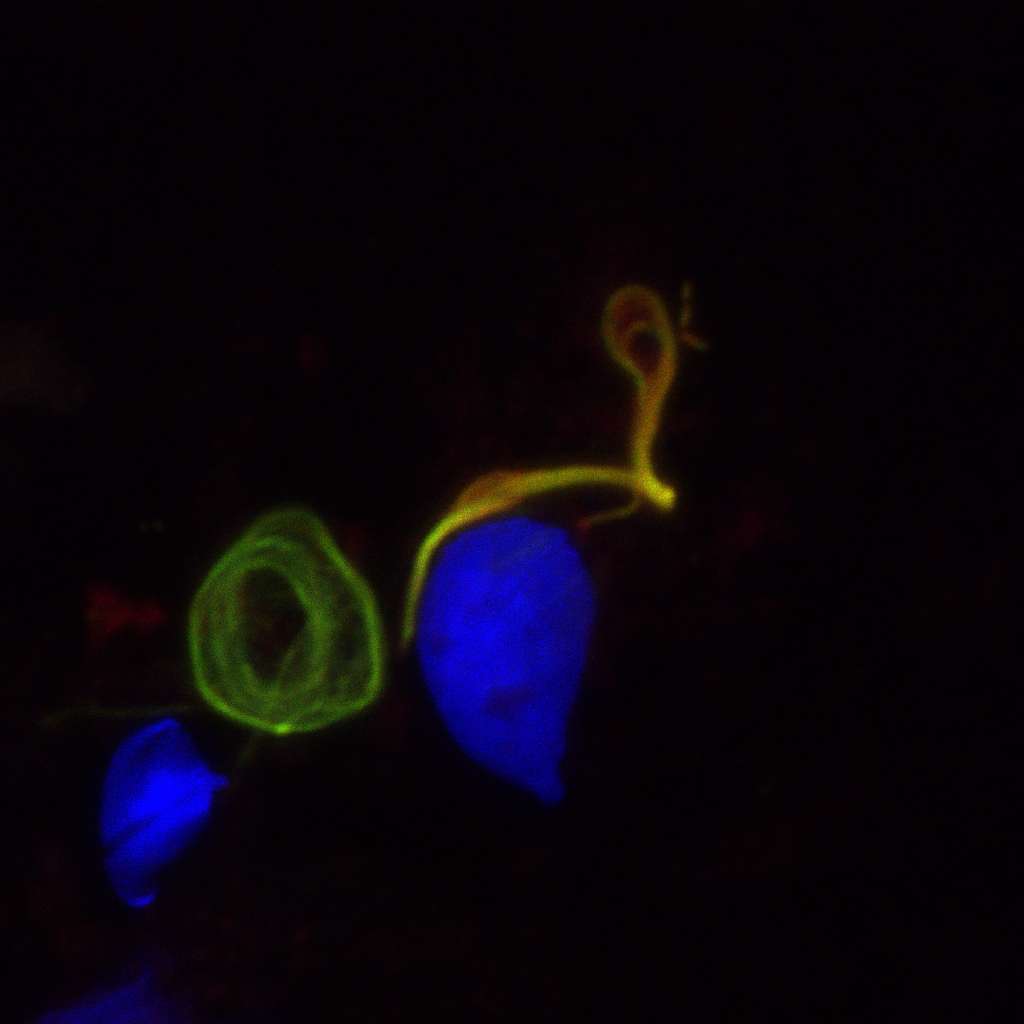

Supplement: Supplementary file 15 [file Data_Sheet_12.ZIP › Fig6H/KO/cfap53 ko IFT88 TRITC M ACTUB.lif_9 2_Processed001.tif]

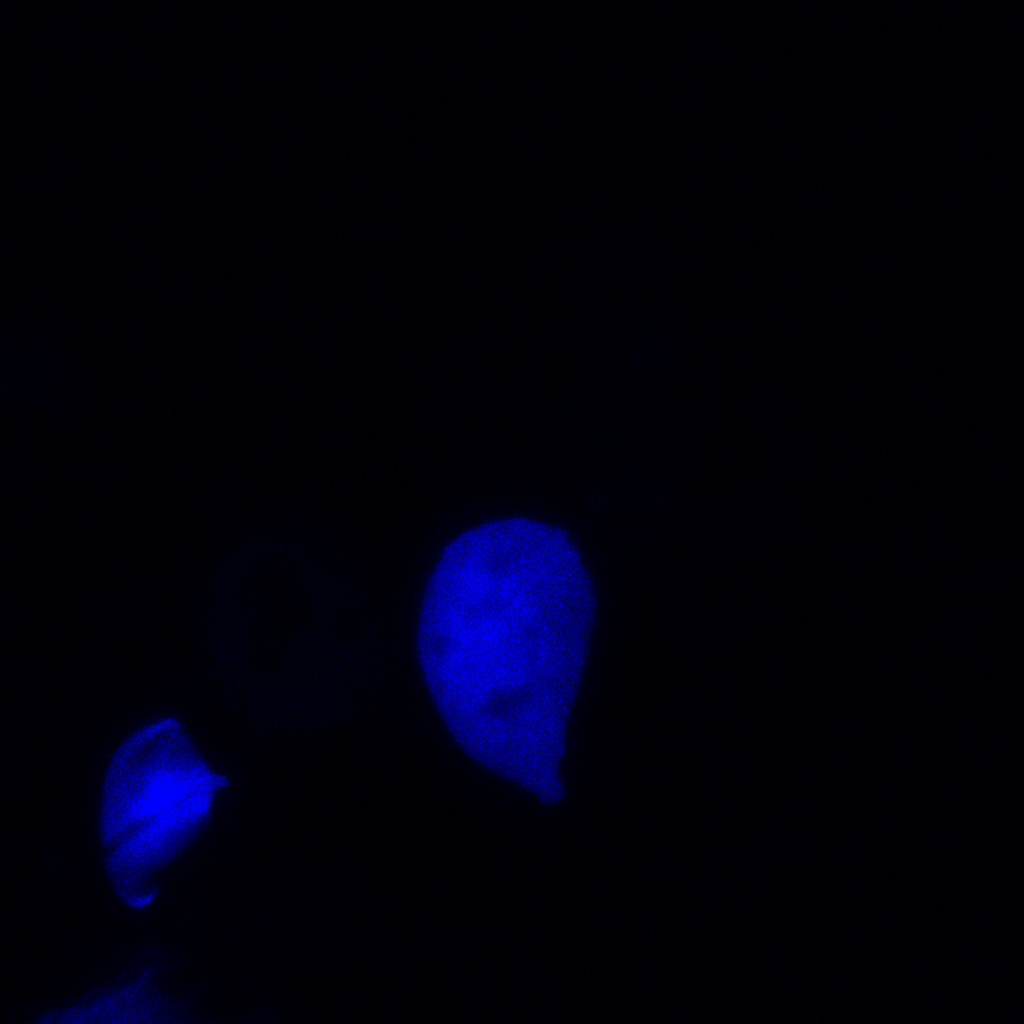

Supplement: Supplementary file 15 [file Data_Sheet_12.ZIP › Fig6H/KO/cfap53 ko IFT88 TRITC M ACTUB.lif_9 2_Processed001_ch00.tif]

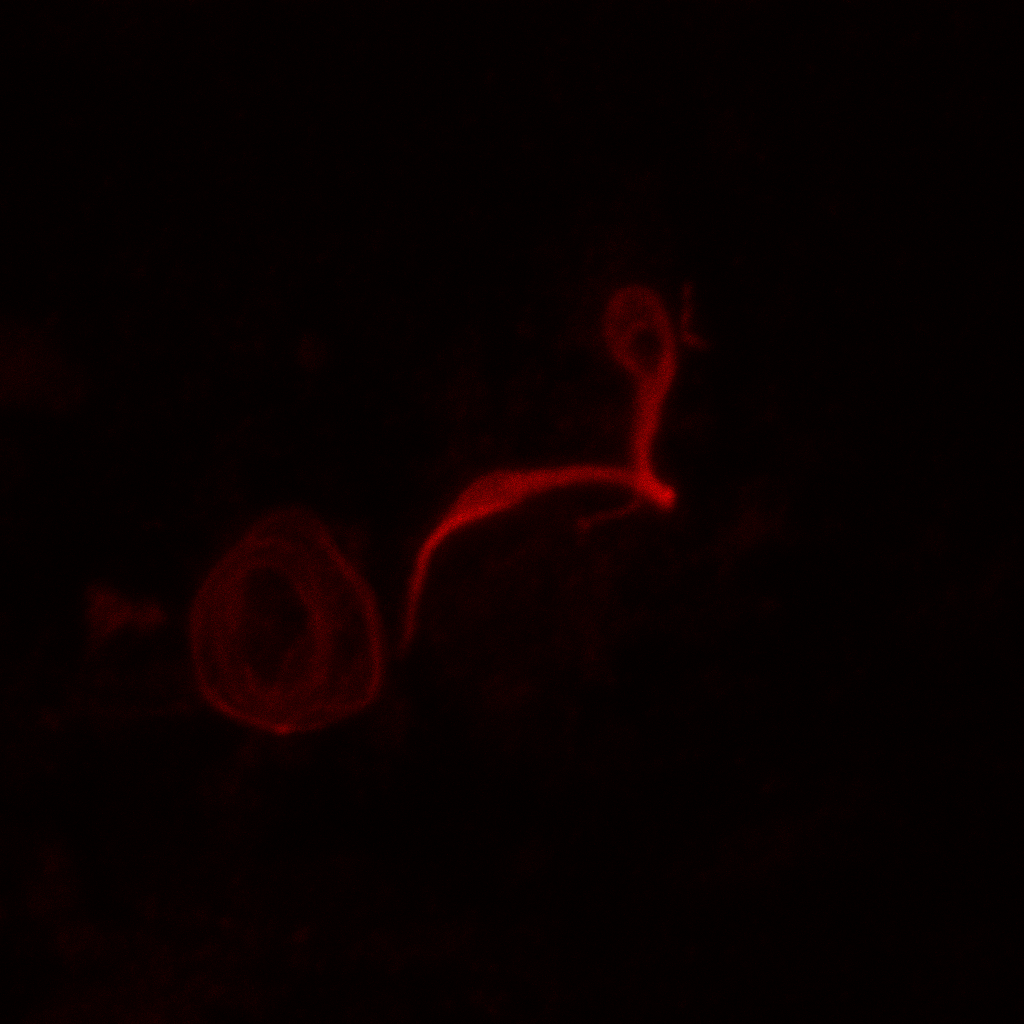

Supplement: Supplementary file 15 [file Data_Sheet_12.ZIP › Fig6H/KO/cfap53 ko IFT88 TRITC M ACTUB.lif_9 2_Processed001_ch02.tif]

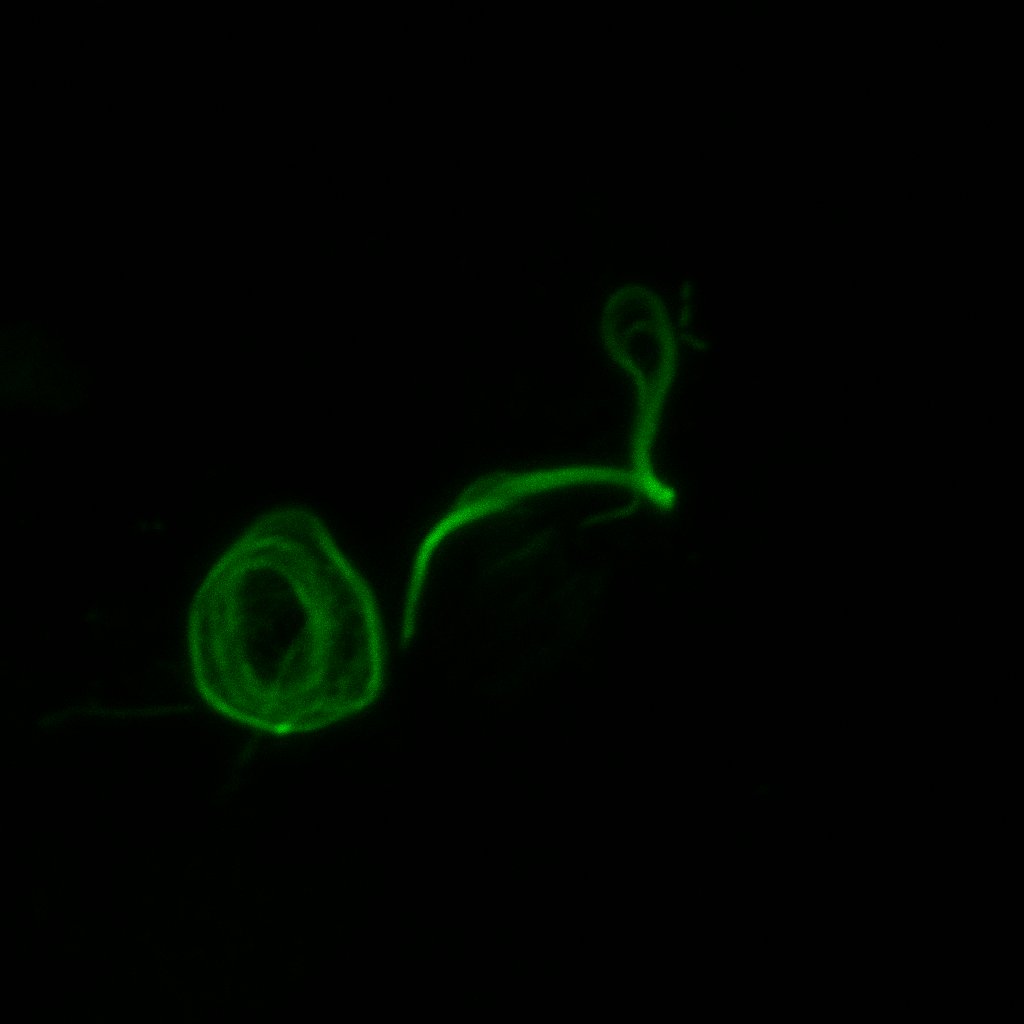

Supplement: Supplementary file 15 [file Data_Sheet_12.ZIP › Fig6H/KO/cfap53 ko IFT88 TRITC M ACTUB.lif_9 2_Processed001_ch03.tif]

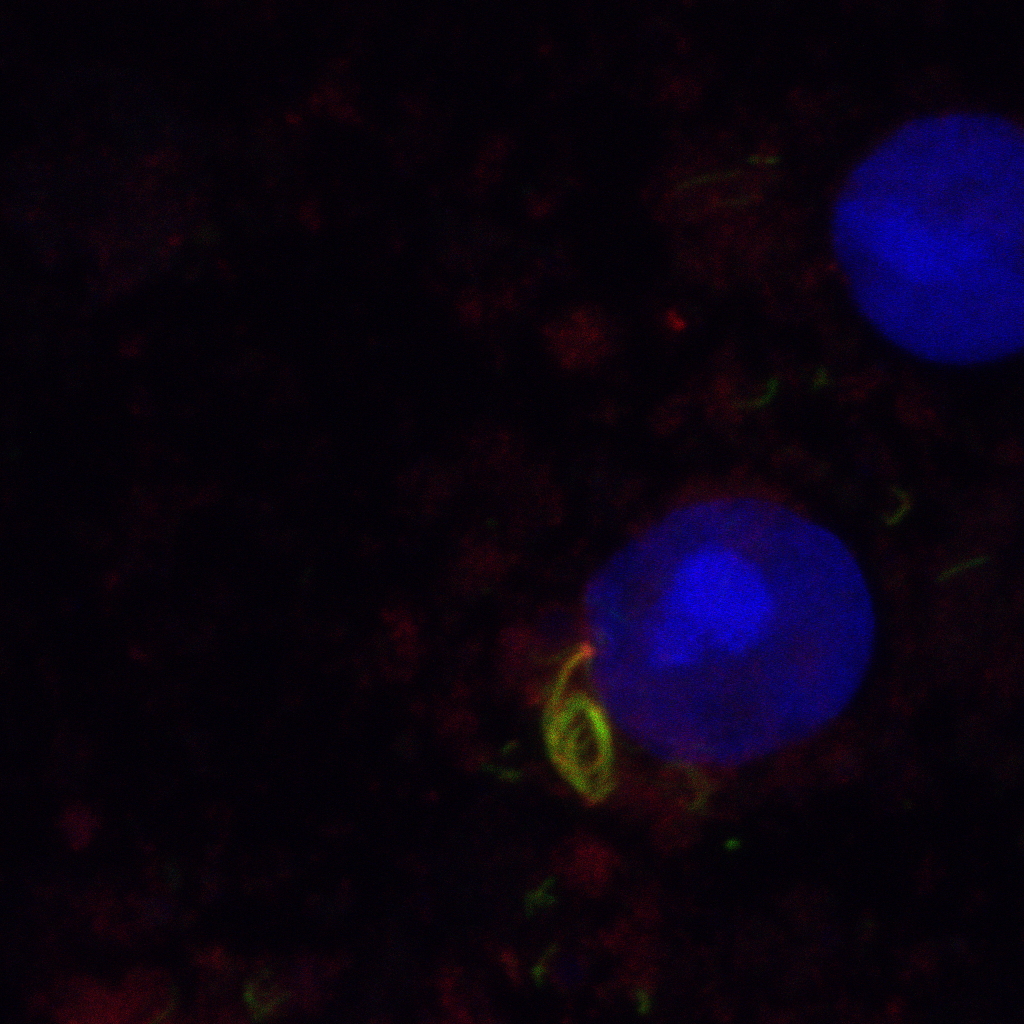

Supplement: Supplementary file 15 [file Data_Sheet_12.ZIP › Fig6H/KO/cfap53 ko IFT88 TRITC M ACTUB.lif_ROUND BESR_Processed001.tif]

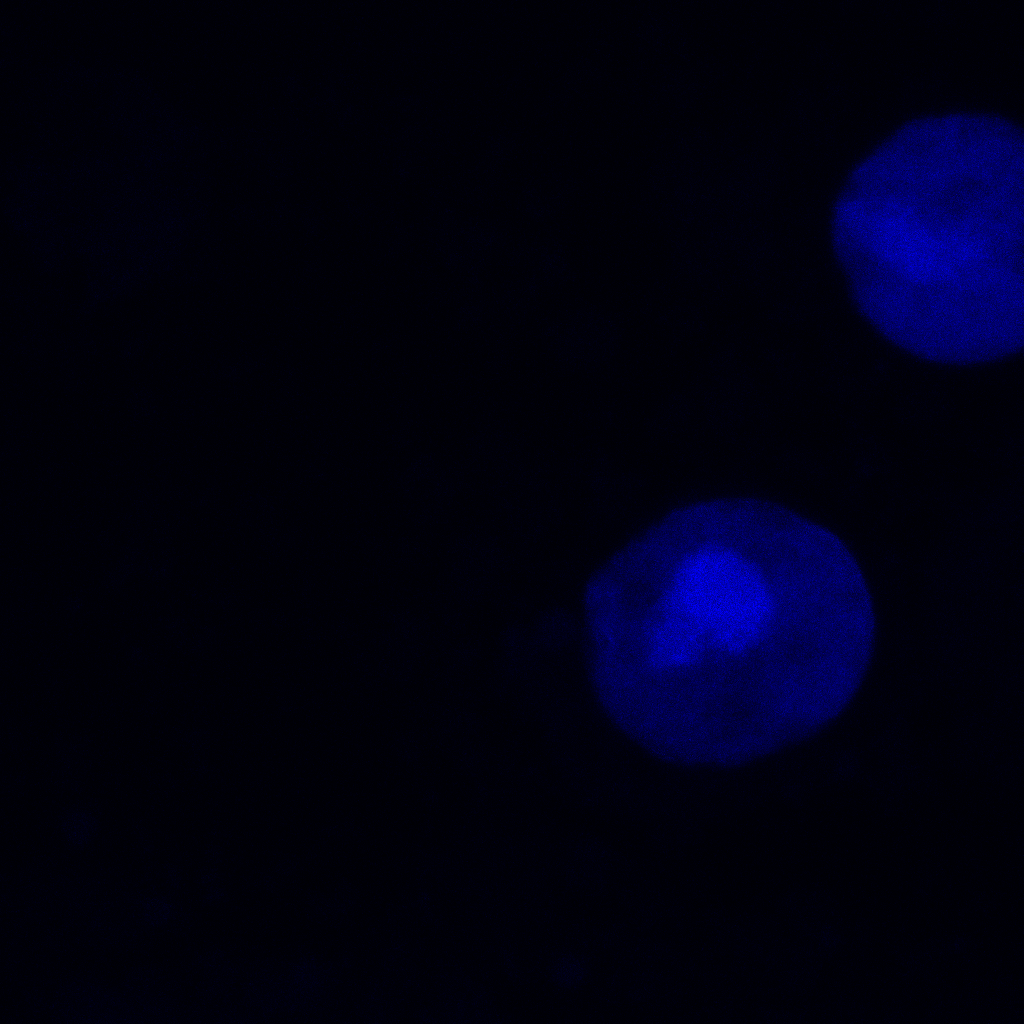

Supplement: Supplementary file 15 [file Data_Sheet_12.ZIP › Fig6H/KO/cfap53 ko IFT88 TRITC M ACTUB.lif_ROUND BESR_Processed001_ch00.tif]

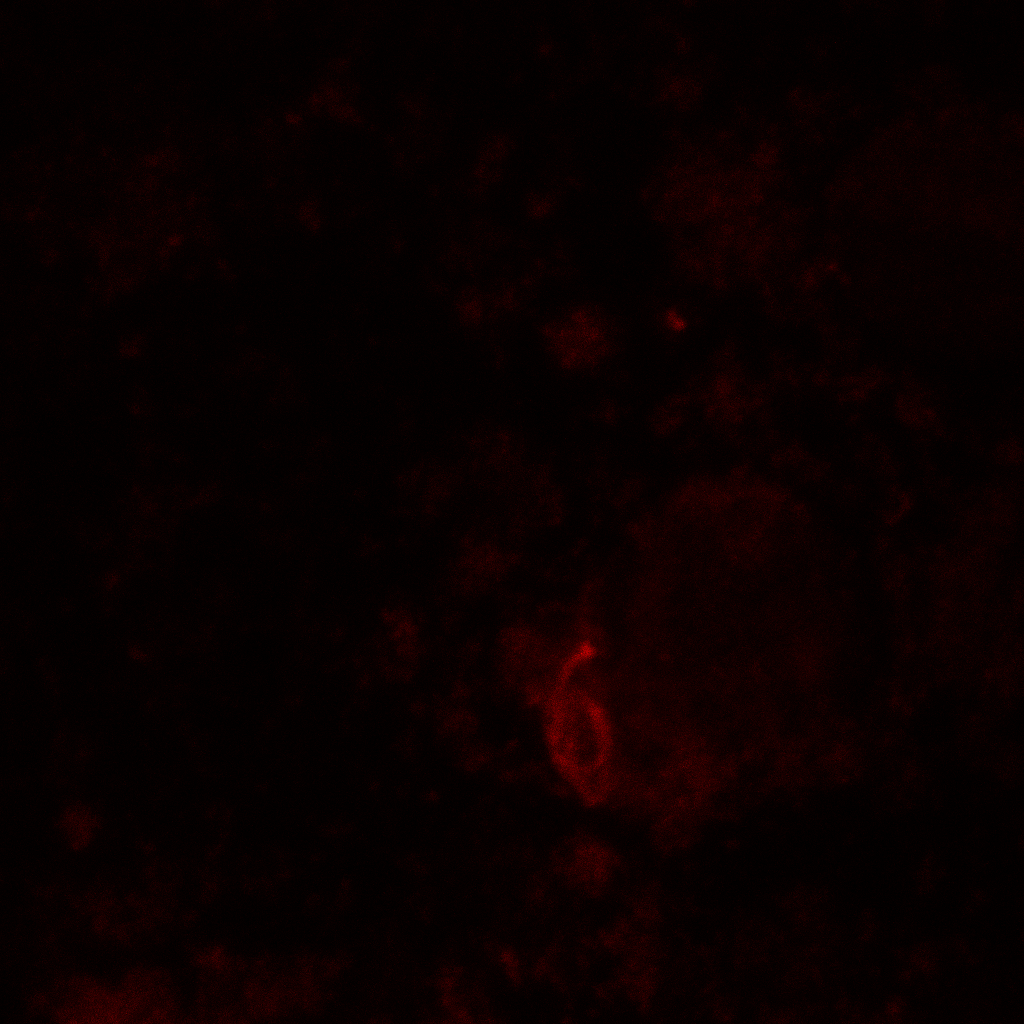

Supplement: Supplementary file 15 [file Data_Sheet_12.ZIP › Fig6H/KO/cfap53 ko IFT88 TRITC M ACTUB.lif_ROUND BESR_Processed001_ch02.tif]

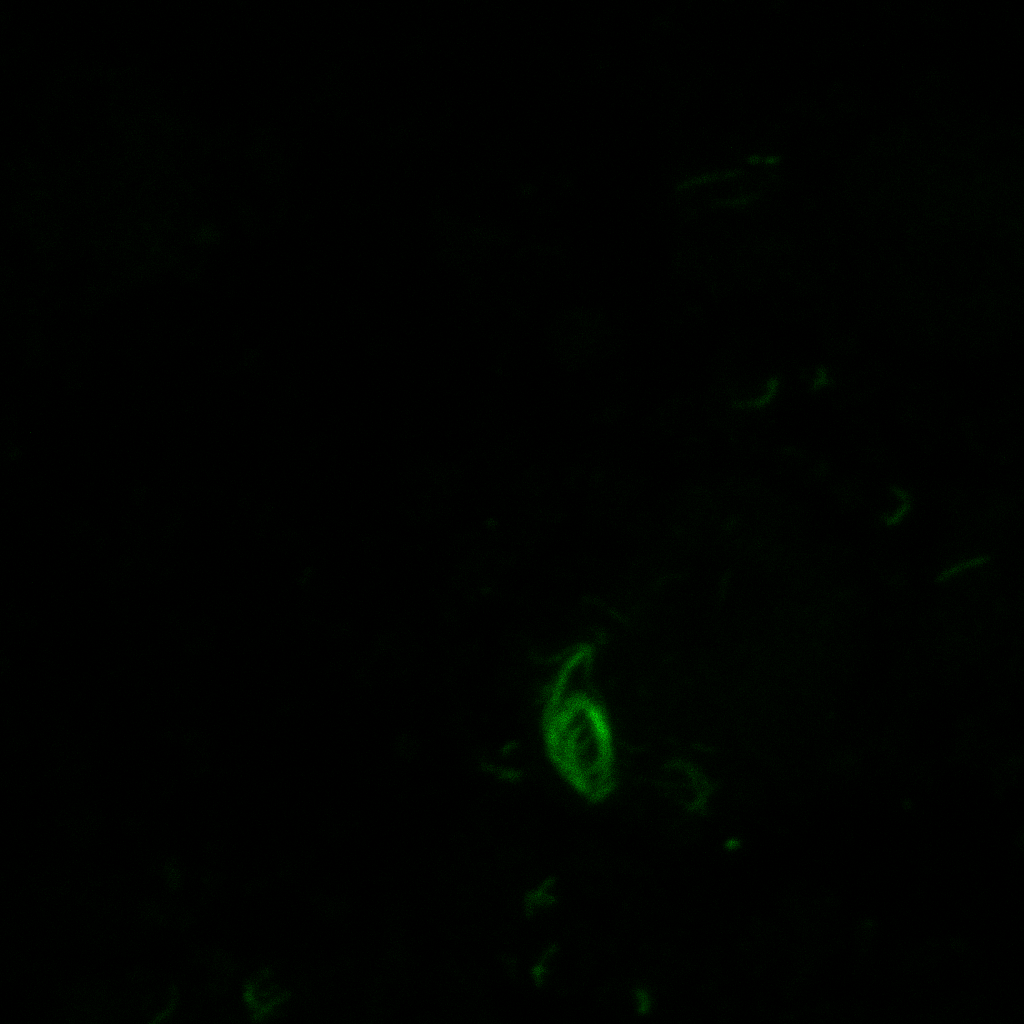

Supplement: Supplementary file 15 [file Data_Sheet_12.ZIP › Fig6H/KO/cfap53 ko IFT88 TRITC M ACTUB.lif_ROUND BESR_Processed001_ch03.tif]

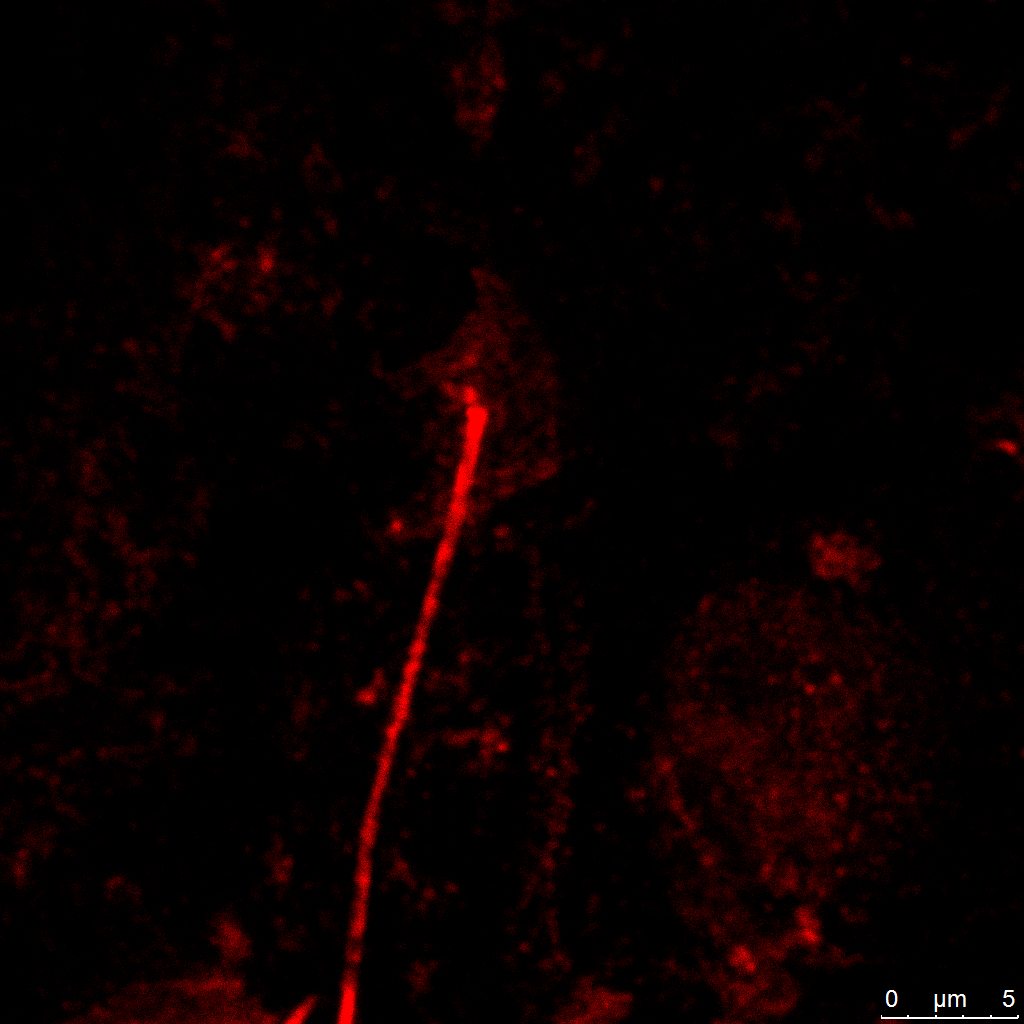

Supplement: Supplementary file 15 [file Data_Sheet_12.ZIP › Fig6H/WT/cfap53 wt ift88 tritc tubulin 13-14 BEST_z3_ch02.tif]

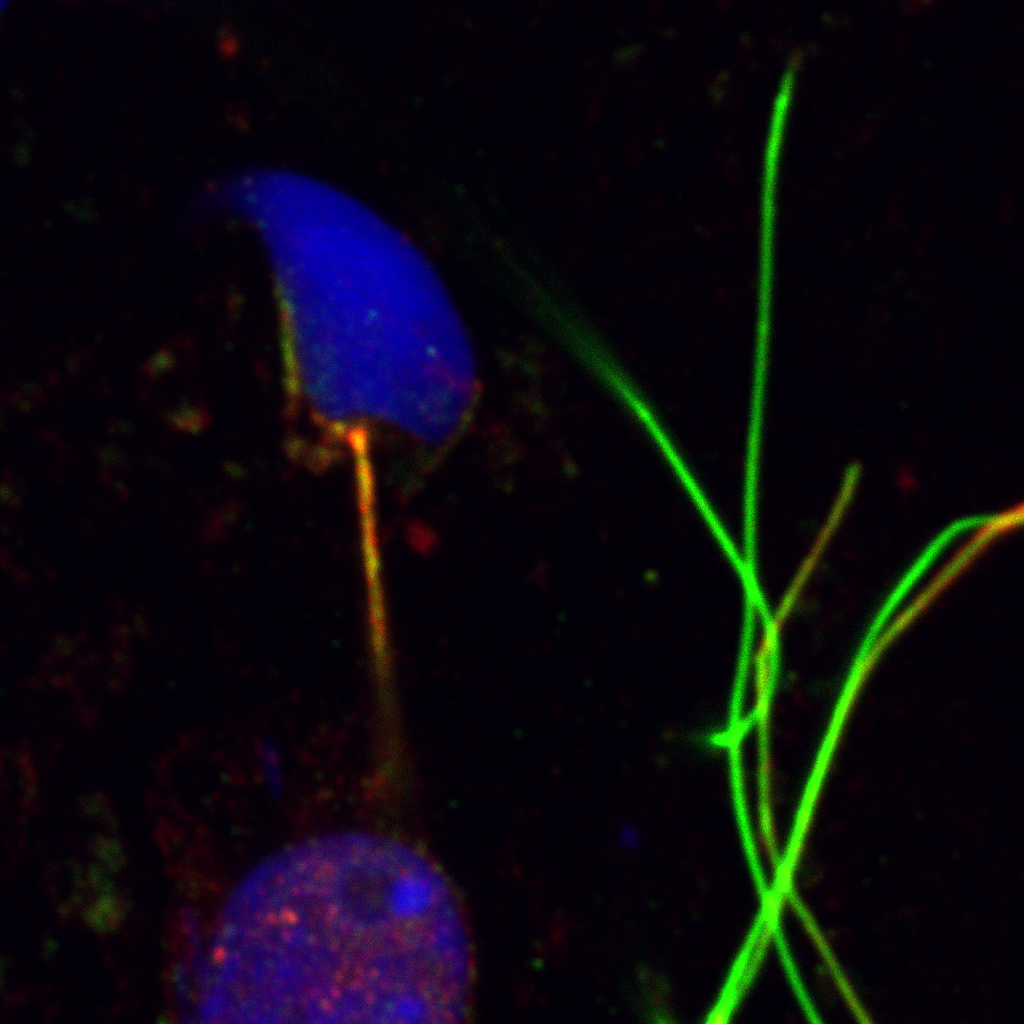

Supplement: Supplementary file 15 [file Data_Sheet_12.ZIP › Fig6H/WT/cfap53 wt ift88 tritc tubulin cy5.lif_11 2 BEST_Processed001.tif]

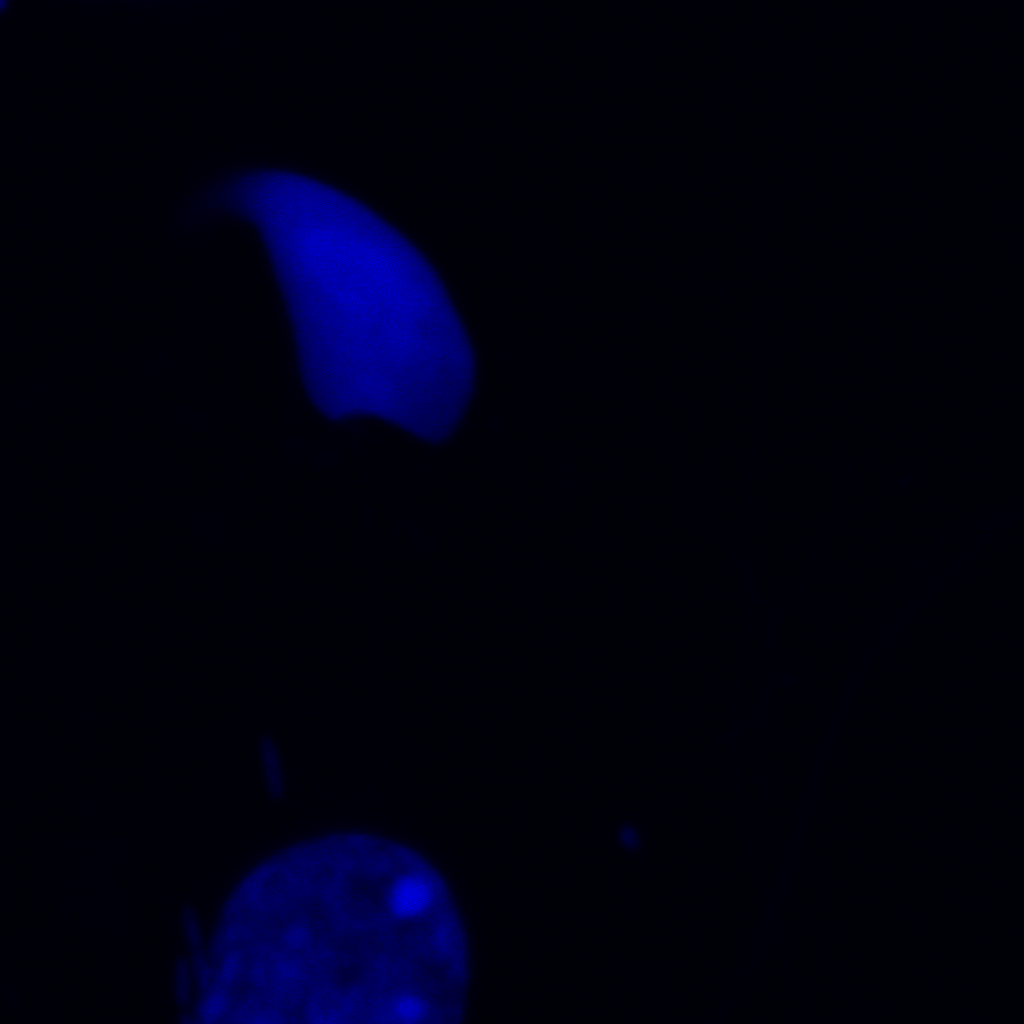

Supplement: Supplementary file 15 [file Data_Sheet_12.ZIP › Fig6H/WT/cfap53 wt ift88 tritc tubulin cy5.lif_11 2 BEST_Processed001_ch00.tif]

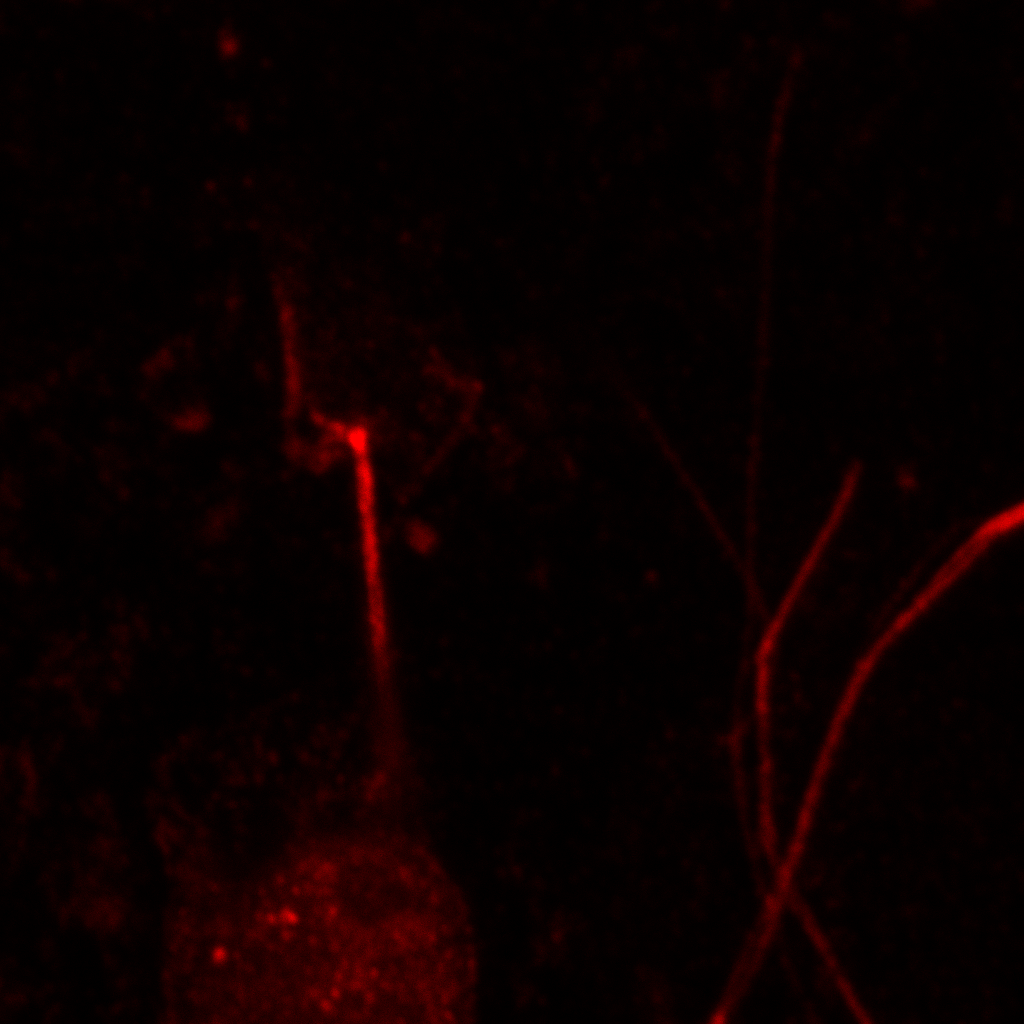

Supplement: Supplementary file 15 [file Data_Sheet_12.ZIP › Fig6H/WT/cfap53 wt ift88 tritc tubulin cy5.lif_11 2 BEST_Processed001_ch02.tif]
