# Supplementary figures and images for: Essential Role of CFAP53 in Sperm Flagellum Biogenesis (part 3 of 3)
Source: Front Cell Dev Biol. 2021 May 28;9:676910. doi: 10.3389/fcell.2021.676910 (PMC8195676; doi:10.3389/fcell.2021.676910)

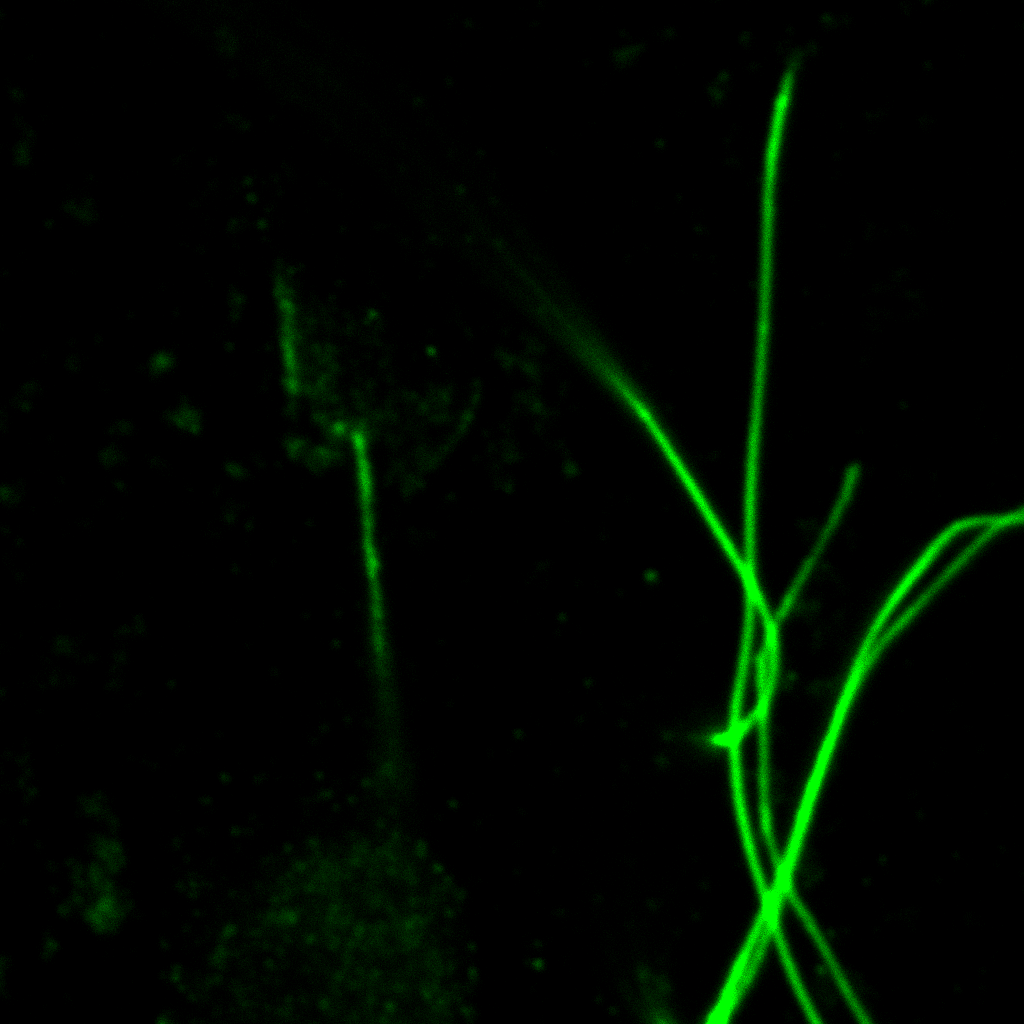

Supplement: Supplementary file 15 [file Data_Sheet_12.ZIP › Fig6H/WT/cfap53 wt ift88 tritc tubulin cy5.lif_11 2 BEST_Processed001_ch03.tif]

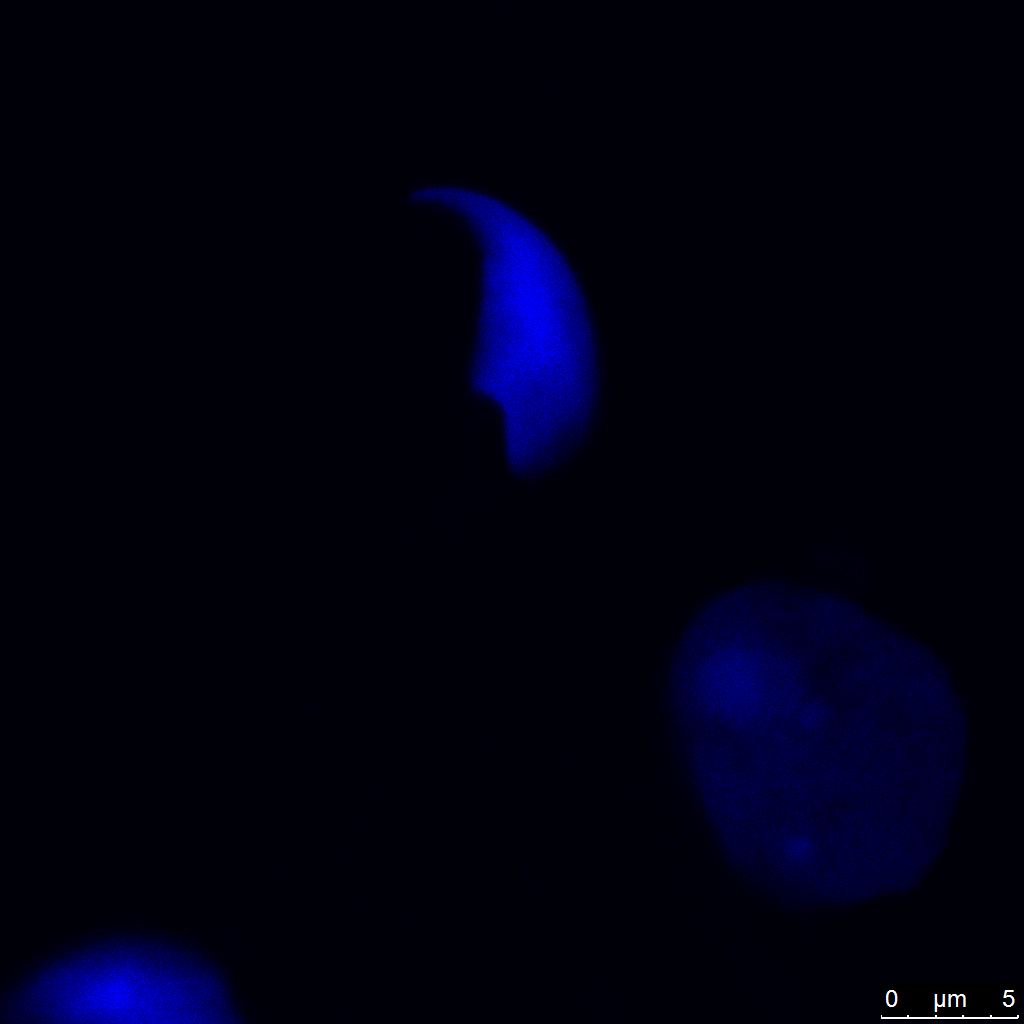

Supplement: Supplementary file 15 [file Data_Sheet_12.ZIP › Fig6H/WT/cfap53 wt ift88 tritc tubulin cy5.lif_13-14 BEST_z3_ch00.tif]

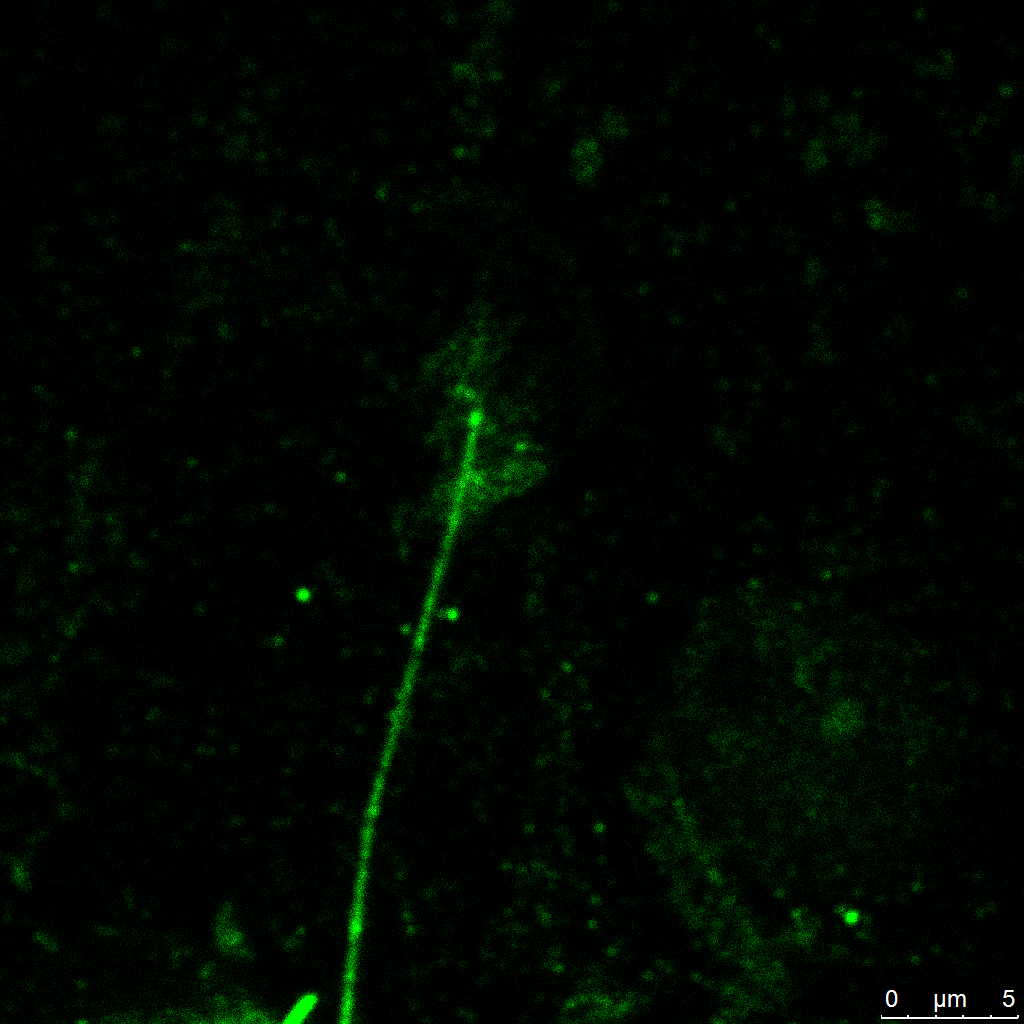

Supplement: Supplementary file 15 [file Data_Sheet_12.ZIP › Fig6H/WT/cfap53 wt ift88 tritc tubulin cy5.lif_13-14 BEST_z3_ch03.tif]

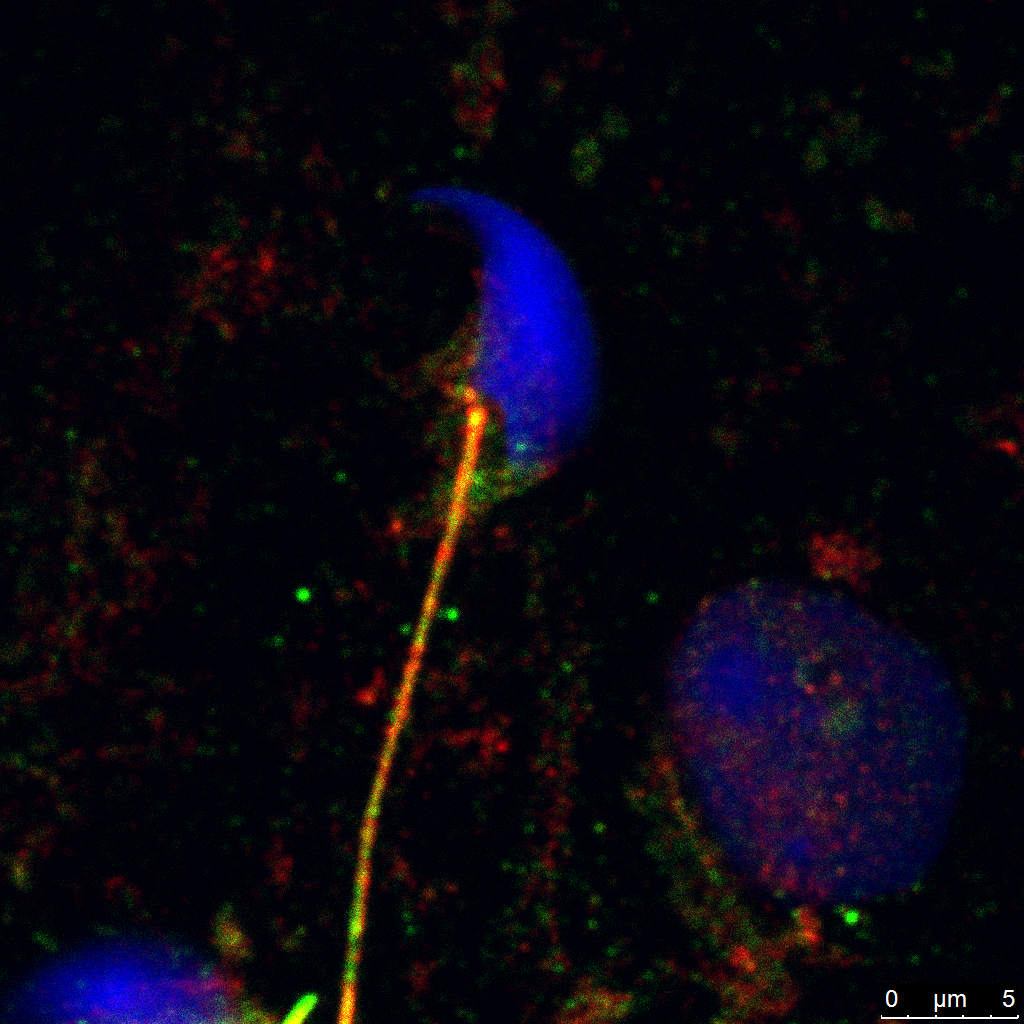

Supplement: Supplementary file 15 [file Data_Sheet_12.ZIP › Fig6H/WT/cfap53 wt ift88 tritc tubulin cy5.lif_13-14BEST_z3.tif]

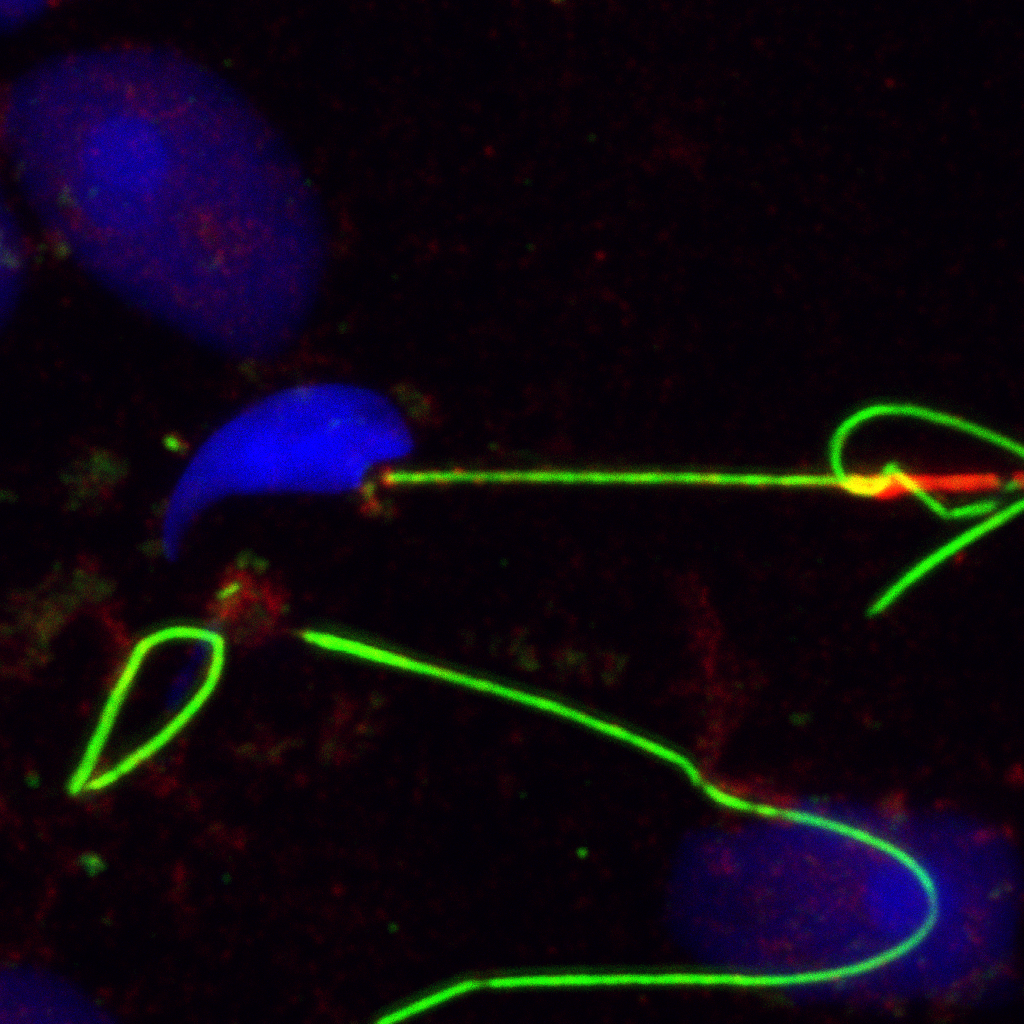

Supplement: Supplementary file 15 [file Data_Sheet_12.ZIP › Fig6H/WT/cfap53 wt ift88 tritc tubulin cy5.lif_15-16_Processed001.tif]

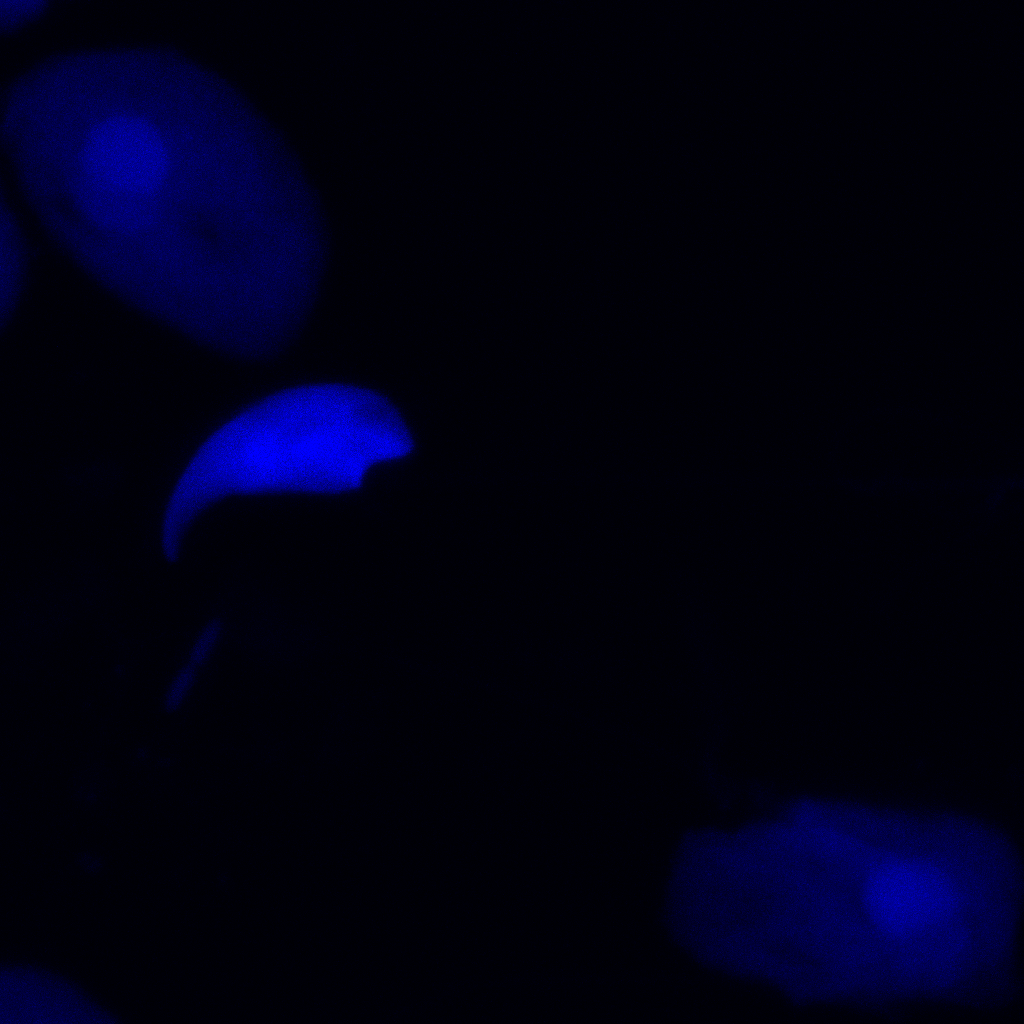

Supplement: Supplementary file 15 [file Data_Sheet_12.ZIP › Fig6H/WT/cfap53 wt ift88 tritc tubulin cy5.lif_15-16_Processed001_ch00.tif]

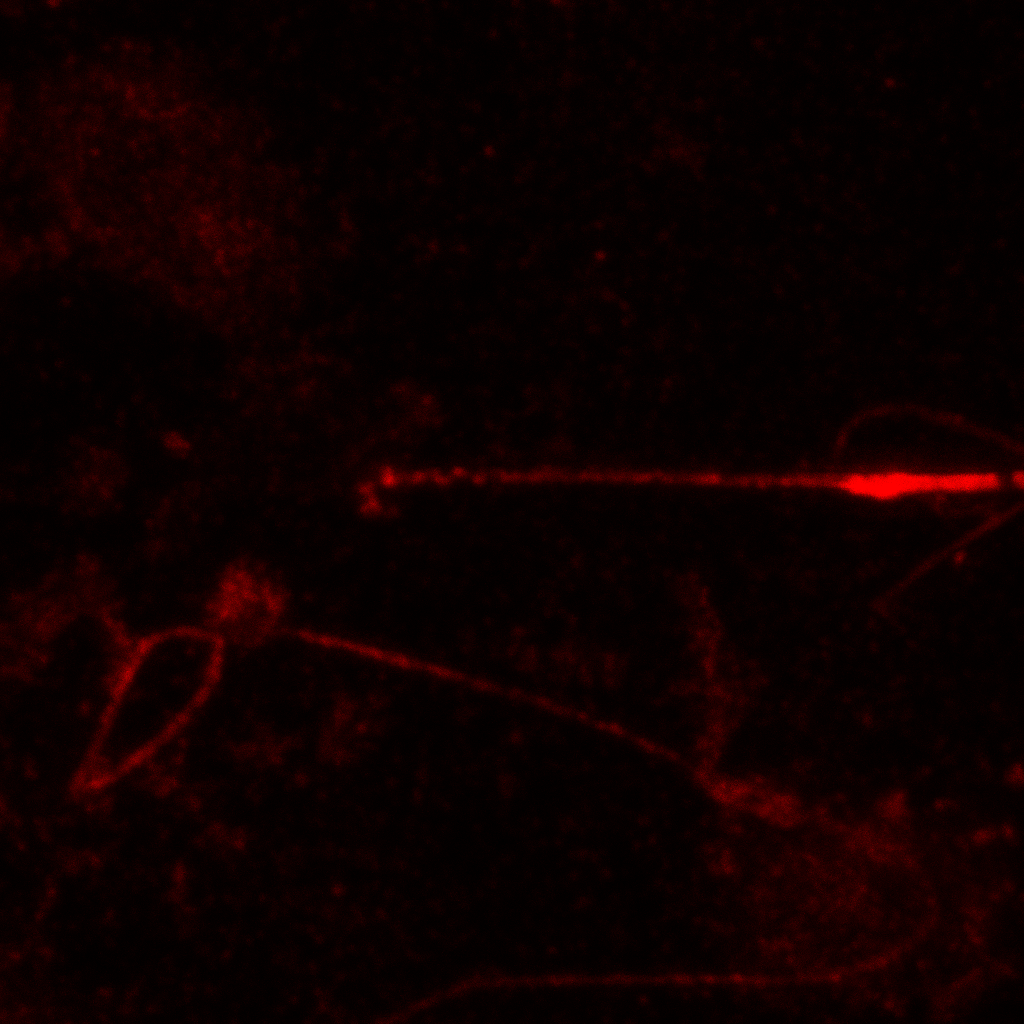

Supplement: Supplementary file 15 [file Data_Sheet_12.ZIP › Fig6H/WT/cfap53 wt ift88 tritc tubulin cy5.lif_15-16_Processed001_ch02.tif]

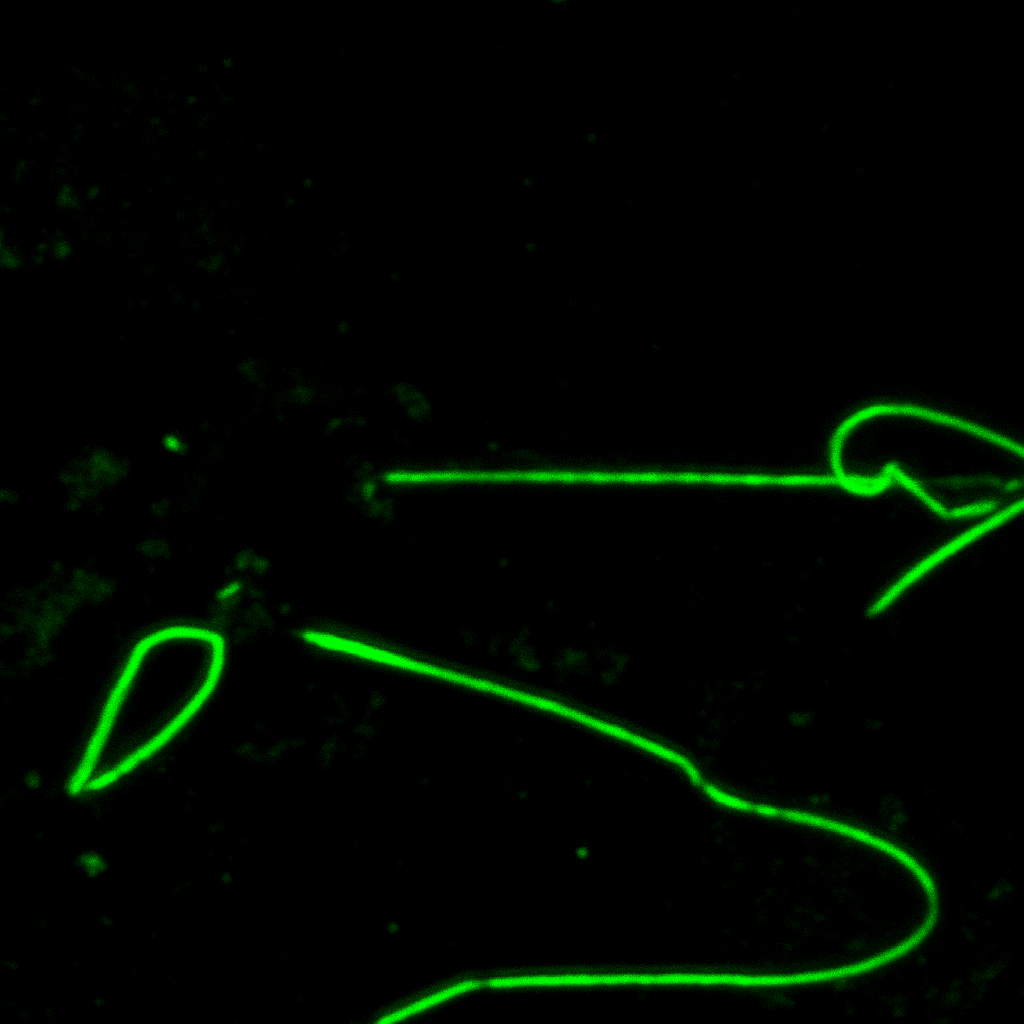

Supplement: Supplementary file 15 [file Data_Sheet_12.ZIP › Fig6H/WT/cfap53 wt ift88 tritc tubulin cy5.lif_15-16_Processed001_ch03.tif]

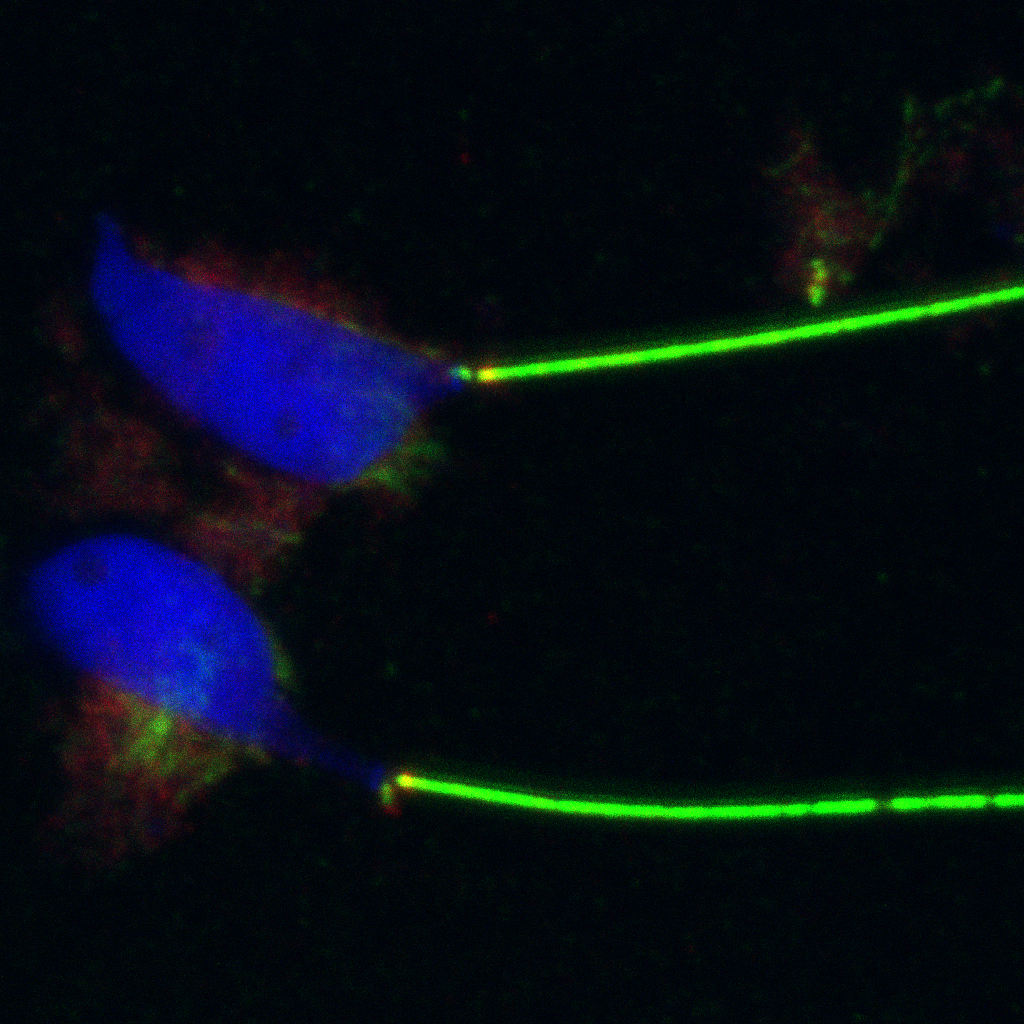

Supplement: Supplementary file 15 [file Data_Sheet_12.ZIP › Fig6H/WT/cfap53 wt ift88 tritc tubulin cy5.lif_9-10_Processed001.tif]

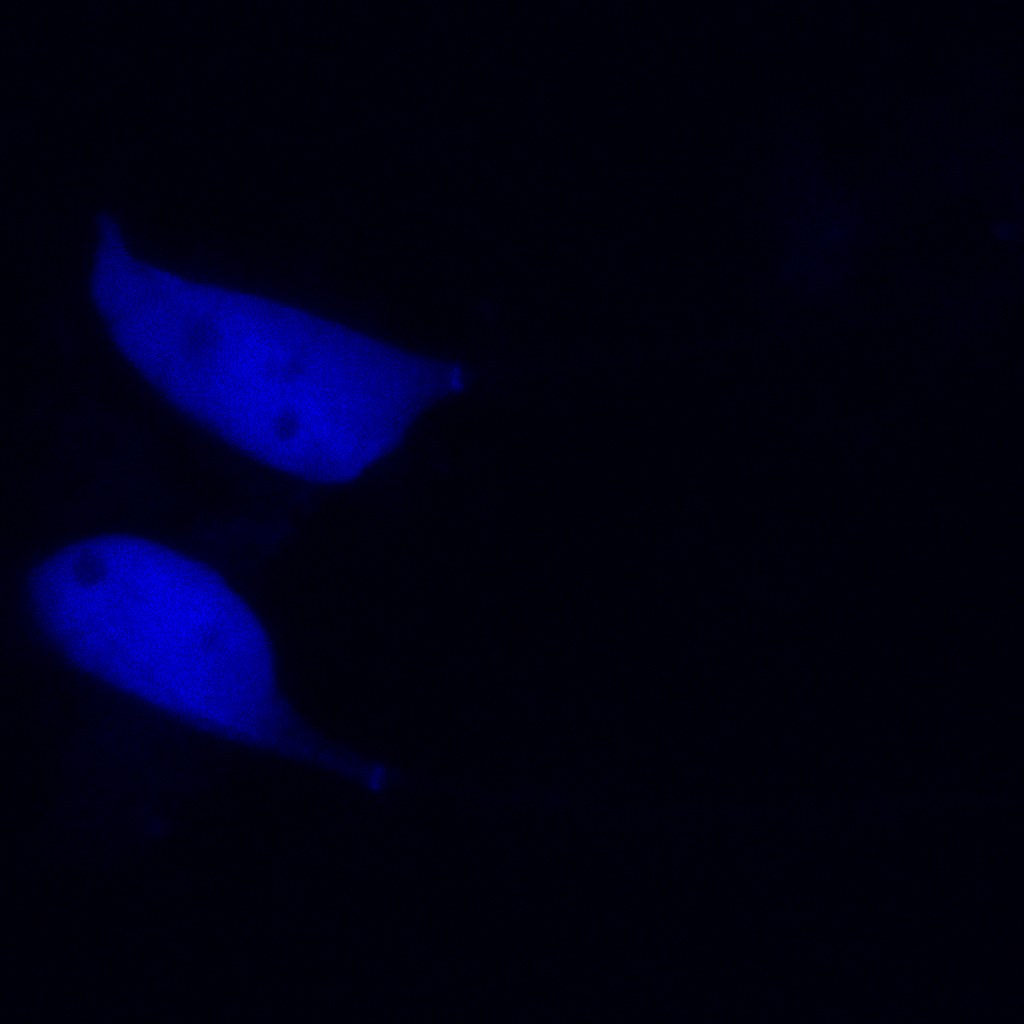

Supplement: Supplementary file 15 [file Data_Sheet_12.ZIP › Fig6H/WT/cfap53 wt ift88 tritc tubulin cy5.lif_9-10_Processed001_ch00.tif]

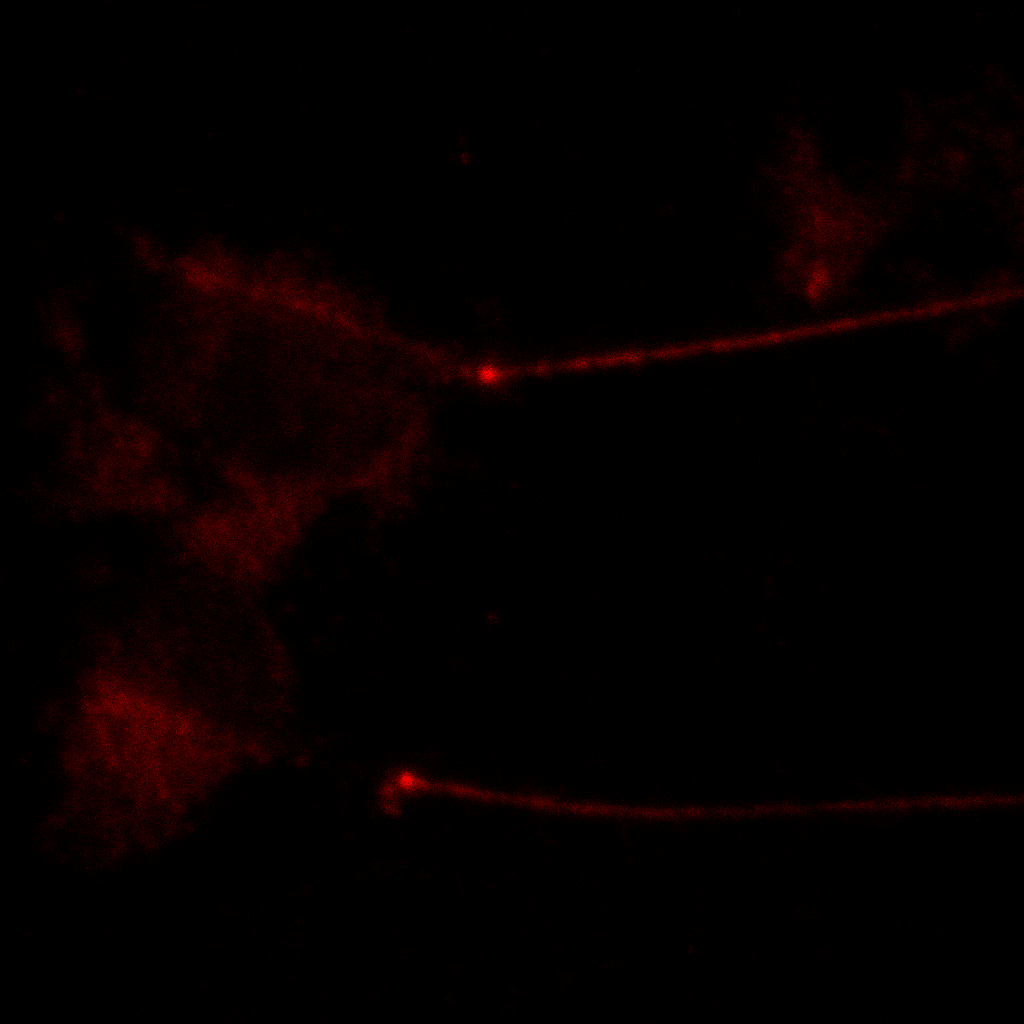

Supplement: Supplementary file 15 [file Data_Sheet_12.ZIP › Fig6H/WT/cfap53 wt ift88 tritc tubulin cy5.lif_9-10_Processed001_ch02.tif]

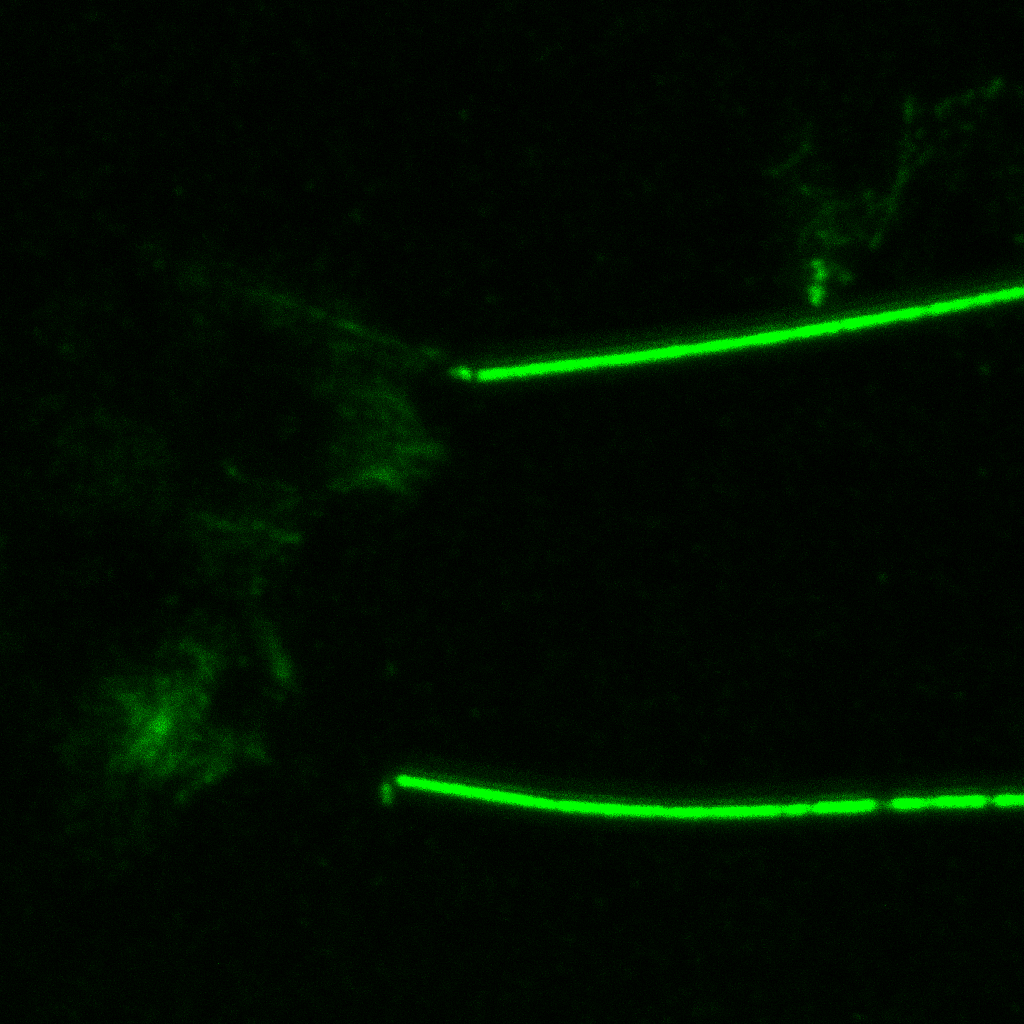

Supplement: Supplementary file 15 [file Data_Sheet_12.ZIP › Fig6H/WT/cfap53 wt ift88 tritc tubulin cy5.lif_9-10_Processed001_ch03.tif]

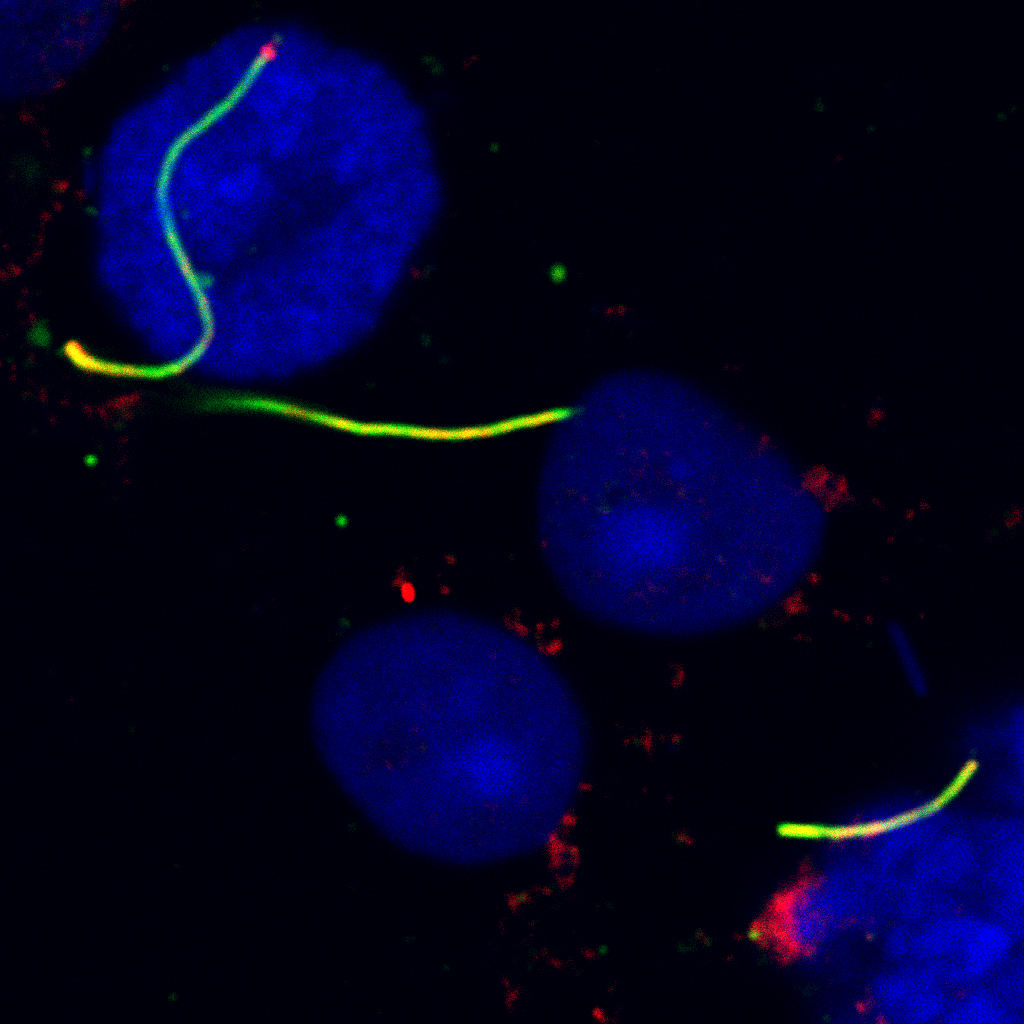

Supplement: Supplementary file 15 [file Data_Sheet_12.ZIP › Fig6H/WT/cfap53 wt ift88 tritc tubulin cy5.lif_ROUND SPERM BEST_z3.tif]

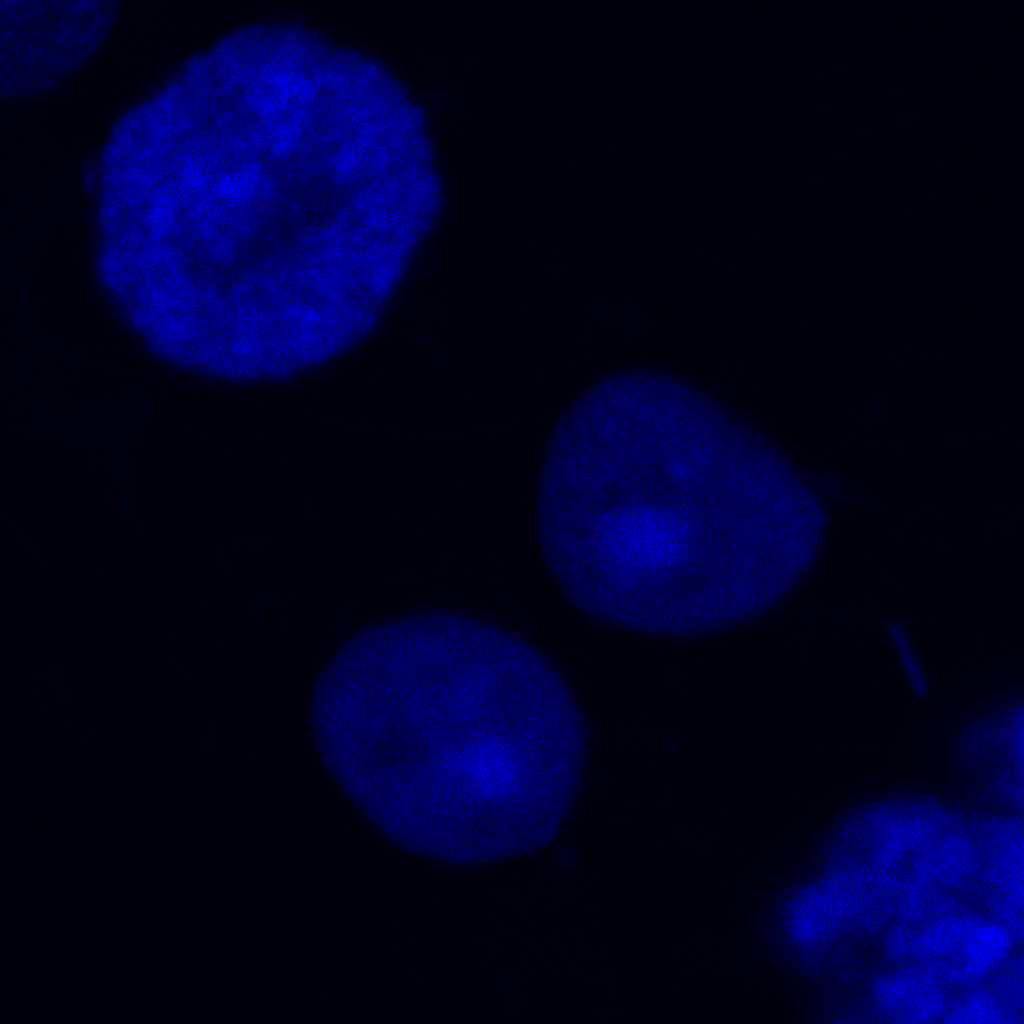

Supplement: Supplementary file 15 [file Data_Sheet_12.ZIP › Fig6H/WT/cfap53 wt ift88 tritc tubulin cy5.lif_ROUND SPERM BEST_z3_ch00.tif]

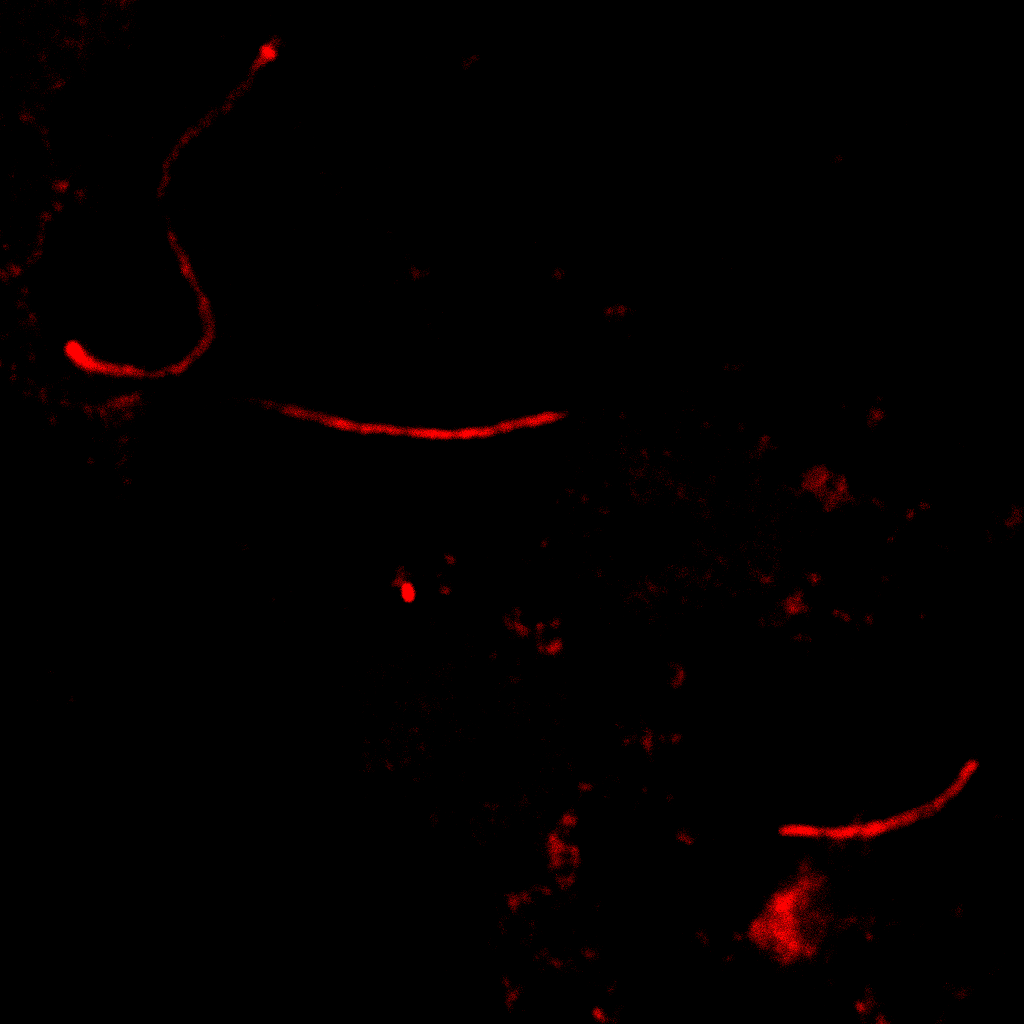

Supplement: Supplementary file 15 [file Data_Sheet_12.ZIP › Fig6H/WT/cfap53 wt ift88 tritc tubulin cy5.lif_ROUND SPERM BEST_z3_ch02.tif]

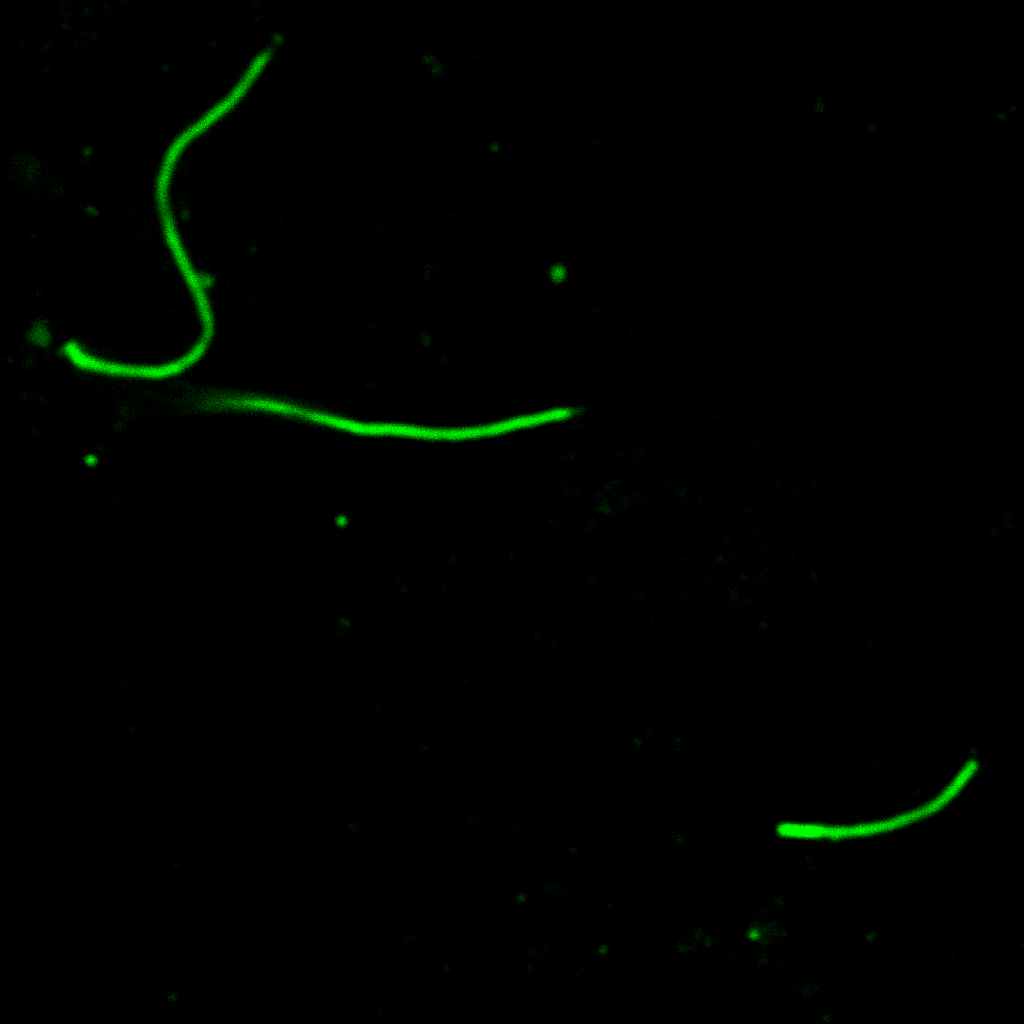

Supplement: Supplementary file 15 [file Data_Sheet_12.ZIP › Fig6H/WT/cfap53 wt ift88 tritc tubulin cy5.lif_ROUND SPERM BEST_z3_ch03.tif]

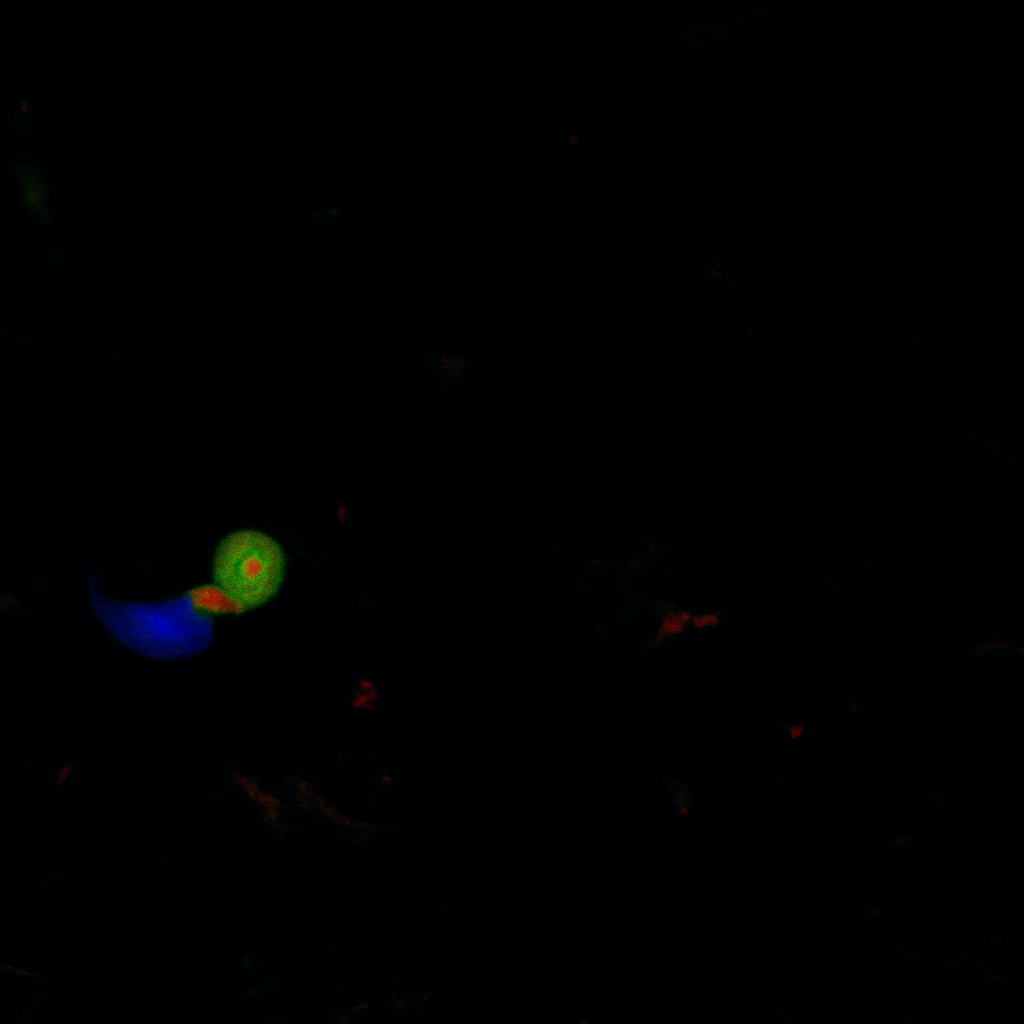

Supplement: Supplementary file 16 [file Data_Sheet_13.ZIP › original microscopy Supplementary Figure 2/KO/tublin fitc mitotracker red .lif_Series034_z0.tif]

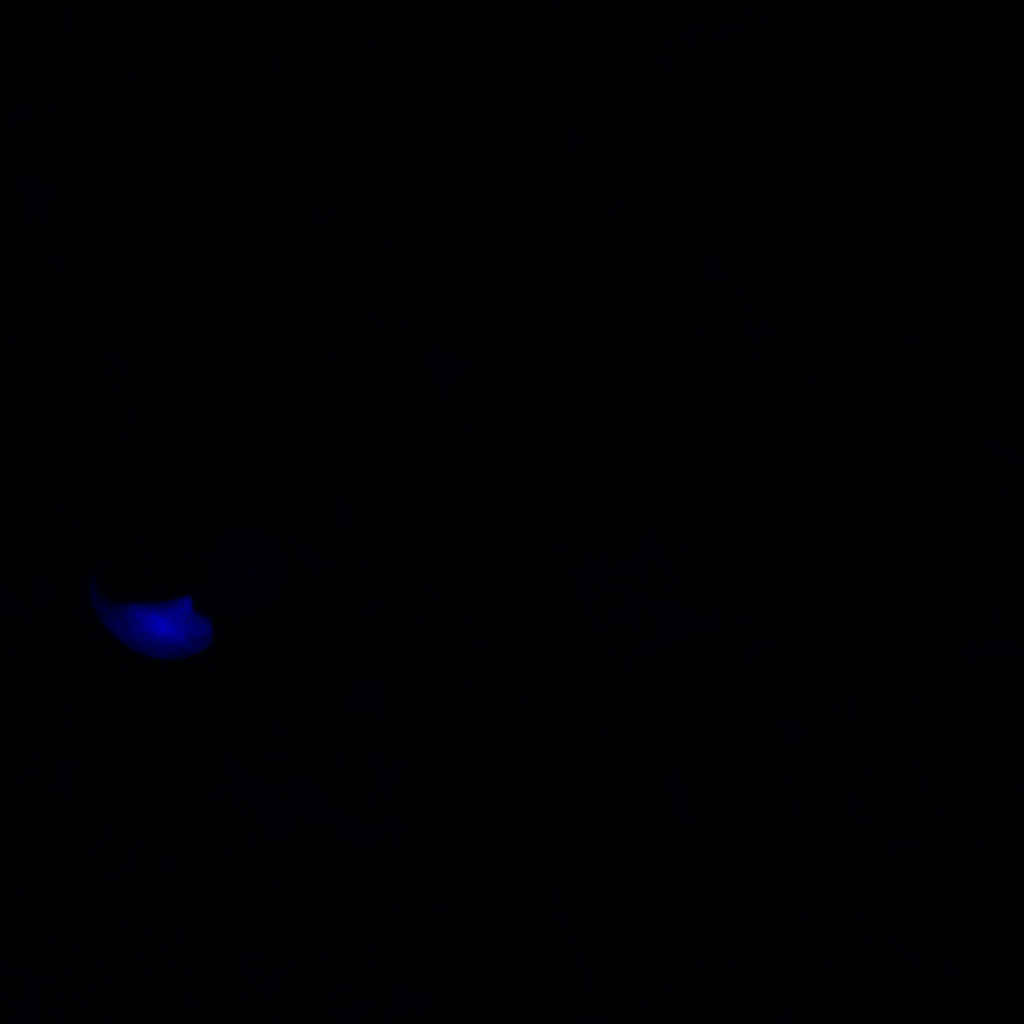

Supplement: Supplementary file 16 [file Data_Sheet_13.ZIP › original microscopy Supplementary Figure 2/KO/tublin fitc mitotracker red .lif_Series034_z0_ch00.tif]

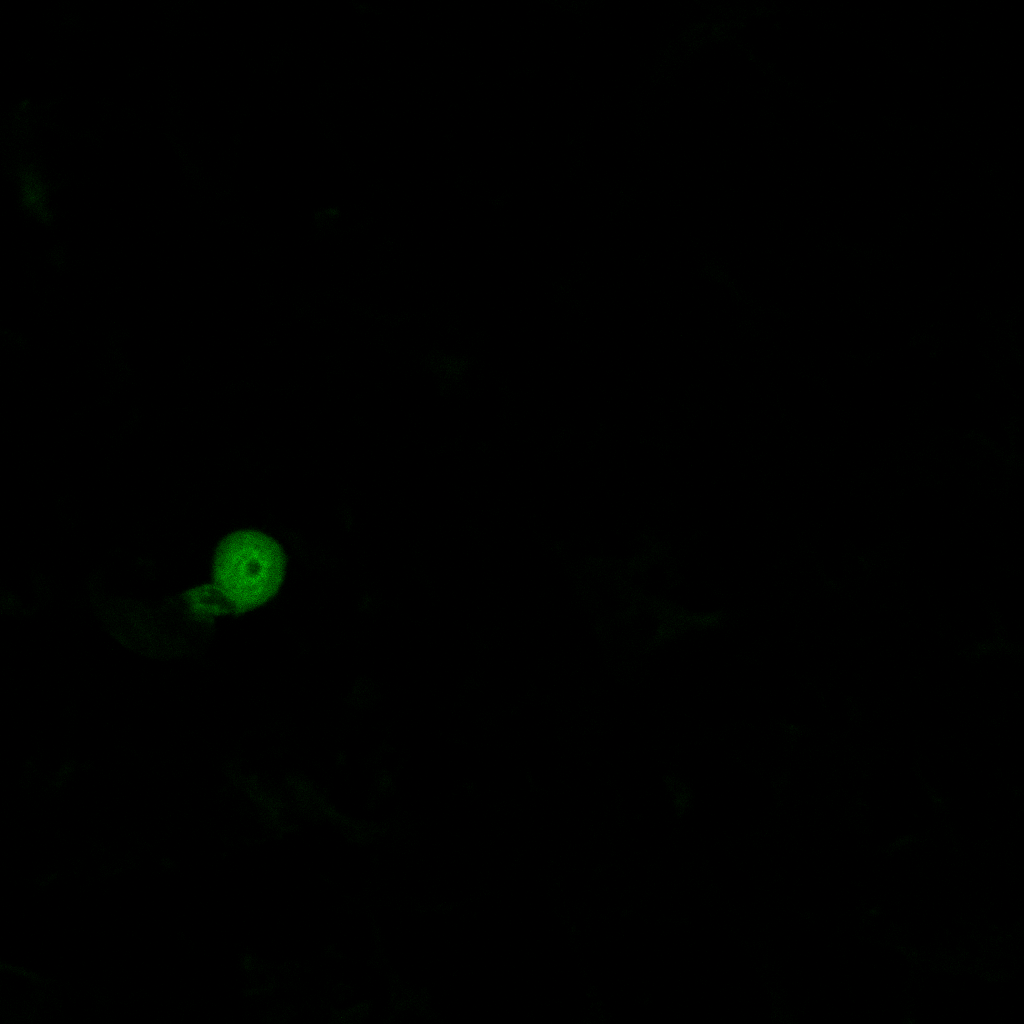

Supplement: Supplementary file 16 [file Data_Sheet_13.ZIP › original microscopy Supplementary Figure 2/KO/tublin fitc mitotracker red .lif_Series034_z0_ch01.tif]

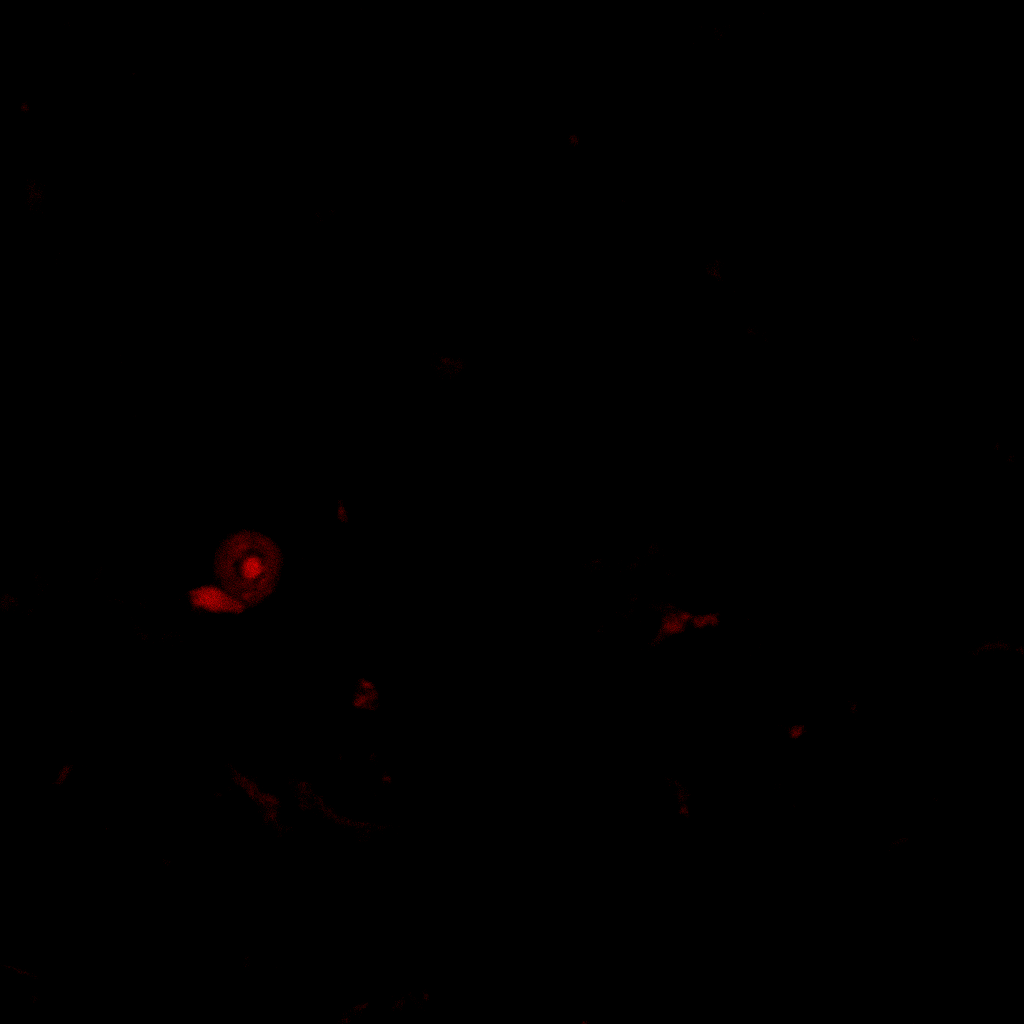

Supplement: Supplementary file 16 [file Data_Sheet_13.ZIP › original microscopy Supplementary Figure 2/KO/tublin fitc mitotracker red .lif_Series034_z0_ch02.tif]

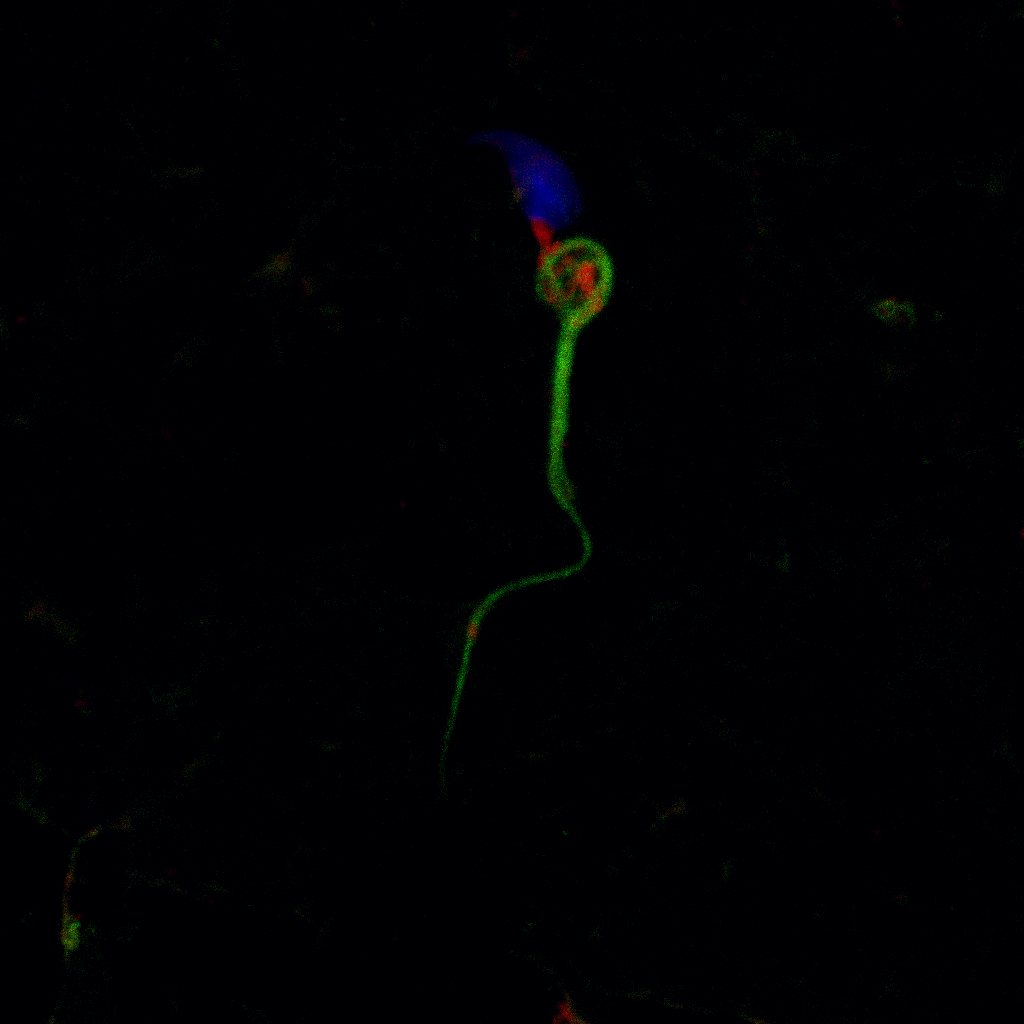

Supplement: Supplementary file 16 [file Data_Sheet_13.ZIP › original microscopy Supplementary Figure 2/KO/tublin fitc mitotracker red .lif_Series040_Processed001.tif]

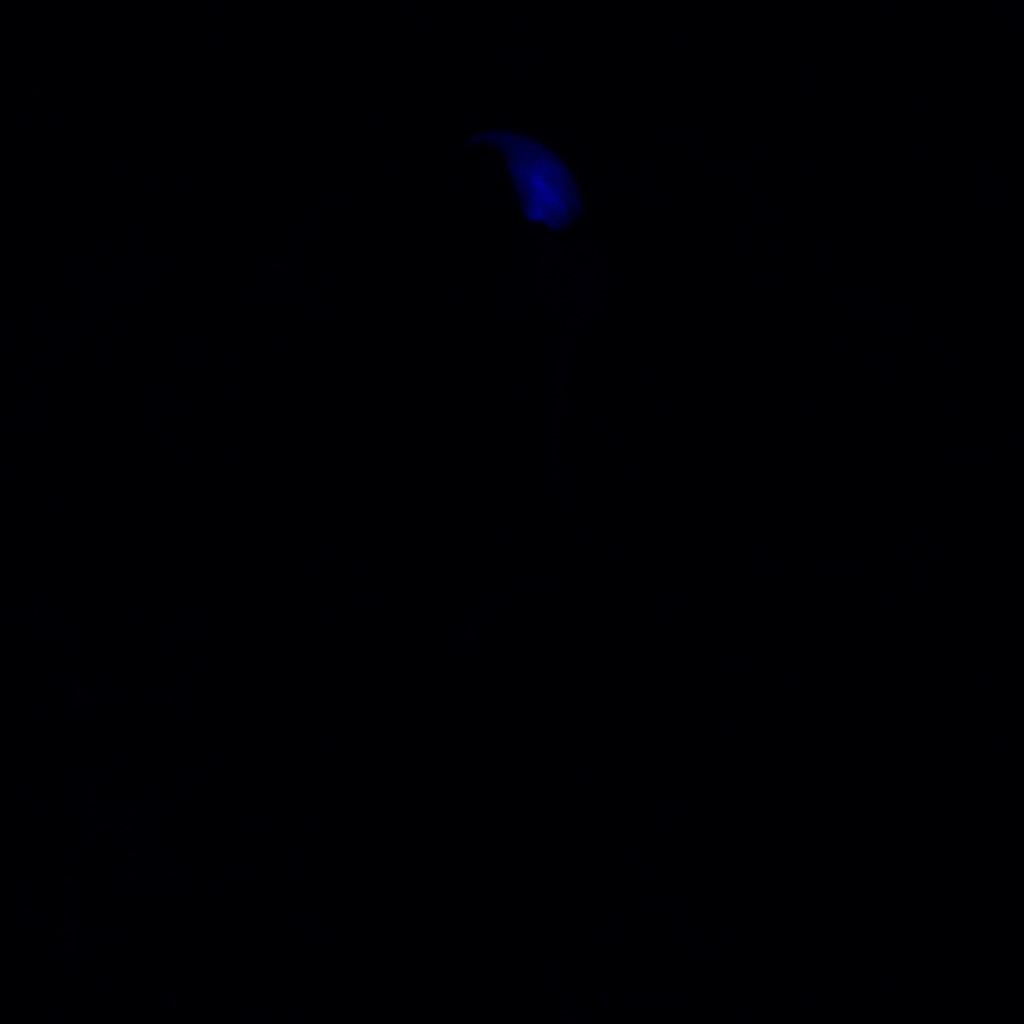

Supplement: Supplementary file 16 [file Data_Sheet_13.ZIP › original microscopy Supplementary Figure 2/KO/tublin fitc mitotracker red .lif_Series040_Processed001_ch00.tif]

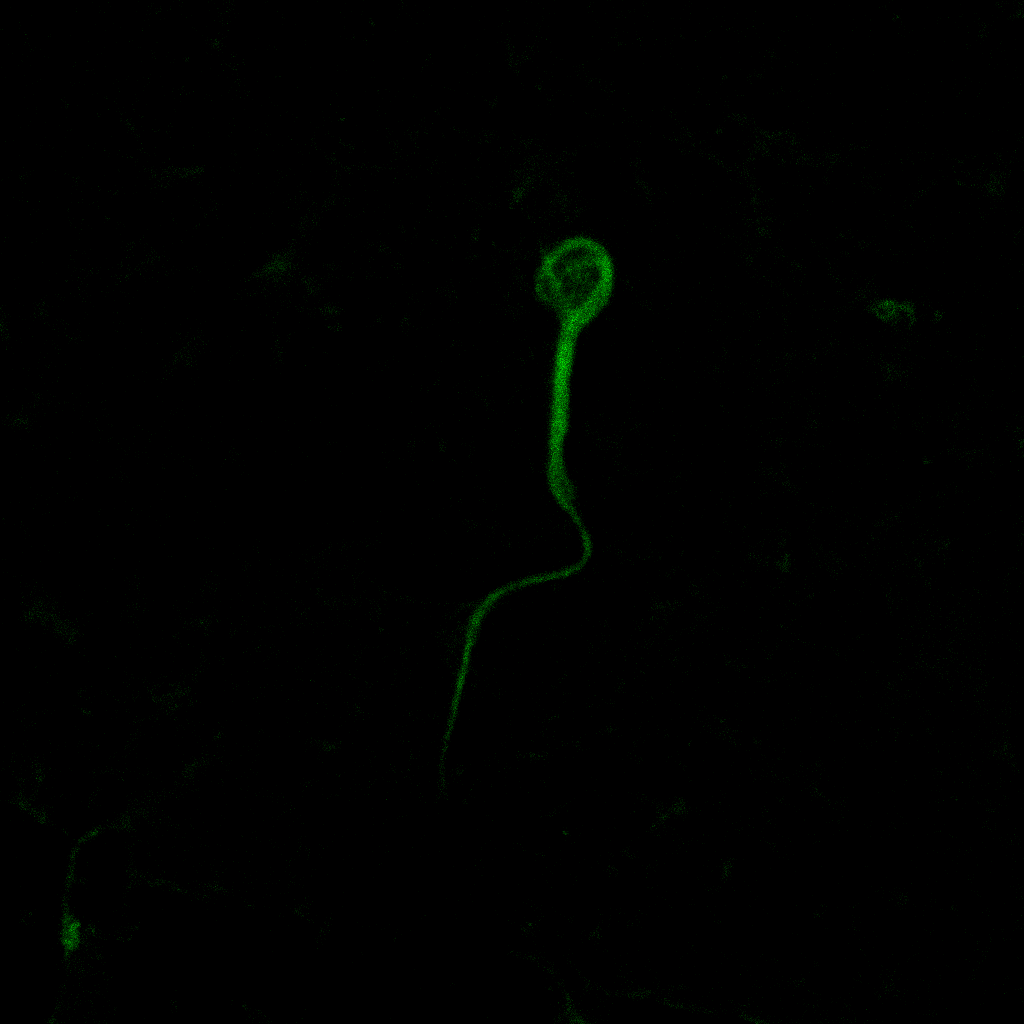

Supplement: Supplementary file 16 [file Data_Sheet_13.ZIP › original microscopy Supplementary Figure 2/KO/tublin fitc mitotracker red .lif_Series040_Processed001_ch01.tif]

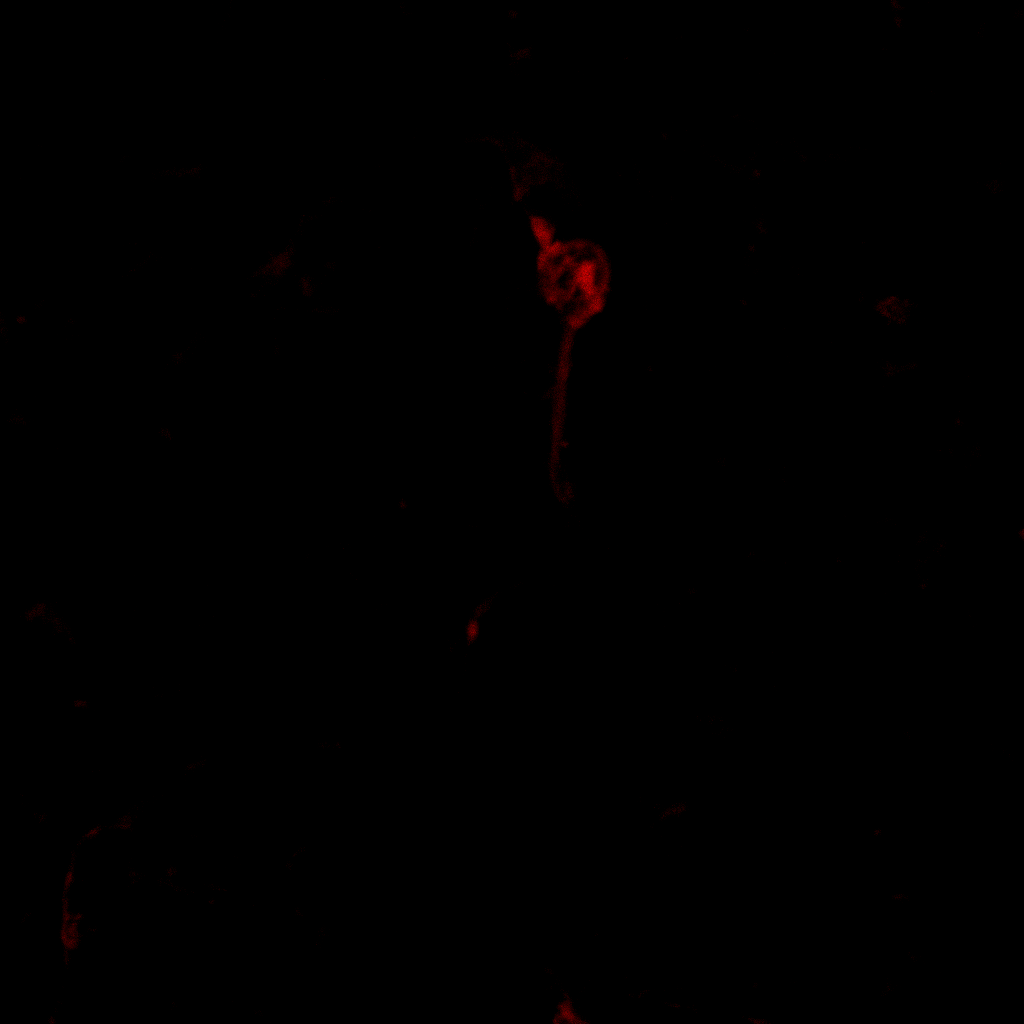

Supplement: Supplementary file 16 [file Data_Sheet_13.ZIP › original microscopy Supplementary Figure 2/KO/tublin fitc mitotracker red .lif_Series040_Processed001_ch02.tif]

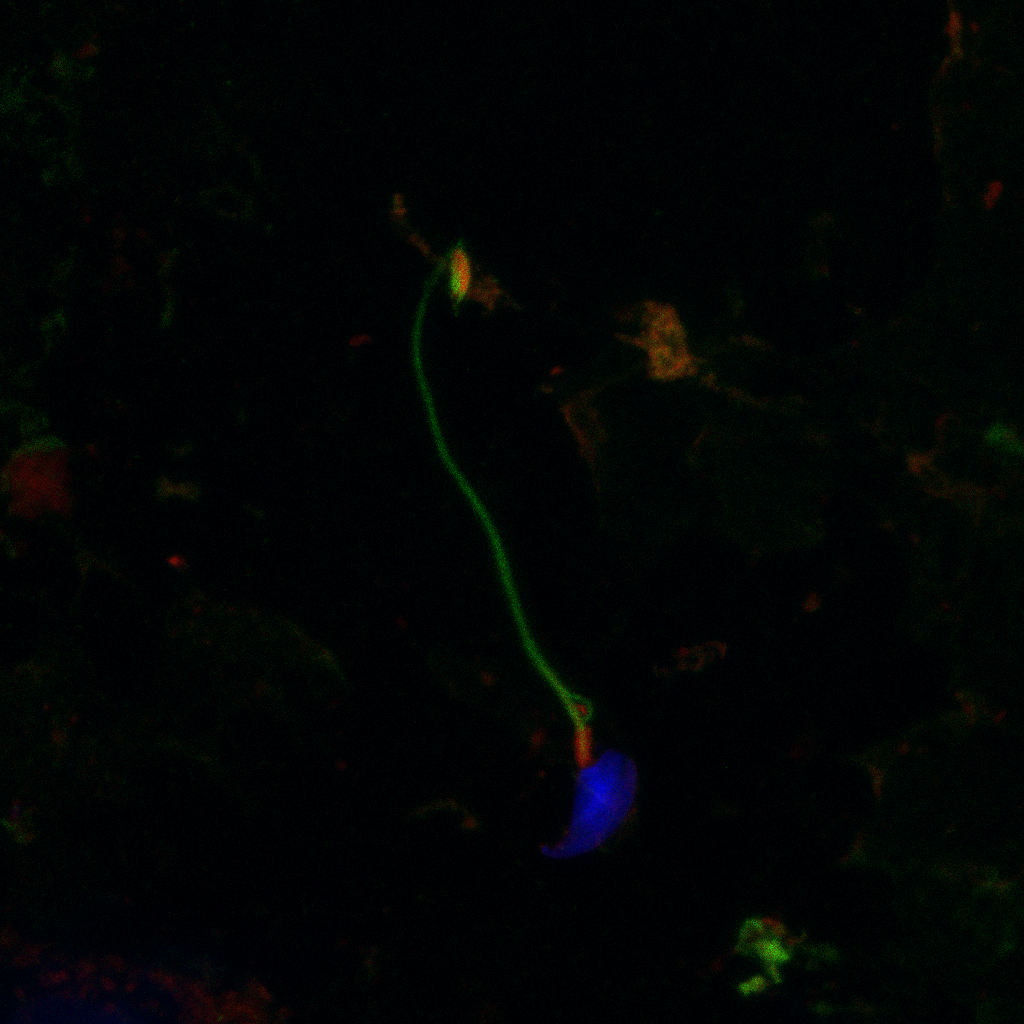

Supplement: Supplementary file 16 [file Data_Sheet_13.ZIP › original microscopy Supplementary Figure 2/KO/tublin fitc mitotracker red .lif_Series048_Processed001.tif]

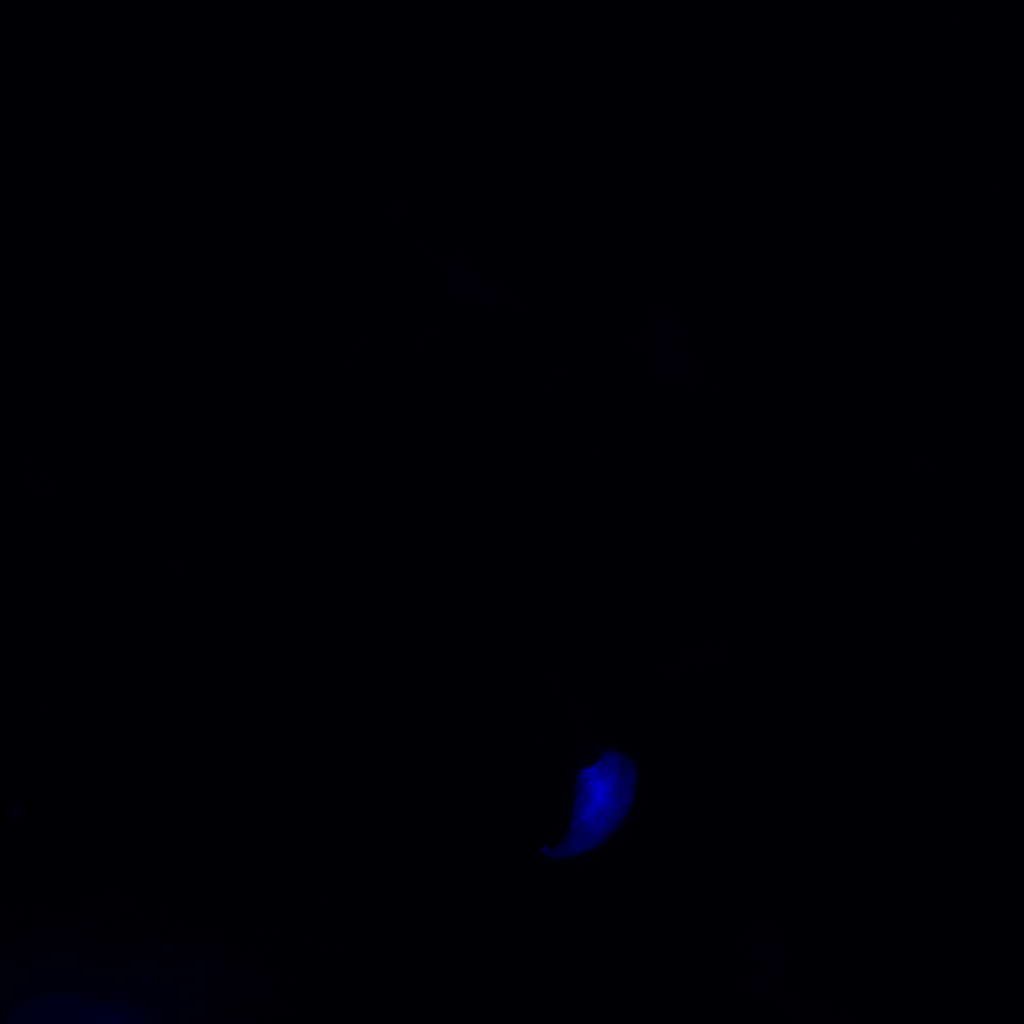

Supplement: Supplementary file 16 [file Data_Sheet_13.ZIP › original microscopy Supplementary Figure 2/KO/tublin fitc mitotracker red .lif_Series048_Processed001_ch00.tif]

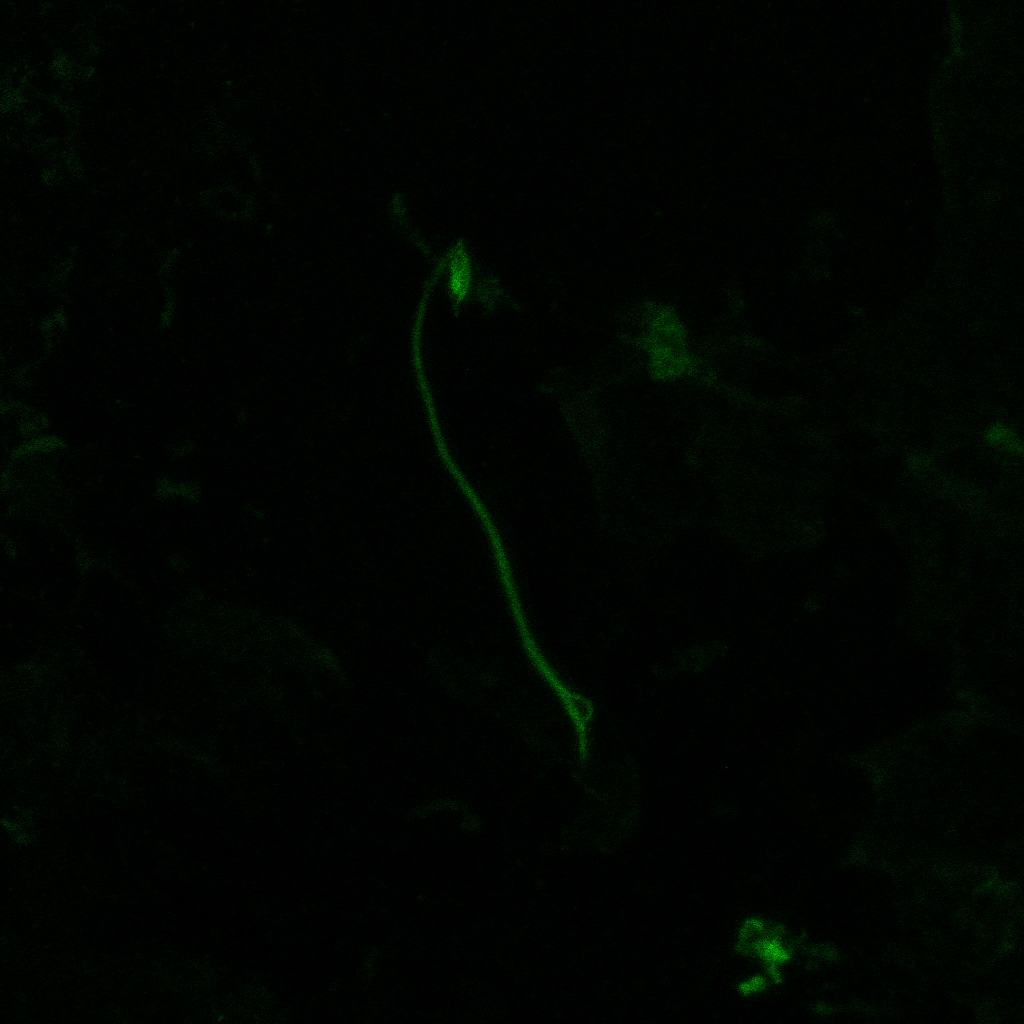

Supplement: Supplementary file 16 [file Data_Sheet_13.ZIP › original microscopy Supplementary Figure 2/KO/tublin fitc mitotracker red .lif_Series048_Processed001_ch01.tif]

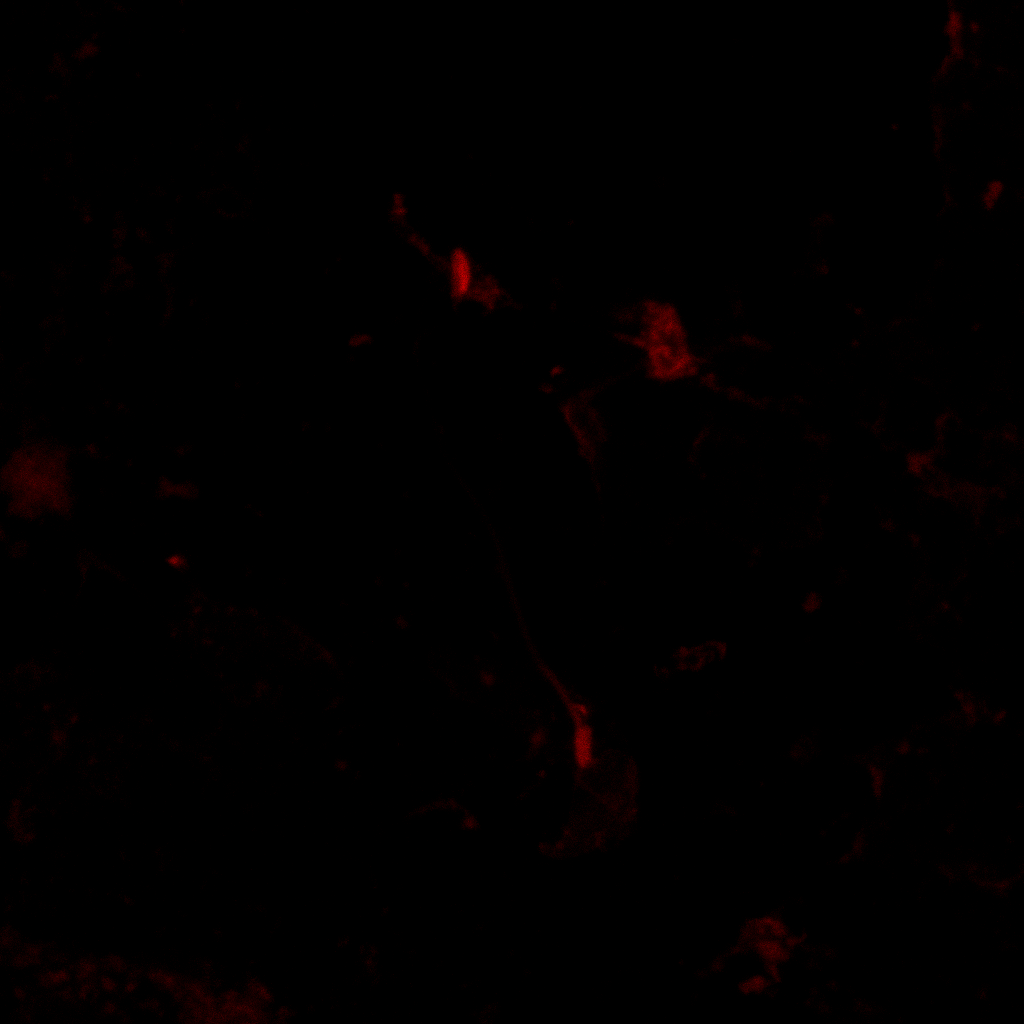

Supplement: Supplementary file 16 [file Data_Sheet_13.ZIP › original microscopy Supplementary Figure 2/KO/tublin fitc mitotracker red .lif_Series048_Processed001_ch02.tif]

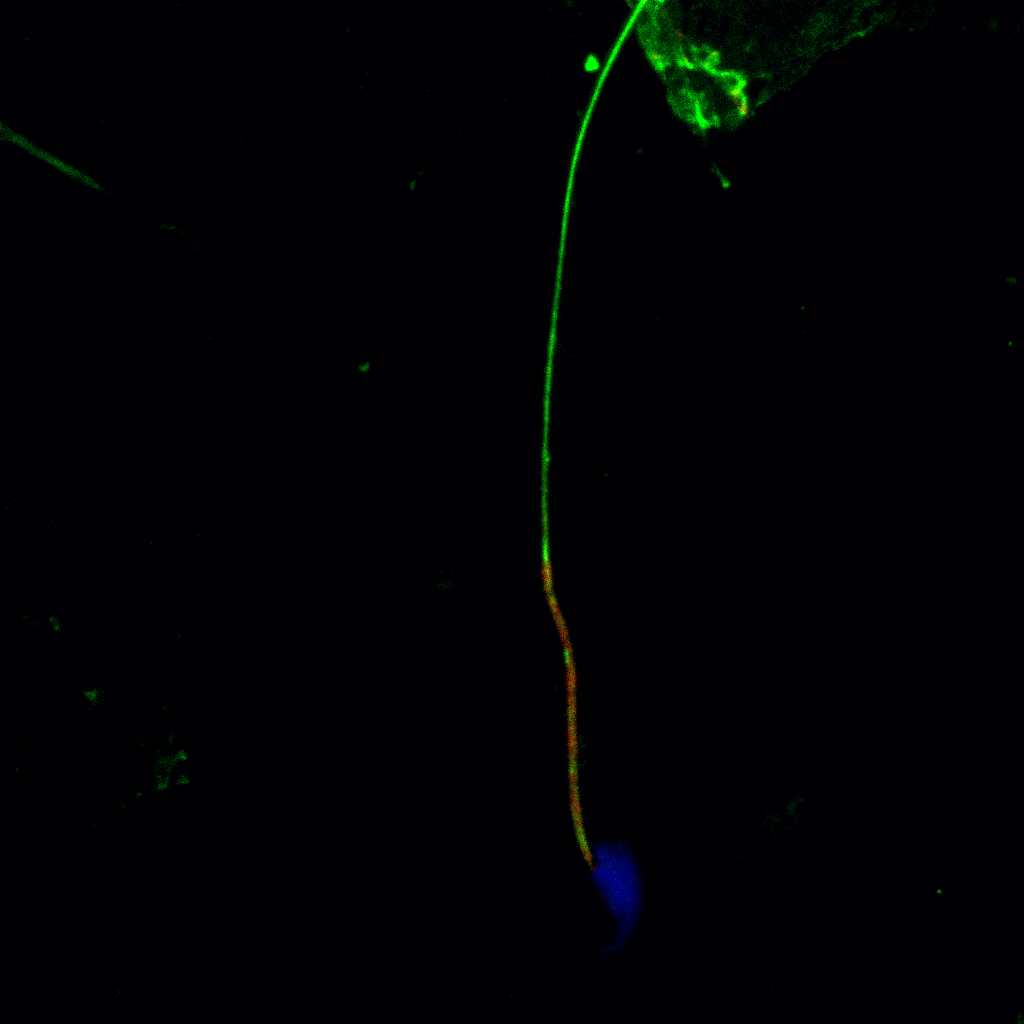

Supplement: Supplementary file 16 [file Data_Sheet_13.ZIP › original microscopy Supplementary Figure 2/WT/tublin fitc mitotracker red .lif_Series011_Processed001.tif]

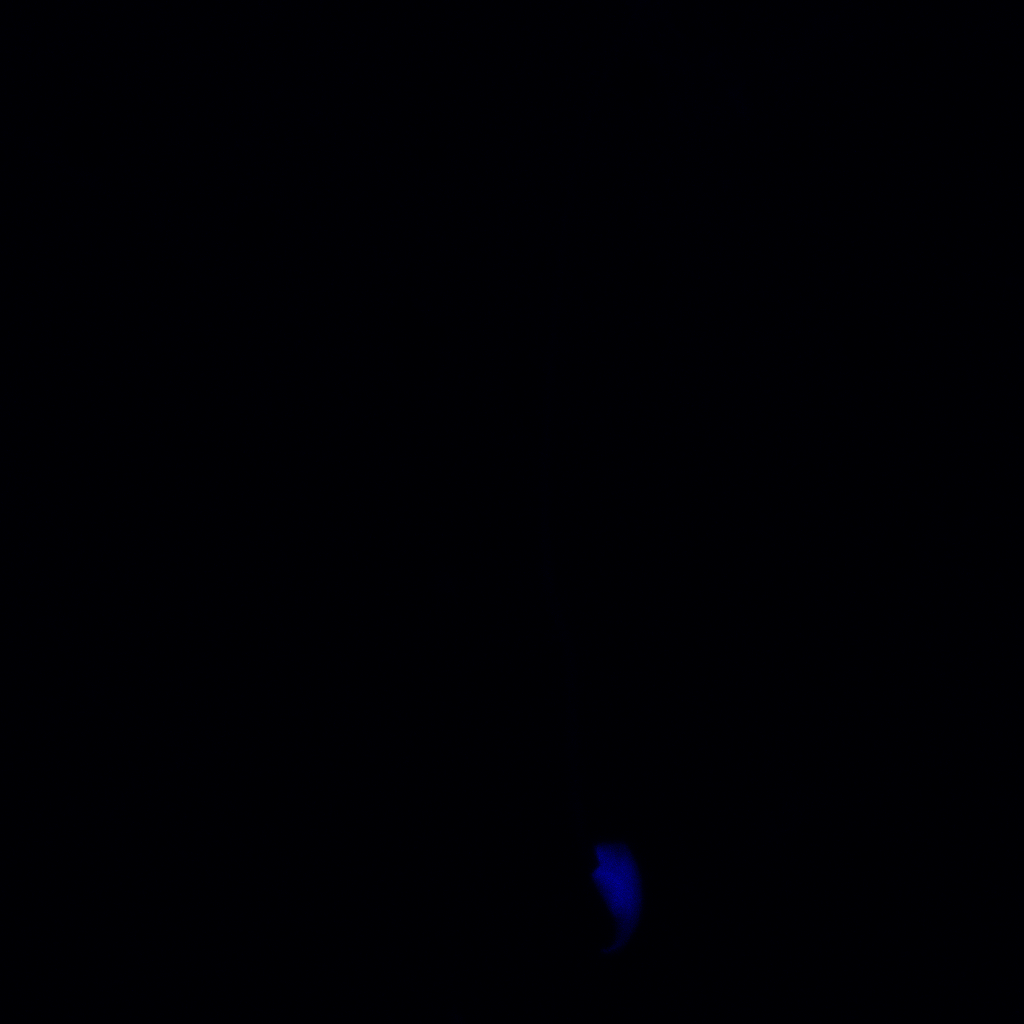

Supplement: Supplementary file 16 [file Data_Sheet_13.ZIP › original microscopy Supplementary Figure 2/WT/tublin fitc mitotracker red .lif_Series011_Processed001_ch00.tif]

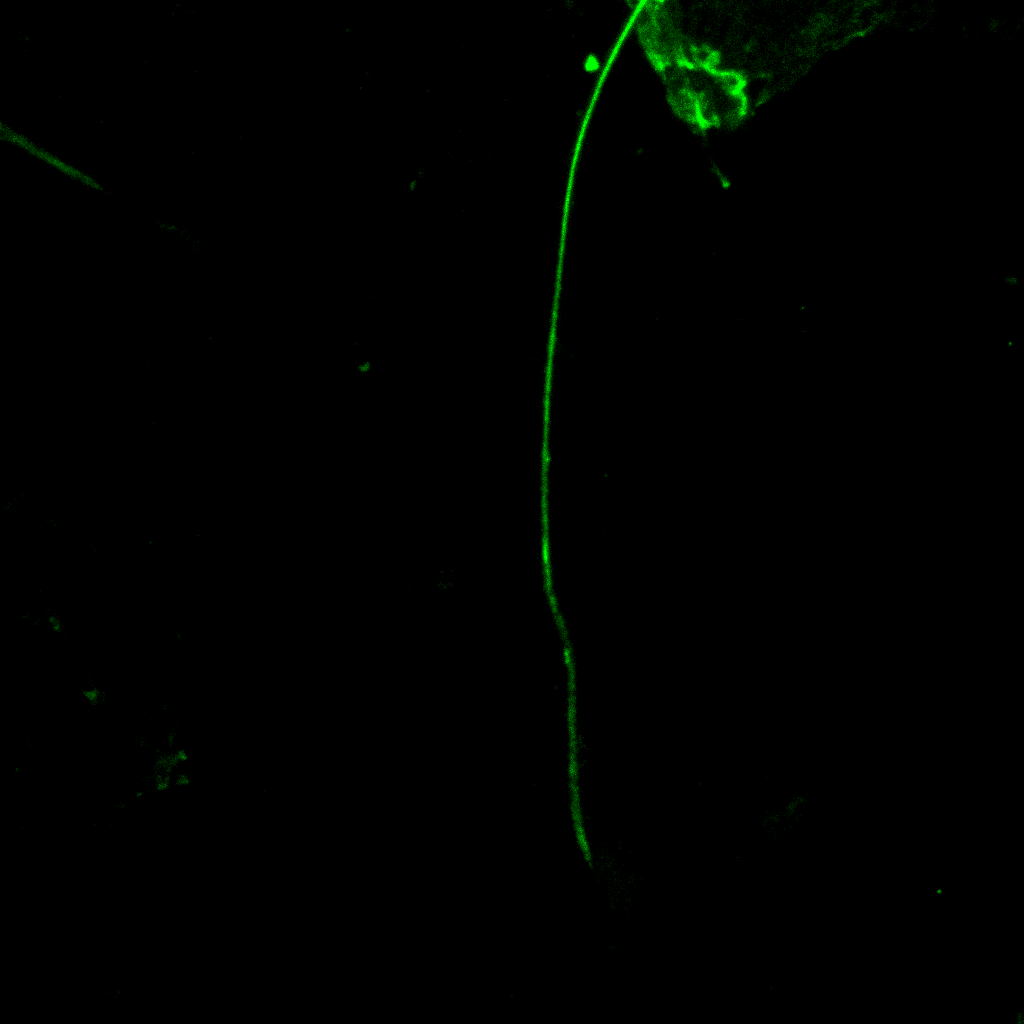

Supplement: Supplementary file 16 [file Data_Sheet_13.ZIP › original microscopy Supplementary Figure 2/WT/tublin fitc mitotracker red .lif_Series011_Processed001_ch01.tif]

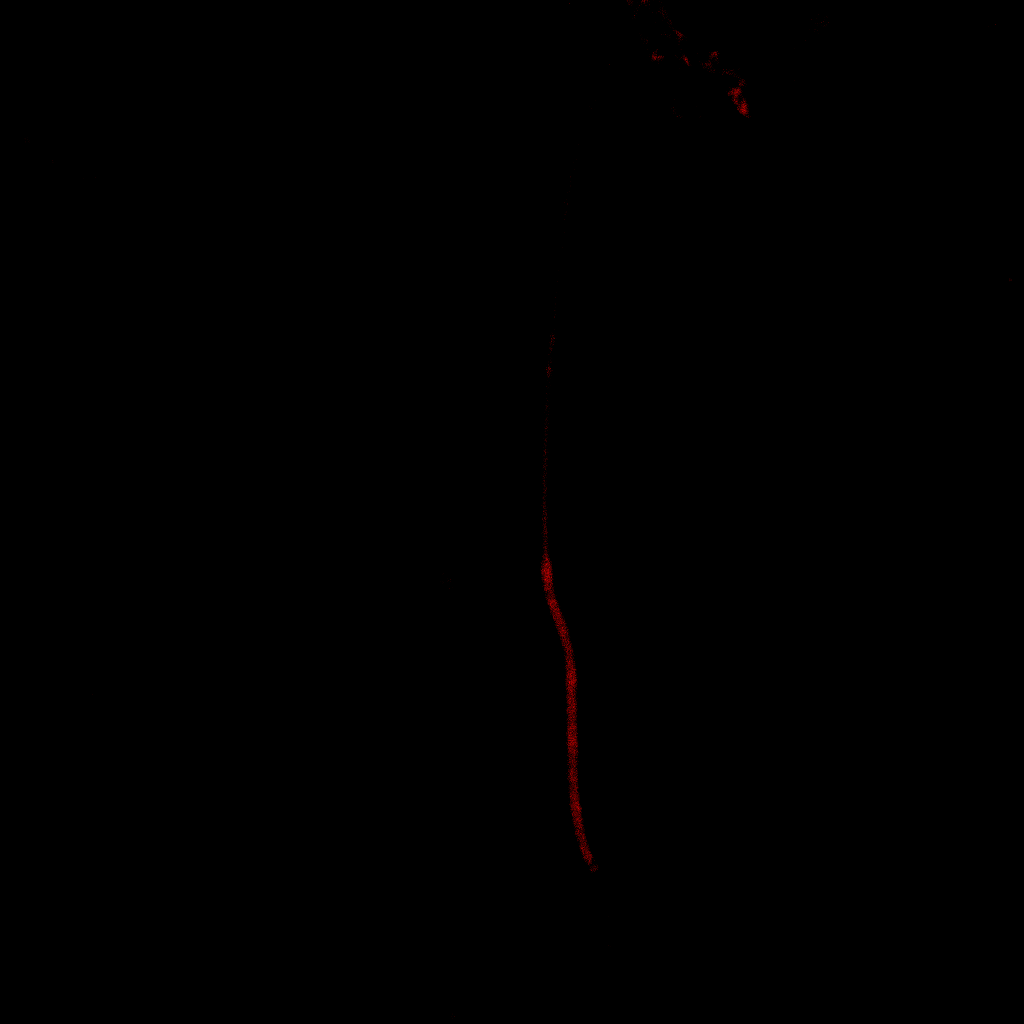

Supplement: Supplementary file 16 [file Data_Sheet_13.ZIP › original microscopy Supplementary Figure 2/WT/tublin fitc mitotracker red .lif_Series011_Processed001_ch02.tif]
